# Association mapping for important agronomic traits in wild and cultivated *Vigna* species using cross-species and cross-genera simple sequence repeat markers

**DOI:** 10.3389/fgene.2022.1000440

**Published:** 2022-11-03

**Authors:** Gita Kumari, P. S. Shanmugavadivel, G. Roopa Lavanya, Pravin Tiwari, Dharmpal Singh, P. G. Gore, Kuldeep Tripathi, Ramakrishnan Madhavan Nair, Sanjeev Gupta, Aditya Pratap

**Affiliations:** ^1^ ICAR-Indian Institute of Pulses Research, Kanpur, India; ^2^ Sam Higginbottom University of Agricultural Technology and Sciences, Prayagraj, India; ^3^ ICAR-National Bureau of Plant Genetic Resources, New Delhi, India; ^4^ World Vegetable Centre, ICRISAT Campus, Patancheru, India

**Keywords:** *Vigna*, *V*. *radiata*, cross-species SSR, genetic diversity, association study

## Abstract

The genus *Vigna* is an agronomically important taxon, with many of its species inhabiting a wide range of environments and offering numerous useful genes for the improvement of the cultivated types. The present study aimed to detect the genomic regions associated with yield-attributing traits by genome-wide association mapping. A diverse panel of 98 wild and cultivated *Vigna* accessions (acc.) belonging to 13 different species was evaluated for yield and related traits during the *kharif* season of 2017 and 2018. The panel was also genotyped using 92 cross-genera and cross-species simple sequence repeat markers to study the population genetic structure and useful market-trait associations. The PCA and trait correlation established relationships amongst the traits during both seasons while 100-seed weight (HSW) had a positive correlation with pod length (PL), and days to first flowering (DFF) with days to maturity (DM). The population genetic structure analysis grouped different acc. into three genetically distinct sub-populations with SP-1 comprising 34 acc., SP-2 (24 acc.), and SP-3 (33 acc.) and one admixture group (7 acc.). Mixed linear model analysis revealed an association of 13 markers, namely, VR018, VR039, VR022, CEDG033, GMES0337, MBSSR008, CEDG220, VM27, CP1225, CP08695, CEDG100, CEDG008, and CEDG096A with nine traits. Seven of the aforementioned markers, namely, VR018 for plant height (PH) and terminal leaflet length (TLL), VR022 for HSW and pod length (PL), CEDG033 for DFF and DM, MBSSR008 for DFF and DM, CP1225 for CC at 30 days (CC30), DFF and DM, CEDG100 for PH and terminal leaflet length (TLL), and CEDG096A for CC30 and chlorophyll content at 45 days were associated with multiple traits. The marker CEDG100, associated with HSW, PH, and TLL, is co-localized in gene-encoding histone–lysine N-methyltransferase *ATX5*. Similarly, VR22, associated with PL and HSW, is co-located in gene-encoding *SHOOT GRAVITROPISM 5* in mungbean. These associations may be highly useful for marker-assisted genetic improvement of mungbean and other related *Vigna* species.

## 1 Introduction

The genus *Vigna* is an important taxon playing a prominent role in food and nutritional security and environmental sustainability. Several members of this genus are known to inhabit a wide range of agro-ecological regions across the globe including farmlands in tropical and subtropical regions, pastures, sandy beaches, mountain cliffs, roadsides, and field bunds. A few species are also found inhabiting extreme climates, thereby possessing special adaptive genes (Takahashi et al., 2016); therefore, these can serve as a potential source for yield- and adaptation-related traits (Pratap et al., 2015; Pratap, 2021). Most members of this genus are diploid with 2n = 2x = 22 having an exception of *V. glabrescens* which is a tetraploid (2n = 4x = 44). This genus is characterized by five sub-genera, namely, *Haydonia*, *Lasiospron*, *Ceratropis*, *Plectotropis*, and *Vigna*. Among these, the crop species have been developed from the later three sub-genera. *Ceratotropis*, also known as the Asiatic *Vigna*, is the most important in terms of most of the agronomic species categorized in this group. The agronomic species in the genus *Vigna* are autogamous and cross-pollination does not occur in nature.

India has been bestowed with an abundance of genetic diversity of *Vigna* including its cultivated, wild, and weedy types (Bisht et al., 2005; Pratap et al., 2015). It is the largest producer, processor, consumer, and importer of the two most important members of this group, the mungbean (*V. radiata* L. emWilczek) and the urdbean (*V. mungo* L. Hepper). The country is also considered as the region of the first domestication of important cultivated *Vigna* species including mungbean, urdbean (Baudoin and Marechal, 1988), ricebean (de Candolle, 1886), and mothbean (Smartt, 1985), while the progenitors of mungbean and urdbean, namely, *V. sublobata* and *V. sylvestris*, are found in abundance in cultivated as well as waste lands (Chandel et al., 1984; Lawn and Cottrell, 1988). Fortunately, the large variability of *Vigna* species spread across the Indian subcontinent has been collected extensively and conserved in the National Gene Banks at the ICAR-National Bureau of Plant Genetic Resources, New Delhi (ICAR-NBPGR), and ICAR-Indian Institute of Pulses Research, Kanpur (ICAR-IIPR). Most of these accessions have been studied and characterized at the morphological and molecular levels (Pratap et al., 2015; Pratap et al., 2017; Gore et al., 2019; Pratap et al., 2021b; Gore et al., 2021; Kumari et al., 2021), and many of them including *V. radiata*, *V. mungo*, *V. umbellata*, *V. sylvestris*, and *V. trilobata* have been deployed in hybridization programs for the genetic improvement of the cultivated types. A few of the wild relatives of *Vigna* are also available for neo-domestication (Gore et al., 2019). Some of them are valued as forage, cover, and green manure crops (Kumari et al., 2021). Many species also confer numerous valuable genes for agronomic, stress resistance, and seed quality traits (Pratap et al., 2015; Douglas et al., 2020; Pratap et al., 2020), namely, resistance/tolerance to drought and waterlogging (Bisht et al., 2005; Tomooka et al., 2006), soil salinity (Chankaew et al., 2014; Yoshida et al., 2016), heat and cold stress (HanumanthaRao et al., 2016), acidic or alkaline soils (Soares et al., 2014), bruchids (Aidbhavi et al., 2021), Cercospora leaf spot (Chankaew et al., 2011; Singh et al., 2017), and yellow mosaic disease [for a review, please see (Singh et al., 2020)] in addition to superior agronomic traits and photo-thermo period insensitivity (Pratap et al., 2014b; Basu et al., 2019). Extensive efforts have been undertaken for their genetic improvement and methods such as hybridization, selection, and mutation have been abundantly deployed in addition to pre-breeding and distant hybridization (Singh et al., 2017; Pratap et al., 2021b).

Information on the diversity at the genetic level, population genetic structure, and marker–trait association for useful traits provides useful information for deploying marker-assisted breeding for targeted and time-bound genetic improvement of crop plants. Information on the association of these traits with seed yield is of utmost importance to undertake a selective breeding program toward developing a desirable combination of yield-contributing traits. In this direction, the genome-wide association study (GWAS) is a high-resolution method for genetic mapping of traits using existing germplasm and their phenotypic information for the trait concerned (Flint-Garcia et al., 2003). It also helps us understand the genetic basis of complex traits and allows studying a wide range of alleles at each locus and the identification of useful marker–trait associations at the whole-genome level in addition to the identification of elite alleles for significantly associated loci (Rohilla et al., 2021). The higher mapping resolution of traits from association mapping provides an opportunity for the adoption of MAS in crop breeding programs (Mackay and Powell, 2007). Nonetheless, very few reports are available which document genetic diversity in *Vigna* species at the molecular level, while no report is available on useful marker–trait associations in wild and diverse *Vigna* accessions. The present study aimed to evaluate the genetic diversity and marker–trait associations in an extensive panel of wild and cultivated *Vigna* species genotyped with cross-genera and cross-species SSR markers so as to identify useful marker–trait associations which could be effectively deployed not only in mungbean improvement programs but also in other related *Vigna* crops such as urdbean which lack genomic information.

## 2 Materials and methods

### 2.1 Plant materials

The plant materials for this study comprised 98 genotypes belonging to cultivated (13 accessions) and wild (85 accessions) *Vigna* species (Supplementary Table S1). Species-wise, the accessions (acc.) belonged to *V. umbellata* (16 acc.), *V. trilobata* (20 acc.), *V. mungo* var. *mungo* (9 acc.), *V. radiata* (6 acc.), *V. radiata* var. *radiata* (6 acc.), *V. radiata* var. *sublobata* (6 acc.), *V. silvestris* (4 acc.), *V. unguiculata* (4 acc.), *V. dalzelliana* and *V. trinervia* var. *bourneae* (3 acc. each), *V. radiata* var. *setulosa*, *V. stipulaceae* and *V. vexiliata* (2 acc. each), and *V. trinervia* and *V. glabrescenc*e (1 acc. each). All the accessions were collected under the “National Exploration Plan” coordinated by the ICAR-NBPGR, New Delhi, which is the nodal institute at the national level for the collection and conservation of plant genetic resources in India, supported by the ICAR-IIPR, Kanpur, and other institutes of the Indian Council of Agricultural Research (ICAR). A team of experts comprising taxonomists/botanists and crop breeders undertook different exploration missions to collect these wild accessions over the years and identified each accession. Subsequently, seed samples of all collections were multiplied and deposited in the National Genebank housed at the ICAR-NBPGR and are available freely to all researchers nationally and globally as per national legislation. As evidenced from previous morphological analysis as well as the literature available, this panel of genotypes represented a wide range of genetic diversity to represent important recombination events as well as high genetic diversity.

### 2.2 Phenotyping of the selected panel

All the *Vigna* genotypes were grown in natural field conditions in the wide hybridization garden in an augmented design during *Kharif* (rainy season) 2017 and 2018 at the Main Research Farm, ICAR-IIPR, Kanpur, India. Kanpur is located at 26°27′N latitude, 80°14′E longitude, 152.4 m above mean sea level (amsl) and experiences tropical climate with a long-term mean annual rainfall of 820 mm. (Annual report, 2019). About 80% of the total rainfall is received during the southwest monsoon season (July–September). The recommended package of agronomic practices for growing mungbean in the region was followed to raise healthy plants. The phenotypic data were recorded on five random plants of each genotype at the specified stage of plant growth, and these were averaged for use in the downstream analysis. The traits included chlorophyll content at 30 days (CC30) and 45 days (CC45), measured using a SPAD photometer from five top leaves in five random plants of each accession, averaged over all samples; days to first flowering (DFF); days to first pod maturity (DM); plant height in cm (PH); peduncle length in cm (PEDLTH); pod length in cm (PL); terminal leaflet length in cm (TLL); number of seeds per plant (NSPP); and 100-seed weight in g (HSW).

### 2.3 Phenotypic data analysis

The phenotypic data were analyzed for the descriptive statistics (R package = “pastecs”), and the linear mixed model approach was followed to calculate BLUP values for each genotype using the R package (“lme4”). The BLUP values were considered for determining the strength of a relationship between variables following trait correlation (R packages = “Corrplot” and “PerformanceAnalytics”) and principal component analysis (PCA) of the traits (R packages = “factoxtra” and “FactoMineR”).

### 2.4 Genotyping the panel with cross-genera and cross-species simple sequence repeats

The total genomic DNA was extracted from fresh young leaves of each accession at the early vegetative stage (within 10–12 days of sowing) following the CTAB method (Doyle and Doyle, 1990) with slight modifications (Pratap et al., 2015). The quality of the extracted DNA was analyzed on 0.8% agarose gel and the quantity was determined using a Nanodrop spectrophotometer ND 1000 (Nanodrop Technologies, DE, United States). The DNA of each sample was normalized to a concentration of 20–30 ng/μL. The panel was genotyped with 92 polymorphic SSRs belonging to different *Vigna* species namely, cowpea (Li et al., 2001), adzuki bean (Wang et al., 2004), mungbean (Kumar et al., 2002; Somta et al., 2009), and common bean (Gaitan-Solis et al., 2002; Blair et al., 2003) (Supplementary Table S2). PCR amplification was carried out on a tetrad thermal cycler (G-strom, Somerset, United Kingdom) in a reaction volume of 20 μL containing 50–60 ng template DNA, 0.4 µL 10 mM dNTPs, 0.6 U of *Taq* DNA polymerase (Fermentas, Mumbai), 2 µL of 10X *Taq* buffer A (Fermentas, Mumbai) with MgCl_2_, and 5 pmole each of forward and reverse primers (ILS, India). PCR amplifications were performed at an initial denaturation of 95°C for 5 min, followed by 35 cycles of denaturation at 95°C for 15 s, primer-specific annealing for 15 s at 45–55°C, and extension at 72°C for 1 min followed by the final extension at 72°C for 5 min. The PCR products were resolved on 3.5% metaphor^®^agarose gel in 1X TAE buffer for 3–4 h at 80–100 V and stained with ethidium bromide. The gels were documented using a gel documentation system (Uvitech, Cambridge), and alleles were recorded on all genotypes according to their fragment sizes (in base pairs).

### 2.5 Population structure analysis

The Bayesian model-based STRUCTURE v2.3.4 tool was used to find the number of sub-populations (Q-matrix) in the selected *Vigna* association panel using 92 SSR markers (Pritchard et al., 2000). The genotypic data were analyzed with 10 independent runs for each cluster (*K*), ranging from 1 to 10 by setting the burn-in period of 30,000 and the number of Markov chain Monte Carlo iterations of 60,000 along with the admixture model and correlated allele frequencies. The optimum number of sub-populations (k) was determined using Structure Harvester Web v0.6.94 (Earl and Vonholdt, 2012) based on the ad-hoc criterion (Delta *K*) (Evanno et al., 2005).

### 2.6 Analysis of linkage disequilibrium between markers and marker–trait association

The software program TASSEL v2.0.1 (Bradbury et al., 2007) was used to evaluate linkage disequilibrium between the studied markers and marker–trait associations (MTAs) using the genotypic and 2 years’ phenotypic data following the general linear model (GLM with Q-matrix) and mixed linear model (MLM with Q + K matrix). The Q-matrix derived from the STRUCTURE program and the relative kinship matrix calculated by TASSEL software was used for GLM and MLM analyses. The number of permutation runs in GLM was set to 10,000 to obtain a marker significance value of corrected *p* < 0.00001 (at alpha = 0.001 and *n* = 92) to declare MTAs. MLM with Q + K matrix was analyzed following default run parameters, namely, convergence criterion of 1.0 × 10^−4^, and the maximum number of iterations was set to 200. Significant MTAs were declared at alpha 0.05, and corrected *p*-value of ≤0.00054 with relative magnitude was represented by the *R* ^2^ value as the portion of variation explained by the marker.

### 2.7 Localization of markers associated with traits on the *V. radiata* genome

Primer sequences of all associated SSRs were BLAST searched against the *V. radiata* var*. radiata* (tax id: 3,916) genome and searched for their 100% identity to find their exact physical position in a genome. The corresponding physical position of each SSR was used to extract the nucleotide sequences to search and verify the presence of the SSR motif flanking the primer region following the SSRIT tool (Temnykh et al., 2001) and searched for the co-localized or flanking genes.

## 3 Results

### 3.1 Descriptive statistics

The selected *Vigna* panel revealed wide phenotypic variations for all the 10 traits in both growing seasons (Table 1). The highest coefficient of variation (CV) was observed for HSW (83%) followed by PH (65%) and DFF (52%). The lowest CV was observed for CC45 (19%) and CC30 (15%). Data from both seasons were comparable to each other, and not much deviation was observed.

**TABLE 1:** Descriptive statistics of quantitative traits.

Descriptive statistics	PH		CC30		CC45		TLL		PEDLTH		DFF		DM			PL		NSPP		HSW	
	2017	2018	2017	2018	2017	2018	2017	2018	2017	2018	2017	2018		2017	2018	2017	2018	2017	2018	2017	2018
Minimum	3.2	5.8	3.86	25.59	26.84	28.65	3.2	2.6	5.8	5.9	26	25		35	37	2.64	2.62	3.58	3.56	0.596	0.65
Maximum	144.1	197.2	52.84	60.3	63.32	67.16	12	11.6	34.6	34.6	132	129		56	151	16.5	11.56	13.8	13.4	19.927	20.7
Range	140.9	191.4	28.98	34.71	36.48	38.51	8.8	9	28.8	28.7	106	104		121	114	13.86	8.94	10.22	9.84	19.331	20.05
Sum	4,768.31	5,714.6	3,756.28	4,008.94	4,079.7	4,081.22	800	744.9	1,486.94	1,563.6	4,478	4,336		6,401	6,303	549.21	554.165	855.88	855.93	287.807	291.42
Mean	48.65	58.31	38.33	40.91	41.63	41.65	8.16	7.6	15.17	15.96	45.69	44.25		65.32	64.31	5.6	5.65	8.73	8.73	2.94	2.97
SE mean	3.18	3.56	0.61	0.61	0.8	0.72	0.23	0.22	0.67	0.68	2.35	2.34		2.65	2.63	0.197	0.17	0.26	0.26	0.25	0.25
Var.	993.66	1,241.53	36.84	36.53	62.31	50.8	5.04	4.79	43.83	45.61	541.39	536.66		689.89	677.02	3.47	2.89	6.49	6.63	5.97	6.15
St. deviation	31.52	35.24	6.07	6.04	7.89	7.13	2.25	2.19	6.62	6.75	23.27	23.17		26.27	26.02	1.86	1.7	2.555	2.58	2.44	2.48
CV	0.65	0.6	0.16	0.15	0.19	0.17	0.28	0.29	0.44	0.42	0.51	0.52		0.4	0.41	0.33	0.3	0.29	0.29	0.83	0.83

Where PH: plant height in cm; CC30: chlorophyll content at 30 days; CC45: chlorophyll content at 45 days; DFF: days to first flowering; DM: days to first pod maturity; PH: plant height in cm; PEDLTH: peduncle length in cm; PL: pod length in cm; TLL: terminal leaflet length in cm; NSPP: number of seeds per plant; and HSW: 100-seed weight in g.

### 3.2 Correlation of traits with genotypic values obtained from the mixed model

**FIGURE 1:**
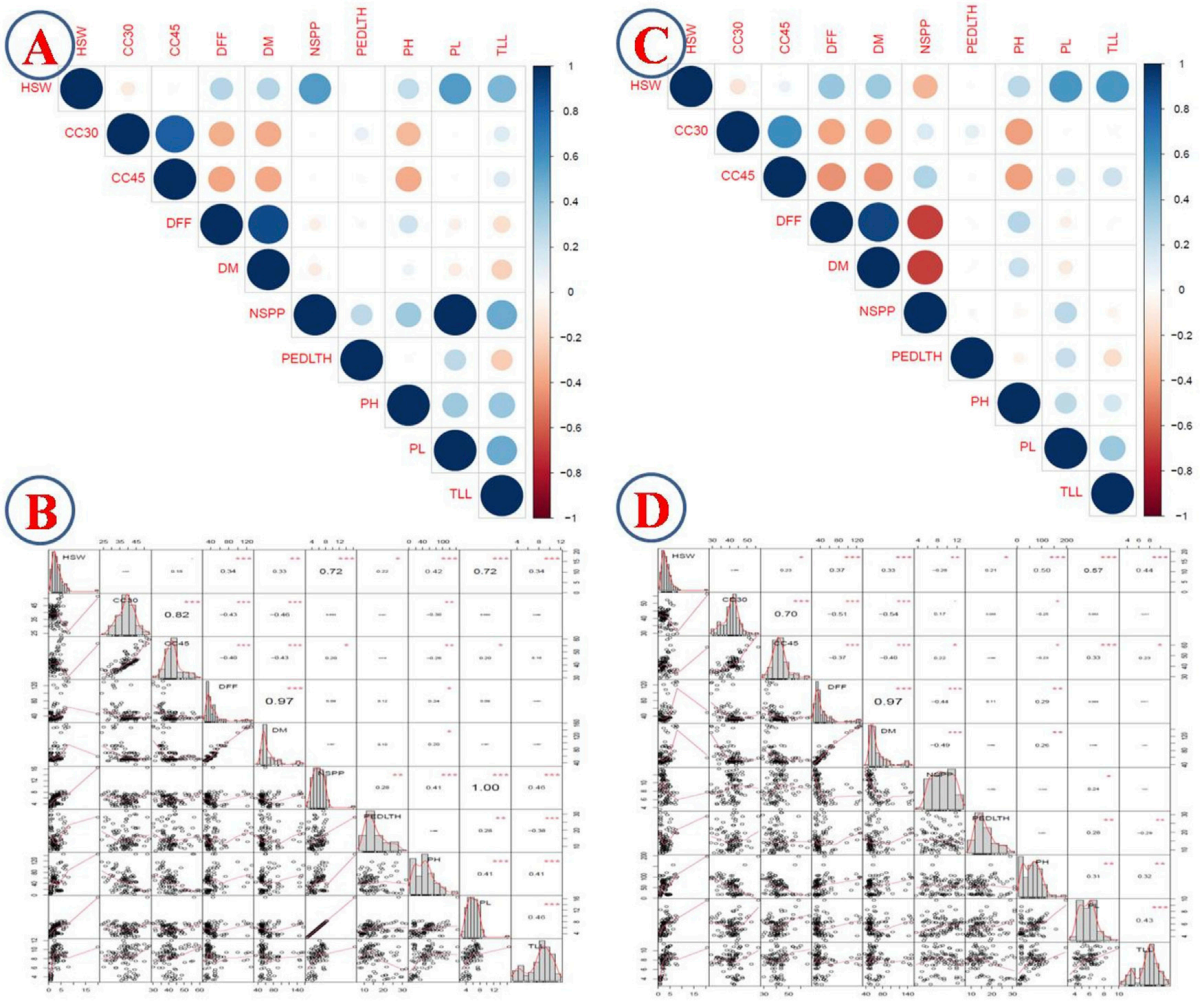
Correlation analysis of quantitative traits with BLUP values; **(A)** correlogram of 2017 data; **(B)** correlation table of 2017 data; **(C)** correlogram of 2018 data; **(D)** correlation table of 2018.

Correlation of traits with predicted genotype values of each genotype from the mixed model approach revealed a high positive correlation between NSPP and PL (1.0), DFF and DM (0.97), CC30 and CC45 (0.82), HSW and NSPP (0.72), and HSW and PL (0.72). HSW exhibited a significant positive correlation with most of the traits except CC30, CC45, and peduncle length. NSPP recorded a significant positive correlation with peduncle length, PH, TLL, and PL. PH, DFF, and DM traits revealed a negative correlation with CC45 and CC30. Likewise, PL recorded a significant negative correlation with TLL (−0.38) (Figure 1).

The predicted genotype values of each genotype with 2018 data revealed highly significant positive correlations between DFF and DM (0.97), CC30 and CC45 (0.7), and HSW and PL (0.57). On the contrary, PH, DFF, and DM revealed a negative correlation with CC45 and CC30 as in the 2017 data. Similarly, DFF and DM had a significant negative correlation with NSPP. PH, PL, and TLL revealed a positive correlation with each of these three traits (Figure 1).

### 3.3 Principal component analysis with BLUP

**FIGURE 2:**
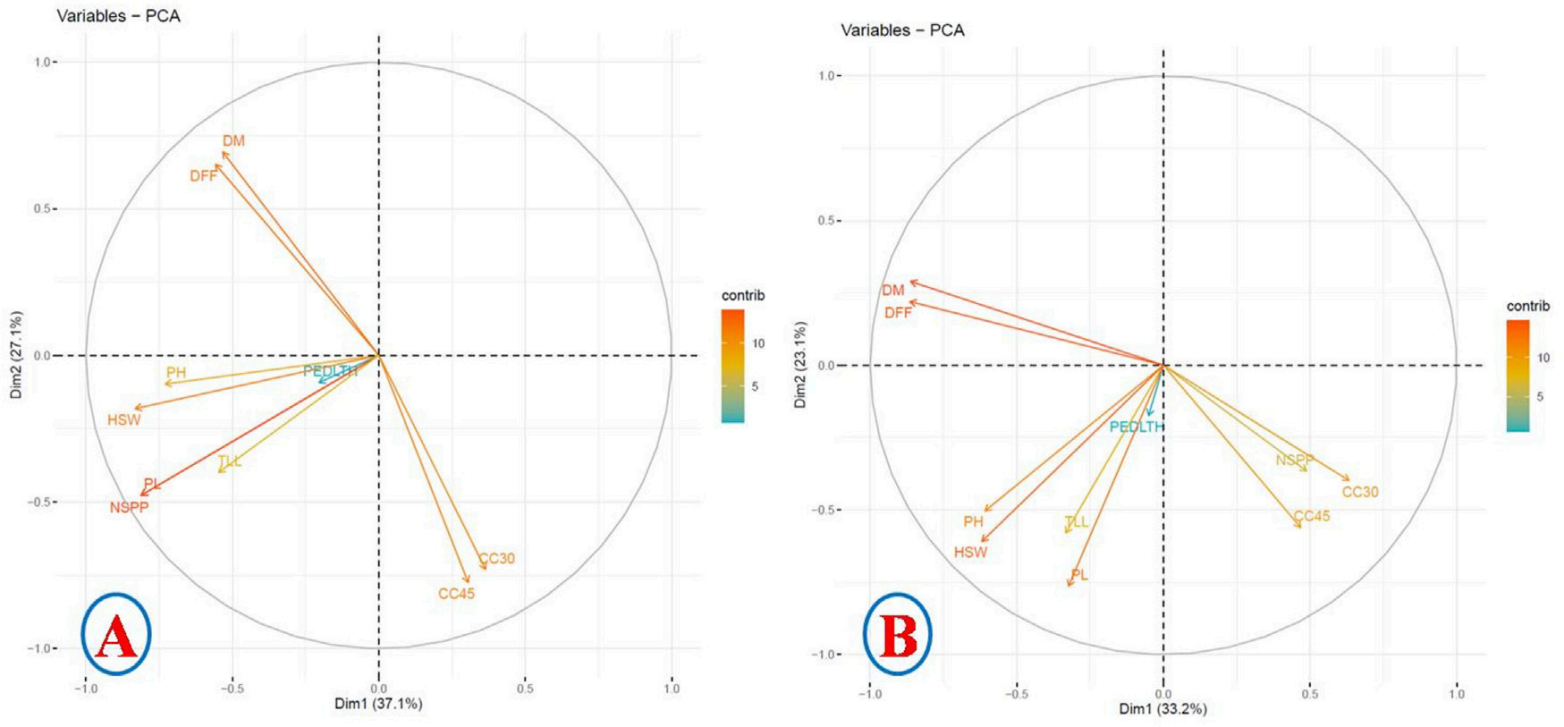
Principal component analysis with correlation. **(A)** 2017 data. **(B)** 2018 data.

The principal component analysis (PCA) established relationships amongst the studied traits during 2017 and 2018. The PCA of 2017 data revealed that dimension 1 (37%), dimension 2 (27%), dimension 3 (12.5%), and dimension 4 (10.12%) altogether contributed around 86.62% of the explained phenotypic variances of the studied traits (Figure 2). Dimension 1 had more than 10% contribution from HSW, NSPP, PH, and PL. Dimensions 2 and 4 had >10% contribution from DFF, DM, CC30, and CC45. Dimension 3 had >10% variation from PEDLTH and TLL. The PCA also revealed that DFF and DM had a negative correlation with CC30 and CC45. HSW showed a positive correlation with the pod length and number of pods per plant (Figure 2).

The PCA of 2018 also revealed that dimension 1 (33.2%), dimension 2 (23.13%), dimension 3 (12.99%), and dimension 4 (10.81%) altogether contributed around 80.13% of the explained phenotypic variances of the studied traits (Figure 2). The phenotypic variance of >10% in dimension 1 was contributed by PH, CC30, DM, DFF, and HSW. Dimension 2 had >10% phenotypic variance contribution from PH, PL, TLL, HSW, and CC45. Dimension 3 had >10% phenotypic variances contribution from PEDLTH, TLL, and CC45, and dimension 4 had >10% phenotypic variances contribution from NSPP, PEDLTH, TLL, and CC30. The PCA also revealed a negative correlation of DFF and DM with NSPP, CC30, and CC45 (Figure 2).

### 3.4 Population genetic structure analysis

**FIGURE 3:**
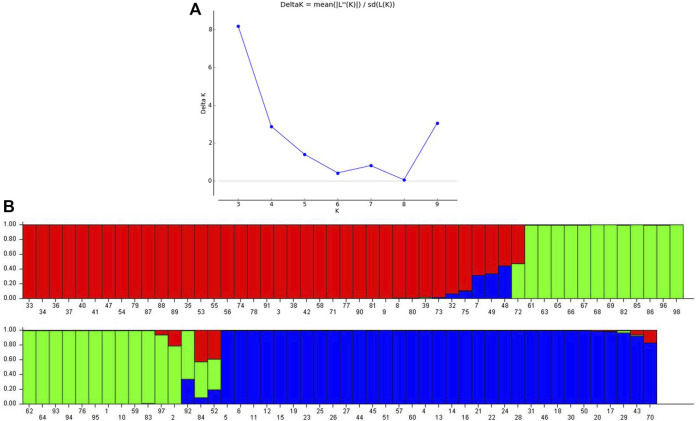
Population genetic structure analysis: **(A)** Evanno Delta K-based sub-population prediction. **(B)** Three distinct sub-populations in a chosen *Vigna* panel.

Population genetic structure analysis revealed three sub-populations (*K* = 3) in the selected panel of 13 cultivated mungbean varieties and 85 accessions belonging to 15 different *Vigna* species (Figure 3). The major sub-population 1 (Q1 in red color) represented 34 genotypes (35%) accommodating *V. trilobata* (17 acc.), *V. umbellata* (13 acc.), *V. vexillata* (2 acc.), and *V. dalzelliana* (2 acc.). Sub-population 2 (Q2 in green color) comprised 24 (24.5%) genotypes including cultivated *Vigna* (13 acc.); *V. trilobata* and *V. umbellata* (3 acc. each); *V. stipulaceae* (2 acc.); and 1 accession each of *V. unguiculata*, *V. glabrescence*, and *V. radiata*. Sub-population 3 (Q3 in blue color) consisted of 33 genotypes, and these belonged to *V. mungo* (9 acc.), *V. radiata* (11 acc.), *V. radiata* var*. sublobata* (6 acc.), *V. trinervia* var*. bourneae* (3 acc.), and *V. silvestris* and *V. radiata* var*. setulosa* (2 acc. each). A total of seven genotypes, namely, IC247408 (*V. dalzelliana*), IC277014 (*V. silvestris*), IC277021 (*V. silvestris*), JAP/10–51 (*V. trinervia*), TCR279, IC298665, and NSB007 (*V. unguiculata*), were considered as the admixture class since these shared genomic content from Q1, Q2, and Q3 sub-populations. The genotype IC247408 considered as the admixture class had a major genome frequency belonging to Q1 (0.684), whereas two other genotypes belonging to *V. dalzelliana* clustered with *V. trilobata* and *V. umbellata*. Similarly, TCR279 (0.65), IC298665 (0.42), and NSB007 (0.49) belonging to *V. unguiculata* had major genome frequency related to the Q2 sub-population, whereas Goa cowpea (*V. unguiculata*) grouped with the genotypes of cultivated mungbean varieties and *V. trilobata*, *V. umbellata*, *V. stipulaceae*, *V. glabrescence*, and *V. radiata.*

### 3.5 LD between studied markers

**FIGURE 4:**
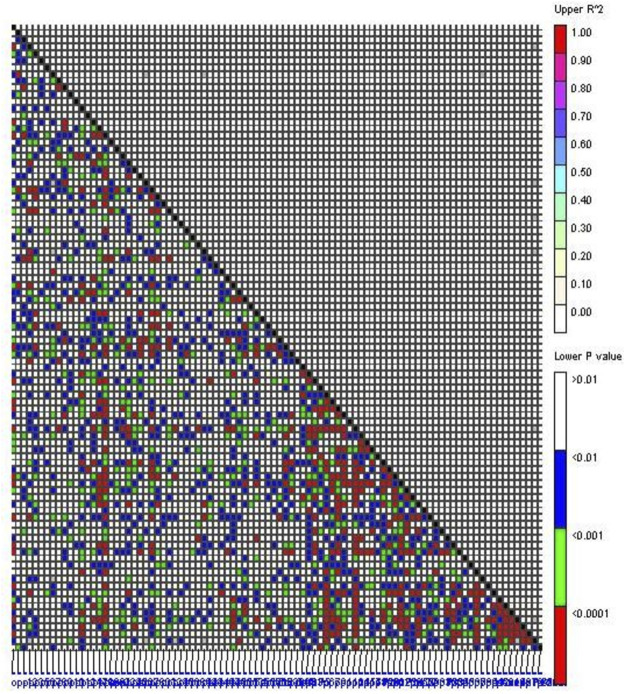
Linkage disequilibrium between marker pairs. The lower diagonal part represents *p*-values between the marker pairs; the upper diagonal part represents the *R* ^2^ value between marker pairs.

The *r* ^2^ value between alleles of two loci varied between 0.007 (CEDG071 vs. BMD-35) and 0.048 (CEDG256 vs. CEDG185) (Figure 4). Most of the marker pairs had *r* ^2^ values less than 0.02 (Supplementary Table S4). The observed *r* ^2^ values were near zero which indicated that the markers were in perfect equilibrium, and therefore, two markers will not provide identical information. Similarly, the D′value between two loci varied from 0.83 (CEDG150 vs. CEDG100) to 0.28 (CEDG060 vs. BMD-6). Most marker pairs have a D’value of more than 0.55 (3,250 pairs), thus indicating the existence of optimal recombination in the present population.

### 3.6 GLM (Q)-based association analysis

GLM with Q and phenotypic data of the experiments conducted in 2017 identified 34 MTAs for 9 out of 10 studied traits except for NSPP at a corrected *p*-value of <0.00001 (Supplementary Table S3). A maximum of 8 MTAs were identified for HSW followed by 6 for DFF; 5 for DM; 4 for PL; 3 each for CC30, PEDLTH, and PH; and 1 each for CC45 and TLL. The marker CEDG225 was observed to have an association with CC30, CC45, DFF, DM, and PH. Similarly, the markers, VR022, CEDG118, CP1225, and DMBSSR016, were also associated with HSW and PL. The markers CEDG096A, CEDG033, and JMES1424 were found associated with highly correlated traits such as DFF and DM.

GLM with Q and the phenotypic data of the experiments conducted in 2018 identified 30 MTAs for 8 out of the 10 studied traits, except NSPP and PL, at a corrected *p*-value < 0.00001 (Supplementary Table S3). A maximum of 9 MTAs for HSW and the lowest 1 MTA for CC45 were identified. A total of 6 MTAs each for DFF and DM and 2 each for CC30, PEDLTH, PH, and TLL were also identified. Of these MTAs, the loci CEDG225, CEDG096A, CEDG033, MBSSR008, and CEDG100 were observed to be associated with more than one trait. The marker CEDG225 was associated with CC30, DFF, DM, and TLL. Similarly, CEDG096A was associated with CC30, CC45, DFF, and DM. The markers CEDG033 and MBSSR008 were also associated with DFF and DM. Marker CEDG100 was associated with HSW and PH. A total of 24 MTAs associated with HSW (8), DFF (6), DM (5), PEDLTH (2), PH (1), TLL (1), and CC30 (1) were consistently expressed in both seasons.

### 3.7 Mixed linear model (Q+K)-based association analysis

**FIGURE 5:**
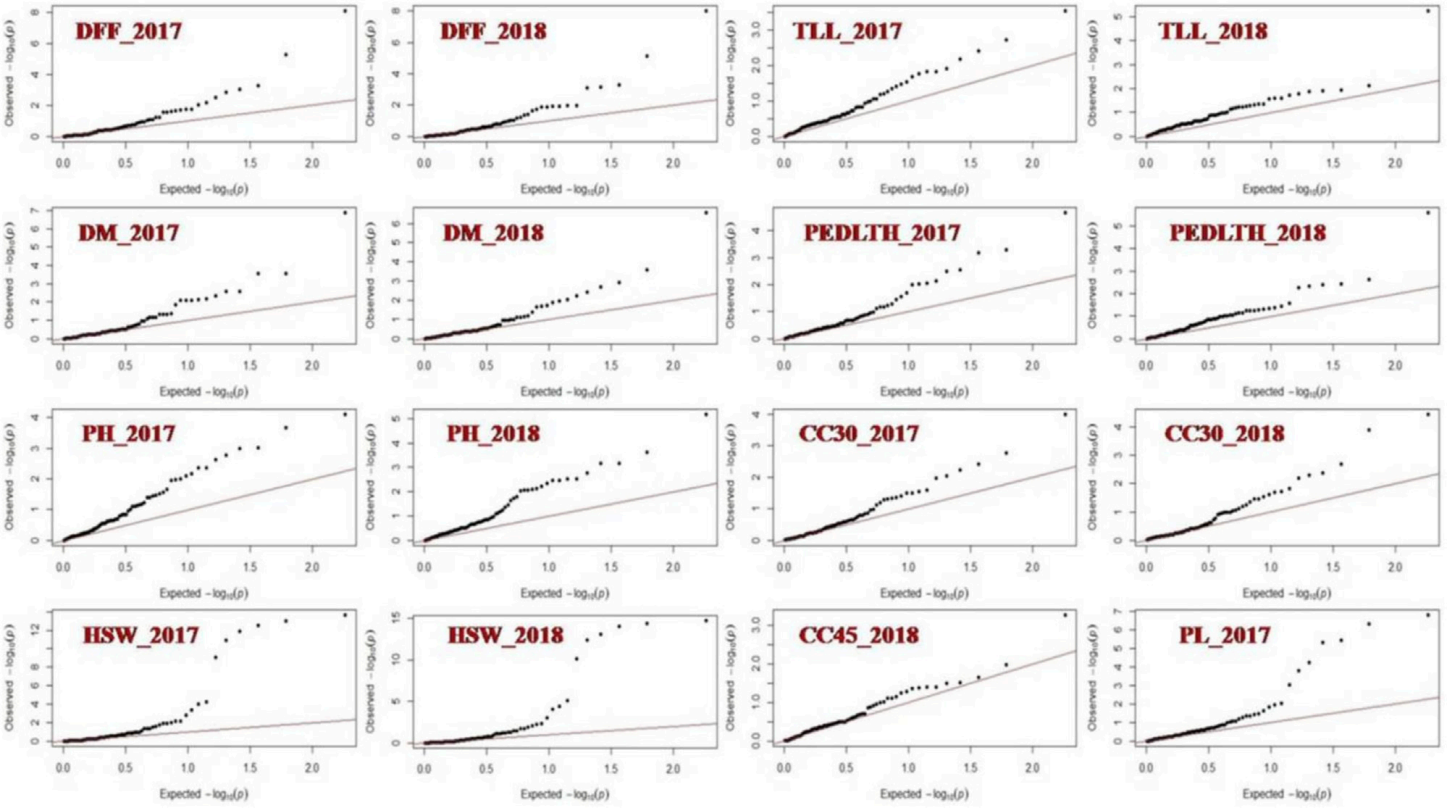
QQ bi-plot showing the association of markers with yield-related traits in *Vigna* species.

MLM analysis revealed an association of 13 markers, namely, VR018, VR039, VR022, CEDG033, GMES0337, MBSSR008, CEDG220, VM27, CP1225, CP08695, CEDG100, CEDG008, and CEDG096A, with 9 out of 10 studied traits except for NSPP from the data of both the years at value < 0.00054 with the Bonferroni correction (alpha 0.05; n:92) (Table 2; Figure 5). Of these 13 markers, seven markers, namely, VR018 (PH and TLL), VR022 (HSW and PL), CEDG033 (DFF and DM), MBSSR008 (DFF and DM), CP1225 (CC30, DFF, and DM), CEDG100 (PH and TLL), and CEDG096A (CC30 and CC45) were observed to be associated with multiple traits. The phenotypic variation explained by these MTAs varied from 1.52 (DFF-CP1225) to 34.87 (PH-CEDG100). The maximum number of 3 MTAs was identified for traits like CC30, DFF, DM, PH, TL, and HSW, and a minimum of 1 MTA was identified for PL (VR022) and CC45 (CEDG096A). The MTAs identified for DFF and DM were also similar. The association of markers CEDG033 and MBSSR008 linked with DFF and DM were consistent across the 2 years of experimentation, explaining up to 15.54% phenotypic variation. The markers CEDG100, CP08695, and VR018 were linked with PH expression, and among these, VR018 identified from 2 years of data explained 34.86% and 19.87% phenotypic variation in 2017 and 2018, respectively. Importantly, markers such as VR022, GMES0337, and CEDG008 have been consistently identified as linked with HSW expression in the 2 years’ data, and the phenotypic variation explained by them varied from 1.62 to 2.5%. The phenotypic variation of MTA linked with CC30 (CEDG096A, CP1225, and VR039) varied from 3.09 to 11.58. The marker CEDG096A linked with CC30 and CC45 was identified only in 2018 which explained 11.58% and 13% phenotypic variation for these traits, respectively. A total of 15 MTAs (CEDG096A and VR039 for CC30; CEDG096A for CC45; CEDG033 and MBSSR008 for DFF and DM; CEDG100 and CP08695 for PH; VR022 for PL; VR018 for TLL; CEDG220 for PEDLTH; and VR022, GMES0337, and CEDG008 for HSW) identified for 9 traits from both seasons following the MLM (Q + K) approach were also identified through the GLM approach.

**TABLE 2:** Significant marker–trait associations identified from the MLM (Q + K) approach in different environments.

Trait	Locus	Allele	2017 data			2018 data		
			F-value	*p*-value	*R* ^2^	F-value	*p*-value	*R* ^2^
DFF	CEDC033	150	4.34	5.54E-06	14.49	4.25	7.54E-06	15.14
	MBSSR008	200	6.52	8.34E-09	2.34	6.50	9.81E-09	3.13
	CP1225	205	-			2.92	5.35E-04	1.52
DM	CEDC033	150	3.2	2.93E-04	15.51	3.25	2.55E-04	15.54
	MBSSR008	220	5.60	1.35E-07	2.86	5.38	2.72E-07	2.80
	CP1225	205	3.08	2.91E-04	2.61	-		
PH	CP08695	260	3.86	7.83E-05	22.49	-		
	CEDG100	220	3.10	2.11E-04	34.87	4.00	6.69E-06	19.87
	VR018	220	-			3.07	2.44E-04	17.93
HSW	VR022	290	9.80	2.94E-13	2.48	11.28	9.62E-15	2.12
	GMES0337	195	4.06	9.08E-05	2.19	4.90	8.11E-06	2.52
	CEDG008	115	2.89	4.49E-04	1.63	3.32	8.11E-05	1.79
PL	VR022	290	5.18	1.52E-07	2.06	-		
TLL	CEDG100	185	3.02	2.91E-04	1.96	-		
	VR018	235	-			4.04	5.80E-06	1.96
PEDLTH	CEDG220	145	4.27	2.13E-05	7.27	4.96	2.52E-06	6.98
	VM27	140	2.85	4.96E-04	11.82	-		
CC30	VR039	140	3.78	9.97E-05	6.16	-		
	CP1225	190	-			3.65	3.62E-05	3.10
	CEDG096A	195	-			3.17	1.26E-04	11.59
CC45	CEDG096A	195	-			2.81	5.39E-04	13.06

*p* ≤ 0.00054; *R* ^2^ = phenotypic variance; PH: plant height in cm; CC30: chlorophyll content at 30 days; CC45: chlorophyll content at 45 days; DFF: days to first flowering; DM: days to first pod maturity; PH: plant height in cm; PEDLTH: peduncle length in cm; PL: pod length in cm; TLL: terminal leaflet length in cm; and HSW: 100-seed weight in g.

### 3.8 Genes co-localized with trait-linked simple sequence repeat markers

The primer pairs of five markers were exactly (100%) aligned on the *Vigna* reference genome (*V. radiata* var*. radiata* (taxid:3,916)) (Table 3) and also possessed the same SSR motif as reported earlier in related *Vigna* species. Interestingly, four markers, namely, CP00361, CEDG100, VR022, and VR039, linked with various traits in this study are co-localized in protein-coding genes of the mungbean genome such as uncharacterized protein (LOC106765295), histone–lysine N-methyltransferase *ATX5* (LOC106775878), *SHOOT GRAVITROPISM 5* (LOC106774869), and putative UPF0481 protein *At3g02645* (LOC106767008), respectively. Another primer pair of the marker CEDG220 associated with peduncle length is found to be intergenic between the genes coding for GTP-binding nuclear protein Ran-3 (LOC106768487) and cytochrome P450 734A1 (LOC106767283).

**TABLE 3:** Trait-linked SSRs flanking candidate genes in *V. radiata*.

S. No.	Marker name	Genes of *V. radiata* var*. radiata* nesting SSR markers	Start and end of forward primer	Start and end of reverse primer	Amplicon length in *V. radiata*	SSR motifs in *V. radiata var. radiata*
1	CP00361	Uncharacterized LOC106765295	322–345	106–129	240	(CT)5N(CT)5N (TC)8
2	CEDG100	Histone–lysine N-methyltransferase ATX5 (LOC106775878)	283–304	465–444	183	(CT)6N (TC)9
3	CEDG220	CHR7; intergenic between LOC106768487 (42,176,444.42,178,582) and LOC106767283 (42,196,013.42,199,609)	152–172	23–44	150	(CT)12
4	VR022	SHOOT GRAVITROPISM 5 (LOC106774869)	66–92	188–209	144	(TCTA)5N (TC)5
5	VR039	Putative UPF0481 protein *At3g02645* (LOC106767008)	1,117–1,138	1,226–1,246	130	(AGA)5

## 4 Discussion

The test panel of 98 highly diverse *Vigna* genotypes belonging to cultivated (13 acc.) and wild (85 acc.) species was genotyped with cross-species and cross-genera SSR primers from adzuki bean-, common bean-, and scarlet runner bean (Pratap et al., 2015; Kumari et al., 2021)-identified MTAs for 10 quantitative traits. The SSRs can be effectively utilized for linkage studies and gene/QTL/association/comparative mapping studies in crops such as *Vigna* sp. In recent years, efforts have been made to develop various SSR markers such as genic/EST- and genomic-SSRs in many *Vigna* species (Wang et al., 2004; Gwag et al., 2006; Somta et al., 2008; Chen et al., 2015). Successful transferability of SSRs from adzuki bean, common bean, and scarlet runner bean was reported earlier in mungbean (Wang et al., 2009; Dikshit et al., 2012; Gupta et al., 2013a; Pratap et al., 2015; Pratap et al., 2016; Pratap et al., 2020; Kumari et al., 2021). Earlier, cross-genera- and cross-species-specific SSRs have been very well utilized in linkage and association mapping for identifying genes/QTLs responsible for various traits in mungbean, blackgram, *V. marina*, and cowpea in addition to their use in studying genetic diversity (Isemura et al., 2012; Gupta et al., 2013b; Singh et al., 2020; Rohilla et al., 2021; Vadivel et al., 2021).

Agronomically important yield-attributing traits, namely, PH, NSP, HSW, DF, DM, and resistance to various biotic and abiotic stresses, in most of the crop plants are likely to be controlled by many genes/QTLs with additive gene actions (Bharadwaj et al., 2021). Therefore, effective selection could be practiced along with modern genomic tools even in the early generations of crops. A large amount of phenotypic variation and correlations among traits observed in the panel of cultivated and wild *Vigna* accessions in the current study are in agreement with the earlier reports (Pratap et al., 2017; Azam et al., 2018; Nair et al., 2019; Kumari et al., 2021). The variability at the genotypic and phenotypic levels in the present study could be attributed to a large number of accessions belonging to 19 *Vigna* species which represented different eco-geographical regions of India. The positive correlation of HSW and PL, DFF, and DM was observed, which will greatly favor the development of short-duration mungbean cultivars with higher yield potential. Simultaneously, a focus will be required on the number of pods in a bunch in order to maintain more yield per plant.

Assigning different individuals to defined populations is highly beneficial in population genetics studies (Pritchard et al., 2000), whereas making a population classification can provide an inference of individual ancestry that might not have been adequately defined beforehand. In this study, 92.8% of the studied genotypes were distinctly grouped into three sub-populations and the remaining 7.2% of the studied lines were grouped as the admixture class. Majority of the cultivated genotypes of mungbean and the genotypes belonging to the primary gene pool such as *V. mungo* (9 acc.), *V. radiata* (11 acc.), *V. radiata* var*. sublobata* (6 acc.), *V. trinervia* var. *bourneae* (3 acc.), *V. silvestris* (2 acc.), and *V. radiata* var. *setulosa* (2 acc.) were clustered in sub-populations 2 and 3, respectively. However, most of the genotypes (88%) in sub-population 1 belonged to the secondary gene pool, and these included *V. trilobata* (17 acc.) and *V. umbellata* (13 acc.). *V. trilobata* have a higher number of pods with small seeds and varying capacities of plant growth habit (Pandiyan et al., 2012). Rice bean (*V. umbellata*) is mainly grown in northern India and Southeast Asia, and is considered as a donor for resistance to bruchids, yellow mosaic virus, Cercospora leaf spot, and bacterial leaf spot (Bhanu et al., 2018; Aidbhavi et al., 2021). The accession IC 251342 of *V. umbellata* was reported as photo- and thermo-period tolerant (Pratap et al., 2014a). Therefore, *V. umbellata* can be effectively utilized as a donor in mungbean and urdbean improvement programs to impart biotic and abiotic stress tolerance with due consideration of addressing pre- and post-fertilization barriers (Kumar et al., 2007; Chen et al., 2017; Pratap et al., 2018; Pratap et al., 2021b). So far, limited studies have been undertaken on the analysis of the population genetic structure in mungbean and other *Vigna* species; in most of them, 3–4 sub-populations in various germplasm panels and 6 sub-populations in a panel consisting of released varieties, advanced breeding materials, and elite lines were identified (Noble et al., 2012; Breria et al., 2020; Sokolkova et al., 2020; Kumari et al., 2021). The grouping of genotypes in our study is also in conformity with the earlier studies. Furthermore, the genotypes belonging to specific gene pools were grouped together as per the gene-pool classification of mungbean (Fatokun et al., 1993). Nonetheless, since the taxonomy of *Vigna* crops to date is primarily based on morphological attributes, the chance of misclassification of few *Vigna* species could have been encountered (Tomooka et al., 2006). Therefore, correct molecular taxonomy of the wild relatives of *Vigna* accessions is of prime importance in order to decipher their relationship and diversity among the various accessions of *Vigna* species for their further effective usage in various *Vigna* improvement programs (Takahashi et al., 2018; Kumari et al., 2021).

Mapping gene(s) or genomic region(s) which regulate important traits helps us to effectively utilize modern breeding technologies to expedite the varietal development process. Few studies on mapping genes/QTLs following QTL and association mapping approaches were reported in mungbean which included salinity stress tolerance (Breria et al., 2020), MYMIV resistance (Chankaew et al., 2011; Singh et al., 2017; Singh et al., 2020), maturity and hypocotyl color (Sokolkova et al., 2020), seed mineral concentration (Wu et al., 2020), and domestication-related traits (Isemura et al., 2012). QTL mapping for HSW, NPP, NSP, and MYMIV tolerance was reported using a limited number (100) of segregating RILs and few (46) polymorphic SSR markers (Singh et al., 2013). Hence, mapping genes/QTLs following a diverse panel consisting of an extensive collection of different *Vigna* species will really benefit not only the mungbean development program but also other closely related *Vigna* species. Out of the identified MTAs from GLM and MLM approaches in this study, 11 MTAs were previously reported in various mapping populations of *Vigna* species following the QTL mapping approach for traits such as MYMIV (CEDG100) in mungbean (Kitsanachandee et al., 2013), MYMV (CEDG225) in blackgram (Gupta et al., 2013b; Vadivel et al., 2021) and mungbean (CEDG225, MBSSR008, and VM27) (Singh et al., 2020), Cercosporaleaf spot (CEDG008) (Tantasawat et al., 2020), HSW (VM27) in *V. marina* (Chankaew et al., 2014), and HSW (CP1225) in mungbean (Alam et al., 2014). In addition to these, QTLs related to domestication-related traits such as seed length and number of seeds per pod linked/flanking the SSR marker CEDG220; pod width with CEDG096A; total number of pods with CEDG096A; stem internode length with CEDG271 and CP00361; HSW with JMES1424; and percent of shattered pods with MBSSR008 in mungbean using BC_1_F_1_ biparental a mapping population derived from a cross between a wild mungbean accession (JP211874) and a cultivated mungbean landrace (JP229096 cv. Sukhothai) (Isemura et al., 2012) were also identified for different traits in our study. The marker CP1225 identified for HSW in this study is consistent with an earlier report (Alam et al., 2014) where it was identified for the same trait in the F_2_ mungbean mapping population derived from BM1 × BM6 by single regression analysis.

The marker CEDG100 was associated with HSW, PH, and TLL, and this MTA was consistently expressed for PH expression in both the seasons’ data explaining >30% of phenotypic variation, and hence, was considered as a major and consistent MTA for PH. This genic/EST SSR marker co-localized gene encoding histone–lysine N-methyltransferase ATX5 is implicated in epigenetics, specifically in the methylation of histones. Histone methylation is an important epigenetic modification which plays a crucial role in regulating the gene expression by either increasing or decreasing the target gene expression and genome stability (Kumar and Mohapatra, 2021). The histone–lysine methyltransferase (HKMTase), specifically ATX5, is involved in the methylation of lysine residue (H3K4 di- and trimethylation) present on the tail of the histone protein and *ARABIDOPSIS TRITHORAX 5* (*ATX5*) reported to function in abscisic acid and dehydration stress responses (Chen et al., 2017; Liu et al., 2018a) and glucose signaling in *Arabidopsis* (Liu et al., 2018b). Importantly, *ATX5* along with *ATX3 and ATX4* in *Arabidopsis* has a role in determining plant height (Chen et al., 2017). The triple mutant of *atx3-1*, *atx4-1*, and *atx5-1* exhibits dwarf and small rosette leaf phenotypes without altering the flowering time in *Arabidopsis* (Chen et al., 2017). Similarly, the VR22 marker associated with PL and HSW is co-located in gene-encoding *SHOOT GRAVITROPISM 5* (*SGR5*) (LOC106774869) in mungbean. Gravity affects many biological processes including negative (directing shoots upwards) and positive (downwards) gravitropism. *SGRs* have been shown to mediate the gravitropic responses of different plant organs. SRG5 is a C2H2-type ZF protein and functions primarily in the early steps of gravity perception in inflorescence stems (Morita et al., 2006). The mutant of *SGR5* exhibits an altered gravitropic response of the inflorescence stems by altering the patterns of starch accumulation or deposition in the endodermal cells of inflorescence stems (Tanimoto et al., 2008; Kim et al., 2016). Alternative splicing of *SGR5* into *SGR5a* and *SGR5b* modulates the gravitropic response of inflorescence stems at high temperatures in *Arabidopsis* (Kim et al., 2016). In addition to *SGR5*, other genes determining shoot gravitropism are *BIG GRAIN 1* (*BG1*) in rice (Liu et al., 2015); *LAZY1* (*LA1*) in rice, maize, and tea; and *Arabidopsis* (Overbeek, 1936; Jones and Adair, 1938; Li et al., 2007; Yoshihara et al., 2013), which determine spreading of tillers in rice, prostate culms in maize, and outward orientation branching and wider angles in *Arabidopsis* by primarily modulating polar auxin transport (PAT). Plant lateral organs such as primary and secondary branches are generated at a defined angle termed the gravitropic set-point angle and thus, determine the overall plant architecture which is mainly maintained by gravitropism (Digby and Firn, 1995). Hence, developing a variety with ideal plant architecture (IPA) in *Vigna* is necessary to enhance crop yield by harvesting light sources and converting sources efficiently to sink in plants. The important characteristics of IPA in *Vigna* include increased number of pods per plant as well as bunch, number of seeds per pod, high HSW, and short plant-type with erect growth habit.

Days to flowering and maturity are important in the mungbean improvement program to breed shorter-duration crop varieties with higher yields. The markers CEDC033 and MBSSR008 associated with the expression of DFF and DM have been observed to be consistent in both seasons. However, we could not identify these markers associated with HSW as identified in correlation studies where HSW had a positive correlation with DFF and DM traits. This could be ascribed to genes controlling HSW in *Vigna*. The MTAs identified in this study could be effectively utilized in improving other closely related *Vigna* species which lack genomic information such as trait-linked markers for deploying in marker-assisted crop improvement programs. The MTAs identified in this study for the traits DFF, DM, PH, and HSW can be further explored to utilize in the *Vigna* improvement program following marker-assisted breeding and cloning of the genes to help us understand the molecular mechanism controlling the expression of these traits in various *Vigna* species.

## Data Availability

The original contributions presented in the study are included in the article/Supplementary Materials, further inquiries can be directed to the corresponding author.
