# Supplementary material for: Association mapping for important agronomic traits in wild and cultivated Vigna species using cross-species and cross-genera simple sequence repeat markers
Source: Front Genet. 2022 Nov 3;13:1000440. doi: 10.3389/fgene.2022.1000440 (PMC9669911; doi:10.3389/fgene.2022.1000440)
Supplement: Supplementary file 1 [file Table1.DOCX]

**Supplementary Table 1.** Details of the Mungbean accessions used in the present study

| 1 | IPM-02-3 | Cultivated |
| --- | --- | --- |
| 2 | IPM-205-7 | Cultivated |
| 3 | IPM-2-14 | Cultivated |
| 4 | IPM-2K-14-9 | Cultivated |
| 5 | IPM-302-2 | Cultivated |
| 6 | IPM-410-3 | Cultivated |
| 7 | IPM-99-125 | Cultivated |
| 8 | IPU-07-3 | Cultivated |
| 9 | IPU-2-43 | Cultivated |
| 10 | MH-421 | Cultivated |
| 11 | Pant-U-39 | Cultivated |
| 12 | PDM-139 | Cultivated |
| 13 | VBG-04-008 | Cultivated |
| 14 | IC203864 | *V. dalzelliana* |
| 15 | IC247408 | *V. dalzelliana* |
| 16 | IC331615 | *V. dalzelliana* |
| 17 | IC251372 | *V. glabrescence* |
| 18 | IC251383 | *V. mungo* var*. mungo* |
| 19 | IC251385 | *V. mungo* var*. mungo* |
| 20 | IC251386 | *V. mungo* var*. mungo* |
| 21 | IC251387 | *V. mungo* var*. mungo* |
| 22 | IC251390 | *V. mungo* var*. mungo* |
| 23 | IC251393 | *V. mungo* var*. mungo* |
| 24 | IC251394 | *V. mungo* var*. mungo* |
| 25 | IC251396 | *V. mungo* var*. mungo* |
| 26 | IC251397 | *V. mungo* var*. mungo* |
| 27 | IC251432 | *V. radiata* |
| 28 | IC251433 | *V. radiata* |
| 29 | IC251434 | *V. radiata* |
| 30 | IC260725 | *V. radiata* |
| 31 | IC571775 | *V. radiata* |
| 32 | MungSeed-1 | *V. radiata* |
| 33 | IC251424 | *V. radiata* var*. radiata* |
| 34 | IC251425 | *V. radiata* var*. radiata* |
| 35 | IC251426A | *V. radiata* var*. radiata* |
| 36 | IC251426B | *V. radiata* var*. radiata* |
| 37 | IC251427 | *V. radiata* var*. radiata* |
| 38 | IC251431 | *V. radiata* var*. radiata* |
| 39 | IC251419 | *V. radiata* var*. setulosa* |
| 40 | IC251423 | *V. radiata* var*. setulosa* |
| 41 | IC253920 | *V. radiata.* var*. Sublobata* |
| 42 | IC247406 | *V. radiata.* var*. Sublobata* |
| 43 | IC251416 | *V. radiata.* var*. Sublobata* |
| 44 | IC253924 | *V. radiata.* var*. Sublobata* |
| 45 | IC256158 | *V. radiata.* var*. Sublobata* |
| 46 | IC349699 | *V. radiata.* var*. Sublobata* |
| 47 | IC277014 | *V. silvestris* |
| 48 | IC277021 | *V. silvestris* |
| 49 | IC277031 | *V. silvestris* |
| 50 | IC277036 | *V. silvestris* |
| 51 | Trichy local-1 | *V. stipulaceae* |
| 52 | Trichy local-2 | *V. stipulaceae* |
| 53 | Trichy local | *V. trilobata* |
| 54 | IC251435 | *V. trilobata* |
| 55 | IC251436 | *V. trilobata* |
| 56 | IC251438 | *V. trilobata* |
| 57 | IC276983 | *V. trilobata* |
| 58 | IC331436 | *V. trilobata* |
| 59 | IC331454 | *V. trilobata* |
| 60 | IC331456 | *V. trilobata* |
| 61 | IC349701 | *V. trilobata* |
| 62 | JAP/10-5 | *V. trilobata* |
| 63 | JAP/10-7A | *V. trilobata* |
| 64 | JAP/10-7B | *V. trilobata* |
| 65 | JAP/10-9 | *V. trilobata* |
| 66 | Kumur local | *V. trilobata* |
| 67 | LRM/13-24 | *V. trilobata* |
| 68 | LRM/13-30 | *V. trilobata* |
| 69 | LRM/13-32 | *V. trilobata* |
| 70 | LRM/13-34 | *V. trilobata* |
| 71 | LRM/13-43 | *V. trilobata* |
| 72 | *V.trilobata* | *V. trilobata* |
| 73 | JAP/10-51 | *V. trinervia* |
| 74 | IC210563 | *V. trinervia* var*. bourneae* |
| 75 | IC247407 | *V. trinervia* var*. bourneae* |
| 76 | JAP/10-47 | *V. trinervia* var*. bourneae* |
| 77 | IC197812 | *V. umbellata* |
| 78 | IC528878 | *V. umbellata* |
| 79 | V.umbellata | *V. umbellata* |
| 80 | IC251439 | *V. umbellata* |
| 81 | IC251440 | *V. umbellata* |
| 82 | IC251441 | *V. umbellata* |
| 83 | IC251442 | *V. umbellata* |
| 84 | IC251444 | *V. umbellata* |
| 85 | IC251445 | *V. umbellata* |
| 86 | IC251446 | *V. umbellata* |
| 87 | IC251447 | *V. umbellata* |
| 88 | PRR-2007-2 | *V. umbellata* |
| 89 | PRR-2008-2 | *V. umbellata* |
| 90 | RB-5-1 | *V. umbellata* |
| 91 | RBL-50 | *V. umbellata* |
| 92 | RBL-6 | *V. umbellata* |
| 93 | GoaCowpea3 | *V. unguiculata* |
| 94 | IC298665 | *V. unguiculata* |
| 95 | NSB007 | *V. unguiculata* |
| 96 | TCR279 | *V. unguiculata* |
| 97 | IC248326 | *V. vexillata* |
| 98 | IC248343 | *V. vexillata* |

**Supplementary Table 2** List of SSRs used in present investigation

| S No. | Name of marker | Forward sequence (5’-3’) | Reverse sequence (5’- 3’) | Source | TM (^o^C) |
| --- | --- | --- | --- | --- | --- |
| 1 | CEDG036 | CAGGTATTGTGCAGAGAGAC | TGCACCCAAAAGCTGTAAGC | Adzuki bean | 53 |
| 2 | CEDG291 | CCTCAAGTGGGGTTACC | GGTCAACCTCATTCTCCC | Adzuki bean | 53 |
| 3 | CEDG220 | GGTATTGAAGTCACATGGTCC | GGTTGTTATCTTTGTGCACTCC | Adzuki bean | 53 |
| 4 | CEDG256 | CCTTCACTATGTCCACATCC | GTTGTTGGTCGGTTCAGAG | Adzuki bean | 55 |
| 5 | CEDG136 | GTTCCAAGTCTCCAATCCGTAC | CACTTCACTAGAACTGGTTCAG | Adzuki bean | 54 |
| 6 | CEDG100 | CCCATCAAGTAACTACATAACA | ATGTGGGACTGGACAAATAAAA | Adzuki bean | 48 |
| 7 | CEDG096A | TTACGAAACTGTGGCCTTCAT | TGAACAAAGATGACTTCGGTG | Adzuki bean | 48 |
| 8 | CEDG050 | GGCAGAATCGTACAAGTG | GTCAGATTCTCGCTTGCATG | Adzuki bean | 45 |
| 9 | CP00361 | TAAATGCTAATACTCGCCGCCTTG | ACTGTGTCCGTTCGTCCTTTTCTC | Adzuki bean | 51 |
| 10 | BM146 | GAGATGAGTCCTTTCCCTACCC | TGCAGACACAATTTATGAAGGC | Adzuki bean | 50 |
| 11 | DMBSSR035 | TTCCACAAAGCTTGCTTCAGAT | TTGCATTCCAGCACATTTACA | Adzuki bean | 45 |
| 12 | CEDG084 | ATCAACTGAGGAGCATCATCGA | CAACATTTCAACCTTGGGACAG | Adzuki bean | 48 |
| 13 | CEDG185 | CACGAACCGGTTACAGAGCG | CATCGCATTCCCTTCGCTGC | Adzuki bean | 55 |
| 14 | CEDG033 | GTGAGGTAGCTATGTAGCAC | ACTGGACCGACAAGAGTAAG | Adzuki bean | 45 |
| 15 | DMBSSR199 | AGAAATTAAATCCCCGTCTGCT | AGAGACAGAAGCTCTGGATGTTTT | Adzuki bean | 46 |
| 16 | DMBSSR024 | TTTCTGCGAAGAATCTGAAGG | TTTTGAGTACCATGCTCTTCCT | Adzuki bean | 50 |
| 17 | GMES0337 | TCCCTTTCTGCTTTTGCCTA | GAAATCCACAGCATGCTTCA |  | 48 |
| 18 | CEDG060 | TGCATCGTCTCATGCGACCAG | ATTCTGGCAGCGGAGAAACC | Adzuki bean | 52 |
| 19 | CEDG008 | AGGCGAGGTTTCGTTTCAAG | GCCCATATTTTTACGCCCAC | Adzuki bean | 52 |
| 20 | CEDG115 | GGCTCATTGTACCACTGGATAT | ATGCCTCCTTTCAGGTGATTGT | Adzuki bean | 55 |
| 21 | DMBSSR001 | GCAATCGATGGAGATTTGAA | TTCATGGATTGTTTTCCTTCC | Adzuki bean | 48 |
| 22 | CEDG118 | AACCCAACCAACCCTTGTGGTAAG | GCTGGAATCATAATACCGCCTTGT | Adzuki bean | 52 |
| 23 | CEDG146 | GGTGATCGGATTTCAGAG | GGAGAAGAGAATAGAGACG | Adzuki bean | 48 |
| 24 | CEDG225 | GAGGAAGTGTTGCAGCACC | GTAGACTCTGCAGAGGGATG | Adzuki bean | 52 |
| 25 | JMES1424 | TCTTCGGTGTTGCAATCAAG | ACAACCTTCAAACTGGCTGG |  | 49 |
| 26 | MBSSR008 | ACCATTACCTCCACAATCTC | AATGGAGTTCTACGTGATGG | Adzuki bean | 49 |
| 27 | CEDG271 | GCACTAAAGTTAGACGTGGTTC | CACTCCCACTGCCAAACAAGG | Adzuki bean | 50 |
| 28 | VM37 | TGTCCGCGTTCTATAAATCAGC | CGAGGATGAAGTAACAGATGATC | Adzuki bean | 52 |
| 29 | CEDG073 | CCCCGAAATTCCCCTACAC | AACACCCGCCTCTTTCTCC | Adzuki bean | 50 |
| 30 | CEDG071 | GGTCCATTGAGACGGATCGAG | TCCCACCTCAGCGGAATCC | Adzuki bean | 55 |
| 31 | CP00226 | ACATTTGGAGGCTCAAGTTGGAAA | GGCCTATTACTACAGTCTGGAGGG | Adzuki bean | 50 |
| 32 | DMBSSR059 | TGCCAGATTTGAGAAGAAAGGT | CATGCATGTGGATAAGAATTCAG | Adzuki bean | 52 |
| 33 | VM27 | TCAACAACACCTAGGAGCCAA | ATCGTGACCTAGTGCCCACC | Adzuki bean | 45 |
| 34 | BM212 | AGGAAGGGATCCAAAGTCACTC | TGAACTTTCAGGTATTGATGAATGAAG | Adzuki bean | 48 |
| 35 | CP1225 | TCATCAGCTCATAAGCCACTGGTA | TGAGTATTTAACCTGCGACAGGCA | Adzuki bean | 52 |
| 36 | CEDG267 | GTGCTTGCATATGCAATGTCC | GGCATCTAAGAAATCCATGTCG | Adzuki bean | 55 |
| 37 | DMBSSR016 | GTGCGAAAATTCGAAATGGT | TCAACTCAAGCAATGCTAAGTCA | Adzuki bean | 50 |
| 38 | CEDG075 | GCGACCTCGAAAATGGTGGTTT | TCACCAACTCACTCGCTCACTG | Adzuki bean | 55 |
| 39 | BMD2 | AGCGACAGCAAGAGAACCTC | CAACAAACGGTGATTGACCA | Adzuki bean | 52 |
| 40 | CEDG150 | GAAGGGAATGAAAATGAAACCC | GTTCAATCCATTCAGTCTCC | Adzuki bean | 50 |
| 41 | BM149 | CGATGGATGGATGGTTGCAG | GGGCCGACAAGTTACATCAAATTC | Adzuki bean | 55 |
| 42 | CP08695 | ATTAGTGGCGGCTCACAACAGTTC | ACGGCCTGTTGTCGGATAGAATAA | Adzuki bean | 55 |
| 43 | J01263 | ATGCATGTTCCAACCACCTTCTC | GGAGTGGAACCCTTGCTCTCATC | Common bean | 50 |
| 44 | PV-at001 | GGGAGGGTAGGGAAGCAGTG | GCGAACCACGTTCATGAATGA | Common bean | 45 |
| 45 | PV-ag003 | TCACGTACGAGTTGAATCTCAGGAT | GGTGTCGGAGAGGTTAAGGTTG | Common bean | 45 |
| 46 | PV-ag005 | GCTCACGTACGAGTTGAATCTCAG | ATCTGAGAGCAGCGACATGGTAG | Common bean | 45 |
| 47 | Pv-ctt002 | TCGCCGGGAAAGTTGCCAGT | TAGAAGGAGCGAGGGCCATG | Common bean | 45 |
| 48 | SSR-IAC 127 | GAGGCTAGCCCAACTTA | AGCGCAAGACTTTACTACTC | Common bean | 45 |
| 49 | SSR-IAC 188 | CCTGCCTTTGCCACTCCTC | CTCCTTCTACCCAGCCAAACC | Common bean | 45 |
| 50 | SSR-IAC 195 | TGGACATCAAACAAACAAAAA | TGCATCGGCAGTTCATCA | Common bean | 45 |
| 51 | DQ469392 | TCCCGATTTATAGTTCTCATTT | AGGGACCTCCTTCATCTC | Common bean | 50 |
| 52 | DQ469393 | CATTGAGATTTGAGGTTTCGTT | AGGTATTTCCATCGTGCTTTTC | Common bean | 45 |
| 53 | PvM03 | CCGCCTTCTTCTTCTTCTTC | CGGCGAGTCATCTTTTCC | Common bean | 45 |
| 54 | PvM22 | ACTCTCACAATGGCGGAATC | GGCGTTTTCTCCCTCTTCTT | Common bean | 45 |
| 55 | BMD-5 | CGTGGACTTGAATGGTTTCAG | TCCTTACCCTGTTCTGCTTCTC | Scarlet runner bean | 50 |
| 56 | BMD-6 | CATCGAATGCCCAAGAGAATA | CTCACTGTCTTCCATCCAAGC | Scarlet runner bean | 45 |
| 57 | BMD-8 | TTCATCCTCTCTCCCGAACTT | CTTTTGTGGCTGAGACATGGT | Scarlet runner bean | 50-40 Touchdown |
| 58 | BMD-13 | TCATGCCTGAGAAAGGGTCT | CCCTGCATCAGAAGTCCAAT | Scarlet runner bean/ *Phaseolus vulgaris* L | 45 |
| 59 | BMD-18 | AAAGTTGGACGCACTGTGATT | TCGTGAGGTAGGAGTTTGGTG | Scarlet runner bean | 48.5 |
| 60 | BMD-23 | GGCTTGGTCCTCTCATTGAA | TGGAAATTACCACCATGCAA | Scarlet runner bean | 45 |
| 61 | BMD-26 | CTTGCCTTGTGCTTCCTTCT | TCCATTCCCAACCAAGTTTC | Scarlet runner bean | 55-40 Touchdown |
| 62 | BMD-29 | CTTCACCGATCTGACAGCAG | TTTCTCCACTGGAACACTCG | Scarlet runner bean | 50 |
| 63 | BMD-31 | TGAAGAGGATCGCAAGGTTC | AGCCGAAACACTGTCCTTGT | Scarlet runner bean | 55-40 Touchdown |
| 64 | BMD-48 | CCCCACCAACTCTTTCTTCC | CAGAATTGACTTGGCGAGAA | Scarlet runner bean/ *Phaseolus vulgaris* L | 45 |
| 65 | BMD-35 | TCTCTTCCTTACCCTGTTCTGC | GCGTGGACTTGAATGGTTTC | Scarlet runner bean | 45 |
| 66 | BMD-47 | ACCTGGTCCCTCAAACCAAT | CAATGGAGCACCAAAGATCA | Scarlet runner bean | 45 |
| 67 | BMD-50 | TGGTGAGAGAAGGACAATAGCA | GCCGCTTGTGACGTTTATTT | Scarlet runner bean | 45 |
| 68 | BMD-51 | CGCCAATTCTTCAACCCTAA | GTAGTTCGCCCGAGGACTG | Scarlet runner bean | 48 |
| 69 | BMD-12 | CATCAACAAGGACAGCCTCA | GCAGCTGGCGGGTAAAACAG | Scarlet runner bean | 45 |
| 70 | X21 | AAACATACCCCTGGCAGTTCC | TTCTGACCTAAGAAAGAGCCTGG | Adzuki bean | 48 |
| 71 | X 34 | CGGAAGAAGAACGCAGAGTG | GCATCAACAAGGACTTCTGC | Adzuki bean | 45 |
| 72 | X40 | GATTGGGAATCTGCTGTTG | GTGATCCACACACAGTAC | Adzuki bean | 48 |
| 73 | X49 | GGCAGAATCGTACAAGTG | GTCAGATTCTCGCTTGCATG | Adzuki bean | 45 |
| 74 | X62 | TGGGCTACCAACTTTTCCTC | TGAGCGACATCTTCAACACG | Adzuki bean | 45 |
| 75 | X65 | CAACATTTCAACCTTGGGACAG | ATCAACTGAGGAGCATCATCGA | Adzuki bean | 45 |
| 76 | X87 | GTCCTTGTTTTCCTCTCCATGG | CATCAGCTGTTCAACACCCTGTG | Adzuki bean | 45 |
| 77 | VR011 | TGCATCTTTATTGAGTTCCGTG | GTTTTGGGGTGAATGTTGGATA | Adzuki bean | 45 |
| 78 | VR013 | GCCCAGATTTGTTCATCCTAGA | ACTGTTTTGAGTGGGGAAAAGA | Adzuki bean | 45 |
| 79 | VR015 | AAGATCACACACAACCAACCC | AATTAGTTCCACAGGCCAGATT | Adzuki bean | 48 |
| 80 | VR016 | AGGAGAAATTGTTGTTGTTCGG | GTGTTGATTGTTAGGGAGGGAG | Adzuki bean | 45 |
| 81 | VR018 | ATACAAGGGCAGGTGTAGCATC | CAGAAAACTTCATCCCCAGCTA | Adzuki bean | 50 |
| 82 | VR022 | TCTCTTCTCTCTTCTCTCTTCTTCTTC | TTGTGTCTGAGGCTATGTTGGT | Adzuki bean | 46.5 |
| 83 | VR022 | GCGTGATCGAGGCAGACTAT | GTGGGTAGCTCGGTAATAGCAC | Adzuki bean | 45 |
| 84 | VR024 | GCTCTAAAACACGAAAGGGGT | TCATGGTGGAAGAAAAGCAA | Adzuki bean | 48 |
| 85 | VR025 | GCTGTGGTGTATTTACCTTGGG | ATCCTCCGGTCATTATCTTGTG | Adzuki bean | 45 |
| 86 | VR032 | GATGGCTCTGCATTGAAACC | GATCTTCCCAACTTTCCCTCTC | Adzuki bean | 50-40 Touchdown |
| 87 | VR033 | ACTGAAGAGAATGGGTTAGGGG | TCACATTTGTTGGGTTGAAGAG | Adzuki bean | 45 |
| 88 | VR035 | GCCCGATGTCCTAGCTTTTAG | CCTCAAAACAATCAGAACTCTCG | Adzuki bean | 45 |
| 89 | VR037 | TCTCAGCATCTGTGGTGGTAGT | AGAATCCAACAACTCCTGCTTC | Adzuki bean | 4 |
| 90 | VR039 | TGCTAAAGGTTTCCTCTCAACT | GAATGAAGTCACGCACACAA | Adzuki bean | 45 |
| 91 | VR048 | GGCAGGGAAGGAGGAAAA | CAGCCACAACAAGGCACA | Adzuki bean | 52 |
| 92 | DQ345305 | GAGGCCAATCCCATAACTTT | AGCACCACATCAGAGATTCC | Common bean | 45 |

Where, TM=annealing temperature

**Supplementary Table 3** Significant marker-trait association identified using GLM approach (Q) in different environments

| **Trait** | **Locus** | **F value** | **P value** | **R^2^** |
| --- | --- | --- | --- | --- |
| HSW | VR022 | 8.2835 | 4.88E-12 | 0.6038 |
| HSW | CEDG271 | 6.4942 | 2.08E-09 | 0.5288 |
| HSW | GMES0337 | 7.6688 | 5.28E-09 | 0.4395 |
| HSW | CEDG118 | 8.0598 | 1.95E-10 | 0.5106 |
| HSW | DMBSSR059 | 4.4586 | 7.43E-06 | 0.3818 |
| HSW | CP1225 | 4.7368 | 1.64E-07 | 0.54 |
| HSW | DMBSSR016 | 6.5001 | 1.39E-09 | 0.543 |
| HSW | CEDG100 | 7.4318 | 3.02E-11 | 0.6064 |
| CC30 | VR025 | 5.7211 | 1.63E-06 | 0.372 |
| CC30 | VR039 | 5.4806 | 2.96E-07 | 0.4498 |
| CC30 | CEDG225 | 6.2688 | 2.90E-09 | 0.5644 |
| CC45 | CEDG225 | 4.012 | 7.34E-06 | 0.3818 |
| DFF | BMD-48 | 5.9049 | 2.17E-06 | 0.3539 |
| DFF | CEDC033 | 5.6471 | 4.59E-08 | 0.5112 |
| DFF | CEDG225 | 4.9257 | 2.64E-07 | 0.5113 |
| DFF | JMES1424 | 5.9177 | 2.52E-07 | 0.4272 |
| DFF | MBSSR008 | 8.1217 | 4.81E-11 | 0.5762 |
| DFF | CEDG096A | 3.7772 | 6.13E-06 | 0.5321 |
| DM | CEDC033 | 4.8011 | 7.73E-07 | 0.4811 |
| DM | CEDG225 | 4.7908 | 4.26E-07 | 0.5128 |
| DM | JMES1424 | 5.3314 | 1.34E-06 | 0.4096 |
| DM | MBSSR008 | 6.834 | 1.83E-09 | 0.5463 |
| DM | CEDG096A | 4.2052 | 1.09E-06 | 0.5648 |
| PEDLTH | CEDG271 | 4.0804 | 2.46E-06 | 0.472 |
| PEDLTH | CEDG220 | 4.5934 | 4.81E-06 | 0.3691 |
| PEDLTH | CP00361 | 4.6651 | 3.82E-06 | 0.3723 |
| PH | CEDG225 | 4.0781 | 5.73E-06 | 0.4761 |
| PH | CP08695 | 5.8667 | 9.20E-08 | 0.4748 |
| PH | CEDG100 | 3.9959 | 4.10E-06 | 0.5152 |
| PL | VR022 | 5.3057 | 5.21E-08 | 0.5397 |
| PL | CEDG118 | 4.9496 | 1.54E-06 | 0.4247 |
| PL | CP1225 | 4.0528 | 2.32E-06 | 0.5325 |
| PL | DMBSSR016 | 3.9302 | 9.97E-06 | 0.4565 |
| TLL | VR018 | 4.8209 | 1.78E-07 | 0.5021 |
| P value (0.001/92 where alpha is 0.001 and N=92) | | | |  |

| **Supplementary Table 4: LD between the studied marker loci** | | | | |
| --- | --- | --- | --- | --- |
| **Locus Name 1** | **Locus Name 2** | **R^2** | **D Prime** | **pDiseq** |
| PV-at001 | J01263 | 0.01457538 | 0.60555441 | 0.01383126 |
| PV-ag003 | J01263 | 0.01414834 | 0.58560065 | 0.01686341 |
| PV-ag003 | PV-at001 | 0.01809208 | 0.52387537 | 0.001998 |
| PV-ag005 | J01263 | 0.01904665 | 0.66200609 | 0 |
| PV-ag005 | PV-at001 | 0.01365437 | 0.56502939 | 0.04329004 |
| PV-ag005 | PV-ag003 | 0.01625498 | 0.55465322 | 0.00599401 |
| Pv-ctt002 | J01263 | 0.01578005 | 0.71817064 | 0.000999 |
| Pv-ctt002 | PV-at001 | 0.01226359 | 0.60109763 | 0.00699301 |
| Pv-ctt002 | PV-ag003 | 0.01284834 | 0.62071563 | 0.041841 |
| Pv-ctt002 | PV-ag005 | 0.01470459 | 0.68991983 | 0.000999 |
| SSR-IAC 127 | J01263 | 0.0093752 | 0.54818538 | 0.76923077 |
| SSR-IAC 127 | PV-at001 | 0.01347745 | 0.55365321 | 0.003996 |
| SSR-IAC 127 | PV-ag003 | 0.01493641 | 0.54859254 | 0.00699301 |
| SSR-IAC 127 | PV-ag005 | 0.01201365 | 0.55047652 | 0.14084507 |
| SSR-IAC 127 | Pv-ctt002 | 0.01064923 | 0.62333522 | 0.41666667 |
| SSR-IAC 188 | J01263 | 0.01250371 | 0.69245012 | 0.01510574 |
| SSR-IAC 188 | PV-at001 | 0.01033855 | 0.60332638 | 0.4 |
| SSR-IAC 188 | PV-ag003 | 0.01241198 | 0.59305613 | 0.10989011 |
| SSR-IAC 188 | PV-ag005 | 0.01195307 | 0.62726339 | 0.03787879 |
| SSR-IAC 188 | Pv-ctt002 | 0.01024081 | 0.68576653 | 0.33333333 |
| SSR-IAC 188 | SSR-IAC 127 | 0.01080924 | 0.61099792 | 0.23255814 |
| SSR-IAC 195 | J01263 | 0.01600018 | 0.5760603 | 0 |
| SSR-IAC 195 | PV-at001 | 0.02318261 | 0.56160564 | 0 |
| SSR-IAC 195 | PV-ag003 | 0.00937222 | 0.38974764 | 0.14285714 |
| SSR-IAC 195 | PV-ag005 | 0.01642182 | 0.53595581 | 0.02604167 |
| SSR-IAC 195 | Pv-ctt002 | 0.01465154 | 0.57702312 | 0.10204082 |
| SSR-IAC 195 | SSR-IAC 127 | 0.00913077 | 0.42812136 | 0.55555556 |
| SSR-IAC 195 | SSR-IAC 188 | 0.0125017 | 0.5552454 | 0.03460208 |
| DQ469392 | J01263 | 0.01235055 | 0.69210702 | 0.15873016 |
| DQ469392 | PV-at001 | 0.01663198 | 0.62718273 | 0.000999 |
| DQ469392 | PV-ag003 | 0.01419912 | 0.58640825 | 0.004995 |
| DQ469392 | PV-ag005 | 0.01104274 | 0.59645747 | 0.04975124 |
| DQ469392 | Pv-ctt002 | 0.01407232 | 0.7204019 | 0 |
| DQ469392 | SSR-IAC 127 | 0.01453575 | 0.63973723 | 0.003996 |
| DQ469392 | SSR-IAC 188 | 0.01122353 | 0.67960954 | 0.35714286 |
| DQ469392 | SSR-IAC 195 | 0.01789749 | 0.61576609 | 0 |
| DQ469393 | J01263 | 0.0137932 | 0.61242458 | 0.01644737 |
| DQ469393 | PV-at001 | 0.01414584 | 0.52165052 | 0.09009009 |
| DQ469393 | PV-ag003 | 0.02495534 | 0.57388343 | 0 |
| DQ469393 | PV-ag005 | 0.01475878 | 0.57226536 | 0.01879699 |
| DQ469393 | Pv-ctt002 | 0.01286339 | 0.61991362 | 0.05154639 |
| DQ469393 | SSR-IAC 127 | 0.01552462 | 0.59710117 | 0.01221001 |
| DQ469393 | SSR-IAC 188 | 0.01169246 | 0.60799329 | 0.1754386 |
| DQ469393 | SSR-IAC 195 | 0.01109824 | 0.44796426 | 0.10989011 |
| DQ469393 | DQ469392 | 0.01993547 | 0.64931964 | 0 |
| PvM03 | J01263 | 0.01276076 | 0.74155833 | 0.003996 |
| PvM03 | PV-at001 | 0.01357239 | 0.66563165 | 0 |
| PvM03 | PV-ag003 | 0.01400004 | 0.65856759 | 0.03496503 |
| PvM03 | PV-ag005 | 0.01347827 | 0.70266446 | 0.01930502 |
| PvM03 | Pv-ctt002 | 0.01197858 | 0.74182421 | 0.06289308 |
| PvM03 | SSR-IAC 127 | 0.01493822 | 0.69437102 | 0.01367989 |
| PvM03 | SSR-IAC 188 | 0.0112354 | 0.7322398 | 0.47619048 |
| PvM03 | SSR-IAC 195 | 0.01298299 | 0.61349644 | 0.002997 |
| PvM03 | DQ469392 | 0.01234187 | 0.69701866 | 0.07246377 |
| PvM03 | DQ469393 | 0.0127452 | 0.66887443 | 0.02873563 |
| PvM22 | J01263 | 0.00940915 | 0.48795092 | 0.55555556 |
| PvM22 | PV-at001 | 0.01351838 | 0.46838099 | 0.19230769 |
| PvM22 | PV-ag003 | 0.0129727 | 0.43437825 | 0.10869565 |
| PvM22 | PV-ag005 | 0.01468141 | 0.54963134 | 0.08196721 |
| PvM22 | Pv-ctt002 | 0.01256669 | 0.57467848 | 0.43478261 |
| PvM22 | SSR-IAC 127 | 0.01304282 | 0.5124849 | 0.0621118 |
| PvM22 | SSR-IAC 188 | 0.01075294 | 0.56494054 | 0.43478261 |
| PvM22 | SSR-IAC 195 | 0.01501121 | 0.40895171 | 0.00899101 |
| PvM22 | DQ469392 | 0.01396811 | 0.56260518 | 0.07246377 |
| PvM22 | DQ469393 | 0.01239072 | 0.46507696 | 0.25 |
| PvM22 | PvM03 | 0.01140451 | 0.59784873 | 0.03508772 |
| BMD-5 | J01263 | 0.01547287 | 0.59312604 | 0 |
| BMD-5 | PV-at001 | 0.01558042 | 0.52295123 | 0.03508772 |
| BMD-5 | PV-ag003 | 0.01831215 | 0.53822231 | 0.001998 |
| BMD-5 | PV-ag005 | 0.01870117 | 0.55872307 | 0.004995 |
| BMD-5 | Pv-ctt002 | 0.01531833 | 0.64562835 | 0.001998 |
| BMD-5 | SSR-IAC 127 | 0.01337338 | 0.52631097 | 0.05780347 |
| BMD-5 | SSR-IAC 188 | 0.01279664 | 0.5950459 | 0.00899101 |
| BMD-5 | SSR-IAC 195 | 0.00985533 | 0.40423454 | 0.47619048 |
| BMD-5 | DQ469392 | 0.01411639 | 0.56861441 | 0.07575758 |
| BMD-5 | DQ469393 | 0.01398999 | 0.51895527 | 0.02375297 |
| BMD-5 | PvM03 | 0.01418714 | 0.64029969 | 0.02793296 |
| BMD-5 | PvM22 | 0.01323906 | 0.46030761 | 0.00799201 |
| BMD-6 | J01263 | 0.0187691 | 0.49319131 | 0.02331002 |
| BMD-6 | PV-at001 | 0.01490836 | 0.40632695 | 0.14492754 |
| BMD-6 | PV-ag003 | 0.02679018 | 0.45279119 | 0 |
| BMD-6 | PV-ag005 | 0.01942338 | 0.46907279 | 0.09708738 |
| BMD-6 | Pv-ctt002 | 0.01721756 | 0.53768783 | 0.01172333 |
| BMD-6 | SSR-IAC 127 | 0.01249629 | 0.40459277 | 0.04329004 |
| BMD-6 | SSR-IAC 188 | 0.00934223 | 0.45384864 | 0.47619048 |
| BMD-6 | SSR-IAC 195 | 0.01239823 | 0.33041387 | 0.09090909 |
| BMD-6 | DQ469392 | 0.01285437 | 0.47201701 | 0.02762431 |
| BMD-6 | DQ469393 | 0.0205508 | 0.42363328 | 0.00899101 |
| BMD-6 | PvM03 | 0.01126958 | 0.51117659 | 0.29411765 |
| BMD-6 | PvM22 | 0.00897391 | 0.33177851 | 0.20833333 |
| BMD-6 | BMD-5 | 0.02019168 | 0.40036113 | 0.11111111 |
| BMD-8 | J01263 | 0.01755766 | 0.62038031 | 0 |
| BMD-8 | PV-at001 | 0.01145976 | 0.46579676 | 0.22727273 |
| BMD-8 | PV-ag003 | 0.01731138 | 0.50357856 | 0.06410256 |
| BMD-8 | PV-ag005 | 0.01650779 | 0.56641069 | 0.000999 |
| BMD-8 | Pv-ctt002 | 0.01247258 | 0.57311921 | 0.16666667 |
| BMD-8 | SSR-IAC 127 | 0.01459958 | 0.54433235 | 0.001998 |
| BMD-8 | SSR-IAC 188 | 0.01194103 | 0.5749741 | 0.13888889 |
| BMD-8 | SSR-IAC 195 | 0.01767351 | 0.47703063 | 0.000999 |
| BMD-8 | DQ469392 | 0.0144293 | 0.57909901 | 0.09615385 |
| BMD-8 | DQ469393 | 0.02799923 | 0.62355223 | 0 |
| BMD-8 | PvM03 | 0.01262939 | 0.62297679 | 0.11764706 |
| BMD-8 | PvM22 | 0.01183931 | 0.43881214 | 0.35714286 |
| BMD-8 | BMD-5 | 0.01217926 | 0.47727191 | 0.09708738 |
| BMD-8 | BMD-6 | 0.0186223 | 0.40314019 | 0.06666667 |
| BMD-13 | J01263 | 0.0141774 | 0.74138828 | 0.000999 |
| BMD-13 | PV-at001 | 0.01619859 | 0.67869404 | 0 |
| BMD-13 | PV-ag003 | 0.01660123 | 0.68951076 | 0 |
| BMD-13 | PV-ag005 | 0.01675775 | 0.72066139 | 0.004995 |
| BMD-13 | Pv-ctt002 | 0.01207554 | 0.73910497 | 0.12658228 |
| BMD-13 | SSR-IAC 127 | 0.0127368 | 0.65006872 | 0.01788909 |
| BMD-13 | SSR-IAC 188 | 0.01148794 | 0.72998225 | 0.16129032 |
| BMD-13 | SSR-IAC 195 | 0.01516147 | 0.62607302 | 0.02427184 |
| BMD-13 | DQ469392 | 0.01378823 | 0.7311981 | 0.04901961 |
| BMD-13 | DQ469393 | 0.01664728 | 0.69279281 | 0 |
| BMD-13 | PvM03 | 0.0129106 | 0.77603671 | 0.04385965 |
| BMD-13 | PvM22 | 0.0132805 | 0.61783141 | 0.003996 |
| BMD-13 | BMD-5 | 0.01763075 | 0.68973859 | 0 |
| BMD-13 | BMD-6 | 0.01394111 | 0.52482592 | 0.16666667 |
| BMD-13 | BMD-8 | 0.01530909 | 0.6521454 | 0.001998 |
| BMD-18 | J01263 | 0.0189568 | 0.70545866 | 0 |
| BMD-18 | PV-at001 | 0.01461929 | 0.5900335 | 0.0210084 |
| BMD-18 | PV-ag003 | 0.02065378 | 0.64327157 | 0 |
| BMD-18 | PV-ag005 | 0.0146677 | 0.60906828 | 0.00799201 |
| BMD-18 | Pv-ctt002 | 0.01467562 | 0.70828437 | 0.001998 |
| BMD-18 | SSR-IAC 127 | 0.01386831 | 0.59720768 | 0.02079002 |
| BMD-18 | SSR-IAC 188 | 0.01516102 | 0.70682155 | 0 |
| BMD-18 | SSR-IAC 195 | 0.01294935 | 0.49665473 | 0.06896552 |
| BMD-18 | DQ469392 | 0.01693398 | 0.68788764 | 0.002997 |
| BMD-18 | DQ469393 | 0.01420079 | 0.56977828 | 0.01008065 |
| BMD-18 | PvM03 | 0.01246993 | 0.69727527 | 0.00799201 |
| BMD-18 | PvM22 | 0.00996629 | 0.4848381 | 0.66666667 |
| BMD-18 | BMD-5 | 0.01813482 | 0.61583916 | 0.001998 |
| BMD-18 | BMD-6 | 0.01798027 | 0.47790688 | 0.06134969 |
| BMD-18 | BMD-8 | 0.02214817 | 0.62120655 | 0 |
| BMD-18 | BMD-13 | 0.0160089 | 0.74697945 | 0 |
| BMD-23 | J01263 | 0.01015178 | 0.54931422 | 0.66666667 |
| BMD-23 | PV-at001 | 0.01870177 | 0.53771567 | 0.00699301 |
| BMD-23 | PV-ag003 | 0.01575166 | 0.49620183 | 0.05780347 |
| BMD-23 | PV-ag005 | 0.01616192 | 0.59354849 | 0.000999 |
| BMD-23 | Pv-ctt002 | 0.01588458 | 0.65492555 | 0 |
| BMD-23 | SSR-IAC 127 | 0.01082329 | 0.49558545 | 0.3125 |
| BMD-23 | SSR-IAC 188 | 0.01167572 | 0.60369193 | 0.06493506 |
| BMD-23 | SSR-IAC 195 | 0.02187051 | 0.5431684 | 0.002997 |
| BMD-23 | DQ469392 | 0.01481711 | 0.60246523 | 0.00799201 |
| BMD-23 | DQ469393 | 0.01085213 | 0.48736572 | 0.16666667 |
| BMD-23 | PvM03 | 0.01133186 | 0.62032191 | 0.09259259 |
| BMD-23 | PvM22 | 0.00834338 | 0.40712316 | 0.5 |
| BMD-23 | BMD-5 | 0.01154035 | 0.48335954 | 0.52631579 |
| BMD-23 | BMD-6 | 0.02109312 | 0.44173436 | 0.0116144 |
| BMD-23 | BMD-8 | 0.0164567 | 0.53463472 | 0.05154639 |
| BMD-23 | BMD-13 | 0.01348115 | 0.64452813 | 0.01540832 |
| BMD-23 | BMD-18 | 0.01479021 | 0.57040233 | 0.07462687 |
| BMD-26 | J01263 | 0.01306751 | 0.67020931 | 0.01364256 |
| BMD-26 | PV-at001 | 0.01342675 | 0.59511189 | 0.0591716 |
| BMD-26 | PV-ag003 | 0.01543748 | 0.61622618 | 0.01992032 |
| BMD-26 | PV-ag005 | 0.01532602 | 0.65600657 | 0.07092199 |
| BMD-26 | Pv-ctt002 | 0.01246357 | 0.68945799 | 0.04504505 |
| BMD-26 | SSR-IAC 127 | 0.01686052 | 0.64745981 | 0.000999 |
| BMD-26 | SSR-IAC 188 | 0.01259939 | 0.6926782 | 0.0990099 |
| BMD-26 | SSR-IAC 195 | 0.01290323 | 0.56440347 | 0.01497006 |
| BMD-26 | DQ469392 | 0.01832248 | 0.71487512 | 0 |
| BMD-26 | DQ469393 | 0.01193028 | 0.59510584 | 0.26315789 |
| BMD-26 | PvM03 | 0.01391905 | 0.73347272 | 0.004995 |
| BMD-26 | PvM22 | 0.01053497 | 0.5546538 | 0.13157895 |
| BMD-26 | BMD-5 | 0.01483901 | 0.59798244 | 0.10752688 |
| BMD-26 | BMD-6 | 0.00972054 | 0.43379134 | 0.15625 |
| BMD-26 | BMD-8 | 0.01362653 | 0.58659083 | 0.001998 |
| BMD-26 | BMD-13 | 0.01313846 | 0.68773351 | 0.04484305 |
| BMD-26 | BMD-18 | 0.01494637 | 0.65385599 | 0.00699301 |
| BMD-26 | BMD-23 | 0.00818282 | 0.5073032 | 0.66666667 |
| BMD-29 | J01263 | 0.0170418 | 0.64240453 | 0 |
| BMD-29 | PV-at001 | 0.01380304 | 0.52571184 | 0.28571429 |
| BMD-29 | PV-ag003 | 0.01408312 | 0.51349246 | 0.18867925 |
| BMD-29 | PV-ag005 | 0.0220013 | 0.6262771 | 0 |
| BMD-29 | Pv-ctt002 | 0.01540755 | 0.68624132 | 0 |
| BMD-29 | SSR-IAC 127 | 0.01162778 | 0.51911411 | 0.83333333 |
| BMD-29 | SSR-IAC 188 | 0.01168066 | 0.63401023 | 0.25 |
| BMD-29 | SSR-IAC 195 | 0.01553278 | 0.50723042 | 0.0877193 |
| BMD-29 | DQ469392 | 0.02093756 | 0.66434719 | 0 |
| BMD-29 | DQ469393 | 0.01275123 | 0.52055445 | 0.19607843 |
| BMD-29 | PvM03 | 0.01657627 | 0.72903875 | 0 |
| BMD-29 | PvM22 | 0.02399129 | 0.58212233 | 0.000999 |
| BMD-29 | BMD-5 | 0.03437179 | 0.59339193 | 0 |
| BMD-29 | BMD-6 | 0.01636489 | 0.42976342 | 0.004995 |
| BMD-29 | BMD-8 | 0.01306706 | 0.51638899 | 0.05076142 |
| BMD-29 | BMD-13 | 0.01849093 | 0.73228988 | 0 |
| BMD-29 | BMD-18 | 0.01455211 | 0.60520032 | 0 |
| BMD-29 | BMD-23 | 0.01054918 | 0.48153598 | 0.625 |
| BMD-29 | BMD-26 | 0.01353803 | 0.63115911 | 0.03344482 |
| BMD-31 | J01263 | 0.01456071 | 0.58874302 | 0.001998 |
| BMD-31 | PV-at001 | 0.01298935 | 0.50054422 | 0.03968254 |
| BMD-31 | PV-ag003 | 0.01094288 | 0.45805827 | 0.32258065 |
| BMD-31 | PV-ag005 | 0.02228547 | 0.64521327 | 0 |
| BMD-31 | Pv-ctt002 | 0.01358514 | 0.63000108 | 0.04132231 |
| BMD-31 | SSR-IAC 127 | 0.01169991 | 0.51347674 | 0.05586592 |
| BMD-31 | SSR-IAC 188 | 0.01170639 | 0.61020447 | 0.12345679 |
| BMD-31 | SSR-IAC 195 | 0.01371087 | 0.45725829 | 0.02132196 |
| BMD-31 | DQ469392 | 0.01868801 | 0.64649492 | 0 |
| BMD-31 | DQ469393 | 0.0212505 | 0.57495015 | 0.000999 |
| BMD-31 | PvM03 | 0.0097764 | 0.59010837 | 0.38461538 |
| BMD-31 | PvM22 | 0.0141859 | 0.47295103 | 0.11235955 |
| BMD-31 | BMD-5 | 0.01662511 | 0.51143745 | 0.04587156 |
| BMD-31 | BMD-6 | 0.01596949 | 0.40912609 | 0.11627907 |
| BMD-31 | BMD-8 | 0.01721435 | 0.55708721 | 0 |
| BMD-31 | BMD-13 | 0.01438599 | 0.64434976 | 0.001998 |
| BMD-31 | BMD-18 | 0.01633823 | 0.58909562 | 0 |
| BMD-31 | BMD-23 | 0.02002886 | 0.55348213 | 0.01253133 |
| BMD-31 | BMD-26 | 0.01247072 | 0.58432599 | 0.02150538 |
| BMD-31 | BMD-29 | 0.01755161 | 0.56634348 | 0.001998 |
| BMD-48 | J01263 | 0.01124578 | 0.50431774 | 0.08264463 |
| BMD-48 | PV-at001 | 0.00947718 | 0.42004234 | 0.17241379 |
| BMD-48 | PV-ag003 | 0.01518782 | 0.4626401 | 0.17241379 |
| BMD-48 | PV-ag005 | 0.01916859 | 0.55397863 | 0.00799201 |
| BMD-48 | Pv-ctt002 | 0.01057196 | 0.5494553 | 0.83333333 |
| BMD-48 | SSR-IAC 127 | 0.01415802 | 0.52501968 | 0.00899101 |
| BMD-48 | SSR-IAC 188 | 0.01232052 | 0.53997796 | 0.05952381 |
| BMD-48 | SSR-IAC 195 | 0.01621569 | 0.44719621 | 0.11627907 |
| BMD-48 | DQ469392 | 0.01292147 | 0.54332629 | 0.0952381 |
| BMD-48 | DQ469393 | 0.01644944 | 0.53498178 | 0.00699301 |
| BMD-48 | PvM03 | 0.0161781 | 0.61857059 | 0 |
| BMD-48 | PvM22 | 0.0104414 | 0.4190657 | 0.29411765 |
| BMD-48 | BMD-5 | 0.01232293 | 0.4154171 | 0.71428571 |
| BMD-48 | BMD-6 | 0.01941094 | 0.3853572 | 0.02155172 |
| BMD-48 | BMD-8 | 0.01973159 | 0.51843855 | 0.002997 |
| BMD-48 | BMD-13 | 0.01555534 | 0.63725614 | 0.003996 |
| BMD-48 | BMD-18 | 0.02255753 | 0.63318086 | 0 |
| BMD-48 | BMD-23 | 0.01665931 | 0.50044233 | 0.01464129 |
| BMD-48 | BMD-26 | 0.01068081 | 0.5047832 | 0.58823529 |
| BMD-48 | BMD-29 | 0.01236571 | 0.46557443 | 0.38461538 |
| BMD-48 | BMD-31 | 0.01869717 | 0.46946798 | 0.00699301 |
| BMD-35 | J01263 | 0.01385755 | 0.54983825 | 0.03236246 |
| BMD-35 | PV-at001 | 0.01422458 | 0.47783316 | 0.00599401 |
| BMD-35 | PV-ag003 | 0.01821233 | 0.46929915 | 0.00899101 |
| BMD-35 | PV-ag005 | 0.01279001 | 0.46232417 | 0.22727273 |
| BMD-35 | Pv-ctt002 | 0.0131773 | 0.57671271 | 0.0456621 |
| BMD-35 | SSR-IAC 127 | 0.00994689 | 0.43900645 | 0.1369863 |
| BMD-35 | SSR-IAC 188 | 0.01153329 | 0.56217761 | 0.16949153 |
| BMD-35 | SSR-IAC 195 | 0.0214588 | 0.48063828 | 0.01324503 |
| BMD-35 | DQ469392 | 0.01236299 | 0.54055024 | 0.22222222 |
| BMD-35 | DQ469393 | 0.01825932 | 0.52610697 | 0.001998 |
| BMD-35 | PvM03 | 0.01053665 | 0.58853083 | 0.18867925 |
| BMD-35 | PvM22 | 0.01421888 | 0.44899797 | 0.15625 |
| BMD-35 | BMD-5 | 0.01999745 | 0.53361949 | 0 |
| BMD-35 | BMD-6 | 0.00923087 | 0.3040587 | 0.66666667 |
| BMD-35 | BMD-8 | 0.01755415 | 0.48068557 | 0.000999 |
| BMD-35 | BMD-13 | 0.01338057 | 0.60364396 | 0.03184713 |
| BMD-35 | BMD-18 | 0.01842804 | 0.59608028 | 0 |
| BMD-35 | BMD-23 | 0.01712714 | 0.48538539 | 0.09345794 |
| BMD-35 | BMD-26 | 0.0140625 | 0.57924208 | 0.03984064 |
| BMD-35 | BMD-29 | 0.01886246 | 0.52417863 | 0.02785515 |
| BMD-35 | BMD-31 | 0.02339535 | 0.55043381 | 0 |
| BMD-35 | BMD-48 | 0.01115473 | 0.37696577 | 0.12658228 |
| BMD-47 | J01263 | 0.01585429 | 0.66271073 | 0 |
| BMD-47 | PV-at001 | 0.01202505 | 0.55830145 | 0.43478261 |
| BMD-47 | PV-ag003 | 0.01023067 | 0.5022541 | 0.37037037 |
| BMD-47 | PV-ag005 | 0.01086786 | 0.56922207 | 0.03676471 |
| BMD-47 | Pv-ctt002 | 0.01144976 | 0.65145017 | 0.14925373 |
| BMD-47 | SSR-IAC 127 | 0.01326687 | 0.60136034 | 0.02079002 |
| BMD-47 | SSR-IAC 188 | 0.01444688 | 0.68728752 | 0.004995 |
| BMD-47 | DQ469392 | 0.0157729 | 0.64991463 | 0.001998 |
| BMD-47 | DQ469393 | 0.01812236 | 0.6114054 | 0 |
| BMD-47 | PvM03 | 0.01119768 | 0.68415771 | 0.58823529 |
| BMD-47 | PvM22 | 0.01372012 | 0.5347865 | 0.06711409 |
| BMD-47 | BMD-5 | 0.01056495 | 0.51848783 | 0.20408163 |
| BMD-47 | BMD-6 | 0.01655235 | 0.49106121 | 0.01101322 |
| BMD-47 | BMD-8 | 0.01110487 | 0.51278929 | 0.58823529 |
| BMD-47 | BMD-13 | 0.01202008 | 0.68876656 | 0.03508772 |
| BMD-47 | BMD-18 | 0.01195662 | 0.60301178 | 0.22222222 |
| BMD-47 | BMD-23 | 0.01570067 | 0.58847139 | 0 |
| BMD-47 | BMD-26 | 0.01252208 | 0.6364871 | 0.55555556 |
| BMD-47 | BMD-29 | 0.0149893 | 0.62696734 | 0.01658375 |
| BMD-47 | BMD-31 | 0.01641562 | 0.58927762 | 0.23809524 |
| BMD-47 | BMD-48 | 0.01011019 | 0.47001918 | 0.26315789 |
| BMD-47 | BMD-35 | 0.0117791 | 0.49406716 | 0.23255814 |
| BMD-50 | J01263 | 0.01822845 | 0.5796625 | 0.000999 |
| BMD-50 | PV-at001 | 0.02189511 | 0.5507842 | 0 |
| BMD-50 | PV-ag003 | 0.01372012 | 0.43254371 | 0.03058104 |
| BMD-50 | PV-ag005 | 0.02278151 | 0.55702858 | 0 |
| BMD-50 | Pv-ctt002 | 0.02588725 | 0.64802017 | 0 |
| BMD-50 | SSR-IAC 127 | 0.01379645 | 0.49257602 | 0.00599401 |
| BMD-50 | SSR-IAC 188 | 0.01447737 | 0.57112695 | 0.00699301 |
| BMD-50 | SSR-IAC 195 | 0.01550485 | 0.4253171 | 0.04975124 |
| BMD-50 | DQ469392 | 0.01668164 | 0.58481258 | 0.000999 |
| BMD-50 | DQ469393 | 0.01485694 | 0.48497705 | 0.000999 |
| BMD-50 | PvM03 | 0.01503539 | 0.61483838 | 0 |
| BMD-50 | PvM22 | 0.01186147 | 0.39768616 | 0.3125 |
| BMD-50 | BMD-5 | 0.02928359 | 0.5632234 | 0 |
| BMD-50 | BMD-6 | 0.0114293 | 0.31357317 | 0.26315789 |
| BMD-50 | BMD-8 | 0.01429936 | 0.41843629 | 0.01412429 |
| BMD-50 | BMD-13 | 0.01493277 | 0.61877688 | 0.001998 |
| BMD-50 | BMD-18 | 0.02536644 | 0.61748474 | 0 |
| BMD-50 | BMD-23 | 0.01904633 | 0.49771136 | 0.00599401 |
| BMD-50 | BMD-26 | 0.01885633 | 0.60927401 | 0 |
| BMD-50 | BMD-29 | 0.0212089 | 0.55629178 | 0.001998 |
| BMD-50 | BMD-31 | 0.01636195 | 0.48230752 | 0 |
| BMD-50 | BMD-48 | 0.01504791 | 0.40242827 | 0.10309278 |
| BMD-50 | BMD-35 | 0.02038333 | 0.46529108 | 0 |
| BMD-50 | BMD-47 | 0.01804299 | 0.56767831 | 0.00599401 |
| BMD-51 | J01263 | 0.0137102 | 0.61937149 | 0.04807692 |
| BMD-51 | PV-at001 | 0.01268653 | 0.5036219 | 0.01062699 |
| BMD-51 | PV-ag003 | 0.01671215 | 0.53762555 | 0 |
| BMD-51 | PV-ag005 | 0.01565573 | 0.54827856 | 0.02949853 |
| BMD-51 | Pv-ctt002 | 0.01950343 | 0.6926108 | 0 |
| BMD-51 | SSR-IAC 127 | 0.01036217 | 0.48005682 | 0.07575758 |
| BMD-51 | SSR-IAC 188 | 0.01115439 | 0.59802612 | 0.14925373 |
| BMD-51 | SSR-IAC 195 | 0.01608165 | 0.49115056 | 0.000999 |
| BMD-51 | DQ469392 | 0.01261535 | 0.6085395 | 0.0210084 |
| BMD-51 | DQ469393 | 0.01213666 | 0.53673034 | 0.09090909 |
| BMD-51 | PvM03 | 0.01507735 | 0.68161773 | 0 |
| BMD-51 | PvM22 | 0.01234655 | 0.4806422 | 0.17241379 |
| BMD-51 | BMD-5 | 0.01944862 | 0.57374331 | 0.000999 |
| BMD-51 | BMD-6 | 0.01504407 | 0.41408509 | 0.05813953 |
| BMD-51 | BMD-8 | 0.01475197 | 0.51071194 | 0.03533569 |
| BMD-51 | BMD-13 | 0.01466476 | 0.66447925 | 0 |
| BMD-51 | BMD-18 | 0.01966393 | 0.65848598 | 0 |
| BMD-51 | BMD-23 | 0.01662067 | 0.54273553 | 0.001998 |
| BMD-51 | BMD-26 | 0.01789359 | 0.6376093 | 0 |
| BMD-51 | BMD-29 | 0.01742567 | 0.55815907 | 0.03289474 |
| BMD-51 | BMD-31 | 0.01285334 | 0.52534565 | 0.03717472 |
| BMD-51 | BMD-48 | 0.016113 | 0.51683041 | 0.02164502 |
| BMD-51 | BMD-35 | 0.0146614 | 0.50062617 | 0.001998 |
| BMD-51 | BMD-47 | 0.01200693 | 0.55600895 | 0.03952569 |
| BMD-51 | BMD-50 | 0.03690957 | 0.60778296 | 0 |
| BMD-12 | J01263 | 0.01255595 | 0.53659289 | 0.15625 |
| BMD-12 | PV-at001 | 0.01077713 | 0.45808973 | 0.02898551 |
| BMD-12 | PV-ag003 | 0.01477239 | 0.46960687 | 0.003996 |
| BMD-12 | PV-ag005 | 0.01559469 | 0.55032958 | 0.01317523 |
| BMD-12 | Pv-ctt002 | 0.00991738 | 0.55047955 | 0.66666667 |
| BMD-12 | SSR-IAC 127 | 0.01366527 | 0.50052695 | 0.03257329 |
| BMD-12 | SSR-IAC 188 | 0.01001181 | 0.5509263 | 0.29411765 |
| BMD-12 | SSR-IAC 195 | 0.00894594 | 0.38621006 | 0.4 |
| BMD-12 | DQ469392 | 0.01109659 | 0.53831631 | 0.22727273 |
| BMD-12 | DQ469393 | 0.01020239 | 0.44209553 | 0.25641026 |
| BMD-12 | PvM03 | 0.01275792 | 0.63552052 | 0.04878049 |
| BMD-12 | PvM22 | 0.00975631 | 0.40935957 | 0.22222222 |
| BMD-12 | BMD-5 | 0.01602922 | 0.49800065 | 0.00699301 |
| BMD-12 | BMD-6 | 0.01220619 | 0.36111687 | 0.22727273 |
| BMD-12 | BMD-8 | 0.02082711 | 0.55057249 | 0 |
| BMD-12 | BMD-13 | 0.01185668 | 0.59530659 | 0.2173913 |
| BMD-12 | BMD-18 | 0.02106873 | 0.59275012 | 0 |
| BMD-12 | BMD-23 | 0.01065508 | 0.45129887 | 0.04587156 |
| BMD-12 | BMD-26 | 0.01167652 | 0.54827429 | 0.24390244 |
| BMD-12 | BMD-29 | 0.01296713 | 0.47929007 | 0.11904762 |
| BMD-12 | BMD-31 | 0.01082442 | 0.44030946 | 0.2 |
| BMD-12 | BMD-48 | 0.01679339 | 0.44201041 | 0.06578947 |
| BMD-12 | BMD-35 | 0.01703978 | 0.47797892 | 0.02688172 |
| BMD-12 | BMD-47 | 0.0105767 | 0.50277071 | 0.52631579 |
| BMD-12 | BMD-50 | 0.01506594 | 0.43177433 | 0.01282051 |
| BMD-12 | BMD-51 | 0.01559773 | 0.52825037 | 0.001998 |
| X21 | J01263 | 0.0153893 | 0.67392818 | 0 |
| X21 | PV-at001 | 0.01151192 | 0.51068897 | 0.14492754 |
| X21 | PV-ag003 | 0.01184008 | 0.51026838 | 0.1754386 |
| X21 | PV-ag005 | 0.02233097 | 0.67700367 | 0 |
| X21 | Pv-ctt002 | 0.02140724 | 0.73620408 | 0 |
| X21 | SSR-IAC 127 | 0.01196349 | 0.55678663 | 0.20833333 |
| X21 | SSR-IAC 188 | 0.01377305 | 0.65475885 | 0.04878049 |
| X21 | SSR-IAC 195 | 0.01878762 | 0.56100568 | 0.001998 |
| X21 | DQ469392 | 0.01152029 | 0.61231682 | 0.05747126 |
| X21 | DQ469393 | 0.00873371 | 0.4793517 | 0.625 |
| X21 | PvM03 | 0.0127375 | 0.67380771 | 0.001998 |
| X21 | PvM22 | 0.00924854 | 0.44678073 | 0.38461538 |
| X21 | BMD-5 | 0.0163948 | 0.53948542 | 0 |
| X21 | BMD-6 | 0.02195917 | 0.48248263 | 0.03205128 |
| X21 | BMD-8 | 0.01637564 | 0.58203432 | 0 |
| X21 | BMD-13 | 0.0122998 | 0.66792529 | 0.04784689 |
| X21 | BMD-18 | 0.01385608 | 0.58513399 | 0.004995 |
| X21 | BMD-23 | 0.02190875 | 0.62622226 | 0 |
| X21 | BMD-26 | 0.01593086 | 0.61482295 | 0.001998 |
| X21 | BMD-29 | 0.01735536 | 0.60715758 | 0.01002004 |
| X21 | BMD-31 | 0.01305983 | 0.54715377 | 0.11904762 |
| X21 | BMD-48 | 0.01147012 | 0.48815173 | 0.20408163 |
| X21 | BMD-35 | 0.01130461 | 0.49519871 | 0.24390244 |
| X21 | BMD-47 | 0.01322445 | 0.60079177 | 0.04608295 |
| X21 | BMD-50 | 0.04076365 | 0.58912364 | 0 |
| X21 | BMD-51 | 0.04061033 | 0.64996687 | 0 |
| X21 | BMD-12 | 0.01209668 | 0.48623613 | 0.08547009 |
| X 34 | J01263 | 0.01381629 | 0.64539248 | 0.02475248 |
| X 34 | PV-at001 | 0.01339377 | 0.55625949 | 0.23255814 |
| X 34 | PV-ag003 | 0.01474177 | 0.55024938 | 0.01088139 |
| X 34 | PV-ag005 | 0.01358007 | 0.6020575 | 0.01295337 |
| X 34 | Pv-ctt002 | 0.01116707 | 0.6330628 | 0.16393443 |
| X 34 | SSR-IAC 127 | 0.01032756 | 0.54139768 | 0.43478261 |
| X 34 | SSR-IAC 188 | 0.01158108 | 0.65502899 | 0.03076923 |
| X 34 | SSR-IAC 195 | 0.01339707 | 0.51007167 | 0.13333333 |
| X 34 | DQ469392 | 0.01138681 | 0.60794795 | 0.83333333 |
| X 34 | DQ469393 | 0.01446859 | 0.58565734 | 0.09615385 |
| X 34 | PvM03 | 0.0101939 | 0.65671971 | 0.66666667 |
| X 34 | PvM22 | 0.01073995 | 0.49685449 | 0.38461538 |
| X 34 | BMD-5 | 0.01166833 | 0.49710198 | 0.04524887 |
| X 34 | BMD-6 | 0.00976084 | 0.41657231 | 0.37037037 |
| X 34 | BMD-8 | 0.01390103 | 0.55293688 | 0.03333333 |
| X 34 | BMD-13 | 0.01312357 | 0.67939763 | 0.03546099 |
| X 34 | BMD-18 | 0.01776555 | 0.6655944 | 0 |
| X 34 | BMD-23 | 0.01314021 | 0.54496183 | 0.71428571 |
| X 34 | BMD-26 | 0.01055369 | 0.59737709 | 0.28571429 |
| X 34 | BMD-29 | 0.01181298 | 0.5674949 | 0.04048583 |
| X 34 | BMD-31 | 0.01706096 | 0.58416434 | 0.004995 |
| X 34 | BMD-48 | 0.01144476 | 0.50794806 | 0.52631579 |
| X 34 | BMD-35 | 0.01438905 | 0.52148715 | 0.02849003 |
| X 34 | BMD-47 | 0.01191687 | 0.58797924 | 0.4 |
| X 34 | BMD-50 | 0.01399619 | 0.50679599 | 0.00899101 |
| X 34 | BMD-51 | 0.01497596 | 0.5907777 | 0.000999 |
| X 34 | BMD-12 | 0.01274873 | 0.52865495 | 0.12048193 |
| X 34 | X21 | 0.00930449 | 0.53109625 | 0.5 |
| X40 | J01263 | 0.0125508 | 0.55138826 | 0.0273224 |
| X40 | PV-at001 | 0.00983322 | 0.43593106 | 0.5 |
| X40 | PV-ag003 | 0.01290097 | 0.46183862 | 0.07246377 |
| X40 | PV-ag005 | 0.01414197 | 0.52202101 | 0.12987013 |
| X40 | Pv-ctt002 | 0.01395034 | 0.59187646 | 0.17857143 |
| X40 | SSR-IAC 127 | 0.00898994 | 0.45372382 | 0.34482759 |
| X40 | SSR-IAC 188 | 0.01271495 | 0.57764744 | 0.12195122 |
| X40 | SSR-IAC 195 | 0.01988175 | 0.4585212 | 0.03731343 |
| X40 | DQ469392 | 0.01297023 | 0.55711312 | 0.25641026 |
| X40 | DQ469393 | 0.01229409 | 0.49239932 | 0.11904762 |
| X40 | PvM03 | 0.00963645 | 0.5690576 | 0.71428571 |
| X40 | PvM22 | 0.01680733 | 0.49522249 | 0.000999 |
| X40 | BMD-5 | 0.01096152 | 0.4372052 | 0.25641026 |
| X40 | BMD-6 | 0.01341879 | 0.38287614 | 0.11627907 |
| X40 | BMD-8 | 0.01287616 | 0.44649974 | 0.11627907 |
| X40 | BMD-13 | 0.00897526 | 0.5531651 | 0.55555556 |
| X40 | BMD-18 | 0.0113931 | 0.51111371 | 0.01396648 |
| X40 | BMD-23 | 0.01772256 | 0.48798324 | 0.20408163 |
| X40 | BMD-26 | 0.01095828 | 0.55114601 | 0.01043841 |
| X40 | BMD-29 | 0.01304427 | 0.48711665 | 0.08 |
| X40 | BMD-31 | 0.01681855 | 0.47973031 | 0.004995 |
| X40 | BMD-48 | 0.01289148 | 0.43149708 | 0.66666667 |
| X40 | BMD-35 | 0.01297111 | 0.43807617 | 0.04694836 |
| X40 | BMD-47 | 0.02398381 | 0.57891921 | 0.02994012 |
| X40 | BMD-50 | 0.02597011 | 0.51966808 | 0.001998 |
| X40 | BMD-51 | 0.02128274 | 0.53420717 | 0.002997 |
| X40 | BMD-12 | 0.00994984 | 0.42480126 | 0.2173913 |
| X40 | X21 | 0.01972985 | 0.56671297 | 0.02145923 |
| X40 | X 34 | 0.01386784 | 0.52666058 | 0.3125 |
| X49 | J01263 | 0.01191936 | 0.57395465 | 0.13888889 |
| X49 | PV-at001 | 0.0219056 | 0.55619657 | 0.001998 |
| X49 | PV-ag003 | 0.0121029 | 0.45148964 | 0.3030303 |
| X49 | PV-ag005 | 0.01500379 | 0.56387346 | 0.001998 |
| X49 | Pv-ctt002 | 0.01190837 | 0.59430943 | 0.11627907 |
| X49 | SSR-IAC 127 | 0.01085928 | 0.47715781 | 0.16129032 |
| X49 | SSR-IAC 188 | 0.01644136 | 0.65013582 | 0 |
| X49 | SSR-IAC 195 | 0.01552921 | 0.44118475 | 0.03508772 |
| X49 | DQ469392 | 0.01095746 | 0.5391869 | 0.52631579 |
| X49 | DQ469393 | 0.01270101 | 0.49759234 | 0.03952569 |
| X49 | PvM03 | 0.0135882 | 0.65316205 | 0.00599401 |
| X49 | PvM22 | 0.0129444 | 0.47931156 | 0.0877193 |
| X49 | BMD-5 | 0.01401255 | 0.47043888 | 0.001998 |
| X49 | BMD-6 | 0.01329523 | 0.3730598 | 0.05291005 |
| X49 | BMD-8 | 0.01139994 | 0.45343447 | 0.03184713 |
| X49 | BMD-13 | 0.01036554 | 0.58539997 | 0.10869565 |
| X49 | BMD-18 | 0.01902559 | 0.58185857 | 0.000999 |
| X49 | BMD-23 | 0.01484364 | 0.48189615 | 0.01872659 |
| X49 | BMD-26 | 0.01771414 | 0.59306392 | 0.01219512 |
| X49 | BMD-29 | 0.01735435 | 0.53143045 | 0.02364066 |
| X49 | BMD-31 | 0.01660061 | 0.51068535 | 0.01485884 |
| X49 | BMD-48 | 0.01134364 | 0.41574573 | 0.2 |
| X49 | BMD-35 | 0.01396631 | 0.46426185 | 0.05235602 |
| X49 | BMD-47 | 0.01196445 | 0.53399065 | 0.0136612 |
| X49 | BMD-50 | 0.01506465 | 0.43634244 | 0.03367003 |
| X49 | BMD-51 | 0.0176033 | 0.51036168 | 0 |
| X49 | BMD-12 | 0.00988567 | 0.4172378 | 0.76923077 |
| X49 | X21 | 0.01290439 | 0.52083484 | 0.11904762 |
| X49 | X 34 | 0.01355636 | 0.52241563 | 0.02475248 |
| X49 | X40 | 0.01561208 | 0.48226112 | 0.02777778 |
| X62 | J01263 | 0.0143958 | 0.58247585 | 0.00599401 |
| X62 | PV-at001 | 0.01073483 | 0.47593246 | 0.29411765 |
| X62 | PV-ag003 | 0.01437572 | 0.48065957 | 0.06622517 |
| X62 | PV-ag005 | 0.01103095 | 0.50033704 | 0.76923077 |
| X62 | Pv-ctt002 | 0.00869025 | 0.53562314 | 0.90909091 |
| X62 | SSR-IAC 127 | 0.00942081 | 0.46500325 | 0.5 |
| X62 | SSR-IAC 188 | 0.01204001 | 0.58982075 | 0.23809524 |
| X62 | SSR-IAC 195 | 0.01130429 | 0.41555473 | 0.26315789 |
| X62 | DQ469392 | 0.01361631 | 0.56771575 | 0.20408163 |
| X62 | DQ469393 | 0.01133041 | 0.48483805 | 0.3030303 |
| X62 | PvM03 | 0.01074079 | 0.62248027 | 0.5 |
| X62 | PvM22 | 0.01309108 | 0.45956331 | 0.27027027 |
| X62 | BMD-5 | 0.01937358 | 0.54017123 | 0.000999 |
| X62 | BMD-6 | 0.01142204 | 0.37002361 | 0.35714286 |
| X62 | BMD-8 | 0.01013054 | 0.42579198 | 0.16666667 |
| X62 | BMD-13 | 0.01434571 | 0.63601322 | 0.01824818 |
| X62 | BMD-18 | 0.01539267 | 0.54424161 | 0.01094092 |
| X62 | BMD-23 | 0.0099053 | 0.4487016 | 0.83333333 |
| X62 | BMD-26 | 0.01178614 | 0.57793289 | 0.32258065 |
| X62 | BMD-29 | 0.01281396 | 0.50846756 | 0.10204082 |
| X62 | BMD-31 | 0.01343233 | 0.49317899 | 0.02898551 |
| X62 | BMD-48 | 0.01396819 | 0.46004232 | 0.09803922 |
| X62 | BMD-35 | 0.01645627 | 0.4698392 | 0.002997 |
| X62 | BMD-47 | 0.00924646 | 0.50543129 | 0.71428571 |
| X62 | BMD-50 | 0.01446451 | 0.44142781 | 0.01992032 |
| X62 | BMD-51 | 0.01287069 | 0.50611548 | 0.05555556 |
| X62 | BMD-12 | 0.01295213 | 0.46880038 | 0.13888889 |
| X62 | X21 | 0.00945922 | 0.48248683 | 0.18181818 |
| X62 | X 34 | 0.00711677 | 0.44493908 | 0.90909091 |
| X62 | X40 | 0.01221944 | 0.47433454 | 0.08130081 |
| X62 | X49 | 0.01132887 | 0.45695616 | 0.08928571 |
| X65 | J01263 | 0.01211552 | 0.53604555 | 0.11764706 |
| X65 | PV-at001 | 0.01219586 | 0.49564525 | 0.12195122 |
| X65 | PV-ag003 | 0.01672012 | 0.5299725 | 0.00899101 |
| X65 | PV-ag005 | 0.01268466 | 0.52721508 | 0.08849558 |
| X65 | Pv-ctt002 | 0.01315631 | 0.63214498 | 0.02659574 |
| X65 | SSR-IAC 127 | 0.01471157 | 0.54540935 | 0.03460208 |
| X65 | SSR-IAC 188 | 0.01019306 | 0.56804493 | 0.71428571 |
| X65 | SSR-IAC 195 | 0.01094279 | 0.42958631 | 0.07751938 |
| X65 | DQ469392 | 0.01359428 | 0.57589612 | 0.03134796 |
| X65 | DQ469393 | 0.01814857 | 0.51760733 | 0.003996 |
| X65 | PvM03 | 0.01209349 | 0.62387932 | 0.03861004 |
| X65 | PvM22 | 0.01208127 | 0.46265034 | 0.16129032 |
| X65 | BMD-5 | 0.01375476 | 0.49290376 | 0.04651163 |
| X65 | BMD-6 | 0.01618721 | 0.41722863 | 0.00799201 |
| X65 | BMD-8 | 0.01554599 | 0.50819813 | 0.00699301 |
| X65 | BMD-13 | 0.01247874 | 0.63256239 | 0.03311258 |
| X65 | BMD-18 | 0.01246978 | 0.54036802 | 0.02762431 |
| X65 | BMD-23 | 0.00939643 | 0.44151166 | 0.43478261 |
| X65 | BMD-26 | 0.01347019 | 0.59088526 | 0.01697793 |
| X65 | BMD-29 | 0.01307782 | 0.51012654 | 0.09615385 |
| X65 | BMD-31 | 0.01506748 | 0.50485956 | 0.00899101 |
| X65 | BMD-48 | 0.01512606 | 0.46487416 | 0.1010101 |
| X65 | BMD-35 | 0.01429988 | 0.48443697 | 0.03389831 |
| X65 | BMD-47 | 0.01168774 | 0.53352334 | 0.625 |
| X65 | BMD-50 | 0.01076953 | 0.39351799 | 0.25 |
| X65 | BMD-51 | 0.01835355 | 0.54761422 | 0.003996 |
| X65 | BMD-12 | 0.01207609 | 0.47435737 | 0.05494505 |
| X65 | X21 | 0.0101748 | 0.49437229 | 0.3030303 |
| X65 | X 34 | 0.01270803 | 0.55574615 | 0.13333333 |
| X65 | X40 | 0.01154689 | 0.4286637 | 0.55555556 |
| X65 | X49 | 0.02184 | 0.5655277 | 0 |
| X65 | X62 | 0.0140294 | 0.46633401 | 0.07692308 |
| X87 | J01263 | 0.0129458 | 0.71736295 | 0.07194245 |
| X87 | PV-at001 | 0.01225935 | 0.66502898 | 0.15384615 |
| X87 | PV-ag003 | 0.01172538 | 0.63191545 | 0.1754386 |
| X87 | PV-ag005 | 0.02112168 | 0.77483149 | 0 |
| X87 | Pv-ctt002 | 0.01241203 | 0.75480411 | 0.00899101 |
| X87 | SSR-IAC 127 | 0.01161578 | 0.65716656 | 0.5 |
| X87 | SSR-IAC 188 | 0.01127545 | 0.73038963 | 0.25641026 |
| X87 | SSR-IAC 195 | ? | ? | ? |
| X87 | DQ469392 | 0.01137944 | 0.71199523 | 0.0625 |
| X87 | DQ469393 | 0.01223537 | 0.66228862 | 0.01677852 |
| X87 | PvM03 | 0.01223211 | 0.76845892 | 0.03322259 |
| X87 | PvM22 | 0.01428428 | 0.65019519 | 0 |
| X87 | BMD-5 | 0.01296897 | 0.62954268 | 0.06944444 |
| X87 | BMD-6 | 0.01314599 | 0.56275191 | 0.15873016 |
| X87 | BMD-8 | 0.012408 | 0.62121778 | 0.10204082 |
| X87 | BMD-13 | 0.01327934 | 0.75898998 | 0.000999 |
| X87 | BMD-18 | 0.01238637 | 0.68863653 | 0.15151515 |
| X87 | BMD-23 | 0.01352834 | 0.65801567 | 0.02994012 |
| X87 | BMD-26 | 0.01635651 | 0.74241086 | 0.000999 |
| X87 | BMD-29 | 0.0127088 | 0.68371447 | 0.07092199 |
| X87 | BMD-31 | 0.01672111 | 0.70702506 | 0 |
| X87 | BMD-48 | 0.01202942 | 0.61601494 | 0.03787879 |
| X87 | BMD-35 | 0.01304603 | 0.62488043 | 0.03676471 |
| X87 | BMD-47 | 0.0141729 | 0.70860137 | 0.01221001 |
| X87 | BMD-50 | 0.01457791 | 0.62033951 | 0.004995 |
| X87 | BMD-51 | 0.01183767 | 0.64728462 | 0.17241379 |
| X87 | BMD-12 | 0.01257732 | 0.62282593 | 0.0286533 |
| X87 | X21 | 0.0094236 | 0.63515968 | 0.47619048 |
| X87 | X 34 | 0.01009928 | 0.64297469 | 0.55555556 |
| X87 | X40 | 0.01112951 | 0.61560845 | 0.2 |
| X87 | X49 | 0.01033973 | 0.5978834 | 0.18181818 |
| X87 | X62 | 0.00943441 | 0.59478714 | 0.625 |
| X87 | X65 | 0.01357851 | 0.65532944 | 0.02421308 |
| VR011 | J01263 | 0.01367433 | 0.68927055 | 0.01138952 |
| VR011 | PV-at001 | 0.01519959 | 0.56889209 | 0.00599401 |
| VR011 | PV-ag003 | 0.01878809 | 0.62219004 | 0 |
| VR011 | PV-ag005 | 0.02335616 | 0.70616653 | 0 |
| VR011 | Pv-ctt002 | 0.01856183 | 0.72612168 | 0 |
| VR011 | SSR-IAC 127 | 0.01251643 | 0.59819031 | 0.11363636 |
| VR011 | SSR-IAC 188 | 0.01022405 | 0.65235341 | 0.83333333 |
| VR011 | SSR-IAC 195 | 0.01542315 | 0.59250864 | 0.01319261 |
| VR011 | DQ469392 | 0.01485176 | 0.69096618 | 0.003996 |
| VR011 | DQ469393 | 0.01192631 | 0.58243483 | 0.12345679 |
| VR011 | PvM03 | 0.01446861 | 0.73715343 | 0 |
| VR011 | PvM22 | 0.01123634 | 0.53194202 | 0.55555556 |
| VR011 | BMD-5 | 0.01740754 | 0.57589441 | 0.0621118 |
| VR011 | BMD-6 | 0.01571357 | 0.47181893 | 0.14285714 |
| VR011 | BMD-8 | 0.01283231 | 0.5615023 | 0.06578947 |
| VR011 | BMD-13 | 0.0148129 | 0.73824599 | 0 |
| VR011 | BMD-18 | 0.01585452 | 0.68920234 | 0 |
| VR011 | BMD-23 | 0.02204302 | 0.66044719 | 0 |
| VR011 | BMD-26 | 0.01052909 | 0.62357496 | 0.11764706 |
| VR011 | BMD-29 | 0.01914109 | 0.64172089 | 0.003996 |
| VR011 | BMD-31 | 0.01690816 | 0.59835878 | 0.00599401 |
| VR011 | BMD-48 | 0.02074284 | 0.62191628 | 0 |
| VR011 | BMD-35 | 0.0113411 | 0.49441553 | 0.35714286 |
| VR011 | BMD-47 | 0.01164919 | 0.62167808 | 0.58823529 |
| VR011 | BMD-50 | 0.0249397 | 0.60039085 | 0 |
| VR011 | BMD-51 | 0.01889391 | 0.62862377 | 0 |
| VR011 | BMD-12 | 0.01479994 | 0.57035726 | 0.0128866 |
| VR011 | X21 | 0.02552435 | 0.70987086 | 0 |
| VR011 | X 34 | 0.01033298 | 0.59036687 | 0.23255814 |
| VR011 | X40 | 0.01394826 | 0.57247057 | 0.08 |
| VR011 | X49 | 0.0107822 | 0.52087432 | 0.45454545 |
| VR011 | X62 | 0.01190485 | 0.55013854 | 0.08064516 |
| VR011 | X65 | 0.01082816 | 0.54974108 | 0.14925373 |
| VR011 | X87 | 0.01289413 | 0.73430509 | 0.00899101 |
| VR013 | J01263 | 0.01232238 | 0.60822196 | 0.28571429 |
| VR013 | PV-at001 | 0.00674015 | 0.42157056 | 0.90909091 |
| VR013 | PV-ag003 | 0.0132924 | 0.47837458 | 0.11904762 |
| VR013 | PV-ag005 | 0.01217789 | 0.52473365 | 0.08403361 |
| VR013 | Pv-ctt002 | 0.01016506 | 0.58782929 | 0.35714286 |
| VR013 | SSR-IAC 127 | 0.01178927 | 0.49864846 | 0.21276596 |
| VR013 | SSR-IAC 188 | 0.01097466 | 0.59118998 | 0.25641026 |
| VR013 | SSR-IAC 195 | 0.00866426 | 0.40556618 | 0.71428571 |
| VR013 | DQ469392 | 0.00937305 | 0.53211757 | 0.33333333 |
| VR013 | DQ469393 | 0.01442026 | 0.5412633 | 0.0105042 |
| VR013 | PvM03 | 0.01001595 | 0.62007238 | 0.71428571 |
| VR013 | PvM22 | 0.00682622 | 0.38868045 | 0.90909091 |
| VR013 | BMD-5 | 0.00888585 | 0.44743564 | 0.47619048 |
| VR013 | BMD-6 | 0.00671494 | 0.32936741 | 0.90909091 |
| VR013 | BMD-8 | 0.01347849 | 0.49721642 | 0.1010101 |
| VR013 | BMD-13 | 0.01174421 | 0.65625979 | 0.13157895 |
| VR013 | BMD-18 | 0.00947968 | 0.51130179 | 0.71428571 |
| VR013 | BMD-23 | 0.01129694 | 0.48089271 | 0.13333333 |
| VR013 | BMD-26 | 0.01225822 | 0.59814364 | 0.33333333 |
| VR013 | BMD-29 | 0.00918556 | 0.46786963 | 0.90909091 |
| VR013 | BMD-31 | 0.00880263 | 0.44435658 | 0.76923077 |
| VR013 | BMD-48 | 0.01458366 | 0.4846888 | 0.02808989 |
| VR013 | BMD-35 | 0.00839871 | 0.38615411 | 0.71428571 |
| VR013 | BMD-47 | 0.00784166 | 0.49552732 | 0.76923077 |
| VR013 | BMD-50 | 0.01102977 | 0.40447649 | 0.5 |
| VR013 | BMD-51 | 0.01032564 | 0.47834269 | 0.43478261 |
| VR013 | BMD-12 | 0.008803 | 0.43759225 | 0.5 |
| VR013 | X21 | 0.01128221 | 0.53566181 | 0.22727273 |
| VR013 | X 34 | 0.00694547 | 0.45804901 | 0.90909091 |
| VR013 | X40 | 0.01196314 | 0.47900757 | 0.16129032 |
| VR013 | X49 | 0.00823613 | 0.42831872 | 0.83333333 |
| VR013 | X62 | 0.00880042 | 0.44638521 | 0.52631579 |
| VR013 | X65 | 0.01247156 | 0.50408953 | 0.15625 |
| VR013 | X87 | 0.00994338 | 0.62140135 | 0.47619048 |
| VR013 | VR011 | 0.01609194 | 0.60048426 | 0.001998 |
| VR015 | J01263 | 0.0120141 | 0.50602942 | 0.12987013 |
| VR015 | PV-at001 | 0.0148183 | 0.4530677 | 0.04237288 |
| VR015 | PV-ag003 | 0.01430897 | 0.41540127 | 0.02645503 |
| VR015 | PV-ag005 | 0.01342025 | 0.47356253 | 0.05524862 |
| VR015 | Pv-ctt002 | 0.01264292 | 0.56057383 | 0.01824818 |
| VR015 | SSR-IAC 127 | 0.01246852 | 0.44172906 | 0.16393443 |
| VR015 | SSR-IAC 188 | 0.01274136 | 0.53711318 | 0.10638298 |
| VR015 | SSR-IAC 195 | 0.02131678 | 0.4479537 | 0 |
| VR015 | DQ469392 | 0.01339023 | 0.54849035 | 0.0147929 |
| VR015 | DQ469393 | 0.01091286 | 0.41882889 | 0.1754386 |
| VR015 | PvM03 | 0.0109277 | 0.58625317 | 0.5 |
| VR015 | PvM22 | 0.01060832 | 0.37077178 | 0.43478261 |
| VR015 | BMD-5 | 0.01470139 | 0.43739194 | 0.01335113 |
| VR015 | BMD-6 | 0.01283898 | 0.33829735 | 0.0625 |
| VR015 | BMD-8 | 0.01381659 | 0.40466118 | 0.09433962 |
| VR015 | BMD-13 | 0.01161485 | 0.56726553 | 0.02272727 |
| VR015 | BMD-18 | 0.02017803 | 0.57084237 | 0 |
| VR015 | BMD-23 | 0.01669871 | 0.47067541 | 0.004995 |
| VR015 | BMD-26 | 0.00794229 | 0.4705004 | 0.76923077 |
| VR015 | BMD-29 | 0.01343645 | 0.46725691 | 0.22727273 |
| VR015 | BMD-31 | 0.00740206 | 0.34766586 | 0.76923077 |
| VR015 | BMD-48 | 0.01289469 | 0.37708678 | 0.19230769 |
| VR015 | BMD-35 | 0.01650657 | 0.42072129 | 0.03846154 |
| VR015 | BMD-47 | 0.0119086 | 0.49027238 | 0.04347826 |
| VR015 | BMD-50 | 0.02040626 | 0.41920236 | 0 |
| VR015 | BMD-51 | 0.01260516 | 0.42836185 | 0.03401361 |
| VR015 | BMD-12 | 0.01215594 | 0.40837665 | 0.13888889 |
| VR015 | X21 | 0.01397269 | 0.48315668 | 0.05747126 |
| VR015 | X 34 | 0.01050046 | 0.47671887 | 0.2173913 |
| VR015 | X40 | 0.01596963 | 0.44964572 | 0.03690037 |
| VR015 | X49 | 0.0140758 | 0.44822067 | 0.0308642 |
| VR015 | X62 | 0.00947636 | 0.35987363 | 0.76923077 |
| VR015 | X65 | 0.01444289 | 0.47786888 | 0.00699301 |
| VR015 | X87 | 0.0106662 | 0.55170938 | 0.3030303 |
| VR015 | VR011 | 0.01469716 | 0.55602248 | 0.05681818 |
| VR015 | VR013 | 0.01540812 | 0.49224405 | 0 |
| VR016 | J01263 | 0.01276906 | 0.64023006 | 0.001998 |
| VR016 | PV-at001 | 0.01419753 | 0.60327539 | 0.0128866 |
| VR016 | PV-ag003 | 0.0155459 | 0.57178539 | 0.02096436 |
| VR016 | PV-ag005 | 0.01356767 | 0.60786191 | 0.05 |
| VR016 | Pv-ctt002 | 0.01259354 | 0.65594862 | 0.03533569 |
| VR016 | SSR-IAC 127 | 0.01241474 | 0.5706769 | 0.26315789 |
| VR016 | SSR-IAC 188 | 0.00820027 | 0.59043819 | 0.90909091 |
| VR016 | SSR-IAC 195 | 0.013033 | 0.51334971 | 0.10638298 |
| VR016 | DQ469392 | 0.01424343 | 0.66221596 | 0.01219512 |
| VR016 | DQ469393 | 0.01522113 | 0.60636805 | 0.001998 |
| VR016 | PvM03 | 0.01241597 | 0.68950216 | 0.00899101 |
| VR016 | PvM22 | 0.01551353 | 0.536758 | 0.025 |
| VR016 | BMD-5 | 0.01397417 | 0.52612287 | 0.01219512 |
| VR016 | BMD-6 | 0.01055487 | 0.41631478 | 0.2 |
| VR016 | BMD-8 | 0.01498375 | 0.56649027 | 0 |
| VR016 | BMD-13 | 0.0148442 | 0.72149448 | 0.000999 |
| VR016 | BMD-18 | 0.01346379 | 0.58620973 | 0.04201681 |
| VR016 | BMD-23 | 0.01416605 | 0.56935883 | 0.01324503 |
| VR016 | BMD-26 | 0.01341513 | 0.64669115 | 0.01440922 |
| VR016 | BMD-29 | 0.01231269 | 0.59032414 | 0.01795332 |
| VR016 | BMD-31 | 0.0160706 | 0.61131329 | 0.00799201 |
| VR016 | BMD-48 | 0.01409309 | 0.52899419 | 0.03676471 |
| VR016 | BMD-35 | 0.01612341 | 0.55596268 | 0 |
| VR016 | BMD-47 | 0.01265057 | 0.62767828 | 0.09708738 |
| VR016 | BMD-50 | 0.01525145 | 0.49756861 | 0.003996 |
| VR016 | BMD-51 | 0.01564919 | 0.57546938 | 0.01388889 |
| VR016 | BMD-12 | 0.00866647 | 0.46731761 | 0.55555556 |
| VR016 | X21 | 0.01575418 | 0.6069157 | 0.000999 |
| VR016 | X 34 | 0.01354735 | 0.5946959 | 0.004995 |
| VR016 | X40 | 0.00985467 | 0.50323304 | 0.1754386 |
| VR016 | X49 | 0.01362949 | 0.53748494 | 0.07142857 |
| VR016 | X62 | 0.01094504 | 0.52395056 | 0.34482759 |
| VR016 | X65 | 0.01592799 | 0.60742713 | 0.000999 |
| VR016 | X87 | 0.01164066 | 0.68955743 | 0.02217295 |
| VR016 | VR011 | 0.01148735 | 0.60280635 | 0.18867925 |
| VR016 | VR013 | 0.01009546 | 0.52733895 | 0.55555556 |
| VR016 | VR015 | 0.01115015 | 0.46594173 | 0.27027027 |
| VR018 | J01263 | 0.01525837 | 0.73503769 | 0 |
| VR018 | PV-at001 | 0.01781948 | 0.70282039 | 0 |
| VR018 | PV-ag003 | 0.01196255 | 0.57119974 | 0.125 |
| VR018 | PV-ag005 | 0.01776957 | 0.69257766 | 0 |
| VR018 | Pv-ctt002 | 0.01287484 | 0.72193073 | 0.08403361 |
| VR018 | SSR-IAC 127 | 0.01063217 | 0.60911841 | 0.06666667 |
| VR018 | SSR-IAC 188 | 0.01336297 | 0.72386745 | 0 |
| VR018 | SSR-IAC 195 | 0.01671678 | 0.61329959 | 0.003996 |
| VR018 | DQ469392 | 0.01225601 | 0.69178589 | 0.07092199 |
| VR018 | DQ469393 | 0.01380531 | 0.64003785 | 0.01408451 |
| VR018 | PvM03 | 0.0102072 | 0.69765246 | 0.83333333 |
| VR018 | PvM22 | 0.00963896 | 0.53408792 | 0.19607843 |
| VR018 | BMD-5 | 0.01775715 | 0.6161506 | 0 |
| VR018 | BMD-6 | 0.0148863 | 0.50714451 | 0.03267974 |
| VR018 | BMD-8 | 0.01379855 | 0.5957159 | 0.01036269 |
| VR018 | BMD-13 | 0.01356444 | 0.75943588 | 0.00799201 |
| VR018 | BMD-18 | 0.01484456 | 0.6830197 | 0.000999 |
| VR018 | BMD-23 | 0.01565431 | 0.62557395 | 0.05882353 |
| VR018 | BMD-26 | 0.01056293 | 0.65341542 | 0.55555556 |
| VR018 | BMD-29 | 0.01398426 | 0.65459958 | 0.00799201 |
| VR018 | BMD-31 | 0.01632732 | 0.63879237 | 0.003996 |
| VR018 | BMD-48 | 0.01376244 | 0.56476634 | 0.05681818 |
| VR018 | BMD-35 | 0.01427452 | 0.60184972 | 0.01172333 |
| VR018 | BMD-47 | 0.0152673 | 0.67269157 | 0.00799201 |
| VR018 | BMD-50 | 0.0227592 | 0.65546963 | 0 |
| VR018 | BMD-51 | 0.01368036 | 0.63602314 | 0.01841621 |
| VR018 | BMD-12 | 0.01288194 | 0.57190221 | 0.04878049 |
| VR018 | X21 | 0.01840073 | 0.65759491 | 0.004995 |
| VR018 | X 34 | 0.01251347 | 0.63985021 | 0.06369427 |
| VR018 | X40 | 0.01584348 | 0.6119412 | 0.02298851 |
| VR018 | X49 | 0.01522378 | 0.61074074 | 0.000999 |
| VR018 | X62 | 0.01539878 | 0.63339217 | 0.04761905 |
| VR018 | X65 | 0.01339903 | 0.61416666 | 0.02754821 |
| VR018 | X87 | 0.01349143 | 0.76046486 | 0.004995 |
| VR018 | VR011 | 0.01376645 | 0.70671764 | 0.02173913 |
| VR018 | VR013 | 0.00758633 | 0.52865692 | 0.90909091 |
| VR018 | VR015 | 0.01399855 | 0.58790314 | 0.0136612 |
| VR018 | VR016 | 0.01332222 | 0.67617506 | 0.003996 |
| VR022 | J01263 | 0.01363625 | 0.61578901 | 0.00599401 |
| VR022 | PV-at001 | 0.0156377 | 0.54487948 | 0.01538462 |
| VR022 | PV-ag003 | 0.0124394 | 0.49219218 | 0.04651163 |
| VR022 | PV-ag005 | 0.01560822 | 0.60312619 | 0 |
| VR022 | Pv-ctt002 | 0.01339844 | 0.64432938 | 0.01005025 |
| VR022 | SSR-IAC 127 | 0.01409872 | 0.57776616 | 0.001998 |
| VR022 | SSR-IAC 188 | 0.01130703 | 0.62218055 | 0.3125 |
| VR022 | SSR-IAC 195 | 0.0113788 | 0.46345155 | 0.10204082 |
| VR022 | DQ469392 | 0.01444411 | 0.62650593 | 0.04132231 |
| VR022 | DQ469393 | 0.01470345 | 0.56251304 | 0.000999 |
| VR022 | PvM03 | 0.01256746 | 0.65156965 | 0.01219512 |
| VR022 | PvM22 | 0.01361966 | 0.5107029 | 0.001998 |
| VR022 | BMD-5 | 0.01909535 | 0.57603644 | 0.001998 |
| VR022 | BMD-6 | 0.0210892 | 0.47136339 | 0 |
| VR022 | BMD-8 | 0.0188183 | 0.55980456 | 0 |
| VR022 | BMD-13 | 0.01358962 | 0.68572334 | 0 |
| VR022 | BMD-18 | 0.01687346 | 0.57531281 | 0.000999 |
| VR022 | BMD-23 | 0.01500468 | 0.54755664 | 0.01538462 |
| VR022 | BMD-26 | 0.01212952 | 0.59611409 | 0.00599401 |
| VR022 | BMD-29 | 0.00978036 | 0.51054272 | 0.25 |
| VR022 | BMD-31 | 0.01911669 | 0.59881088 | 0 |
| VR022 | BMD-48 | 0.0178091 | 0.54532989 | 0.03571429 |
| VR022 | BMD-35 | 0.01102641 | 0.45422142 | 0.02531646 |
| VR022 | BMD-47 | 0.01339252 | 0.58785676 | 0.11111111 |
| VR022 | BMD-50 | 0.01746744 | 0.52912257 | 0 |
| VR022 | BMD-51 | 0.01042289 | 0.50330037 | 0.23809524 |
| VR022 | BMD-12 | 0.01130654 | 0.47252034 | 0.17857143 |
| VR022 | X21 | 0.01057127 | 0.52947913 | 0.14925373 |
| VR022 | X 34 | 0.01328974 | 0.58841181 | 0.05780347 |
| VR022 | X40 | 0.01126122 | 0.47553716 | 0.10204082 |
| VR022 | X49 | 0.01243358 | 0.51017058 | 0.04166667 |
| VR022 | X62 | 0.01012009 | 0.467756 | 0.3125 |
| VR022 | X65 | 0.01089943 | 0.48071003 | 0.0862069 |
| VR022 | X87 | 0.01200285 | 0.66052935 | 0.32258065 |
| VR022 | VR011 | 0.01007275 | 0.54726709 | 0.47619048 |
| VR022 | VR013 | 0.01125314 | 0.5081999 | 0.5 |
| VR022 | VR015 | 0.01707888 | 0.48108424 | 0.004995 |
| VR022 | VR016 | 0.01479867 | 0.57904169 | 0.000999 |
| VR022 | VR018 | 0.01022827 | 0.58666609 | 0.27027027 |
| VR022 | J01263 | 0.01314586 | 0.69867365 | 0.01727116 |
| VR022 | PV-at001 | 0.01308016 | 0.62936915 | 0.0152207 |
| VR022 | PV-ag003 | 0.01251979 | 0.59128075 | 0.04310345 |
| VR022 | PV-ag005 | 0.01503725 | 0.67133908 | 0.00599401 |
| VR022 | Pv-ctt002 | 0.01610632 | 0.78375267 | 0 |
| VR022 | SSR-IAC 127 | 0.01057795 | 0.60555348 | 0.1754386 |
| VR022 | SSR-IAC 188 | 0.01507334 | 0.75118505 | 0 |
| VR022 | SSR-IAC 195 | 0.01119015 | 0.51883613 | 0.2 |
| VR022 | DQ469392 | 0.01201403 | 0.66509641 | 0.26315789 |
| VR022 | DQ469393 | 0.01488231 | 0.64227233 | 0.0297619 |
| VR022 | PvM03 | 0.01443263 | 0.77821276 | 0 |
| VR022 | PvM22 | 0.01394667 | 0.58923265 | 0.05291005 |
| VR022 | BMD-5 | 0.02032726 | 0.68167167 | 0 |
| VR022 | BMD-6 | 0.01235335 | 0.48695378 | 0.16129032 |
| VR022 | BMD-8 | 0.01633222 | 0.64091076 | 0.000999 |
| VR022 | BMD-13 | 0.01247415 | 0.73505117 | 0.01025641 |
| VR022 | BMD-18 | 0.01380107 | 0.66542891 | 0.01166861 |
| VR022 | BMD-23 | 0.01661101 | 0.65089952 | 0.01015228 |
| VR022 | BMD-26 | 0.01470558 | 0.71629866 | 0.0129199 |
| VR022 | BMD-29 | 0.01283106 | 0.64417476 | 0.09174312 |
| VR022 | BMD-31 | 0.0122961 | 0.60582167 | 0.01191895 |
| VR022 | BMD-48 | 0.01248385 | 0.57783048 | 0.0122549 |
| VR022 | BMD-35 | 0.01436272 | 0.59778042 | 0.000999 |
| VR022 | BMD-47 | 0.01165725 | 0.65286521 | 0.0990099 |
| VR022 | BMD-50 | 0.0153737 | 0.59073952 | 0 |
| VR022 | BMD-51 | 0.01450296 | 0.66869878 | 0.000999 |
| VR022 | BMD-12 | 0.01174533 | 0.59217294 | 0.15384615 |
| VR022 | X21 | 0.01483212 | 0.68129296 | 0.00799201 |
| VR022 | X 34 | 0.01204595 | 0.65779672 | 0.0390625 |
| VR022 | X40 | 0.01212888 | 0.58127559 | 0.12987013 |
| VR022 | X49 | 0.01271788 | 0.56593813 | 0.01422475 |
| VR022 | X62 | 0.01101104 | 0.57300548 | 0.29411765 |
| VR022 | X65 | 0.01303063 | 0.62771109 | 0.01324503 |
| VR022 | X87 | 0.01068819 | 0.718161 | 0.35714286 |
| VR022 | VR011 | 0.0127803 | 0.6979081 | 0.0141844 |
| VR022 | VR013 | 0.0104029 | 0.60130246 | 0.58823529 |
| VR022 | VR015 | 0.01537982 | 0.57967392 | 0.01510574 |
| VR022 | VR016 | 0.01468118 | 0.69234221 | 0 |
| VR022 | VR018 | 0.01506567 | 0.73383257 | 0 |
| VR022 | VR022 | 0.02019736 | 0.69707834 | 0 |
| VR024 | J01263 | 0.01176495 | 0.50056532 | 0.20833333 |
| VR024 | PV-at001 | 0.01103112 | 0.42063695 | 0.24390244 |
| VR024 | PV-ag003 | 0.0161717 | 0.46875841 | 0.000999 |
| VR024 | PV-ag005 | 0.01292245 | 0.44707805 | 0.15384615 |
| VR024 | Pv-ctt002 | 0.01450317 | 0.5776965 | 0.04444444 |
| VR024 | SSR-IAC 127 | 0.01068399 | 0.41954261 | 0.41666667 |
| VR024 | SSR-IAC 188 | 0.0118809 | 0.53745383 | 0.12820513 |
| VR024 | SSR-IAC 195 | 0.01768758 | 0.44704743 | 0.000999 |
| VR024 | DQ469392 | 0.02176626 | 0.57341827 | 0.00899101 |
| VR024 | DQ469393 | 0.00957103 | 0.41347631 | 0.11111111 |
| VR024 | PvM03 | 0.01195653 | 0.57749692 | 0.06849315 |
| VR024 | PvM22 | 0.0077821 | 0.35169001 | 0.38461538 |
| VR024 | BMD-5 | 0.01299653 | 0.40893967 | 0.125 |
| VR024 | BMD-6 | 0.01205185 | 0.30630873 | 0.13513514 |
| VR024 | BMD-8 | 0.01170116 | 0.41846628 | 0.10204082 |
| VR024 | BMD-13 | 0.01111181 | 0.55101506 | 0.12048193 |
| VR024 | BMD-18 | 0.01409305 | 0.50971094 | 0.01048218 |
| VR024 | BMD-23 | 0.01302752 | 0.44252532 | 0.05376344 |
| VR024 | BMD-26 | 0.01440265 | 0.53742339 | 0.23255814 |
| VR024 | BMD-29 | 0.01139483 | 0.42333961 | 0.3030303 |
| VR024 | BMD-31 | 0.01083521 | 0.40477639 | 0.10989011 |
| VR024 | BMD-48 | 0.01637951 | 0.39677958 | 0.55555556 |
| VR024 | BMD-35 | 0.0098701 | 0.36028074 | 0.22727273 |
| VR024 | BMD-47 | 0.01185023 | 0.48852816 | 0.04672897 |
| VR024 | BMD-50 | 0.02220717 | 0.42408631 | 0.01785714 |
| VR024 | BMD-51 | 0.01896507 | 0.46421491 | 0.01623377 |
| VR024 | BMD-12 | 0.02070516 | 0.49404256 | 0.01137656 |
| VR024 | X21 | 0.02532066 | 0.49870589 | 0.02096436 |
| VR024 | X 34 | 0.00877682 | 0.43512411 | 0.20408163 |
| VR024 | X40 | 0.0122046 | 0.41285995 | 0.09009009 |
| VR024 | X49 | 0.01245537 | 0.39003095 | 0.19230769 |
| VR024 | X62 | 0.01720651 | 0.42624373 | 0.58823529 |
| VR024 | X65 | 0.01171198 | 0.4185688 | 0.08196721 |
| VR024 | X87 | 0.01161686 | 0.5684645 | 0.09009009 |
| VR024 | VR011 | 0.01267785 | 0.50813308 | 0.01992032 |
| VR024 | VR013 | 0.007426 | 0.38493419 | 0.55555556 |
| VR024 | VR015 | 0.02233692 | 0.44365722 | 0.000999 |
| VR024 | VR016 | 0.00900832 | 0.41433408 | 0.52631579 |
| VR024 | VR018 | 0.00962772 | 0.49834217 | 0.13157895 |
| VR024 | VR022 | 0.01307368 | 0.45994326 | 0.05128205 |
| VR024 | VR022 | 0.00984773 | 0.47555454 | 0.83333333 |
| VR025 | J01263 | 0.01515391 | 0.60550181 | 0.001998 |
| VR025 | PV-at001 | 0.01144745 | 0.46347916 | 0.14492754 |
| VR025 | PV-ag003 | 0.02000003 | 0.51782309 | 0 |
| VR025 | PV-ag005 | 0.01767694 | 0.54426726 | 0.003996 |
| VR025 | Pv-ctt002 | 0.01682162 | 0.654384 | 0 |
| VR025 | SSR-IAC 127 | 0.01139655 | 0.48450611 | 0.12048193 |
| VR025 | SSR-IAC 188 | 0.01078512 | 0.56184156 | 0.41666667 |
| VR025 | SSR-IAC 195 | 0.01488441 | 0.41504879 | 0.2 |
| VR025 | DQ469392 | 0.01527761 | 0.60532642 | 0.01264223 |
| VR025 | DQ469393 | 0.01162548 | 0.47519029 | 0.58823529 |
| VR025 | PvM03 | 0.0130198 | 0.6219025 | 0.0304878 |
| VR025 | PvM22 | 0.00867153 | 0.38335316 | 0.71428571 |
| VR025 | BMD-5 | 0.01807859 | 0.5040992 | 0.01572327 |
| VR025 | BMD-6 | 0.01402242 | 0.37385642 | 0.12658228 |
| VR025 | BMD-8 | 0.01833458 | 0.49281226 | 0.00899101 |
| VR025 | BMD-13 | 0.01686747 | 0.68683138 | 0 |
| VR025 | BMD-18 | 0.0169828 | 0.56858749 | 0 |
| VR025 | BMD-23 | 0.01545937 | 0.54294913 | 0.00599401 |
| VR025 | BMD-26 | 0.01179058 | 0.54875693 | 0.14925373 |
| VR025 | BMD-29 | 0.01636879 | 0.51382507 | 0.03875969 |
| VR025 | BMD-31 | 0.0122996 | 0.46246266 | 0.01984127 |
| VR025 | BMD-48 | 0.01466816 | 0.44976608 | 0.02237136 |
| VR025 | BMD-35 | 0.02055839 | 0.52217711 | 0 |
| VR025 | BMD-47 | 0.01298244 | 0.55986056 | 0.03278689 |
| VR025 | BMD-50 | 0.02431198 | 0.50317267 | 0 |
| VR025 | BMD-51 | 0.03420493 | 0.62243229 | 0 |
| VR025 | BMD-12 | 0.01157984 | 0.42835709 | 0.66666667 |
| VR025 | X21 | 0.02303145 | 0.56089081 | 0 |
| VR025 | X 34 | 0.01162263 | 0.53494539 | 0.0625 |
| VR025 | X40 | 0.02142262 | 0.50009812 | 0.001998 |
| VR025 | X49 | 0.0119928 | 0.43857508 | 0.08064516 |
| VR025 | X62 | 0.01238804 | 0.45789752 | 0.12195122 |
| VR025 | X65 | 0.00991843 | 0.39952405 | 0.52631579 |
| VR025 | X87 | 0.01265635 | 0.63829828 | 0.02237136 |
| VR025 | VR011 | 0.01716646 | 0.6104139 | 0.001998 |
| VR025 | VR013 | 0.00928122 | 0.43764469 | 0.55555556 |
| VR025 | VR015 | 0.01105666 | 0.37230633 | 0.45454545 |
| VR025 | VR016 | 0.01987333 | 0.57290233 | 0 |
| VR025 | VR018 | 0.01360216 | 0.58906668 | 0.06097561 |
| VR025 | VR022 | 0.0140115 | 0.51939599 | 0.01890359 |
| VR025 | VR022 | 0.01654743 | 0.63063569 | 0.000999 |
| VR025 | VR024 | 0.01275133 | 0.37411877 | 0.35714286 |
| VR032 | J01263 | 0.01420193 | 0.57207903 | 0.0125 |
| VR032 | PV-at001 | 0.01078034 | 0.4809596 | 0.06802721 |
| VR032 | PV-ag003 | 0.01648668 | 0.50029784 | 0.02331002 |
| VR032 | PV-ag005 | 0.01717492 | 0.5631149 | 0.004995 |
| VR032 | Pv-ctt002 | 0.01383151 | 0.60167564 | 0.0625 |
| VR032 | SSR-IAC 127 | 0.00902676 | 0.45889135 | 0.34482759 |
| VR032 | SSR-IAC 188 | 0.00973324 | 0.54636117 | 0.66666667 |
| VR032 | SSR-IAC 195 | 0.01102805 | 0.41507417 | 0.55555556 |
| VR032 | DQ469392 | 0.01232352 | 0.5527444 | 0.41666667 |
| VR032 | DQ469393 | 0.01107191 | 0.48097867 | 0.3125 |
| VR032 | PvM03 | 0.01096193 | 0.62046903 | 0.26315789 |
| VR032 | PvM22 | 0.01019444 | 0.44007265 | 0.26315789 |
| VR032 | BMD-5 | 0.01956029 | 0.52488424 | 0.02557545 |
| VR032 | BMD-6 | 0.02007931 | 0.41621313 | 0.00799201 |
| VR032 | BMD-8 | 0.01595197 | 0.49424944 | 0 |
| VR032 | BMD-13 | 0.01372625 | 0.61101472 | 0.001998 |
| VR032 | BMD-18 | 0.01474054 | 0.57444428 | 0.004995 |
| VR032 | BMD-23 | 0.01127747 | 0.45921917 | 0.32258065 |
| VR032 | BMD-26 | 0.01068318 | 0.54657648 | 0.14285714 |
| VR032 | BMD-29 | 0.01326882 | 0.46432078 | 0.06097561 |
| VR032 | BMD-31 | 0.01551834 | 0.49308898 | 0.004995 |
| VR032 | BMD-48 | 0.01197588 | 0.43863881 | 0.26315789 |
| VR032 | BMD-35 | 0.01477639 | 0.45841992 | 0.00799201 |
| VR032 | BMD-47 | 0.0103811 | 0.517627 | 0.71428571 |
| VR032 | BMD-50 | 0.01119351 | 0.40392523 | 0.28571429 |
| VR032 | BMD-51 | 0.01015809 | 0.46008062 | 0.45454545 |
| VR032 | BMD-12 | 0.01263057 | 0.46501922 | 0.19607843 |
| VR032 | X21 | 0.01078046 | 0.48656684 | 0.52631579 |
| VR032 | X 34 | 0.00878271 | 0.48076794 | 0.90909091 |
| VR032 | X40 | 0.01356877 | 0.46231764 | 0.17857143 |
| VR032 | X49 | 0.01090834 | 0.44332139 | 0.11764706 |
| VR032 | X62 | 0.01326929 | 0.47631926 | 0.03875969 |
| VR032 | X65 | 0.01484768 | 0.48704466 | 0.00799201 |
| VR032 | X87 | 0.01039239 | 0.60064173 | 0.52631579 |
| VR032 | VR011 | 0.01136949 | 0.52896664 | 0.25 |
| VR032 | VR013 | 0.01252846 | 0.48835326 | 0.16949153 |
| VR032 | VR015 | 0.0122402 | 0.41622877 | 0.2 |
| VR032 | VR016 | 0.01093671 | 0.5226975 | 0.16666667 |
| VR032 | VR018 | 0.01286054 | 0.58436309 | 0.16666667 |
| VR032 | VR022 | 0.01648157 | 0.53278123 | 0.002997 |
| VR032 | VR022 | 0.01513303 | 0.60856264 | 0.0166113 |
| VR032 | VR024 | 0.00906765 | 0.35496698 | 0.83333333 |
| VR032 | VR025 | 0.01812202 | 0.51482291 | 0.000999 |
| VR033 | J01263 | 0.01555542 | 0.62896989 | 0 |
| VR033 | PV-at001 | 0.01612485 | 0.52422508 | 0.000999 |
| VR033 | PV-ag003 | 0.01586242 | 0.50012101 | 0.00699301 |
| VR033 | PV-ag005 | 0.0181648 | 0.56727923 | 0 |
| VR033 | Pv-ctt002 | 0.02595066 | 0.74187747 | 0 |
| VR033 | SSR-IAC 127 | 0.01176729 | 0.49142639 | 0.17857143 |
| VR033 | SSR-IAC 188 | 0.01520402 | 0.63445393 | 0.001998 |
| VR033 | SSR-IAC 195 | 0.01832435 | 0.4804977 | 0.002997 |
| VR033 | DQ469392 | 0.02079768 | 0.64749462 | 0 |
| VR033 | DQ469393 | 0.01446095 | 0.50219868 | 0.02415459 |
| VR033 | PvM03 | 0.01273381 | 0.6144991 | 0.03236246 |
| VR033 | PvM22 | 0.01424517 | 0.44708771 | 0.33333333 |
| VR033 | BMD-5 | 0.02005888 | 0.53587069 | 0 |
| VR033 | BMD-6 | 0.02761191 | 0.47735788 | 0 |
| VR033 | BMD-8 | 0.02000098 | 0.52334599 | 0.001998 |
| VR033 | BMD-13 | 0.01142385 | 0.61607794 | 0.35714286 |
| VR033 | BMD-18 | 0.01707047 | 0.5778388 | 0.000999 |
| VR033 | BMD-23 | 0.01777052 | 0.52880052 | 0 |
| VR033 | BMD-26 | 0.01357893 | 0.58285959 | 0.05376344 |
| VR033 | BMD-29 | 0.01396189 | 0.55382632 | 0.04672897 |
| VR033 | BMD-31 | 0.01717736 | 0.50995178 | 0.00699301 |
| VR033 | BMD-48 | 0.01363426 | 0.45388868 | 0.01517451 |
| VR033 | BMD-35 | 0.01575172 | 0.47824011 | 0.01766784 |
| VR033 | BMD-47 | 0.01390931 | 0.5603596 | 0.01270648 |
| VR033 | BMD-50 | 0.02829135 | 0.54038667 | 0 |
| VR033 | BMD-51 | 0.02380655 | 0.54497612 | 0 |
| VR033 | BMD-12 | 0.01655337 | 0.48257062 | 0.01132503 |
| VR033 | X21 | 0.02664492 | 0.56941466 | 0 |
| VR033 | X 34 | 0.01125621 | 0.52500206 | 0.05586592 |
| VR033 | X40 | 0.01933092 | 0.49377574 | 0.01631321 |
| VR033 | X49 | 0.01428658 | 0.46155092 | 0.00899101 |
| VR033 | X62 | 0.0138121 | 0.47023121 | 0.05405405 |
| VR033 | X65 | 0.01322046 | 0.46954896 | 0.12820513 |
| VR033 | X87 | 0.01255259 | 0.63420222 | 0.01592357 |
| VR033 | VR011 | 0.0185149 | 0.60436372 | 0.000999 |
| VR033 | VR013 | 0.00779818 | 0.42245345 | 0.76923077 |
| VR033 | VR015 | 0.01259364 | 0.40748265 | 0.16129032 |
| VR033 | VR016 | 0.01207672 | 0.5374247 | 0.10416667 |
| VR033 | VR018 | 0.01366711 | 0.62127549 | 0.03546099 |
| VR033 | VR022 | 0.01370075 | 0.52087372 | 0.07407407 |
| VR033 | VR022 | 0.01612358 | 0.65670694 | 0 |
| VR033 | VR024 | 0.0212055 | 0.47442056 | 0.000999 |
| VR033 | VR025 | 0.01928494 | 0.48229294 | 0 |
| VR033 | VR032 | 0.00891389 | 0.41321617 | 0.55555556 |
| VR035 | J01263 | 0.01434369 | 0.54946892 | 0.0390625 |
| VR035 | PV-at001 | 0.01369669 | 0.46825028 | 0.00899101 |
| VR035 | PV-ag003 | 0.01164789 | 0.43170022 | 0.66666667 |
| VR035 | PV-ag005 | 0.01558232 | 0.54804468 | 0.003996 |
| VR035 | Pv-ctt002 | 0.01267464 | 0.57966846 | 0.02457002 |
| VR035 | SSR-IAC 127 | 0.01270433 | 0.48521545 | 0.1369863 |
| VR035 | SSR-IAC 188 | 0.01252986 | 0.59691853 | 0.07575758 |
| VR035 | SSR-IAC 195 | 0.01566481 | 0.47312144 | 0.02762431 |
| VR035 | DQ469392 | 0.01035637 | 0.52326227 | 0.38461538 |
| VR035 | DQ469393 | 0.0148199 | 0.50779706 | 0.00599401 |
| VR035 | PvM03 | 0.01174902 | 0.60752553 | 0.04032258 |
| VR035 | PvM22 | 0.00814194 | 0.38176326 | 0.90909091 |
| VR035 | BMD-5 | 0.01060665 | 0.4445517 | 0.21276596 |
| VR035 | BMD-6 | 0.00837415 | 0.29912766 | 0.47619048 |
| VR035 | BMD-8 | 0.0154506 | 0.51502052 | 0.000999 |
| VR035 | BMD-13 | 0.01359224 | 0.65069273 | 0.0120919 |
| VR035 | BMD-18 | 0.01081021 | 0.51068294 | 0.27027027 |
| VR035 | BMD-23 | 0.0155205 | 0.48393171 | 0.0131406 |
| VR035 | BMD-26 | 0.01358225 | 0.58138944 | 0.0148368 |
| VR035 | BMD-29 | 0.01354969 | 0.48689083 | 0.09259259 |
| VR035 | BMD-31 | 0.01815783 | 0.52382988 | 0.001998 |
| VR035 | BMD-48 | 0.0135088 | 0.43395968 | 0.01453488 |
| VR035 | BMD-35 | 0.01209339 | 0.42260141 | 0.12195122 |
| VR035 | BMD-47 | 0.01532875 | 0.5696636 | 0.01270648 |
| VR035 | BMD-50 | 0.0158266 | 0.43259063 | 0 |
| VR035 | BMD-51 | 0.01193616 | 0.48820273 | 0.04878049 |
| VR035 | BMD-12 | 0.01483517 | 0.48112055 | 0.01331558 |
| VR035 | X21 | 0.0117323 | 0.49605183 | 0.0297619 |
| VR035 | X 34 | 0.00984991 | 0.49950339 | 0.66666667 |
| VR035 | X40 | 0.01429272 | 0.46498613 | 0.06993007 |
| VR035 | X49 | 0.01121487 | 0.44678696 | 0.20408163 |
| VR035 | X62 | 0.01060954 | 0.44713796 | 0.21276596 |
| VR035 | X65 | 0.00787603 | 0.39794936 | 0.45454545 |
| VR035 | X87 | 0.01377398 | 0.6406857 | 0.0243309 |
| VR035 | VR011 | 0.0132781 | 0.55778703 | 0.01538462 |
| VR035 | VR013 | 0.0091 | 0.44586902 | 0.5 |
| VR035 | VR015 | 0.01971922 | 0.45248516 | 0 |
| VR035 | VR016 | 0.0123289 | 0.54711576 | 0.00999001 |
| VR035 | VR018 | 0.01484831 | 0.60256321 | 0.002997 |
| VR035 | VR022 | 0.01513458 | 0.5163897 | 0.01242236 |
| VR035 | VR022 | 0.01773235 | 0.63040838 | 0 |
| VR035 | VR024 | 0.0145271 | 0.42671972 | 0.0990099 |
| VR035 | VR025 | 0.00911153 | 0.4083649 | 0.43478261 |
| VR035 | VR032 | 0.01084319 | 0.4225855 | 0.4 |
| VR035 | VR033 | 0.01672665 | 0.47296104 | 0.000999 |
| VR037 | J01263 | 0.01404504 | 0.67109012 | 0 |
| VR037 | PV-at001 | 0.01333529 | 0.59124482 | 0.00899101 |
| VR037 | PV-ag003 | 0.0106891 | 0.5135849 | 0.37037037 |
| VR037 | PV-ag005 | 0.01314572 | 0.61349159 | 0.00599401 |
| VR037 | Pv-ctt002 | 0.01021698 | 0.65166815 | 0.11627907 |
| VR037 | SSR-IAC 127 | 0.01123673 | 0.56591267 | 0.08695652 |
| VR037 | SSR-IAC 188 | 0.01106953 | 0.65482053 | 0.26315789 |
| VR037 | SSR-IAC 195 | 0.01927863 | 0.58672187 | 0 |
| VR037 | DQ469392 | 0.01451537 | 0.68721491 | 0.003996 |
| VR037 | DQ469393 | 0.01739017 | 0.63876279 | 0 |
| VR037 | PvM03 | 0.01307507 | 0.70000896 | 0.00899101 |
| VR037 | PvM22 | 0.01408838 | 0.56103018 | 0.1010101 |
| VR037 | BMD-5 | 0.01685157 | 0.58337896 | 0.002997 |
| VR037 | BMD-6 | 0.02116486 | 0.49545155 | 0.00599401 |
| VR037 | BMD-8 | 0.01463788 | 0.56014614 | 0 |
| VR037 | BMD-13 | 0.01333061 | 0.72087806 | 0.00599401 |
| VR037 | BMD-18 | 0.01610934 | 0.66206849 | 0 |
| VR037 | BMD-23 | 0.01695429 | 0.6098781 | 0 |
| VR037 | BMD-26 | 0.0109823 | 0.62687144 | 0.07194245 |
| VR037 | BMD-29 | 0.02267953 | 0.71936603 | 0 |
| VR037 | BMD-31 | 0.01390221 | 0.58434559 | 0 |
| VR037 | BMD-48 | 0.01214095 | 0.51776967 | 0.2173913 |
| VR037 | BMD-35 | 0.01615015 | 0.56852811 | 0 |
| VR037 | BMD-47 | 0.01893714 | 0.70214142 | 0 |
| VR037 | BMD-50 | 0.01719188 | 0.54164315 | 0 |
| VR037 | BMD-51 | 0.01480798 | 0.61416372 | 0.003996 |
| VR037 | BMD-12 | 0.01084367 | 0.48189033 | 0.83333333 |
| VR037 | X21 | 0.01923921 | 0.65925104 | 0 |
| VR037 | X 34 | 0.01327052 | 0.61789571 | 0.004995 |
| VR037 | X40 | 0.01237438 | 0.53613546 | 0.06849315 |
| VR037 | X49 | 0.01042591 | 0.4859024 | 0.38461538 |
| VR037 | X62 | 0.01574187 | 0.58036388 | 0.002997 |
| VR037 | X65 | 0.01341079 | 0.56602322 | 0.0166113 |
| VR037 | X87 | 0.01264754 | 0.70375079 | 0.00899101 |
| VR037 | VR011 | 0.01397196 | 0.66301676 | 0.01416431 |
| VR037 | VR013 | 0.00925148 | 0.52487858 | 0.76923077 |
| VR037 | VR015 | 0.00960068 | 0.44256161 | 0.76923077 |
| VR037 | VR016 | 0.01762468 | 0.64913365 | 0 |
| VR037 | VR018 | 0.0116252 | 0.66172572 | 0.08264463 |
| VR037 | VR022 | 0.0171801 | 0.61282117 | 0 |
| VR037 | VR022 | 0.01439044 | 0.68853902 | 0.003996 |
| VR037 | VR024 | 0.01311207 | 0.46686167 | 0.2173913 |
| VR037 | VR025 | 0.01228987 | 0.51895369 | 0.000999 |
| VR037 | VR032 | 0.01411445 | 0.55533028 | 0.01355014 |
| VR037 | VR033 | 0.01140672 | 0.53007786 | 0.06666667 |
| VR037 | VR035 | 0.01245376 | 0.5332602 | 0.07092199 |
| VR039 | J01263 | 0.0182429 | 0.67746465 | 0 |
| VR039 | PV-at001 | 0.01557931 | 0.56877886 | 0 |
| VR039 | PV-ag003 | 0.01828102 | 0.5661396 | 0.000999 |
| VR039 | PV-ag005 | 0.01504215 | 0.59380618 | 0.01004016 |
| VR039 | Pv-ctt002 | 0.01829329 | 0.71394523 | 0 |
| VR039 | SSR-IAC 127 | 0.01273859 | 0.55224711 | 0.01256281 |
| VR039 | SSR-IAC 188 | 0.01191121 | 0.63400243 | 0.09433962 |
| VR039 | SSR-IAC 195 | 0.01910818 | 0.52236307 | 0.000999 |
| VR039 | DQ469392 | 0.01481522 | 0.6305079 | 0.02053388 |
| VR039 | DQ469393 | 0.01031619 | 0.48991047 | 0.83333333 |
| VR039 | PvM03 | 0.01248391 | 0.67647826 | 0.01455604 |
| VR039 | PvM22 | 0.00799135 | 0.42561842 | 0.76923077 |
| VR039 | BMD-5 | 0.01640571 | 0.53159362 | 0.004995 |
| VR039 | BMD-6 | 0.0120849 | 0.38961577 | 0.0877193 |
| VR039 | BMD-8 | 0.01804114 | 0.54675096 | 0.000999 |
| VR039 | BMD-13 | 0.01194969 | 0.6594703 | 0.09433962 |
| VR039 | BMD-18 | 0.01600964 | 0.62545154 | 0 |
| VR039 | BMD-23 | 0.01185104 | 0.53328949 | 0.125 |
| VR039 | BMD-26 | 0.0136891 | 0.61613067 | 0.001998 |
| VR039 | BMD-29 | 0.01957408 | 0.61476774 | 0.000999 |
| VR039 | BMD-31 | 0.02067374 | 0.61090494 | 0 |
| VR039 | BMD-48 | 0.01151174 | 0.46780679 | 0.14084507 |
| VR039 | BMD-35 | 0.01599193 | 0.53504339 | 0 |
| VR039 | BMD-47 | 0.01005558 | 0.54898958 | 0.66666667 |
| VR039 | BMD-50 | 0.02415497 | 0.52062462 | 0.000999 |
| VR039 | BMD-51 | 0.02552067 | 0.58238581 | 0 |
| VR039 | BMD-12 | 0.01441496 | 0.52198276 | 0.00799201 |
| VR039 | X21 | 0.02690528 | 0.64282551 | 0 |
| VR039 | X 34 | 0.01091258 | 0.5728723 | 0.1 |
| VR039 | X40 | 0.01819456 | 0.54695927 | 0.000999 |
| VR039 | X49 | 0.01324851 | 0.51917925 | 0.000999 |
| VR039 | X62 | 0.01209948 | 0.52886312 | 0.03533569 |
| VR039 | X65 | 0.0094508 | 0.47632138 | 0.71428571 |
| VR039 | X87 | 0.0119983 | 0.68016986 | 0.05405405 |
| VR039 | VR011 | 0.01311239 | 0.62919442 | 0.01508296 |
| VR039 | VR013 | 0.00866242 | 0.48107013 | 0.90909091 |
| VR039 | VR015 | 0.01116998 | 0.42312158 | 0.41666667 |
| VR039 | VR016 | 0.0141029 | 0.60563587 | 0.02242152 |
| VR039 | VR018 | 0.01675227 | 0.68326556 | 0.000999 |
| VR039 | VR022 | 0.01356426 | 0.53760324 | 0 |
| VR039 | VR022 | 0.01446705 | 0.64695336 | 0.001998 |
| VR039 | VR024 | 0.02529949 | 0.52271896 | 0 |
| VR039 | VR025 | 0.03040938 | 0.60031375 | 0 |
| VR039 | VR032 | 0.0187025 | 0.56981762 | 0.000999 |
| VR039 | VR033 | 0.02382301 | 0.56563319 | 0 |
| VR039 | VR035 | 0.02112468 | 0.5961159 | 0 |
| VR039 | VR037 | 0.01354119 | 0.60030558 | 0.001998 |
| VR048 | J01263 | 0.01666483 | 0.61306727 | 0.001998 |
| VR048 | PV-at001 | 0.01496671 | 0.49627664 | 0 |
| VR048 | PV-ag003 | 0.01493624 | 0.47910636 | 0.03546099 |
| VR048 | PV-ag005 | 0.01654565 | 0.56322043 | 0.000999 |
| VR048 | Pv-ctt002 | 0.01428757 | 0.61209017 | 0 |
| VR048 | SSR-IAC 127 | 0.0117712 | 0.47324251 | 0.05235602 |
| VR048 | SSR-IAC 188 | 0.01630549 | 0.63650818 | 0 |
| VR048 | SSR-IAC 195 | 0.02159463 | 0.5183969 | 0 |
| VR048 | DQ469392 | 0.01376676 | 0.55178413 | 0.05586592 |
| VR048 | DQ469393 | 0.01905455 | 0.54092032 | 0 |
| VR048 | PvM03 | 0.0119568 | 0.60135478 | 0.06622517 |
| VR048 | PvM22 | 0.01128471 | 0.41170266 | 0.71428571 |
| VR048 | BMD-5 | 0.02180608 | 0.52492099 | 0 |
| VR048 | BMD-6 | 0.01385392 | 0.36704579 | 0.1010101 |
| VR048 | BMD-8 | 0.01949434 | 0.52636349 | 0 |
| VR048 | BMD-13 | 0.0145924 | 0.63003488 | 0.000999 |
| VR048 | BMD-18 | 0.02665403 | 0.6334908 | 0 |
| VR048 | BMD-23 | 0.013597 | 0.47968604 | 0.02673797 |
| VR048 | BMD-26 | 0.02198103 | 0.61673619 | 0.000999 |
| VR048 | BMD-29 | 0.01388076 | 0.48194771 | 0.06535948 |
| VR048 | BMD-31 | 0.0136504 | 0.49838281 | 0 |
| VR048 | BMD-48 | 0.01446626 | 0.45005389 | 0.001998 |
| VR048 | BMD-35 | 0.02106606 | 0.50370702 | 0 |
| VR048 | BMD-47 | 0.01279876 | 0.5423805 | 0.05494505 |
| VR048 | BMD-50 | 0.01985088 | 0.47730853 | 0 |
| VR048 | BMD-51 | 0.01598286 | 0.52644195 | 0.001998 |
| VR048 | BMD-12 | 0.01592314 | 0.46288422 | 0.02538071 |
| VR048 | X21 | 0.01233135 | 0.46487893 | 0.09708738 |
| VR048 | X 34 | 0.01381379 | 0.51482727 | 0.01642036 |
| VR048 | X40 | 0.01800303 | 0.51678423 | 0.003996 |
| VR048 | X49 | 0.03210718 | 0.5927042 | 0 |
| VR048 | X62 | 0.01768062 | 0.53140341 | 0 |
| VR048 | X65 | 0.01383329 | 0.46336296 | 0.01757469 |
| VR048 | X87 | 0.0107935 | 0.59628269 | 0.15625 |
| VR048 | VR011 | 0.01193215 | 0.54433029 | 0.15384615 |
| VR048 | VR013 | 0.01273343 | 0.47563348 | 0.3030303 |
| VR048 | VR015 | 0.01626081 | 0.43551193 | 0.003996 |
| VR048 | VR016 | 0.00953876 | 0.47371202 | 0.3125 |
| VR048 | VR018 | 0.01148655 | 0.55855413 | 0.08196721 |
| VR048 | VR022 | 0.01597593 | 0.51842939 | 0 |
| VR048 | VR022 | 0.01784184 | 0.66894395 | 0 |
| VR048 | VR024 | 0.01458022 | 0.43333872 | 0.02832861 |
| VR048 | VR025 | 0.01553831 | 0.46905504 | 0 |
| VR048 | VR032 | 0.00989584 | 0.3979413 | 0.52631579 |
| VR048 | VR033 | 0.01773336 | 0.5116845 | 0.002997 |
| VR048 | VR035 | 0.01294727 | 0.43592329 | 0.05813953 |
| VR048 | VR037 | 0.01730796 | 0.5841839 | 0 |
| VR048 | VR039 | 0.01516776 | 0.52400341 | 0.0147929 |
| DQ345305 | J01263 | 0.01410537 | 0.66632918 | 0.01879699 |
| DQ345305 | PV-at001 | 0.01505549 | 0.57654746 | 0.05882353 |
| DQ345305 | PV-ag003 | 0.011351 | 0.54292904 | 0.03731343 |
| DQ345305 | PV-ag005 | 0.01663605 | 0.63691352 | 0 |
| DQ345305 | Pv-ctt002 | 0.01435432 | 0.69654611 | 0.01644737 |
| DQ345305 | SSR-IAC 127 | 0.01070133 | 0.5625104 | 0.16949153 |
| DQ345305 | SSR-IAC 188 | 0.01186707 | 0.66845053 | 0.08 |
| DQ345305 | SSR-IAC 195 | 0.01848732 | 0.55562392 | 0.00699301 |
| DQ345305 | DQ469392 | 0.01586334 | 0.66626186 | 0 |
| DQ345305 | DQ469393 | 0.01327798 | 0.55994982 | 0.01941748 |
| DQ345305 | PvM03 | 0.01449609 | 0.7257725 | 0 |
| DQ345305 | PvM22 | 0.01114975 | 0.50437655 | 0.25 |
| DQ345305 | BMD-5 | 0.01574073 | 0.58075881 | 0 |
| DQ345305 | BMD-6 | 0.01212405 | 0.45325353 | 0.001998 |
| DQ345305 | BMD-8 | 0.01410797 | 0.56841823 | 0.05263158 |
| DQ345305 | BMD-13 | 0.01207559 | 0.67141614 | 0.05128205 |
| DQ345305 | BMD-18 | 0.01373453 | 0.63554139 | 0.00799201 |
| DQ345305 | BMD-23 | 0.0133864 | 0.54732757 | 0.15151515 |
| DQ345305 | BMD-26 | 0.01063385 | 0.61074432 | 0.625 |
| DQ345305 | BMD-29 | 0.01315543 | 0.56383045 | 0.02109705 |
| DQ345305 | BMD-31 | 0.01451365 | 0.56156506 | 0.05025126 |
| DQ345305 | BMD-48 | 0.01492688 | 0.53196627 | 0.28571429 |
| DQ345305 | BMD-35 | 0.01235594 | 0.49777107 | 0.20833333 |
| DQ345305 | BMD-47 | 0.01169401 | 0.57766176 | 0.08264463 |
| DQ345305 | BMD-50 | 0.01343565 | 0.50740883 | 0.05128205 |
| DQ345305 | BMD-51 | 0.01283886 | 0.555956 | 0.003996 |
| DQ345305 | BMD-12 | 0.01308343 | 0.52158579 | 0.22222222 |
| DQ345305 | X21 | 0.0123823 | 0.59160807 | 0.0877193 |
| DQ345305 | X 34 | 0.00950209 | 0.56759609 | 0.47619048 |
| DQ345305 | X40 | 0.01730631 | 0.57708167 | 0.000999 |
| DQ345305 | X49 | 0.01706844 | 0.57242829 | 0.00699301 |
| DQ345305 | X62 | 0.00924832 | 0.50188555 | 0.83333333 |
| DQ345305 | X65 | 0.01382489 | 0.57812138 | 0.0591716 |
| DQ345305 | X87 | 0.01148715 | 0.67523494 | 0.18181818 |
| DQ345305 | VR011 | 0.0141548 | 0.64320144 | 0.01371742 |
| DQ345305 | VR013 | 0.01029648 | 0.53765667 | 0.26315789 |
| DQ345305 | VR015 | 0.01579318 | 0.53508063 | 0.002997 |
| DQ345305 | VR016 | 0.01014171 | 0.58601521 | 0.58823529 |
| DQ345305 | VR018 | 0.01410738 | 0.66712436 | 0.000999 |
| DQ345305 | VR022 | 0.01692011 | 0.60918352 | 0 |
| DQ345305 | VR022 | 0.01332692 | 0.66664963 | 0.04926108 |
| DQ345305 | VR024 | 0.0082731 | 0.41136558 | 0.83333333 |
| DQ345305 | VR025 | 0.01164176 | 0.52133241 | 0.20833333 |
| DQ345305 | VR032 | 0.01210412 | 0.51607763 | 0.47619048 |
| DQ345305 | VR033 | 0.01385004 | 0.56952202 | 0.00799201 |
| DQ345305 | VR035 | 0.015077 | 0.56466915 | 0.004995 |
| DQ345305 | VR037 | 0.01334197 | 0.63129528 | 0.10989011 |
| DQ345305 | VR039 | 0.01446019 | 0.60654664 | 0.00599401 |
| DQ345305 | VR048 | 0.01869384 | 0.58026231 | 0 |
| CEDG036 | J01263 | 0.01216436 | 0.54238301 | 0.0280112 |
| CEDG036 | PV-at001 | 0.00810647 | 0.38922553 | 0.76923077 |
| CEDG036 | PV-ag003 | 0.00974325 | 0.38539763 | 0.58823529 |
| CEDG036 | PV-ag005 | 0.01171341 | 0.46520545 | 0.27027027 |
| CEDG036 | Pv-ctt002 | 0.01340103 | 0.57632407 | 0.07692308 |
| CEDG036 | SSR-IAC 127 | 0.01283454 | 0.46670237 | 0.10309278 |
| CEDG036 | SSR-IAC 188 | 0.0151877 | 0.58968358 | 0.002997 |
| CEDG036 | SSR-IAC 195 | 0.01184675 | 0.36866075 | 0.58823529 |
| CEDG036 | DQ469392 | 0.01203812 | 0.52083722 | 0.15873016 |
| CEDG036 | DQ469393 | 0.01365542 | 0.48838124 | 0.07874016 |
| CEDG036 | PvM03 | 0.01113075 | 0.55667496 | 0.34482759 |
| CEDG036 | PvM22 | 0.01336738 | 0.41788353 | 0.08403361 |
| CEDG036 | BMD-5 | 0.01578145 | 0.44048427 | 0.05434783 |
| CEDG036 | BMD-6 | 0.01149181 | 0.3098292 | 0.25 |
| CEDG036 | BMD-8 | 0.01610009 | 0.45918228 | 0.05181347 |
| CEDG036 | BMD-13 | 0.00973701 | 0.54231683 | 0.23255814 |
| CEDG036 | BMD-18 | 0.01381573 | 0.53389245 | 0.001998 |
| CEDG036 | BMD-23 | 0.01072261 | 0.44071237 | 0.1369863 |
| CEDG036 | BMD-26 | 0.01062705 | 0.49782919 | 0.32258065 |
| CEDG036 | BMD-29 | 0.01314205 | 0.49300809 | 0.00899101 |
| CEDG036 | BMD-31 | 0.01499067 | 0.47251357 | 0.04081633 |
| CEDG036 | BMD-48 | 0.00809785 | 0.34855336 | 0.83333333 |
| CEDG036 | BMD-35 | 0.01506571 | 0.42554905 | 0.00799201 |
| CEDG036 | BMD-47 | 0.01331638 | 0.5298277 | 0.05714286 |
| CEDG036 | BMD-50 | 0.01941317 | 0.43344942 | 0.001998 |
| CEDG036 | BMD-51 | 0.01620278 | 0.48544969 | 0.00599401 |
| CEDG036 | BMD-12 | 0.01539203 | 0.490888 | 0.00899101 |
| CEDG036 | X21 | 0.01179441 | 0.4863739 | 0.06060606 |
| CEDG036 | X 34 | 0.0134814 | 0.50587333 | 0.27777778 |
| CEDG036 | X40 | 0.01349556 | 0.45111932 | 0.00699301 |
| CEDG036 | X49 | 0.01290382 | 0.44762571 | 0.16129032 |
| CEDG036 | X62 | 0.01614278 | 0.46932684 | 0.04878049 |
| CEDG036 | X65 | 0.01546722 | 0.45309539 | 0.11764706 |
| CEDG036 | X87 | 0.01009763 | 0.55746096 | 0.41666667 |
| CEDG036 | VR011 | 0.01033284 | 0.49660492 | 0.76923077 |
| CEDG036 | VR013 | 0.01194466 | 0.43939111 | 0.10752688 |
| CEDG036 | VR015 | 0.01035118 | 0.35063261 | 0.58823529 |
| CEDG036 | VR016 | 0.01235519 | 0.49915398 | 0.0286533 |
| CEDG036 | VR018 | 0.01414721 | 0.57446771 | 0.03891051 |
| CEDG036 | VR022 | 0.0132612 | 0.46097741 | 0.01855288 |
| CEDG036 | VR022 | 0.01309531 | 0.58101916 | 0.05882353 |
| CEDG036 | VR024 | 0.01709286 | 0.42150722 | 0.01166861 |
| CEDG036 | VR025 | 0.01131764 | 0.40935622 | 0.2173913 |
| CEDG036 | VR032 | 0.0126807 | 0.43685052 | 0.10204082 |
| CEDG036 | VR033 | 0.01145771 | 0.41699413 | 0.21276596 |
| CEDG036 | VR035 | 0.00964009 | 0.3735773 | 0.45454545 |
| CEDG036 | VR037 | 0.01356017 | 0.5000457 | 0.11627907 |
| CEDG036 | VR039 | 0.00823771 | 0.40182343 | 0.32258065 |
| CEDG036 | VR048 | 0.01349196 | 0.44549995 | 0.02531646 |
| CEDG036 | DQ345305 | 0.01221005 | 0.49812071 | 0.06756757 |
| CEDG291 | J01263 | 0.01362323 | 0.67636515 | 0.00599401 |
| CEDG291 | PV-at001 | 0.0140309 | 0.62105622 | 0.03095975 |
| CEDG291 | PV-ag003 | 0.01246432 | 0.59290942 | 0.08064516 |
| CEDG291 | PV-ag005 | 0.01376618 | 0.63251083 | 0.24390244 |
| CEDG291 | Pv-ctt002 | 0.01222706 | 0.6783903 | 0.2173913 |
| CEDG291 | SSR-IAC 127 | 0.01022062 | 0.54947217 | 0.71428571 |
| CEDG291 | SSR-IAC 188 | 0.01078319 | 0.65740104 | 0.45454545 |
| CEDG291 | SSR-IAC 195 | 0.01194968 | 0.52463429 | 0.05747126 |
| CEDG291 | DQ469392 | 0.01350441 | 0.63897096 | 0.32258065 |
| CEDG291 | DQ469393 | 0.01676966 | 0.64520021 | 0 |
| CEDG291 | PvM03 | 0.01270527 | 0.72291713 | 0.002997 |
| CEDG291 | PvM22 | 0.01287644 | 0.54796701 | 0.07751938 |
| CEDG291 | BMD-5 | 0.01656547 | 0.61854777 | 0 |
| CEDG291 | BMD-6 | 0.02141336 | 0.52614746 | 0 |
| CEDG291 | BMD-8 | 0.01684027 | 0.59275603 | 0.000999 |
| CEDG291 | BMD-13 | 0.01321917 | 0.72962984 | 0.000999 |
| CEDG291 | BMD-18 | 0.01171798 | 0.61709835 | 0.03690037 |
| CEDG291 | BMD-23 | 0.01719404 | 0.61671914 | 0.06578947 |
| CEDG291 | BMD-26 | 0.01050486 | 0.64361527 | 0.52631579 |
| CEDG291 | BMD-29 | 0.01065576 | 0.6015033 | 0.09345794 |
| CEDG291 | BMD-31 | 0.0156138 | 0.62155464 | 0.01557632 |
| CEDG291 | BMD-48 | 0.01166201 | 0.51621538 | 0.10526316 |
| CEDG291 | BMD-35 | 0.01633201 | 0.61378848 | 0 |
| CEDG291 | BMD-47 | 0.01350515 | 0.62306385 | 0.58823529 |
| CEDG291 | BMD-50 | 0.01071914 | 0.49815633 | 0.18518519 |
| CEDG291 | BMD-51 | 0.01265226 | 0.58909631 | 0.041841 |
| CEDG291 | BMD-12 | 0.00884852 | 0.50613416 | 0.76923077 |
| CEDG291 | X21 | 0.01042259 | 0.57616264 | 0.66666667 |
| CEDG291 | X 34 | 0.01004446 | 0.57505427 | 0.26315789 |
| CEDG291 | X40 | 0.01427625 | 0.58527695 | 0.000999 |
| CEDG291 | X49 | 0.01250146 | 0.57342677 | 0.004995 |
| CEDG291 | X62 | 0.00910426 | 0.52840559 | 0.55555556 |
| CEDG291 | X65 | 0.01250708 | 0.58704026 | 0.06329114 |
| CEDG291 | X87 | 0.01284361 | 0.73344319 | 0.01669449 |
| CEDG291 | VR011 | 0.01117914 | 0.64018366 | 0.83333333 |
| CEDG291 | VR013 | 0.01366375 | 0.634036 | 0.03558719 |
| CEDG291 | VR015 | 0.01459683 | 0.55775202 | 0.01730104 |
| CEDG291 | VR016 | 0.01350684 | 0.66171334 | 0 |
| CEDG291 | VR018 | 0.01562401 | 0.72207601 | 0 |
| CEDG291 | VR022 | 0.01370302 | 0.61509848 | 0.0140647 |
| CEDG291 | VR022 | 0.01329757 | 0.71724725 | 0.001998 |
| CEDG291 | VR024 | 0.00871399 | 0.46891931 | 0.43478261 |
| CEDG291 | VR025 | 0.01544674 | 0.60862819 | 0.002997 |
| CEDG291 | VR032 | 0.0111858 | 0.56013175 | 0.15625 |
| CEDG291 | VR033 | 0.01376092 | 0.57743943 | 0.16666667 |
| CEDG291 | VR035 | 0.01220741 | 0.53522415 | 0.52631579 |
| CEDG291 | VR037 | 0.01140076 | 0.63548033 | 0.25641026 |
| CEDG291 | VR039 | 0.01345073 | 0.59543835 | 0.01776199 |
| CEDG291 | VR048 | 0.01262788 | 0.55065184 | 0.01196172 |
| CEDG291 | DQ345305 | 0.01414935 | 0.63962862 | 0.02881844 |
| CEDG291 | CEDG036 | 0.02567925 | 0.62348986 | 0 |
| CEDG220 | J01263 | 0.01790579 | 0.65886565 | 0 |
| CEDG220 | PV-at001 | 0.01372361 | 0.53159333 | 0.04545455 |
| CEDG220 | PV-ag003 | 0.01676843 | 0.54890077 | 0.004995 |
| CEDG220 | PV-ag005 | 0.01515943 | 0.57356443 | 0.003996 |
| CEDG220 | Pv-ctt002 | 0.01350307 | 0.65151599 | 0.02053388 |
| CEDG220 | SSR-IAC 127 | 0.01191832 | 0.52038916 | 0.27027027 |
| CEDG220 | SSR-IAC 188 | 0.01388865 | 0.66304474 | 0.01644737 |
| CEDG220 | SSR-IAC 195 | 0.01539156 | 0.51536788 | 0.05291005 |
| CEDG220 | DQ469392 | 0.0163026 | 0.62964368 | 0.001998 |
| CEDG220 | DQ469393 | 0.01319854 | 0.53064837 | 0.01396648 |
| CEDG220 | PvM03 | 0.01315835 | 0.66744226 | 0.01044932 |
| CEDG220 | PvM22 | 0.01027094 | 0.46880986 | 0.5 |
| CEDG220 | BMD-5 | 0.01720977 | 0.54789995 | 0 |
| CEDG220 | BMD-6 | 0.01327981 | 0.41261688 | 0.12048193 |
| CEDG220 | BMD-8 | 0.01679162 | 0.57115754 | 0 |
| CEDG220 | BMD-13 | 0.01671968 | 0.72090932 | 0 |
| CEDG220 | BMD-18 | 0.02549189 | 0.69583061 | 0 |
| CEDG220 | BMD-23 | 0.01402114 | 0.54377983 | 0.0990099 |
| CEDG220 | BMD-26 | 0.01296302 | 0.62639292 | 0.00899101 |
| CEDG220 | BMD-29 | 0.01321468 | 0.5402467 | 0.02061856 |
| CEDG220 | BMD-31 | 0.01500948 | 0.54562179 | 0.01398601 |
| CEDG220 | BMD-48 | 0.02175254 | 0.54029785 | 0 |
| CEDG220 | BMD-35 | 0.01649642 | 0.51698623 | 0 |
| CEDG220 | BMD-47 | 0.01217414 | 0.57438843 | 0.05681818 |
| CEDG220 | BMD-50 | 0.01559255 | 0.50957065 | 0 |
| CEDG220 | BMD-51 | 0.01651433 | 0.54209627 | 0.01980198 |
| CEDG220 | BMD-12 | 0.0152481 | 0.51261906 | 0.05494505 |
| CEDG220 | X21 | 0.01619486 | 0.59589005 | 0 |
| CEDG220 | X 34 | 0.01322561 | 0.58540317 | 0.08928571 |
| CEDG220 | X40 | 0.01308631 | 0.47818092 | 0.03984064 |
| CEDG220 | X49 | 0.01341759 | 0.51593789 | 0.04273504 |
| CEDG220 | X62 | 0.01362908 | 0.53663253 | 0.01776199 |
| CEDG220 | X65 | 0.01224515 | 0.50630126 | 0.07407407 |
| CEDG220 | X87 | 0.01210816 | 0.68428979 | 0.08196721 |
| CEDG220 | VR011 | 0.01610862 | 0.64976835 | 0 |
| CEDG220 | VR013 | 0.01361299 | 0.5525634 | 0.03690037 |
| CEDG220 | VR015 | 0.01388733 | 0.46423448 | 0.03460208 |
| CEDG220 | VR016 | 0.01312485 | 0.59230818 | 0.02617801 |
| CEDG220 | VR018 | 0.01247904 | 0.6225159 | 0.07042254 |
| CEDG220 | VR022 | 0.01279533 | 0.55477267 | 0.04366812 |
| CEDG220 | VR022 | 0.01144743 | 0.62098337 | 0.12048193 |
| CEDG220 | VR024 | 0.00952308 | 0.41664834 | 0.83333333 |
| CEDG220 | VR025 | 0.02018012 | 0.56636039 | 0 |
| CEDG220 | VR032 | 0.00940407 | 0.44750499 | 0.76923077 |
| CEDG220 | VR033 | 0.01251393 | 0.4945498 | 0.0203666 |
| CEDG220 | VR035 | 0.01542703 | 0.54226627 | 0 |
| CEDG220 | VR037 | 0.01151766 | 0.56545044 | 0.07692308 |
| CEDG220 | VR039 | 0.01636455 | 0.5934519 | 0 |
| CEDG220 | VR048 | 0.01414034 | 0.49565884 | 0.02898551 |
| CEDG220 | DQ345305 | 0.01332058 | 0.57544936 | 0.04032258 |
| CEDG220 | CEDG036 | 0.0159854 | 0.478461 | 0.07518797 |
| CEDG220 | CEDG291 | 0.02086296 | 0.67793456 | 0 |
| CEDG136 | J01263 | 0.01056744 | 0.56677662 | 0.41666667 |
| CEDG136 | PV-at001 | 0.01309697 | 0.56205468 | 0.15873016 |
| CEDG136 | PV-ag003 | 0.0105248 | 0.48371029 | 0.0625 |
| CEDG136 | PV-ag005 | 0.01270536 | 0.56999101 | 0.12048193 |
| CEDG136 | Pv-ctt002 | 0.01218325 | 0.64422659 | 0.08849558 |
| CEDG136 | SSR-IAC 127 | 0.01094156 | 0.53081364 | 0.43478261 |
| CEDG136 | SSR-IAC 188 | 0.0135289 | 0.66696302 | 0.0105042 |
| CEDG136 | SSR-IAC 195 | 0.01480594 | 0.51253987 | 0.02673797 |
| CEDG136 | DQ469392 | 0.00948625 | 0.5739566 | 0.18181818 |
| CEDG136 | DQ469393 | 0.00872805 | 0.46682254 | 0.58823529 |
| CEDG136 | PvM03 | 0.01469644 | 0.70343984 | 0.001998 |
| CEDG136 | PvM22 | 0.01072235 | 0.48578941 | 0.15384615 |
| CEDG136 | BMD-5 | 0.01490029 | 0.52348925 | 0.12820513 |
| CEDG136 | BMD-6 | 0.00769388 | 0.35858757 | 0.43478261 |
| CEDG136 | BMD-8 | 0.01313616 | 0.51803373 | 0.12987013 |
| CEDG136 | BMD-13 | 0.01328776 | 0.66283103 | 0.01123596 |
| CEDG136 | BMD-18 | 0.01573855 | 0.62211336 | 0 |
| CEDG136 | BMD-23 | 0.00891 | 0.46998809 | 0.90909091 |
| CEDG136 | BMD-26 | 0.01131458 | 0.62168424 | 0.20408163 |
| CEDG136 | BMD-29 | 0.01379195 | 0.57802622 | 0 |
| CEDG136 | BMD-31 | 0.01811123 | 0.58455378 | 0.01644737 |
| CEDG136 | BMD-48 | 0.0147076 | 0.49306163 | 0.23255814 |
| CEDG136 | BMD-35 | 0.02127181 | 0.58162875 | 0 |
| CEDG136 | BMD-47 | 0.00864808 | 0.52815078 | 0.71428571 |
| CEDG136 | BMD-50 | 0.01681985 | 0.50644629 | 0.00899101 |
| CEDG136 | BMD-51 | 0.01598436 | 0.58031676 | 0.01865672 |
| CEDG136 | BMD-12 | 0.01143254 | 0.48655417 | 0.27777778 |
| CEDG136 | X21 | 0.01424533 | 0.56991255 | 0.06849315 |
| CEDG136 | X 34 | 0.01085718 | 0.56716714 | 0.29411765 |
| CEDG136 | X40 | 0.01195836 | 0.52371184 | 0.38461538 |
| CEDG136 | X49 | 0.01129996 | 0.49269064 | 0.19230769 |
| CEDG136 | X62 | 0.01343439 | 0.53532369 | 0.24390244 |
| CEDG136 | X65 | 0.00880805 | 0.46683596 | 0.35714286 |
| CEDG136 | X87 | 0.0117617 | 0.6619959 | 0.24390244 |
| CEDG136 | VR011 | 0.01350442 | 0.61268585 | 0.03021148 |
| CEDG136 | VR013 | 0.00924443 | 0.50683552 | 0.4 |
| CEDG136 | VR015 | 0.00944399 | 0.43102895 | 0.43478261 |
| CEDG136 | VR016 | 0.01139098 | 0.55299753 | 0.55555556 |
| CEDG136 | VR018 | 0.01102235 | 0.61425573 | 0.23809524 |
| CEDG136 | VR022 | 0.01132361 | 0.557974 | 0.09803922 |
| CEDG136 | VR022 | 0.01225146 | 0.6492112 | 0.07692308 |
| CEDG136 | VR024 | 0.00887396 | 0.40077414 | 0.90909091 |
| CEDG136 | VR025 | 0.01501481 | 0.53716585 | 0.04405286 |
| CEDG136 | VR032 | 0.01149431 | 0.49252431 | 0.19230769 |
| CEDG136 | VR033 | 0.00793887 | 0.40955998 | 0.76923077 |
| CEDG136 | VR035 | 0.0124187 | 0.51675493 | 0.07092199 |
| CEDG136 | VR037 | 0.01444163 | 0.5984744 | 0.07751938 |
| CEDG136 | VR039 | 0.01552091 | 0.6012403 | 0.002997 |
| CEDG136 | VR048 | 0.01190805 | 0.50421802 | 0.21276596 |
| CEDG136 | DQ345305 | 0.01246598 | 0.59343836 | 0.07042254 |
| CEDG136 | CEDG036 | 0.0142238 | 0.52301599 | 0.00599401 |
| CEDG136 | CEDG291 | 0.01577492 | 0.64895202 | 0.000999 |
| CEDG136 | CEDG220 | 0.01447993 | 0.59345856 | 0.03703704 |
| CEDG100 | J01263 | 0.01271307 | 0.68680345 | 0 |
| CEDG100 | PV-at001 | 0.01185027 | 0.61767866 | 0.14084507 |
| CEDG100 | PV-ag003 | 0.01338431 | 0.62999919 | 0.02415459 |
| CEDG100 | PV-ag005 | 0.01535112 | 0.68224422 | 0.003996 |
| CEDG100 | Pv-ctt002 | 0.01228606 | 0.72607056 | 0.04366812 |
| CEDG100 | SSR-IAC 127 | 0.00897525 | 0.57761869 | 0.625 |
| CEDG100 | SSR-IAC 188 | 0.01380896 | 0.73938151 | 0.000999 |
| CEDG100 | SSR-IAC 195 | 0.01046748 | 0.50561151 | 0.25 |
| CEDG100 | DQ469392 | 0.01176468 | 0.67851226 | 0.52631579 |
| CEDG100 | DQ469393 | 0.01530738 | 0.67594903 | 0.002997 |
| CEDG100 | PvM03 | 0.01238543 | 0.73488609 | 0.02583979 |
| CEDG100 | PvM22 | 0.01301834 | 0.59856874 | 0.01187648 |
| CEDG100 | BMD-5 | 0.02059228 | 0.65713183 | 0 |
| CEDG100 | BMD-6 | 0.01033527 | 0.44653883 | 0.27027027 |
| CEDG100 | BMD-8 | 0.01336581 | 0.61254282 | 0.05208333 |
| CEDG100 | BMD-13 | 0.01324545 | 0.76872572 | 0.004995 |
| CEDG100 | BMD-18 | 0.01472499 | 0.68088175 | 0 |
| CEDG100 | BMD-23 | 0.00998822 | 0.56016636 | 0.90909091 |
| CEDG100 | BMD-26 | 0.01077129 | 0.66194776 | 0.05649718 |
| CEDG100 | BMD-29 | 0.01323196 | 0.6284711 | 0.004995 |
| CEDG100 | BMD-31 | 0.01501339 | 0.63989495 | 0.000999 |
| CEDG100 | BMD-48 | 0.01290948 | 0.52657103 | 0.76923077 |
| CEDG100 | BMD-35 | 0.01694397 | 0.64465798 | 0.004995 |
| CEDG100 | BMD-47 | 0.01338319 | 0.67076674 | 0.18867925 |
| CEDG100 | BMD-50 | 0.01437812 | 0.57796139 | 0.07194245 |
| CEDG100 | BMD-51 | 0.01072425 | 0.59214106 | 0.38461538 |
| CEDG100 | BMD-12 | 0.0105263 | 0.57810474 | 0.13888889 |
| CEDG100 | X21 | 0.01272367 | 0.65460499 | 0.07142857 |
| CEDG100 | X 34 | 0.01310046 | 0.67625968 | 0.16666667 |
| CEDG100 | X40 | 0.01687293 | 0.62086126 | 0.000999 |
| CEDG100 | X49 | 0.01408614 | 0.61421144 | 0.004995 |
| CEDG100 | X62 | 0.01047135 | 0.56394407 | 0.76923077 |
| CEDG100 | X65 | 0.00986313 | 0.55608258 | 0.27777778 |
| CEDG100 | X87 | 0.01158567 | 0.73883243 | 0.03831418 |
| CEDG100 | VR011 | 0.01191394 | 0.67484541 | 0.07462687 |
| CEDG100 | VR013 | 0.01062147 | 0.60427991 | 0.15873016 |
| CEDG100 | VR015 | 0.00943847 | 0.52510839 | 0.3125 |
| CEDG100 | VR016 | 0.01252125 | 0.65746521 | 0.07194245 |
| CEDG100 | VR018 | 0.01569078 | 0.74269157 | 0 |
| CEDG100 | VR022 | 0.01154459 | 0.6157816 | 0.04830918 |
| CEDG100 | VR022 | 0.01315193 | 0.73403446 | 0.00699301 |
| CEDG100 | VR024 | 0.01192869 | 0.54863692 | 0.0862069 |
| CEDG100 | VR025 | 0.01577689 | 0.63330596 | 0.002997 |
| CEDG100 | VR032 | 0.0103116 | 0.55657034 | 0.5 |
| CEDG100 | VR033 | 0.01429377 | 0.64452786 | 0.01388889 |
| CEDG100 | VR035 | 0.0128768 | 0.57791135 | 0.34482759 |
| CEDG100 | VR037 | 0.01355313 | 0.68803109 | 0.01865672 |
| CEDG100 | VR039 | 0.01236371 | 0.63018504 | 0.12195122 |
| CEDG100 | VR048 | 0.01437361 | 0.60036561 | 0.11235955 |
| CEDG100 | DQ345305 | 0.01265022 | 0.69037553 | 0.06622517 |
| CEDG100 | CEDG036 | 0.02010523 | 0.64932893 | 0 |
| CEDG100 | CEDG291 | 0.0151589 | 0.70536101 | 0.000999 |
| CEDG100 | CEDG220 | 0.01563834 | 0.6769329 | 0.002997 |
| CEDG100 | CEDG136 | 0.01335993 | 0.64664824 | 0.00599401 |
| CEDG096A | J01263 | 0.01543444 | 0.76678468 | 0 |
| CEDG096A | PV-at001 | 0.01261896 | 0.65817281 | 0.0591716 |
| CEDG096A | PV-ag003 | 0.01414615 | 0.6620576 | 0.01003009 |
| CEDG096A | PV-ag005 | 0.01266261 | 0.6814051 | 0.05181347 |
| CEDG096A | Pv-ctt002 | 0.01192855 | 0.73984945 | 0.04132231 |
| CEDG096A | SSR-IAC 127 | 0.01122423 | 0.65223244 | 0.37037037 |
| CEDG096A | SSR-IAC 188 | 0.0102742 | 0.70533283 | 0.55555556 |
| CEDG096A | SSR-IAC 195 | 0.01350832 | 0.61895221 | 0.11235955 |
| CEDG096A | DQ469392 | 0.0112675 | 0.70046976 | 0.10526316 |
| CEDG096A | DQ469393 | 0.01203808 | 0.64946478 | 0.04739336 |
| CEDG096A | PvM03 | 0.01340589 | 0.78105787 | 0.002997 |
| CEDG096A | PvM22 | 0.0127588 | 0.61572416 | 0.002997 |
| CEDG096A | BMD-5 | 0.01566762 | 0.65088019 | 0.000999 |
| CEDG096A | BMD-6 | 0.01264575 | 0.54327295 | 0.05714286 |
| CEDG096A | BMD-8 | 0.01405847 | 0.66226758 | 0.01265823 |
| CEDG096A | BMD-13 | 0.01676895 | 0.81458162 | 0 |
| CEDG096A | BMD-18 | 0.01730987 | 0.71667366 | 0 |
| CEDG096A | BMD-23 | 0.00915149 | 0.58669848 | 0.55555556 |
| CEDG096A | BMD-26 | 0.01127701 | 0.69505228 | 0.23255814 |
| CEDG096A | BMD-29 | 0.01611851 | 0.69023745 | 0.000999 |
| CEDG096A | BMD-31 | 0.01624841 | 0.71269977 | 0 |
| CEDG096A | BMD-48 | 0.00936144 | 0.54701603 | 0.45454545 |
| CEDG096A | BMD-35 | 0.012009 | 0.609092 | 0.06410256 |
| CEDG096A | BMD-47 | 0.0109066 | 0.67470389 | 0.01615509 |
| CEDG096A | BMD-50 | 0.01549747 | 0.63996573 | 0.003996 |
| CEDG096A | BMD-51 | 0.01098179 | 0.63453122 | 0.2 |
| CEDG096A | BMD-12 | 0.01069848 | 0.58714313 | 0.45454545 |
| CEDG096A | X21 | 0.01068129 | 0.64587048 | 0.10309278 |
| CEDG096A | X 34 | 0.0110294 | 0.64928236 | 0.24390244 |
| CEDG096A | X40 | 0.00973352 | 0.58941178 | 0.41666667 |
| CEDG096A | X49 | 0.00982082 | 0.57551305 | 0.41666667 |
| CEDG096A | X62 | 0.01242402 | 0.65514797 | 0.08474576 |
| CEDG096A | X65 | 0.0098021 | 0.58073122 | 0.35714286 |
| CEDG096A | X87 | 0.01205075 | 0.77403859 | 0.17241379 |
| CEDG096A | VR011 | 0.01143024 | 0.71568958 | 0.15384615 |
| CEDG096A | VR013 | 0.00975261 | 0.61874365 | 0.37037037 |
| CEDG096A | VR015 | 0.01095568 | 0.57515024 | 0.27777778 |
| CEDG096A | VR016 | 0.01289032 | 0.7044374 | 0.00699301 |
| CEDG096A | VR018 | 0.01236136 | 0.73860994 | 0.01610306 |
| CEDG096A | VR022 | 0.00929377 | 0.60348473 | 0.35714286 |
| CEDG096A | VR022 | 0.01305216 | 0.76653808 | 0.001998 |
| CEDG096A | VR024 | 0.00893554 | 0.49967641 | 0.58823529 |
| CEDG096A | VR025 | 0.01304018 | 0.6347801 | 0.003996 |
| CEDG096A | VR032 | 0.01177585 | 0.61828465 | 0.10638298 |
| CEDG096A | VR033 | 0.01131527 | 0.60434376 | 0.14285714 |
| CEDG096A | VR035 | 0.01245195 | 0.61177492 | 0.07874016 |
| CEDG096A | VR037 | 0.01128312 | 0.68705827 | 0.1754386 |
| CEDG096A | VR039 | 0.01266188 | 0.67332615 | 0.01805054 |
| CEDG096A | VR048 | 0.01251575 | 0.60400023 | 0.11627907 |
| CEDG096A | DQ345305 | 0.01286755 | 0.71926706 | 0.02898551 |
| CEDG096A | CEDG036 | 0.01330513 | 0.60601702 | 0.01251564 |
| CEDG096A | CEDG291 | 0.01204251 | 0.71347287 | 0.01785714 |
| CEDG096A | CEDG220 | 0.0138965 | 0.68399664 | 0 |
| CEDG096A | CEDG136 | 0.01563841 | 0.71104917 | 0 |
| CEDG096A | CEDG100 | 0.01426496 | 0.77835939 | 0 |
| CP00361 | J01263 | 0.01751212 | 0.65794038 | 0 |
| CP00361 | PV-at001 | 0.00970936 | 0.48125782 | 0.28571429 |
| CP00361 | PV-ag003 | 0.01369082 | 0.51914589 | 0.14925373 |
| CP00361 | PV-ag005 | 0.01731301 | 0.5793168 | 0.01949318 |
| CP00361 | Pv-ctt002 | 0.01214501 | 0.61596613 | 0.05464481 |
| CP00361 | SSR-IAC 127 | 0.00947188 | 0.50539451 | 0.55555556 |
| CP00361 | SSR-IAC 188 | 0.01118542 | 0.61648347 | 0.22222222 |
| CP00361 | SSR-IAC 195 | 0.01026725 | 0.41206852 | 0.83333333 |
| CP00361 | DQ469392 | 0.01204748 | 0.59240824 | 0.11111111 |
| CP00361 | DQ469393 | 0.01515499 | 0.56912521 | 0.002997 |
| CP00361 | PvM03 | 0.01503149 | 0.71647978 | 0 |
| CP00361 | PvM22 | 0.01219247 | 0.4582181 | 0.58823529 |
| CP00361 | BMD-5 | 0.02361887 | 0.56869858 | 0 |
| CP00361 | BMD-6 | 0.02087898 | 0.44052961 | 0.07751938 |
| CP00361 | BMD-8 | 0.01169252 | 0.49691561 | 0.35714286 |
| CP00361 | BMD-13 | 0.01719081 | 0.72010386 | 0.004995 |
| CP00361 | BMD-18 | 0.01976057 | 0.66061831 | 0 |
| CP00361 | BMD-23 | 0.01254718 | 0.52068983 | 0.16393443 |
| CP00361 | BMD-26 | 0.00967568 | 0.57589843 | 0.4 |
| CP00361 | BMD-29 | 0.02070361 | 0.63230018 | 0 |
| CP00361 | BMD-31 | 0.01870804 | 0.56914715 | 0 |
| CP00361 | BMD-48 | 0.01087873 | 0.45694127 | 0.15625 |
| CP00361 | BMD-35 | 0.01402048 | 0.48504817 | 0.03690037 |
| CP00361 | BMD-47 | 0.01331667 | 0.58343712 | 0.0877193 |
| CP00361 | BMD-50 | 0.01525133 | 0.50593626 | 0 |
| CP00361 | BMD-51 | 0.01025827 | 0.48813758 | 0.34482759 |
| CP00361 | BMD-12 | 0.01450927 | 0.49386721 | 0.01136364 |
| CP00361 | X21 | 0.01308161 | 0.56649426 | 0.03816794 |
| CP00361 | X 34 | 0.01179363 | 0.56311773 | 0.32258065 |
| CP00361 | X40 | 0.01271643 | 0.48695466 | 0.10526316 |
| CP00361 | X49 | 0.01178677 | 0.507883 | 0.14492754 |
| CP00361 | X62 | 0.01218014 | 0.51870945 | 0.41666667 |
| CP00361 | X65 | 0.01039252 | 0.50445172 | 0.24390244 |
| CP00361 | X87 | 0.01587563 | 0.7128181 | 0.01618123 |
| CP00361 | VR011 | 0.01539856 | 0.61088766 | 0.03012048 |
| CP00361 | VR013 | 0.009722 | 0.50061332 | 0.3125 |
| CP00361 | VR015 | 0.01262085 | 0.47519951 | 0.11904762 |
| CP00361 | VR016 | 0.01426914 | 0.60413515 | 0.11904762 |
| CP00361 | VR018 | 0.01327691 | 0.64345223 | 0.003996 |
| CP00361 | VR022 | 0.01023026 | 0.51459512 | 0.16949153 |
| CP00361 | VR022 | 0.01097417 | 0.61499202 | 0.23255814 |
| CP00361 | VR024 | 0.00988 | 0.41020441 | 0.90909091 |
| CP00361 | VR025 | 0.01362342 | 0.50592681 | 0.1369863 |
| CP00361 | VR032 | 0.01223001 | 0.49884646 | 0.14705882 |
| CP00361 | VR033 | 0.01286225 | 0.49286326 | 0.1754386 |
| CP00361 | VR035 | 0.01073714 | 0.47138358 | 0.38461538 |
| CP00361 | VR037 | 0.01480157 | 0.60790857 | 0.01150748 |
| CP00361 | VR039 | 0.01232434 | 0.53404 | 0.14925373 |
| CP00361 | VR048 | 0.01421842 | 0.52430573 | 0.03164557 |
| CP00361 | DQ345305 | 0.01066837 | 0.54996301 | 0.2 |
| CP00361 | CEDG036 | 0.02010485 | 0.53919761 | 0 |
| CP00361 | CEDG291 | 0.01389055 | 0.63306598 | 0.000999 |
| CP00361 | CEDG220 | 0.02388478 | 0.65679099 | 0 |
| CP00361 | CEDG136 | 0.01805117 | 0.61982114 | 0 |
| CP00361 | CEDG100 | 0.01794078 | 0.69630104 | 0 |
| CP00361 | CEDG096A | 0.01765586 | 0.7278724 | 0 |
| DMBSSR035 | J01263 | 0.01338499 | 0.66323211 | 0.002997 |
| DMBSSR035 | PV-at001 | 0.01410107 | 0.61175687 | 0.01303781 |
| DMBSSR035 | PV-ag003 | 0.01176312 | 0.53169482 | 0.05 |
| DMBSSR035 | PV-ag005 | 0.01401226 | 0.61781913 | 0.000999 |
| DMBSSR035 | Pv-ctt002 | 0.01282765 | 0.70462252 | 0.001998 |
| DMBSSR035 | SSR-IAC 127 | 0.012838 | 0.59553611 | 0.07462687 |
| DMBSSR035 | SSR-IAC 188 | 0.01157906 | 0.66992627 | 0.02890173 |
| DMBSSR035 | SSR-IAC 195 | 0.02025018 | 0.57282627 | 0.001998 |
| DMBSSR035 | DQ469392 | 0.01496729 | 0.65526921 | 0.01156069 |
| DMBSSR035 | DQ469393 | 0.02081696 | 0.66010577 | 0 |
| DMBSSR035 | PvM03 | 0.01026299 | 0.66269996 | 0.26315789 |
| DMBSSR035 | PvM22 | 0.01705132 | 0.57882424 | 0.02178649 |
| DMBSSR035 | BMD-5 | 0.0160661 | 0.61181647 | 0.000999 |
| DMBSSR035 | BMD-6 | 0.00750545 | 0.3603229 | 0.71428571 |
| DMBSSR035 | BMD-8 | 0.02078007 | 0.64506833 | 0 |
| DMBSSR035 | BMD-13 | 0.01625477 | 0.77039169 | 0 |
| DMBSSR035 | BMD-18 | 0.01951937 | 0.72913833 | 0 |
| DMBSSR035 | BMD-23 | 0.01161483 | 0.56559875 | 0.2173913 |
| DMBSSR035 | BMD-26 | 0.01090284 | 0.62339812 | 0.47619048 |
| DMBSSR035 | BMD-29 | 0.01495129 | 0.62865862 | 0.01097695 |
| DMBSSR035 | BMD-31 | 0.01561081 | 0.57872409 | 0 |
| DMBSSR035 | BMD-48 | 0.01725469 | 0.58542879 | 0 |
| DMBSSR035 | BMD-35 | 0.01813324 | 0.57505544 | 0 |
| DMBSSR035 | BMD-47 | 0.01193793 | 0.61195278 | 0.16666667 |
| DMBSSR035 | BMD-50 | 0.01751301 | 0.5766956 | 0 |
| DMBSSR035 | BMD-51 | 0.01255606 | 0.58212701 | 0.02531646 |
| DMBSSR035 | BMD-12 | 0.01482614 | 0.55346258 | 0.0625 |
| DMBSSR035 | X21 | 0.01056523 | 0.56658155 | 0.26315789 |
| DMBSSR035 | X 34 | 0.0138146 | 0.62608587 | 0.00899101 |
| DMBSSR035 | X40 | 0.01619325 | 0.59381934 | 0.01703578 |
| DMBSSR035 | X49 | 0.01439186 | 0.56653311 | 0.08264463 |
| DMBSSR035 | X62 | 0.01232574 | 0.54938177 | 0.03115265 |
| DMBSSR035 | X65 | 0.01400657 | 0.56891651 | 0.001998 |
| DMBSSR035 | X87 | 0.01249533 | 0.71475936 | 0.0330033 |
| DMBSSR035 | VR011 | 0.01158819 | 0.62188376 | 0.13157895 |
| DMBSSR035 | VR013 | 0.00946519 | 0.53581056 | 0.28571429 |
| DMBSSR035 | VR015 | 0.01451545 | 0.54187939 | 0 |
| DMBSSR035 | VR016 | 0.01445228 | 0.65253187 | 0.001998 |
| DMBSSR035 | VR018 | 0.01398573 | 0.69675999 | 0 |
| DMBSSR035 | VR022 | 0.01744239 | 0.65274442 | 0 |
| DMBSSR035 | VR022 | 0.01391662 | 0.69207372 | 0.003996 |
| DMBSSR035 | VR024 | 0.01322022 | 0.50780255 | 0.03690037 |
| DMBSSR035 | VR025 | 0.01600736 | 0.56338623 | 0.001998 |
| DMBSSR035 | VR032 | 0.01382681 | 0.56860673 | 0.02293578 |
| DMBSSR035 | VR033 | 0.01588811 | 0.5912071 | 0.001998 |
| DMBSSR035 | VR035 | 0.01326869 | 0.5690097 | 0.13157895 |
| DMBSSR035 | VR037 | 0.01426563 | 0.63830613 | 0.002997 |
| DMBSSR035 | VR039 | 0.01375515 | 0.59490622 | 0.004995 |
| DMBSSR035 | VR048 | 0.0176823 | 0.60324707 | 0 |
| DMBSSR035 | DQ345305 | 0.01490433 | 0.64616647 | 0.00699301 |
| DMBSSR035 | CEDG036 | 0.01403373 | 0.54224951 | 0 |
| DMBSSR035 | CEDG291 | 0.01381171 | 0.65415389 | 0.001998 |
| DMBSSR035 | CEDG220 | 0.01526255 | 0.62120347 | 0.01133787 |
| DMBSSR035 | CEDG136 | 0.01841544 | 0.64681869 | 0.000999 |
| DMBSSR035 | CEDG100 | 0.01589049 | 0.7111576 | 0 |
| DMBSSR035 | CEDG096A | 0.011319 | 0.68611609 | 0.07246377 |
| DMBSSR035 | CP00361 | 0.01523068 | 0.62985805 | 0 |
| CEDG084 | J01263 | 0.0123607 | 0.59076327 | 0.13513514 |
| CEDG084 | PV-at001 | 0.00889662 | 0.46317041 | 0.55555556 |
| CEDG084 | PV-ag003 | 0.01131669 | 0.45716081 | 0.55555556 |
| CEDG084 | PV-ag005 | 0.01603369 | 0.56497683 | 0.00799201 |
| CEDG084 | Pv-ctt002 | 0.01174088 | 0.60721586 | 0.06535948 |
| CEDG084 | SSR-IAC 127 | 0.01044041 | 0.50781395 | 0.08928571 |
| CEDG084 | SSR-IAC 188 | 0.0097491 | 0.55287418 | 0.37037037 |
| CEDG084 | SSR-IAC 195 | 0.01199226 | 0.41619678 | 0.90909091 |
| CEDG084 | DQ469392 | 0.00995217 | 0.54293351 | 0.66666667 |
| CEDG084 | DQ469393 | 0.01097185 | 0.50545936 | 0.47619048 |
| CEDG084 | PvM03 | 0.01147 | 0.62991018 | 0.27027027 |
| CEDG084 | PvM22 | 0.01512456 | 0.51886003 | 0.01416431 |
| CEDG084 | BMD-5 | 0.01603386 | 0.5195118 | 0.01436782 |
| CEDG084 | BMD-6 | 0.01813762 | 0.42599578 | 0.004995 |
| CEDG084 | BMD-8 | 0.01349128 | 0.50323727 | 0.01362398 |
| CEDG084 | BMD-13 | 0.01147652 | 0.62636198 | 0.05319149 |
| CEDG084 | BMD-18 | 0.01386473 | 0.57123262 | 0.002997 |
| CEDG084 | BMD-23 | 0.01322364 | 0.50818306 | 0.06451613 |
| CEDG084 | BMD-26 | 0.01512085 | 0.59855129 | 0.002997 |
| CEDG084 | BMD-29 | 0.01480894 | 0.54605311 | 0.01468429 |
| CEDG084 | BMD-31 | 0.01424959 | 0.5051214 | 0.02777778 |
| CEDG084 | BMD-48 | 0.01585215 | 0.49230013 | 0.02985075 |
| CEDG084 | BMD-35 | 0.01355674 | 0.46739688 | 0.01883239 |
| CEDG084 | BMD-47 | 0.01826856 | 0.59984476 | 0.00799201 |
| CEDG084 | BMD-50 | 0.01260793 | 0.41748088 | 0.06578947 |
| CEDG084 | BMD-51 | 0.01197462 | 0.51572254 | 0.19607843 |
| CEDG084 | BMD-12 | 0.01642606 | 0.5102467 | 0.01183432 |
| CEDG084 | X21 | 0.01015152 | 0.50503244 | 0.2 |
| CEDG084 | X 34 | 0.01151999 | 0.55084511 | 0.17857143 |
| CEDG084 | X40 | 0.01494921 | 0.49259045 | 0.03802281 |
| CEDG084 | X49 | 0.0138043 | 0.50886496 | 0.02257336 |
| CEDG084 | X62 | 0.00970673 | 0.45614988 | 0.41666667 |
| CEDG084 | X65 | 0.01304521 | 0.51171394 | 0.01675042 |
| CEDG084 | X87 | 0.01268808 | 0.66682352 | 0.04385965 |
| CEDG084 | VR011 | 0.01189495 | 0.59181429 | 0.0255102 |
| CEDG084 | VR013 | 0.01336808 | 0.51387271 | 0.11627907 |
| CEDG084 | VR015 | 0.01305959 | 0.45928978 | 0.09433962 |
| CEDG084 | VR016 | 0.01803182 | 0.61317986 | 0.001998 |
| CEDG084 | VR018 | 0.0117032 | 0.59353719 | 0.25641026 |
| CEDG084 | VR022 | 0.01467615 | 0.54209819 | 0.001998 |
| CEDG084 | VR022 | 0.0126363 | 0.63596385 | 0.0462963 |
| CEDG084 | VR024 | 0.00934374 | 0.38639323 | 0.38461538 |
| CEDG084 | VR025 | 0.01114776 | 0.45294214 | 0.19607843 |
| CEDG084 | VR032 | 0.01252746 | 0.46704696 | 0.06369427 |
| CEDG084 | VR033 | 0.01615365 | 0.53182736 | 0.003996 |
| CEDG084 | VR035 | 0.01500885 | 0.50174072 | 0.000999 |
| CEDG084 | VR037 | 0.00966008 | 0.50198036 | 0.5 |
| CEDG084 | VR039 | 0.01127031 | 0.49123861 | 0.10989011 |
| CEDG084 | VR048 | 0.0107286 | 0.43869047 | 0.10416667 |
| CEDG084 | DQ345305 | 0.01015341 | 0.52675653 | 0.28571429 |
| CEDG084 | CEDG036 | 0.0114366 | 0.4427244 | 0.17857143 |
| CEDG084 | CEDG291 | 0.01134351 | 0.56229797 | 0.3125 |
| CEDG084 | CEDG220 | 0.0137522 | 0.55086098 | 0 |
| CEDG084 | CEDG136 | 0.00850717 | 0.47174956 | 0.76923077 |
| CEDG084 | CEDG100 | 0.01534347 | 0.64619133 | 0.00699301 |
| CEDG084 | CEDG096A | 0.01145514 | 0.63255966 | 0.0625 |
| CEDG084 | CP00361 | 0.01783562 | 0.56974565 | 0.000999 |
| CEDG084 | DMBSSR035 | 0.01368403 | 0.5794166 | 0.01165501 |
| CEDG185 | J01263 | 0.01812881 | 0.70237438 | 0 |
| CEDG185 | PV-at001 | 0.01456928 | 0.62485648 | 0.00599401 |
| CEDG185 | PV-ag003 | 0.01749573 | 0.61227069 | 0.00599401 |
| CEDG185 | PV-ag005 | 0.01654989 | 0.65082542 | 0.00799201 |
| CEDG185 | Pv-ctt002 | 0.01607994 | 0.72748244 | 0.002997 |
| CEDG185 | SSR-IAC 127 | 0.01139785 | 0.58815349 | 0.27027027 |
| CEDG185 | SSR-IAC 188 | 0.01446849 | 0.72083276 | 0.01144165 |
| CEDG185 | SSR-IAC 195 | 0.01272066 | 0.53547332 | 0.04385965 |
| CEDG185 | DQ469392 | 0.0194852 | 0.71111357 | 0 |
| CEDG185 | DQ469393 | 0.01546324 | 0.62001455 | 0.000999 |
| CEDG185 | PvM03 | 0.01168339 | 0.70617574 | 0.01841621 |
| CEDG185 | PvM22 | 0.01231814 | 0.53794475 | 0.07633588 |
| CEDG185 | BMD-5 | 0.01517358 | 0.59306476 | 0.001998 |
| CEDG185 | BMD-6 | 0.01670956 | 0.50561542 | 0.03021148 |
| CEDG185 | BMD-8 | 0.01893976 | 0.6457598 | 0 |
| CEDG185 | BMD-13 | 0.01519222 | 0.73165387 | 0 |
| CEDG185 | BMD-18 | 0.02174145 | 0.7232204 | 0 |
| CEDG185 | BMD-23 | 0.01871426 | 0.63216804 | 0.01912046 |
| CEDG185 | BMD-26 | 0.01291279 | 0.66270119 | 0.004995 |
| CEDG185 | BMD-29 | 0.0172039 | 0.66338035 | 0 |
| CEDG185 | BMD-31 | 0.02055428 | 0.67347878 | 0 |
| CEDG185 | BMD-48 | 0.01867787 | 0.59007575 | 0.000999 |
| CEDG185 | BMD-35 | 0.01879741 | 0.63541663 | 0 |
| CEDG185 | BMD-47 | 0.01552523 | 0.66948863 | 0.0308642 |
| CEDG185 | BMD-50 | 0.01947958 | 0.6157561 | 0 |
| CEDG185 | BMD-51 | 0.01609583 | 0.63985572 | 0.002997 |
| CEDG185 | BMD-12 | 0.01214213 | 0.53923735 | 0.10204082 |
| CEDG185 | X21 | 0.01429561 | 0.64812472 | 0 |
| CEDG185 | X 34 | 0.01930382 | 0.70855263 | 0 |
| CEDG185 | X40 | 0.01173957 | 0.55144305 | 0.01811594 |
| CEDG185 | X49 | 0.01521237 | 0.59439189 | 0.002997 |
| CEDG185 | X62 | 0.01124329 | 0.56113811 | 0.07751938 |
| CEDG185 | X65 | 0.02022129 | 0.59511976 | 0 |
| CEDG185 | X87 | 0.01541321 | 0.73216617 | 0.001998 |
| CEDG185 | VR011 | 0.01454432 | 0.67522763 | 0.000999 |
| CEDG185 | VR013 | 0.01101668 | 0.56249728 | 0.28571429 |
| CEDG185 | VR015 | 0.01365652 | 0.52278431 | 0.09803922 |
| CEDG185 | VR016 | 0.01649115 | 0.66925312 | 0 |
| CEDG185 | VR018 | 0.01551035 | 0.71358258 | 0 |
| CEDG185 | VR022 | 0.01571531 | 0.63738862 | 0.000999 |
| CEDG185 | VR022 | 0.01449849 | 0.70879135 | 0 |
| CEDG185 | VR024 | 0.00865851 | 0.46412082 | 0.5 |
| CEDG185 | VR025 | 0.01599845 | 0.59612025 | 0 |
| CEDG185 | VR032 | 0.01468126 | 0.59285609 | 0.02832861 |
| CEDG185 | VR033 | 0.01829486 | 0.61998452 | 0 |
| CEDG185 | VR035 | 0.0111398 | 0.52956331 | 0.14925373 |
| CEDG185 | VR037 | 0.01487077 | 0.68541686 | 0 |
| CEDG185 | VR039 | 0.01619509 | 0.66279572 | 0 |
| CEDG185 | VR048 | 0.01765947 | 0.60427225 | 0 |
| CEDG185 | DQ345305 | 0.01419966 | 0.64612473 | 0.06849315 |
| CEDG185 | CEDG036 | 0.01726077 | 0.59413401 | 0.000999 |
| CEDG185 | CEDG291 | 0.01830904 | 0.70997564 | 0 |
| CEDG185 | CEDG220 | 0.01745405 | 0.67325214 | 0 |
| CEDG185 | CEDG136 | 0.01488777 | 0.63328236 | 0 |
| CEDG185 | CEDG100 | 0.02880416 | 0.78656787 | 0 |
| CEDG185 | CEDG096A | 0.01363537 | 0.73827237 | 0 |
| CEDG185 | CP00361 | 0.01774527 | 0.69005157 | 0 |
| CEDG185 | DMBSSR035 | 0.01920411 | 0.72913684 | 0 |
| CEDG185 | CEDG084 | 0.01248761 | 0.57917763 | 0.11363636 |
| CEDC033 | J01263 | 0.01437121 | 0.69104935 | 0 |
| CEDC033 | PV-at001 | 0.01216368 | 0.57844746 | 0.14705882 |
| CEDC033 | PV-ag003 | 0.01779913 | 0.63541706 | 0 |
| CEDC033 | PV-ag005 | 0.01281199 | 0.6019714 | 0.05319149 |
| CEDC033 | Pv-ctt002 | 0.01241473 | 0.70401712 | 0.01663894 |
| CEDC033 | SSR-IAC 127 | 0.01269345 | 0.62886536 | 0.03424658 |
| CEDC033 | SSR-IAC 188 | 0.01236483 | 0.70869848 | 0.01531394 |
| CEDC033 | SSR-IAC 195 | 0.01585307 | 0.58686697 | 0.000999 |
| CEDC033 | DQ469392 | 0.01147635 | 0.65170098 | 0.5 |
| CEDC033 | DQ469393 | 0.01265619 | 0.59266492 | 0.09803922 |
| CEDC033 | PvM03 | 0.00995993 | 0.68461333 | 0.35714286 |
| CEDC033 | PvM22 | 0.01208276 | 0.54100732 | 0.2173913 |
| CEDC033 | BMD-5 | 0.01649883 | 0.61477574 | 0 |
| CEDC033 | BMD-6 | 0.01303825 | 0.44882392 | 0.27777778 |
| CEDC033 | BMD-8 | 0.01521337 | 0.59157679 | 0.001998 |
| CEDC033 | BMD-13 | 0.01176373 | 0.69937189 | 0.0617284 |
| CEDC033 | BMD-18 | 0.01609012 | 0.66702048 | 0 |
| CEDC033 | BMD-23 | 0.01454863 | 0.59378561 | 0.02237136 |
| CEDC033 | BMD-26 | 0.0126306 | 0.66282798 | 0.01302083 |
| CEDC033 | BMD-29 | 0.01526807 | 0.63267613 | 0.001998 |
| CEDC033 | BMD-31 | 0.01867725 | 0.66890967 | 0 |
| CEDC033 | BMD-48 | 0.01449867 | 0.56108179 | 0.000999 |
| CEDC033 | BMD-35 | 0.01247354 | 0.51172319 | 0.03690037 |
| CEDC033 | BMD-47 | 0.01147879 | 0.62550447 | 0.0280112 |
| CEDC033 | BMD-50 | 0.01346224 | 0.54860797 | 0.00599401 |
| CEDC033 | BMD-51 | 0.0151143 | 0.5984916 | 0.001998 |
| CEDC033 | BMD-12 | 0.01352927 | 0.56167151 | 0.12195122 |
| CEDC033 | X21 | 0.01437058 | 0.63172218 | 0.000999 |
| CEDC033 | X 34 | 0.01211865 | 0.61432399 | 0.10309278 |
| CEDC033 | X40 | 0.01537875 | 0.5955067 | 0.00599401 |
| CEDC033 | X49 | 0.01408345 | 0.56583129 | 0.01106195 |
| CEDC033 | X62 | 0.01319655 | 0.61321562 | 0.01022495 |
| CEDC033 | X65 | 0.01065216 | 0.54148167 | 0.16666667 |
| CEDC033 | X87 | 0.01059935 | 0.71144103 | 0.2 |
| CEDC033 | VR011 | 0.01074332 | 0.63609246 | 0.71428571 |
| CEDC033 | VR013 | 0.01236706 | 0.58945014 | 0.3030303 |
| CEDC033 | VR015 | 0.01116895 | 0.49579113 | 0.14285714 |
| CEDC033 | VR016 | 0.01442033 | 0.67841738 | 0.000999 |
| CEDC033 | VR018 | 0.01377145 | 0.68581512 | 0.01168224 |
| CEDC033 | VR022 | 0.0146344 | 0.63103428 | 0 |
| CEDC033 | VR022 | 0.01467735 | 0.72696963 | 0 |
| CEDC033 | VR024 | 0.01127713 | 0.47614576 | 0.22727273 |
| CEDC033 | VR025 | 0.01742214 | 0.63688376 | 0 |
| CEDC033 | VR032 | 0.01405539 | 0.58231465 | 0.001998 |
| CEDC033 | VR033 | 0.01293274 | 0.55672357 | 0.04694836 |
| CEDC033 | VR035 | 0.01278878 | 0.55259569 | 0.01831502 |
| CEDC033 | VR037 | 0.0117322 | 0.62533324 | 0.21276596 |
| CEDC033 | VR039 | 0.01853155 | 0.66269199 | 0 |
| CEDC033 | VR048 | 0.01258346 | 0.53586742 | 0.0166113 |
| CEDC033 | DQ345305 | 0.01127503 | 0.60871621 | 0.66666667 |
| CEDC033 | CEDG036 | 0.01529012 | 0.55824817 | 0.000999 |
| CEDC033 | CEDG291 | 0.01648866 | 0.71087634 | 0 |
| CEDC033 | CEDG220 | 0.01647053 | 0.65781223 | 0 |
| CEDC033 | CEDG136 | 0.01572236 | 0.64732346 | 0 |
| CEDC033 | CEDG100 | 0.01044688 | 0.65804055 | 0.24390244 |
| CEDC033 | CEDG096A | 0.01156265 | 0.71485041 | 0.05050505 |
| CEDC033 | CP00361 | 0.01414957 | 0.6236015 | 0.02164502 |
| CEDC033 | DMBSSR035 | 0.01663479 | 0.72103791 | 0 |
| CEDC033 | CEDG084 | 0.01254667 | 0.57116621 | 0.004995 |
| CEDC033 | CEDG185 | 0.01277104 | 0.65117755 | 0.00799201 |
| DMBSSR199 | J01263 | 0.01755291 | 0.60341376 | 0.001998 |
| DMBSSR199 | PV-at001 | 0.01141667 | 0.44467125 | 0.15384615 |
| DMBSSR199 | PV-ag003 | 0.01458552 | 0.47502579 | 0.07407407 |
| DMBSSR199 | PV-ag005 | 0.00961448 | 0.46353129 | 0.71428571 |
| DMBSSR199 | Pv-ctt002 | 0.01055711 | 0.57057608 | 0.625 |
| DMBSSR199 | SSR-IAC 127 | 0.01346117 | 0.50606198 | 0.04 |
| DMBSSR199 | SSR-IAC 188 | 0.01111664 | 0.55815284 | 0.625 |
| DMBSSR199 | SSR-IAC 195 | 0.00897526 | 0.36418974 | 0.47619048 |
| DMBSSR199 | DQ469392 | 0.01152902 | 0.5431246 | 0.23809524 |
| DMBSSR199 | DQ469393 | 0.013141 | 0.49564128 | 0.07194245 |
| DMBSSR199 | PvM03 | 0.01371713 | 0.61212855 | 0.004995 |
| DMBSSR199 | PvM22 | 0.01025033 | 0.42494477 | 0.90909091 |
| DMBSSR199 | BMD-5 | 0.01687664 | 0.48305197 | 0.0204499 |
| DMBSSR199 | BMD-6 | 0.0167292 | 0.39323117 | 0.3030303 |
| DMBSSR199 | BMD-8 | 0.01655385 | 0.48930671 | 0 |
| DMBSSR199 | BMD-13 | 0.01093134 | 0.58551266 | 0.27777778 |
| DMBSSR199 | BMD-18 | 0.01053887 | 0.5076272 | 0.3125 |
| DMBSSR199 | BMD-23 | 0.01098357 | 0.46439838 | 0.13513514 |
| DMBSSR199 | BMD-26 | 0.01074291 | 0.5298459 | 0.3030303 |
| DMBSSR199 | BMD-29 | 0.01700119 | 0.54585025 | 0.01234568 |
| DMBSSR199 | BMD-31 | 0.01651814 | 0.51457675 | 0.003996 |
| DMBSSR199 | BMD-48 | 0.01411573 | 0.45242079 | 0.00799201 |
| DMBSSR199 | BMD-35 | 0.01782899 | 0.46996095 | 0.003996 |
| DMBSSR199 | BMD-47 | 0.0103267 | 0.50568863 | 0.32258065 |
| DMBSSR199 | BMD-50 | 0.01092945 | 0.3981353 | 0.11111111 |
| DMBSSR199 | BMD-51 | 0.01355221 | 0.48958976 | 0.03215434 |
| DMBSSR199 | BMD-12 | 0.00860627 | 0.37974418 | 0.45454545 |
| DMBSSR199 | X21 | 0.01398491 | 0.4972376 | 0.22727273 |
| DMBSSR199 | X 34 | 0.00894092 | 0.48155656 | 0.76923077 |
| DMBSSR199 | X40 | 0.01000083 | 0.41652401 | 0.66666667 |
| DMBSSR199 | X49 | 0.01272456 | 0.44732913 | 0.22222222 |
| DMBSSR199 | X62 | 0.01185568 | 0.43933656 | 0.10309278 |
| DMBSSR199 | X65 | 0.01462653 | 0.48032337 | 0.06666667 |
| DMBSSR199 | X87 | 0.00968332 | 0.58696196 | 0.625 |
| DMBSSR199 | VR011 | 0.0083305 | 0.48605309 | 0.625 |
| DMBSSR199 | VR013 | 0.00872477 | 0.41981905 | 0.76923077 |
| DMBSSR199 | VR015 | 0.01361435 | 0.42156763 | 0.11235955 |
| DMBSSR199 | VR016 | 0.01423091 | 0.5517065 | 0.03521127 |
| DMBSSR199 | VR018 | 0.01395676 | 0.5858798 | 0.01715266 |
| DMBSSR199 | VR022 | 0.0210264 | 0.55285257 | 0 |
| DMBSSR199 | VR022 | 0.01475669 | 0.60184863 | 0.01295337 |
| DMBSSR199 | VR024 | 0.01063657 | 0.40247698 | 0.19607843 |
| DMBSSR199 | VR025 | 0.01153002 | 0.4324536 | 0.32258065 |
| DMBSSR199 | VR032 | 0.00871695 | 0.41962398 | 0.35714286 |
| DMBSSR199 | VR033 | 0.0141503 | 0.48447742 | 0.00899101 |
| DMBSSR199 | VR035 | 0.01446303 | 0.44847845 | 0.002997 |
| DMBSSR199 | VR037 | 0.01187216 | 0.52262008 | 0.0877193 |
| DMBSSR199 | VR039 | 0.0150811 | 0.50999002 | 0.05524862 |
| DMBSSR199 | VR048 | 0.01390615 | 0.43224376 | 0.04385965 |
| DMBSSR199 | DQ345305 | 0.01310737 | 0.52974421 | 0.11363636 |
| DMBSSR199 | CEDG036 | 0.02594269 | 0.47037128 | 0 |
| DMBSSR199 | CEDG291 | 0.02390345 | 0.62582185 | 0.000999 |
| DMBSSR199 | CEDG220 | 0.01366168 | 0.51550561 | 0.00599401 |
| DMBSSR199 | CEDG136 | 0.01243258 | 0.49156161 | 0.3030303 |
| DMBSSR199 | CEDG100 | 0.01390107 | 0.58589616 | 0.5 |
| DMBSSR199 | CEDG096A | 0.01262826 | 0.61869406 | 0.11627907 |
| DMBSSR199 | CP00361 | 0.01269925 | 0.49572878 | 0.08474576 |
| DMBSSR199 | DMBSSR035 | 0.01267852 | 0.52683075 | 0.04424779 |
| DMBSSR199 | CEDG084 | 0.00984353 | 0.41777766 | 0.34482759 |
| DMBSSR199 | CEDG185 | 0.01237002 | 0.5568973 | 0.18181818 |
| DMBSSR199 | CEDC033 | 0.02076937 | 0.62305455 | 0 |
| DMBSSR024 | J01263 | 0.01531891 | 0.66189392 | 0.000999 |
| DMBSSR024 | PV-at001 | 0.01102924 | 0.5272206 | 0.27027027 |
| DMBSSR024 | PV-ag003 | 0.01071291 | 0.48677553 | 0.41666667 |
| DMBSSR024 | PV-ag005 | 0.01388594 | 0.59061959 | 0.01680672 |
| DMBSSR024 | Pv-ctt002 | 0.01579594 | 0.70359823 | 0.00899101 |
| DMBSSR024 | SSR-IAC 127 | 0.01341136 | 0.54702899 | 0.0127551 |
| DMBSSR024 | SSR-IAC 188 | 0.01175853 | 0.64491113 | 0.17241379 |
| DMBSSR024 | SSR-IAC 195 | 0.01125961 | 0.48162338 | 0.43478261 |
| DMBSSR024 | DQ469392 | 0.01260438 | 0.61550858 | 0.02840909 |
| DMBSSR024 | DQ469393 | 0.01415605 | 0.56555632 | 0.01594896 |
| DMBSSR024 | PvM03 | 0.01548925 | 0.70888208 | 0 |
| DMBSSR024 | PvM22 | 0.00970484 | 0.46653161 | 0.25 |
| DMBSSR024 | BMD-5 | 0.01620309 | 0.55103652 | 0.004995 |
| DMBSSR024 | BMD-6 | 0.01614791 | 0.42658048 | 0.05882353 |
| DMBSSR024 | BMD-8 | 0.01499011 | 0.56505663 | 0.002997 |
| DMBSSR024 | BMD-13 | 0.01292044 | 0.68317943 | 0.01322751 |
| DMBSSR024 | BMD-18 | 0.01735824 | 0.65258053 | 0 |
| DMBSSR024 | BMD-23 | 0.01408225 | 0.53346327 | 0.13513514 |
| DMBSSR024 | BMD-26 | 0.00838905 | 0.54960009 | 0.76923077 |
| DMBSSR024 | BMD-29 | 0.01375648 | 0.55418337 | 0.03861004 |
| DMBSSR024 | BMD-31 | 0.01333064 | 0.54863808 | 0.01428571 |
| DMBSSR024 | BMD-48 | 0.01847346 | 0.53175202 | 0.000999 |
| DMBSSR024 | BMD-35 | 0.01349486 | 0.51607791 | 0.004995 |
| DMBSSR024 | BMD-47 | 0.01344728 | 0.5885101 | 0.08547009 |
| DMBSSR024 | BMD-50 | 0.01645329 | 0.52886011 | 0.000999 |
| DMBSSR024 | BMD-51 | 0.01267718 | 0.53876596 | 0.13888889 |
| DMBSSR024 | BMD-12 | 0.01151295 | 0.502435 | 0.12048193 |
| DMBSSR024 | X21 | 0.0083765 | 0.49729909 | 0.83333333 |
| DMBSSR024 | X 34 | 0.01117165 | 0.56164945 | 0.3125 |
| DMBSSR024 | X40 | 0.01179369 | 0.48679912 | 0.3030303 |
| DMBSSR024 | X49 | 0.01480893 | 0.54022687 | 0.003996 |
| DMBSSR024 | X62 | 0.01198171 | 0.50183559 | 0.11363636 |
| DMBSSR024 | X65 | 0.01668229 | 0.56570256 | 0 |
| DMBSSR024 | X87 | 0.01425496 | 0.69295902 | 0.01736111 |
| DMBSSR024 | VR011 | 0.01230874 | 0.61963733 | 0.27777778 |
| DMBSSR024 | VR013 | 0.01227541 | 0.537921 | 0.37037037 |
| DMBSSR024 | VR015 | 0.01524911 | 0.49989133 | 0.00799201 |
| DMBSSR024 | VR016 | 0.01274482 | 0.58691336 | 0.08474576 |
| DMBSSR024 | VR018 | 0.01315141 | 0.65930737 | 0.00899101 |
| DMBSSR024 | VR022 | 0.0120164 | 0.53770808 | 0.10416667 |
| DMBSSR024 | VR022 | 0.0116539 | 0.64131771 | 0.0462963 |
| DMBSSR024 | VR024 | 0.01097845 | 0.44550364 | 0.26315789 |
| DMBSSR024 | VR025 | 0.0122603 | 0.50544275 | 0.04255319 |
| DMBSSR024 | VR032 | 0.01057354 | 0.48846434 | 0.34482759 |
| DMBSSR024 | VR033 | 0.01288946 | 0.52210859 | 0.06410256 |
| DMBSSR024 | VR035 | 0.01296617 | 0.50371652 | 0.00899101 |
| DMBSSR024 | VR037 | 0.01312829 | 0.59309309 | 0.08403361 |
| DMBSSR024 | VR039 | 0.01443369 | 0.58854925 | 0 |
| DMBSSR024 | VR048 | 0.01418023 | 0.51990207 | 0.002997 |
| DMBSSR024 | DQ345305 | 0.01127185 | 0.58541467 | 0.15625 |
| DMBSSR024 | CEDG036 | 0.01691189 | 0.51540252 | 0.01089325 |
| DMBSSR024 | CEDG291 | 0.02809359 | 0.73152666 | 0 |
| DMBSSR024 | CEDG220 | 0.02071212 | 0.6493154 | 0 |
| DMBSSR024 | CEDG136 | 0.01447117 | 0.59564915 | 0.01639344 |
| DMBSSR024 | CEDG100 | 0.01396173 | 0.65712617 | 0.0265252 |
| DMBSSR024 | CEDG096A | 0.0148267 | 0.712228 | 0 |
| DMBSSR024 | CP00361 | 0.02021029 | 0.64610248 | 0 |
| DMBSSR024 | DMBSSR035 | 0.01799275 | 0.66391912 | 0 |
| DMBSSR024 | CEDG084 | 0.01375305 | 0.56721217 | 0 |
| DMBSSR024 | CEDG185 | 0.0135859 | 0.61366918 | 0.002997 |
| DMBSSR024 | CEDC033 | 0.01530498 | 0.67026596 | 0 |
| DMBSSR024 | DMBSSR199 | 0.02192541 | 0.56976762 | 0 |
| GMES0337 | J01263 | 0.01164242 | 0.561049 | 0.09090909 |
| GMES0337 | PV-at001 | 0.01652379 | 0.53278429 | 0.01584786 |
| GMES0337 | PV-ag003 | 0.01211097 | 0.47065188 | 0.0625 |
| GMES0337 | PV-ag005 | 0.01298437 | 0.52408819 | 0.14285714 |
| GMES0337 | Pv-ctt002 | 0.01287559 | 0.63272008 | 0.06896552 |
| GMES0337 | SSR-IAC 127 | 0.00895537 | 0.46907857 | 0.625 |
| GMES0337 | SSR-IAC 188 | 0.01255661 | 0.59738679 | 0.41666667 |
| GMES0337 | SSR-IAC 195 | 0.00919937 | 0.39539543 | 0.71428571 |
| GMES0337 | DQ469392 | 0.01407227 | 0.58234436 | 0.04854369 |
| GMES0337 | DQ469393 | 0.01284413 | 0.49254458 | 0.41666667 |
| GMES0337 | PvM03 | 0.00968496 | 0.59963464 | 0.52631579 |
| GMES0337 | PvM22 | 0.01341859 | 0.46191699 | 0.3125 |
| GMES0337 | BMD-5 | 0.02202896 | 0.54404268 | 0.003996 |
| GMES0337 | BMD-6 | 0.01411992 | 0.37309376 | 0.26315789 |
| GMES0337 | BMD-8 | 0.01123394 | 0.46518546 | 0.15151515 |
| GMES0337 | BMD-13 | 0.01042999 | 0.62592071 | 0.34482759 |
| GMES0337 | BMD-18 | 0.01595858 | 0.60789492 | 0.001998 |
| GMES0337 | BMD-23 | 0.01646802 | 0.54455562 | 0.00599401 |
| GMES0337 | BMD-26 | 0.01011136 | 0.55950215 | 0.43478261 |
| GMES0337 | BMD-29 | 0.01373277 | 0.50816248 | 0.5 |
| GMES0337 | BMD-31 | 0.01288677 | 0.48599878 | 0.1010101 |
| GMES0337 | BMD-48 | 0.01350901 | 0.44782396 | 0.04 |
| GMES0337 | BMD-35 | 0.01104853 | 0.42792639 | 0.38461538 |
| GMES0337 | BMD-47 | 0.0137308 | 0.58098621 | 0.01123596 |
| GMES0337 | BMD-50 | 0.01499689 | 0.48159277 | 0.00899101 |
| GMES0337 | BMD-51 | 0.01542387 | 0.55242409 | 0.00699301 |
| GMES0337 | BMD-12 | 0.01056609 | 0.44832092 | 0.41666667 |
| GMES0337 | X21 | 0.01324401 | 0.55069913 | 0.08403361 |
| GMES0337 | X 34 | 0.00858249 | 0.47996983 | 0.90909091 |
| GMES0337 | X40 | 0.01750792 | 0.52889728 | 0.0141844 |
| GMES0337 | X49 | 0.01401838 | 0.48405186 | 0.04694836 |
| GMES0337 | X62 | 0.01030666 | 0.43190038 | 0.38461538 |
| GMES0337 | X65 | 0.01410571 | 0.50644932 | 0.03436426 |
| GMES0337 | X87 | 0.00903005 | 0.59083725 | 0.76923077 |
| GMES0337 | VR011 | 0.01195599 | 0.56186173 | 0.5 |
| GMES0337 | VR013 | 0.01303251 | 0.47823839 | 0.17241379 |
| GMES0337 | VR015 | 0.01315403 | 0.43710006 | 0.12658228 |
| GMES0337 | VR016 | 0.01491204 | 0.56196969 | 0.04366812 |
| GMES0337 | VR018 | 0.01509794 | 0.65169523 | 0.004995 |
| GMES0337 | VR022 | 0.01130217 | 0.46441641 | 0.76923077 |
| GMES0337 | VR022 | 0.01445122 | 0.6106589 | 0.00799201 |
| GMES0337 | VR024 | 0.012205 | 0.40596011 | 0.27777778 |
| GMES0337 | VR025 | 0.0141431 | 0.49523275 | 0.01367989 |
| GMES0337 | VR032 | 0.01249725 | 0.47398319 | 0.27027027 |
| GMES0337 | VR033 | 0.01449847 | 0.47223941 | 0.06329114 |
| GMES0337 | VR035 | 0.00919724 | 0.43186662 | 0.25 |
| GMES0337 | VR037 | 0.0096894 | 0.51300141 | 0.55555556 |
| GMES0337 | VR039 | 0.0157631 | 0.55840844 | 0.002997 |
| GMES0337 | VR048 | 0.01257801 | 0.45071789 | 0.25641026 |
| GMES0337 | DQ345305 | 0.00924499 | 0.50716519 | 0.71428571 |
| GMES0337 | CEDG036 | 0.01471158 | 0.46401906 | 0.13333333 |
| GMES0337 | CEDG291 | 0.01749628 | 0.63679059 | 0 |
| GMES0337 | CEDG220 | 0.01374365 | 0.55370735 | 0.07092199 |
| GMES0337 | CEDG136 | 0.01745773 | 0.59549984 | 0 |
| GMES0337 | CEDG100 | 0.01288381 | 0.64205552 | 0.02331002 |
| GMES0337 | CEDG096A | 0.01259645 | 0.63479268 | 0.03144654 |
| GMES0337 | CP00361 | 0.01836863 | 0.55653778 | 0.00699301 |
| GMES0337 | DMBSSR035 | 0.01350883 | 0.5699986 | 0.003996 |
| GMES0337 | CEDG084 | 0.01110022 | 0.48968885 | 0.37037037 |
| GMES0337 | CEDG185 | 0.01698091 | 0.64475805 | 0.000999 |
| GMES0337 | CEDC033 | 0.01456699 | 0.60389359 | 0.004995 |
| GMES0337 | DMBSSR199 | 0.01690369 | 0.51347291 | 0.01282051 |
| GMES0337 | DMBSSR024 | 0.02093012 | 0.58250833 | 0.000999 |
| DMBSSR001 | J01263 | 0.01334597 | 0.59085556 | 0.06410256 |
| DMBSSR001 | PV-at001 | 0.01546121 | 0.50854209 | 0.1 |
| DMBSSR001 | PV-ag003 | 0.01261551 | 0.45679968 | 0.3125 |
| DMBSSR001 | PV-ag005 | 0.01916439 | 0.59272881 | 0 |
| DMBSSR001 | Pv-ctt002 | 0.01518263 | 0.64873309 | 0.004995 |
| DMBSSR001 | SSR-IAC 127 | 0.00953802 | 0.44848486 | 0.22222222 |
| DMBSSR001 | SSR-IAC 188 | 0.01478661 | 0.65323923 | 0.001998 |
| DMBSSR001 | SSR-IAC 195 | 0.01605188 | 0.49963635 | 0.07575758 |
| DMBSSR001 | DQ469392 | 0.01238886 | 0.55133487 | 0.10638298 |
| DMBSSR001 | DQ469393 | 0.01371352 | 0.50793443 | 0.10416667 |
| DMBSSR001 | PvM03 | 0.01184672 | 0.61324713 | 0.18181818 |
| DMBSSR001 | PvM22 | 0.0132123 | 0.4667967 | 0.01845018 |
| DMBSSR001 | BMD-5 | 0.02662049 | 0.57594589 | 0 |
| DMBSSR001 | BMD-6 | 0.01010548 | 0.35936152 | 0.25 |
| DMBSSR001 | BMD-8 | 0.02228736 | 0.54231384 | 0 |
| DMBSSR001 | BMD-13 | 0.01335818 | 0.63705931 | 0.002997 |
| DMBSSR001 | BMD-18 | 0.01680631 | 0.5992189 | 0 |
| DMBSSR001 | BMD-23 | 0.01395744 | 0.50099306 | 0.0155521 |
| DMBSSR001 | BMD-26 | 0.01450564 | 0.60511065 | 0.001998 |
| DMBSSR001 | BMD-29 | 0.01651621 | 0.55693497 | 0.003996 |
| DMBSSR001 | BMD-31 | 0.01983438 | 0.56610921 | 0 |
| DMBSSR001 | BMD-48 | 0.02270888 | 0.52473872 | 0.000999 |
| DMBSSR001 | BMD-35 | 0.01817459 | 0.51788107 | 0 |
| DMBSSR001 | BMD-47 | 0.0108843 | 0.52407971 | 0.16393443 |
| DMBSSR001 | BMD-50 | 0.01750632 | 0.47689029 | 0 |
| DMBSSR001 | BMD-51 | 0.01523184 | 0.51879464 | 0.002997 |
| DMBSSR001 | BMD-12 | 0.01467952 | 0.51370709 | 0.02695418 |
| DMBSSR001 | X21 | 0.01624528 | 0.54913267 | 0 |
| DMBSSR001 | X 34 | 0.01331951 | 0.54350903 | 0.002997 |
| DMBSSR001 | X40 | 0.01497375 | 0.47929211 | 0.01976285 |
| DMBSSR001 | X49 | 0.0134194 | 0.46671303 | 0.08403361 |
| DMBSSR001 | X62 | 0.01140233 | 0.46329865 | 0.15625 |
| DMBSSR001 | X65 | 0.01162592 | 0.45676444 | 0.06024096 |
| DMBSSR001 | X87 | 0.0122892 | 0.64102944 | 0.05025126 |
| DMBSSR001 | VR011 | 0.0168691 | 0.63784518 | 0.001998 |
| DMBSSR001 | VR013 | 0.01326967 | 0.49440152 | 0.04854369 |
| DMBSSR001 | VR015 | 0.01283613 | 0.41505555 | 0.10869565 |
| DMBSSR001 | VR016 | 0.01099387 | 0.51879853 | 0.10638298 |
| DMBSSR001 | VR018 | 0.01254413 | 0.5940903 | 0.05235602 |
| DMBSSR001 | VR022 | 0.01697418 | 0.5251655 | 0.00799201 |
| DMBSSR001 | VR022 | 0.01384605 | 0.60730375 | 0.000999 |
| DMBSSR001 | VR024 | 0.01141319 | 0.40114055 | 0.76923077 |
| DMBSSR001 | VR025 | 0.01584304 | 0.50215479 | 0.003996 |
| DMBSSR001 | VR032 | 0.0111759 | 0.44979833 | 0.38461538 |
| DMBSSR001 | VR033 | 0.01222843 | 0.47279729 | 0.10416667 |
| DMBSSR001 | VR035 | 0.01384027 | 0.47469244 | 0.02695418 |
| DMBSSR001 | VR037 | 0.01209502 | 0.52743386 | 0.01949318 |
| DMBSSR001 | VR039 | 0.01549055 | 0.5688956 | 0 |
| DMBSSR001 | VR048 | 0.02079285 | 0.54587912 | 0 |
| DMBSSR001 | DQ345305 | 0.01715301 | 0.58798324 | 0.002997 |
| DMBSSR001 | CEDG036 | 0.01748329 | 0.50362824 | 0.000999 |
| DMBSSR001 | CEDG291 | 0.01228757 | 0.58034124 | 0.00699301 |
| DMBSSR001 | CEDG220 | 0.01752043 | 0.59550179 | 0.000999 |
| DMBSSR001 | CEDG136 | 0.01730085 | 0.60374048 | 0.002997 |
| DMBSSR001 | CEDG100 | 0.01962027 | 0.67962401 | 0 |
| DMBSSR001 | CEDG096A | 0.01340405 | 0.6609359 | 0.000999 |
| DMBSSR001 | CP00361 | 0.02032225 | 0.61635198 | 0 |
| DMBSSR001 | DMBSSR035 | 0.02100374 | 0.64752874 | 0 |
| DMBSSR001 | CEDG084 | 0.01319057 | 0.46310561 | 0.1010101 |
| DMBSSR001 | CEDG185 | 0.0162305 | 0.61659581 | 0 |
| DMBSSR001 | CEDC033 | 0.01498567 | 0.58728279 | 0.001998 |
| DMBSSR001 | DMBSSR199 | 0.01570827 | 0.50859967 | 0.01031992 |
| DMBSSR001 | DMBSSR024 | 0.01889112 | 0.6056267 | 0 |
| DMBSSR001 | GMES0337 | 0.01003665 | 0.45756028 | 0.24390244 |
| CEDG118 | J01263 | 0.01412402 | 0.62120482 | 0.002997 |
| CEDG118 | PV-at001 | 0.01345654 | 0.5130492 | 0.20833333 |
| CEDG118 | PV-ag003 | 0.01682757 | 0.55199395 | 0.01381215 |
| CEDG118 | PV-ag005 | 0.01729455 | 0.56975202 | 0.03759398 |
| CEDG118 | Pv-ctt002 | 0.01222794 | 0.63155701 | 0.00799201 |
| CEDG118 | SSR-IAC 127 | 0.01141526 | 0.52581289 | 0.38461538 |
| CEDG118 | SSR-IAC 188 | 0.01359175 | 0.63071459 | 0.03891051 |
| CEDG118 | SSR-IAC 195 | 0.01036131 | 0.41965288 | 0.71428571 |
| CEDG118 | DQ469392 | 0.01180328 | 0.58518925 | 0.18181818 |
| CEDG118 | DQ469393 | 0.01377096 | 0.55245793 | 0.03571429 |
| CEDG118 | PvM03 | 0.01034326 | 0.62619645 | 0.47619048 |
| CEDG118 | PvM22 | 0.00960124 | 0.43958177 | 0.35714286 |
| CEDG118 | BMD-5 | 0.01831365 | 0.53689442 | 0.004995 |
| CEDG118 | BMD-6 | 0.02434437 | 0.50268642 | 0 |
| CEDG118 | BMD-8 | 0.0108808 | 0.45594346 | 0.08849558 |
| CEDG118 | BMD-13 | 0.01356824 | 0.65240224 | 0.01127396 |
| CEDG118 | BMD-18 | 0.01579682 | 0.62738799 | 0 |
| CEDG118 | BMD-23 | 0.015747 | 0.54674267 | 0.01964637 |
| CEDG118 | BMD-26 | 0.01728017 | 0.60435598 | 0 |
| CEDG118 | BMD-29 | 0.00975928 | 0.48478245 | 0.32258065 |
| CEDG118 | BMD-31 | 0.02020251 | 0.56187725 | 0 |
| CEDG118 | BMD-48 | 0.02353683 | 0.56718396 | 0 |
| CEDG118 | BMD-35 | 0.01337049 | 0.48134184 | 0.05952381 |
| CEDG118 | BMD-47 | 0.01063034 | 0.54761477 | 0.43478261 |
| CEDG118 | BMD-50 | 0.02230054 | 0.5460129 | 0 |
| CEDG118 | BMD-51 | 0.01691463 | 0.59281395 | 0 |
| CEDG118 | BMD-12 | 0.01147786 | 0.47070835 | 0.24390244 |
| CEDG118 | X21 | 0.01691545 | 0.59008235 | 0 |
| CEDG118 | X 34 | 0.01376872 | 0.58646741 | 0.01464129 |
| CEDG118 | X40 | 0.01233291 | 0.48362704 | 0.08928571 |
| CEDG118 | X49 | 0.01140334 | 0.47070033 | 0.38461538 |
| CEDG118 | X62 | 0.01178515 | 0.49444527 | 0.08403361 |
| CEDG118 | X65 | 0.01588023 | 0.54560226 | 0 |
| CEDG118 | X87 | 0.01375504 | 0.67102755 | 0.00699301 |
| CEDG118 | VR011 | 0.01739093 | 0.65056895 | 0 |
| CEDG118 | VR013 | 0.01355276 | 0.54914552 | 0.13513514 |
| CEDG118 | VR015 | 0.01327558 | 0.43144411 | 0.27027027 |
| CEDG118 | VR016 | 0.01396213 | 0.59981969 | 0.01356852 |
| CEDG118 | VR018 | 0.01347849 | 0.63454191 | 0.00599401 |
| CEDG118 | VR022 | 0.01819457 | 0.5775843 | 0.000999 |
| CEDG118 | VR022 | 0.01458412 | 0.67231473 | 0.004995 |
| CEDG118 | VR024 | 0.01113327 | 0.42018543 | 0.43478261 |
| CEDG118 | VR025 | 0.01596931 | 0.52222249 | 0.001998 |
| CEDG118 | VR032 | 0.00951519 | 0.45568518 | 0.43478261 |
| CEDG118 | VR033 | 0.01265233 | 0.4671309 | 0.16129032 |
| CEDG118 | VR035 | 0.01460489 | 0.48916961 | 0.03134796 |
| CEDG118 | VR037 | 0.01156105 | 0.55994271 | 0.06493506 |
| CEDG118 | VR039 | 0.01961883 | 0.59332408 | 0 |
| CEDG118 | VR048 | 0.01573755 | 0.54841019 | 0.001998 |
| CEDG118 | DQ345305 | 0.01356593 | 0.61081296 | 0.01712329 |
| CEDG118 | CEDG036 | 0.01208169 | 0.46991837 | 0.05076142 |
| CEDG118 | CEDG291 | 0.01375334 | 0.62437086 | 0.05 |
| CEDG118 | CEDG220 | 0.01557039 | 0.59425345 | 0.000999 |
| CEDG118 | CEDG136 | 0.01639894 | 0.62281349 | 0.000999 |
| CEDG118 | CEDG100 | 0.01669086 | 0.65535808 | 0.000999 |
| CEDG118 | CEDG096A | 0.01279735 | 0.6739656 | 0.02785515 |
| CEDG118 | CP00361 | 0.01281661 | 0.53494507 | 0.01416431 |
| CEDG118 | DMBSSR035 | 0.01742485 | 0.66985417 | 0 |
| CEDG118 | CEDG084 | 0.01216117 | 0.53298618 | 0.04347826 |
| CEDG118 | CEDG185 | 0.01766052 | 0.65395951 | 0 |
| CEDG118 | CEDC033 | 0.01929277 | 0.6876964 | 0 |
| CEDG118 | DMBSSR199 | 0.01530516 | 0.5172885 | 0.29411765 |
| CEDG118 | DMBSSR024 | 0.0171421 | 0.6159563 | 0.000999 |
| CEDG118 | GMES0337 | 0.01156977 | 0.48451484 | 0.52631579 |
| CEDG118 | DMBSSR001 | 0.02613863 | 0.63979296 | 0 |
| CEDG146 | J01263 | 0.01922975 | 0.63118767 | 0 |
| CEDG146 | PV-at001 | 0.01742076 | 0.52548189 | 0 |
| CEDG146 | PV-ag003 | 0.01451338 | 0.50528258 | 0.00799201 |
| CEDG146 | PV-ag005 | 0.01406022 | 0.54211225 | 0.02710027 |
| CEDG146 | Pv-ctt002 | 0.01443507 | 0.64285025 | 0 |
| CEDG146 | SSR-IAC 127 | 0.00990604 | 0.49225208 | 0.41666667 |
| CEDG146 | SSR-IAC 188 | 0.00973714 | 0.57309246 | 0.1754386 |
| CEDG146 | SSR-IAC 195 | 0.01791434 | 0.46413359 | 0.004995 |
| CEDG146 | DQ469392 | 0.01237444 | 0.55513686 | 0.0990099 |
| CEDG146 | DQ469393 | 0.01504034 | 0.5294851 | 0.04310345 |
| CEDG146 | PvM03 | 0.0134303 | 0.63235171 | 0.11627907 |
| CEDG146 | PvM22 | 0.01585203 | 0.4883179 | 0.08403361 |
| CEDG146 | BMD-5 | 0.01224615 | 0.47378725 | 0.14925373 |
| CEDG146 | BMD-6 | 0.01093451 | 0.36370815 | 0.20408163 |
| CEDG146 | BMD-8 | 0.01868637 | 0.52968222 | 0 |
| CEDG146 | BMD-13 | 0.01850843 | 0.71376261 | 0 |
| CEDG146 | BMD-18 | 0.02330841 | 0.65879262 | 0 |
| CEDG146 | BMD-23 | 0.01532908 | 0.50367074 | 0.07246377 |
| CEDG146 | BMD-26 | 0.01111014 | 0.55122682 | 0.625 |
| CEDG146 | BMD-29 | 0.01706473 | 0.55255073 | 0.01474926 |
| CEDG146 | BMD-31 | 0.01399136 | 0.518587 | 0.000999 |
| CEDG146 | BMD-48 | 0.01873456 | 0.49857234 | 0.00699301 |
| CEDG146 | BMD-35 | 0.01566459 | 0.48754126 | 0.001998 |
| CEDG146 | BMD-47 | 0.01406738 | 0.57114677 | 0.25641026 |
| CEDG146 | BMD-50 | 0.02741821 | 0.58068897 | 0 |
| CEDG146 | BMD-51 | 0.01897751 | 0.55667295 | 0 |
| CEDG146 | BMD-12 | 0.01306066 | 0.47005372 | 0.1 |
| CEDG146 | X21 | 0.00924842 | 0.47514964 | 0.71428571 |
| CEDG146 | X 34 | 0.01618051 | 0.58802878 | 0 |
| CEDG146 | X40 | 0.01115891 | 0.45776191 | 0.45454545 |
| CEDG146 | X49 | 0.0091663 | 0.39817343 | 0.71428571 |
| CEDG146 | X62 | 0.01231385 | 0.50931401 | 0.04149378 |
| CEDG146 | X65 | 0.01431554 | 0.50428238 | 0.02506266 |
| CEDG146 | X87 | 0.01257782 | 0.63692603 | 0.09433962 |
| CEDG146 | VR011 | 0.01599672 | 0.6089162 | 0.00899101 |
| CEDG146 | VR013 | 0.01073971 | 0.4806354 | 0.35714286 |
| CEDG146 | VR015 | 0.01620208 | 0.43735008 | 0.001998 |
| CEDG146 | VR016 | 0.01439764 | 0.57507991 | 0.05524862 |
| CEDG146 | VR018 | 0.01502286 | 0.63666832 | 0 |
| CEDG146 | VR022 | 0.01166882 | 0.5069039 | 0.08695652 |
| CEDG146 | VR022 | 0.01519789 | 0.64184835 | 0.002997 |
| CEDG146 | VR024 | 0.00905124 | 0.38640963 | 0.10309278 |
| CEDG146 | VR025 | 0.01401651 | 0.49068008 | 0.001998 |
| CEDG146 | VR032 | 0.0106221 | 0.44826803 | 0.08695652 |
| CEDG146 | VR033 | 0.01464864 | 0.53494095 | 0.01322751 |
| CEDG146 | VR035 | 0.01170516 | 0.45712873 | 0.05988024 |
| CEDG146 | VR037 | 0.01148652 | 0.55952727 | 0.04048583 |
| CEDG146 | VR039 | 0.01656522 | 0.56881158 | 0.004995 |
| CEDG146 | VR048 | 0.01624398 | 0.50675753 | 0 |
| CEDG146 | DQ345305 | 0.01323003 | 0.54785058 | 0.24390244 |
| CEDG146 | CEDG036 | 0.00952078 | 0.39517819 | 0.76923077 |
| CEDG146 | CEDG291 | 0.01485167 | 0.59779626 | 0 |
| CEDG146 | CEDG220 | 0.01872102 | 0.58177054 | 0 |
| CEDG146 | CEDG136 | 0.01767606 | 0.58091437 | 0.004995 |
| CEDG146 | CEDG100 | 0.01563576 | 0.6469482 | 0.001998 |
| CEDG146 | CEDG096A | 0.01882561 | 0.72035475 | 0 |
| CEDG146 | CP00361 | 0.01834155 | 0.59751353 | 0 |
| CEDG146 | DMBSSR035 | 0.01787938 | 0.64784065 | 0 |
| CEDG146 | CEDG084 | 0.01102593 | 0.47264767 | 0.06493506 |
| CEDG146 | CEDG185 | 0.02356613 | 0.69806983 | 0 |
| CEDG146 | CEDC033 | 0.01260117 | 0.58029516 | 0.0330033 |
| CEDG146 | DMBSSR199 | 0.01475776 | 0.48877503 | 0.00599401 |
| CEDG146 | DMBSSR024 | 0.01527325 | 0.57137722 | 0.01883239 |
| CEDG146 | GMES0337 | 0.01430062 | 0.50493391 | 0.15384615 |
| CEDG146 | DMBSSR001 | 0.01481303 | 0.52355736 | 0.001998 |
| CEDG146 | CEDG118 | 0.01861657 | 0.57906978 | 0 |
| CEDG225 | J01263 | 0.01710549 | 0.66297179 | 0 |
| CEDG225 | PV-at001 | 0.0131343 | 0.58590045 | 0.02754821 |
| CEDG225 | PV-ag003 | 0.01316276 | 0.55451926 | 0.18181818 |
| CEDG225 | PV-ag005 | 0.01785204 | 0.63566554 | 0.001998 |
| CEDG225 | Pv-ctt002 | 0.01213339 | 0.66334138 | 0.25641026 |
| CEDG225 | SSR-IAC 127 | 0.01258178 | 0.57640524 | 0.0862069 |
| CEDG225 | SSR-IAC 188 | 0.01142843 | 0.65774041 | 0.25641026 |
| CEDG225 | SSR-IAC 195 | 0.01213568 | 0.51296475 | 0.12195122 |
| CEDG225 | DQ469392 | 0.01210634 | 0.64817775 | 0.35714286 |
| CEDG225 | DQ469393 | 0.01324456 | 0.59027819 | 0.01692047 |
| CEDG225 | PvM03 | 0.01457658 | 0.71474566 | 0 |
| CEDG225 | PvM22 | 0.00960096 | 0.51936729 | 0.33333333 |
| CEDG225 | BMD-5 | 0.01686493 | 0.60322941 | 0 |
| CEDG225 | BMD-6 | 0.01559357 | 0.4630952 | 0.02375297 |
| CEDG225 | BMD-8 | 0.0141169 | 0.58232471 | 0.000999 |
| CEDG225 | BMD-13 | 0.01741916 | 0.75699407 | 0 |
| CEDG225 | BMD-18 | 0.01661205 | 0.68579282 | 0 |
| CEDG225 | BMD-23 | 0.0150982 | 0.62381168 | 0.01730104 |
| CEDG225 | BMD-26 | 0.01411434 | 0.66498898 | 0 |
| CEDG225 | BMD-29 | 0.01479323 | 0.59386764 | 0.04366812 |
| CEDG225 | BMD-31 | 0.0164996 | 0.60952206 | 0.001998 |
| CEDG225 | BMD-48 | 0.01347405 | 0.55683262 | 0.002997 |
| CEDG225 | BMD-35 | 0.01216262 | 0.52787636 | 0.01438849 |
| CEDG225 | BMD-47 | 0.0114334 | 0.61498503 | 0.25 |
| CEDG225 | BMD-50 | 0.01816935 | 0.57358834 | 0.000999 |
| CEDG225 | BMD-51 | 0.01439089 | 0.6001714 | 0.01748252 |
| CEDG225 | BMD-12 | 0.01368767 | 0.56745018 | 0.04504505 |
| CEDG225 | X21 | 0.01629598 | 0.64481594 | 0 |
| CEDG225 | X 34 | 0.01056498 | 0.59133721 | 0.32258065 |
| CEDG225 | X40 | 0.01460859 | 0.54403839 | 0.06896552 |
| CEDG225 | X49 | 0.00855652 | 0.46957719 | 0.1754386 |
| CEDG225 | X62 | 0.01193545 | 0.53553235 | 0.10869565 |
| CEDG225 | X65 | 0.01528618 | 0.58762053 | 0.001998 |
| CEDG225 | X87 | 0.01375969 | 0.73662252 | 0.002997 |
| CEDG225 | VR011 | 0.01196384 | 0.60732293 | 0.08547009 |
| CEDG225 | VR013 | 0.00876126 | 0.51954232 | 0.71428571 |
| CEDG225 | VR015 | 0.0136137 | 0.5307166 | 0.08928571 |
| CEDG225 | VR016 | 0.0141585 | 0.64124844 | 0.002997 |
| CEDG225 | VR018 | 0.01507162 | 0.66706757 | 0.000999 |
| CEDG225 | VR022 | 0.01414718 | 0.59405369 | 0.000999 |
| CEDG225 | VR022 | 0.01505668 | 0.71075561 | 0 |
| CEDG225 | VR024 | 0.01126615 | 0.44352402 | 0.5 |
| CEDG225 | VR025 | 0.01938704 | 0.6200157 | 0 |
| CEDG225 | VR032 | 0.01278082 | 0.56915511 | 0.078125 |
| CEDG225 | VR033 | 0.01273602 | 0.56095832 | 0.004995 |
| CEDG225 | VR035 | 0.01400822 | 0.56398574 | 0.01044932 |
| CEDG225 | VR037 | 0.01422876 | 0.63460808 | 0.000999 |
| CEDG225 | VR039 | 0.01559623 | 0.63588227 | 0 |
| CEDG225 | VR048 | 0.01431651 | 0.56379652 | 0 |
| CEDG225 | DQ345305 | 0.01319728 | 0.62328142 | 0.06666667 |
| CEDG225 | CEDG036 | 0.01579341 | 0.5302113 | 0.001998 |
| CEDG225 | CEDG291 | 0.02159817 | 0.70565354 | 0 |
| CEDG225 | CEDG220 | 0.01718097 | 0.65228366 | 0 |
| CEDG225 | CEDG136 | 0.01324764 | 0.63752004 | 0.0101833 |
| CEDG225 | CEDG100 | 0.01235513 | 0.66682768 | 0.01612903 |
| CEDG225 | CEDG096A | 0.01604973 | 0.74812263 | 0 |
| CEDG225 | CP00361 | 0.01833687 | 0.65365834 | 0 |
| CEDG225 | DMBSSR035 | 0.01515337 | 0.65601973 | 0 |
| CEDG225 | CEDG084 | 0.01856279 | 0.64921551 | 0 |
| CEDG225 | CEDG185 | 0.01832938 | 0.72789144 | 0 |
| CEDG225 | CEDC033 | 0.01620611 | 0.67841484 | 0 |
| CEDG225 | DMBSSR199 | 0.01775704 | 0.5799048 | 0 |
| CEDG225 | DMBSSR024 | 0.01420675 | 0.62649261 | 0.000999 |
| CEDG225 | GMES0337 | 0.01348896 | 0.57729357 | 0.00899101 |
| CEDG225 | DMBSSR001 | 0.01514351 | 0.59172907 | 0 |
| CEDG225 | CEDG118 | 0.01768094 | 0.65864999 | 0 |
| CEDG225 | CEDG146 | 0.01571334 | 0.59753964 | 0.001998 |
| JMES1424 | J01263 | 0.01694235 | 0.61633999 | 0 |
| JMES1424 | PV-at001 | 0.01392175 | 0.50707952 | 0.19607843 |
| JMES1424 | PV-ag003 | 0.01959892 | 0.54687708 | 0.000999 |
| JMES1424 | PV-ag005 | 0.01455667 | 0.56756843 | 0.04444444 |
| JMES1424 | Pv-ctt002 | 0.01332143 | 0.62670481 | 0.10989011 |
| JMES1424 | SSR-IAC 127 | 0.01201614 | 0.52231152 | 0.09708738 |
| JMES1424 | SSR-IAC 188 | 0.01111512 | 0.58059936 | 0.5 |
| JMES1424 | SSR-IAC 195 | 0.0158633 | 0.51640471 | 0.002997 |
| JMES1424 | DQ469392 | 0.01505742 | 0.60355919 | 0.03205128 |
| JMES1424 | DQ469393 | 0.01019025 | 0.45798439 | 0.52631579 |
| JMES1424 | PvM03 | 0.01102449 | 0.62459955 | 0.06535948 |
| JMES1424 | PvM22 | 0.0116777 | 0.43535996 | 0.14285714 |
| JMES1424 | BMD-5 | 0.01805944 | 0.51063171 | 0.000999 |
| JMES1424 | BMD-6 | 0.00826455 | 0.3072388 | 0.47619048 |
| JMES1424 | BMD-8 | 0.01977477 | 0.554376 | 0 |
| JMES1424 | BMD-13 | 0.01785507 | 0.72183291 | 0 |
| JMES1424 | BMD-18 | 0.02726716 | 0.67182514 | 0 |
| JMES1424 | BMD-23 | 0.01188384 | 0.48845397 | 0.27027027 |
| JMES1424 | BMD-26 | 0.0184508 | 0.66228769 | 0 |
| JMES1424 | BMD-29 | 0.01543658 | 0.5499477 | 0.003996 |
| JMES1424 | BMD-31 | 0.01632767 | 0.5445507 | 0.003996 |
| JMES1424 | BMD-48 | 0.01709795 | 0.49717 | 0 |
| JMES1424 | BMD-35 | 0.01534053 | 0.47148662 | 0.03154574 |
| JMES1424 | BMD-47 | 0.01303143 | 0.55524635 | 0.03333333 |
| JMES1424 | BMD-50 | 0.0267721 | 0.50704523 | 0 |
| JMES1424 | BMD-51 | 0.01885618 | 0.56214725 | 0.002997 |
| JMES1424 | BMD-12 | 0.01514331 | 0.47544893 | 0.01897533 |
| JMES1424 | X21 | 0.01164275 | 0.52085361 | 0.05128205 |
| JMES1424 | X 34 | 0.01126657 | 0.52433963 | 0.3030303 |
| JMES1424 | X40 | 0.01364144 | 0.48621275 | 0.09433962 |
| JMES1424 | X49 | 0.01591871 | 0.49315959 | 0.01321004 |
| JMES1424 | X62 | 0.02317716 | 0.52284275 | 0.002997 |
| JMES1424 | X65 | 0.01392467 | 0.50509132 | 0.07092199 |
| JMES1424 | X87 | 0.01173486 | 0.64085385 | 0.34482759 |
| JMES1424 | VR011 | 0.01413762 | 0.5987757 | 0.01615509 |
| JMES1424 | VR013 | 0.01261698 | 0.50956517 | 0.27777778 |
| JMES1424 | VR015 | 0.01664772 | 0.47299294 | 0.06896552 |
| JMES1424 | VR016 | 0.01128943 | 0.5533006 | 0.10309278 |
| JMES1424 | VR018 | 0.01532962 | 0.65670486 | 0.04098361 |
| JMES1424 | VR022 | 0.01208078 | 0.51423062 | 0.12987013 |
| JMES1424 | VR022 | 0.01580018 | 0.63891622 | 0.001998 |
| JMES1424 | VR024 | 0.0138876 | 0.43095607 | 0.52631579 |
| JMES1424 | VR025 | 0.0114043 | 0.4629038 | 0.2173913 |
| JMES1424 | VR032 | 0.01107038 | 0.47630742 | 0.45454545 |
| JMES1424 | VR033 | 0.01408766 | 0.48474396 | 0.00899101 |
| JMES1424 | VR035 | 0.02011315 | 0.52761689 | 0.001998 |
| JMES1424 | VR037 | 0.01312822 | 0.58103233 | 0.04484305 |
| JMES1424 | VR039 | 0.01257144 | 0.52182552 | 0.00599401 |
| JMES1424 | VR048 | 0.02554409 | 0.57466156 | 0 |
| JMES1424 | DQ345305 | 0.01316733 | 0.55614979 | 0.13157895 |
| JMES1424 | CEDG036 | 0.01034486 | 0.42642135 | 0.28571429 |
| JMES1424 | CEDG291 | 0.01321378 | 0.59645096 | 0.001998 |
| JMES1424 | CEDG220 | 0.02064642 | 0.59701081 | 0 |
| JMES1424 | CEDG136 | 0.01585758 | 0.57348793 | 0 |
| JMES1424 | CEDG100 | 0.01591792 | 0.65926664 | 0.001998 |
| JMES1424 | CEDG096A | 0.02196962 | 0.74490382 | 0 |
| JMES1424 | CP00361 | 0.02043775 | 0.61797001 | 0 |
| JMES1424 | DMBSSR035 | 0.01784095 | 0.61027024 | 0.00699301 |
| JMES1424 | CEDG084 | 0.01063332 | 0.49739751 | 0.3030303 |
| JMES1424 | CEDG185 | 0.02065965 | 0.69266704 | 0 |
| JMES1424 | CEDC033 | 0.01496518 | 0.63066976 | 0 |
| JMES1424 | DMBSSR199 | 0.01716064 | 0.52386567 | 0.03257329 |
| JMES1424 | DMBSSR024 | 0.01226029 | 0.54259608 | 0.01692047 |
| JMES1424 | GMES0337 | 0.01329848 | 0.50873182 | 0.003996 |
| JMES1424 | DMBSSR001 | 0.01933826 | 0.56622243 | 0 |
| JMES1424 | CEDG118 | 0.01553854 | 0.54154497 | 0.01017294 |
| JMES1424 | CEDG146 | 0.02962117 | 0.62424248 | 0 |
| JMES1424 | CEDG225 | 0.01866324 | 0.59266738 | 0 |
| MBSSR008 | J01263 | 0.01349542 | 0.62451902 | 0.00599401 |
| MBSSR008 | PV-at001 | 0.01143692 | 0.53888507 | 0.25 |
| MBSSR008 | PV-ag003 | 0.01748065 | 0.57252299 | 0.01180638 |
| MBSSR008 | PV-ag005 | 0.01949841 | 0.64237227 | 0 |
| MBSSR008 | Pv-ctt002 | 0.01187104 | 0.64705719 | 0.1010101 |
| MBSSR008 | SSR-IAC 127 | 0.01010012 | 0.51611576 | 0.52631579 |
| MBSSR008 | SSR-IAC 188 | 0.01107026 | 0.63731508 | 0.05154639 |
| MBSSR008 | SSR-IAC 195 | 0.01122963 | 0.48617013 | 0.38461538 |
| MBSSR008 | DQ469392 | 0.01042581 | 0.58113503 | 0.32258065 |
| MBSSR008 | DQ469393 | 0.01156888 | 0.55010997 | 0.0243309 |
| MBSSR008 | PvM03 | 0.01236591 | 0.69623499 | 0.09433962 |
| MBSSR008 | PvM22 | 0.0132673 | 0.52600416 | 0.01083424 |
| MBSSR008 | BMD-5 | 0.01048232 | 0.49845443 | 0.13513514 |
| MBSSR008 | BMD-6 | 0.00809101 | 0.31676895 | 0.5 |
| MBSSR008 | BMD-8 | 0.0152108 | 0.56770918 | 0.002997 |
| MBSSR008 | BMD-13 | 0.01512459 | 0.72684838 | 0 |
| MBSSR008 | BMD-18 | 0.01777478 | 0.63704438 | 0 |
| MBSSR008 | BMD-23 | 0.01397952 | 0.56950782 | 0.01199041 |
| MBSSR008 | BMD-26 | 0.01187458 | 0.60766509 | 0.03174603 |
| MBSSR008 | BMD-29 | 0.01646082 | 0.59562785 | 0.01230012 |
| MBSSR008 | BMD-31 | 0.01146484 | 0.52465984 | 0.18181818 |
| MBSSR008 | BMD-48 | 0.01327023 | 0.50599149 | 0.02475248 |
| MBSSR008 | BMD-35 | 0.01361798 | 0.49618723 | 0.05076142 |
| MBSSR008 | BMD-47 | 0.01128523 | 0.58012088 | 0.11235955 |
| MBSSR008 | BMD-50 | 0.01505697 | 0.52175931 | 0.03584229 |
| MBSSR008 | BMD-51 | 0.0158374 | 0.57490519 | 0.001998 |
| MBSSR008 | BMD-12 | 0.01025523 | 0.48357233 | 0.41666667 |
| MBSSR008 | X21 | 0.01666591 | 0.61495137 | 0.00699301 |
| MBSSR008 | X 34 | 0.01269328 | 0.61028064 | 0.04081633 |
| MBSSR008 | X40 | 0.01315893 | 0.52940545 | 0.0304878 |
| MBSSR008 | X49 | 0.00980558 | 0.47139236 | 0.43478261 |
| MBSSR008 | X62 | 0.01144346 | 0.53972924 | 0.18867925 |
| MBSSR008 | X65 | 0.01202915 | 0.52402294 | 0.02949853 |
| MBSSR008 | X87 | 0.01320557 | 0.70305383 | 0.01851852 |
| MBSSR008 | VR011 | 0.01694386 | 0.67537474 | 0.00799201 |
| MBSSR008 | VR013 | 0.0105726 | 0.54391047 | 0.22222222 |
| MBSSR008 | VR015 | 0.01150229 | 0.47029045 | 0.15625 |
| MBSSR008 | VR016 | 0.01227831 | 0.60070669 | 0.0273224 |
| MBSSR008 | VR018 | 0.01262446 | 0.66670974 | 0.000999 |
| MBSSR008 | VR022 | 0.00977467 | 0.52409634 | 0.45454545 |
| MBSSR008 | VR022 | 0.01251622 | 0.65079125 | 0.08474576 |
| MBSSR008 | VR024 | 0.00938963 | 0.43561172 | 0.45454545 |
| MBSSR008 | VR025 | 0.01642696 | 0.55787751 | 0.000999 |
| MBSSR008 | VR032 | 0.00987093 | 0.48992308 | 0.35714286 |
| MBSSR008 | VR033 | 0.01411164 | 0.52582522 | 0.003996 |
| MBSSR008 | VR035 | 0.0152612 | 0.54290057 | 0.06060606 |
| MBSSR008 | VR037 | 0.01502151 | 0.63317863 | 0.000999 |
| MBSSR008 | VR039 | 0.01712616 | 0.6052154 | 0.000999 |
| MBSSR008 | VR048 | 0.01197569 | 0.51268402 | 0.11627907 |
| MBSSR008 | DQ345305 | 0.01333421 | 0.61919431 | 0.0297619 |
| MBSSR008 | CEDG036 | 0.01703884 | 0.5512179 | 0 |
| MBSSR008 | CEDG291 | 0.01410435 | 0.63786102 | 0.000999 |
| MBSSR008 | CEDG220 | 0.02090914 | 0.65506452 | 0 |
| MBSSR008 | CEDG136 | 0.01097053 | 0.56482881 | 0.33333333 |
| MBSSR008 | CEDG100 | 0.01393626 | 0.6639746 | 0.00699301 |
| MBSSR008 | CEDG096A | 0.01228391 | 0.67370076 | 0.01061571 |
| MBSSR008 | CP00361 | 0.02185516 | 0.66762149 | 0 |
| MBSSR008 | DMBSSR035 | 0.01519656 | 0.63507418 | 0 |
| MBSSR008 | CEDG084 | 0.01180174 | 0.54909471 | 0.06410256 |
| MBSSR008 | CEDG185 | 0.01574962 | 0.67549964 | 0 |
| MBSSR008 | CEDC033 | 0.01253773 | 0.61089359 | 0.0154321 |
| MBSSR008 | DMBSSR199 | 0.01452425 | 0.54595436 | 0.12195122 |
| MBSSR008 | DMBSSR024 | 0.01492162 | 0.63405033 | 0.000999 |
| MBSSR008 | GMES0337 | 0.01121102 | 0.52055575 | 0.16129032 |
| MBSSR008 | DMBSSR001 | 0.01750368 | 0.57218687 | 0 |
| MBSSR008 | CEDG118 | 0.01303942 | 0.56218793 | 0.06410256 |
| MBSSR008 | CEDG146 | 0.02199945 | 0.63556051 | 0 |
| MBSSR008 | CEDG225 | 0.01240432 | 0.61907493 | 0.002997 |
| MBSSR008 | JMES1424 | 0.0194987 | 0.64756138 | 0 |
| CEDG271 | J01263 | 0.0163305 | 0.6794283 | 0 |
| CEDG271 | PV-at001 | 0.01157819 | 0.56604373 | 0.28571429 |
| CEDG271 | PV-ag003 | 0.0155905 | 0.56781929 | 0.01466276 |
| CEDG271 | PV-ag005 | 0.02115684 | 0.64583738 | 0.00799201 |
| CEDG271 | Pv-ctt002 | 0.0145199 | 0.70933745 | 0.000999 |
| CEDG271 | SSR-IAC 127 | 0.01169611 | 0.57300032 | 0.02570694 |
| CEDG271 | SSR-IAC 188 | 0.01222224 | 0.66514494 | 0.05617978 |
| CEDG271 | SSR-IAC 195 | 0.01436011 | 0.52773145 | 0.18518519 |
| CEDG271 | DQ469392 | 0.01276456 | 0.63447623 | 0.0877193 |
| CEDG271 | DQ469393 | 0.01319461 | 0.57947768 | 0.09803922 |
| CEDG271 | PvM03 | 0.01314121 | 0.70873201 | 0.27027027 |
| CEDG271 | PvM22 | 0.01158627 | 0.53041473 | 0.08547009 |
| CEDG271 | BMD-5 | 0.02241466 | 0.6188985 | 0 |
| CEDG271 | BMD-6 | 0.02002612 | 0.49702764 | 0.000999 |
| CEDG271 | BMD-8 | 0.01951756 | 0.62267869 | 0 |
| CEDG271 | BMD-13 | 0.01854538 | 0.73577418 | 0 |
| CEDG271 | BMD-18 | 0.0217443 | 0.72224781 | 0 |
| CEDG271 | BMD-23 | 0.01531024 | 0.56895359 | 0.00799201 |
| CEDG271 | BMD-26 | 0.01592491 | 0.66632049 | 0.01466276 |
| CEDG271 | BMD-29 | 0.01957396 | 0.61699637 | 0.00599401 |
| CEDG271 | BMD-31 | 0.01628983 | 0.59803159 | 0 |
| CEDG271 | BMD-48 | 0.01381522 | 0.51435031 | 0.38461538 |
| CEDG271 | BMD-35 | 0.01633662 | 0.56373124 | 0.00599401 |
| CEDG271 | BMD-47 | 0.01018085 | 0.58496211 | 0.71428571 |
| CEDG271 | BMD-50 | 0.01805037 | 0.59004264 | 0 |
| CEDG271 | BMD-51 | 0.01972601 | 0.64845542 | 0 |
| CEDG271 | BMD-12 | 0.01375505 | 0.54444277 | 0.06024096 |
| CEDG271 | X21 | 0.01186048 | 0.59266364 | 0.3030303 |
| CEDG271 | X 34 | 0.01255832 | 0.61839695 | 0.01727116 |
| CEDG271 | X40 | 0.01324086 | 0.54584581 | 0.02840909 |
| CEDG271 | X49 | 0.01388487 | 0.56847037 | 0.09708738 |
| CEDG271 | X62 | 0.01282994 | 0.52386331 | 0.76923077 |
| CEDG271 | X65 | 0.01318786 | 0.57700387 | 0.02267574 |
| CEDG271 | X87 | 0.0150073 | 0.75564544 | 0 |
| CEDG271 | VR011 | 0.02010468 | 0.67954958 | 0 |
| CEDG271 | VR013 | 0.01215428 | 0.58296715 | 0.03030303 |
| CEDG271 | VR015 | 0.01639826 | 0.54602791 | 0.000999 |
| CEDG271 | VR016 | 0.01566052 | 0.65549578 | 0.01336898 |
| CEDG271 | VR018 | 0.01433988 | 0.67866834 | 0.00799201 |
| CEDG271 | VR022 | 0.0136797 | 0.60625833 | 0.00799201 |
| CEDG271 | VR022 | 0.01482884 | 0.70701095 | 0.001998 |
| CEDG271 | VR024 | 0.02018803 | 0.56148304 | 0.003996 |
| CEDG271 | VR025 | 0.01771584 | 0.60052168 | 0.00799201 |
| CEDG271 | VR032 | 0.01551389 | 0.55468793 | 0.01841621 |
| CEDG271 | VR033 | 0.01187511 | 0.53310555 | 0.1754386 |
| CEDG271 | VR035 | 0.01219321 | 0.54784861 | 0.07874016 |
| CEDG271 | VR037 | 0.01456617 | 0.65675282 | 0.00799201 |
| CEDG271 | VR039 | 0.01481469 | 0.58816282 | 0.10752688 |
| CEDG271 | VR048 | 0.01524646 | 0.56687438 | 0.00599401 |
| CEDG271 | DQ345305 | 0.01046927 | 0.58278077 | 0.4 |
| CEDG271 | CEDG036 | 0.01514215 | 0.56454821 | 0 |
| CEDG271 | CEDG291 | 0.01325865 | 0.64561442 | 0.00599401 |
| CEDG271 | CEDG220 | 0.01889118 | 0.64553696 | 0.001998 |
| CEDG271 | CEDG136 | 0.01834919 | 0.65135285 | 0 |
| CEDG271 | CEDG100 | 0.01339508 | 0.6806374 | 0.001998 |
| CEDG271 | CEDG096A | 0.01365416 | 0.72684039 | 0.01022495 |
| CEDG271 | CP00361 | 0.02619177 | 0.70796836 | 0 |
| CEDG271 | DMBSSR035 | 0.02099715 | 0.73081184 | 0 |
| CEDG271 | CEDG084 | 0.01263414 | 0.56329714 | 0.04098361 |
| CEDG271 | CEDG185 | 0.01938063 | 0.69992785 | 0 |
| CEDG271 | CEDC033 | 0.01619632 | 0.69065237 | 0.00799201 |
| CEDG271 | DMBSSR199 | 0.01377742 | 0.52852996 | 0.18867925 |
| CEDG271 | DMBSSR024 | 0.01633021 | 0.64482651 | 0.00699301 |
| CEDG271 | GMES0337 | 0.01589755 | 0.58432183 | 0.002997 |
| CEDG271 | DMBSSR001 | 0.02079192 | 0.65534875 | 0 |
| CEDG271 | CEDG118 | 0.01968239 | 0.63123949 | 0.000999 |
| CEDG271 | CEDG146 | 0.01914945 | 0.66188283 | 0 |
| CEDG271 | CEDG225 | 0.01936227 | 0.69773889 | 0.000999 |
| CEDG271 | JMES1424 | 0.02120018 | 0.64733901 | 0.00599401 |
| CEDG271 | MBSSR008 | 0.01686194 | 0.65062285 | 0 |
| VM37 | J01263 | 0.01249515 | 0.62331695 | 0.02415459 |
| VM37 | PV-at001 | 0.01141152 | 0.52770988 | 0.20408163 |
| VM37 | PV-ag003 | 0.01235865 | 0.52527375 | 0.23255814 |
| VM37 | PV-ag005 | 0.00962025 | 0.51194465 | 0.66666667 |
| VM37 | Pv-ctt002 | 0.01309385 | 0.657761 | 0.004995 |
| VM37 | SSR-IAC 127 | 0.01055513 | 0.53012704 | 0.28571429 |
| VM37 | SSR-IAC 188 | 0.01148141 | 0.63299916 | 0.12195122 |
| VM37 | SSR-IAC 195 | 0.01079905 | 0.4474991 | 0.26315789 |
| VM37 | DQ469392 | 0.01153028 | 0.6128987 | 0.0729927 |
| VM37 | DQ469393 | 0.00939011 | 0.50524979 | 0.76923077 |
| VM37 | PvM03 | 0.01351015 | 0.69175712 | 0.03571429 |
| VM37 | PvM22 | 0.01350088 | 0.51144696 | 0.2173913 |
| VM37 | BMD-5 | 0.0108589 | 0.49060298 | 0.24390244 |
| VM37 | BMD-6 | 0.00998324 | 0.3898451 | 0.23255814 |
| VM37 | BMD-8 | 0.01286609 | 0.5149436 | 0.03571429 |
| VM37 | BMD-13 | 0.01246957 | 0.66967442 | 0.02096436 |
| VM37 | BMD-18 | 0.01576882 | 0.60872107 | 0.003996 |
| VM37 | BMD-23 | 0.01307257 | 0.53654956 | 0.26315789 |
| VM37 | BMD-26 | 0.0120621 | 0.60373952 | 0.01858736 |
| VM37 | BMD-29 | 0.01114209 | 0.51940908 | 0.1754386 |
| VM37 | BMD-31 | 0.01135659 | 0.51109723 | 0.0297619 |
| VM37 | BMD-48 | 0.01423227 | 0.48923128 | 0.33333333 |
| VM37 | BMD-35 | 0.01241909 | 0.49572976 | 0.03436426 |
| VM37 | BMD-47 | 0.01105167 | 0.56954597 | 0.625 |
| VM37 | BMD-50 | 0.01737904 | 0.51320196 | 0.001998 |
| VM37 | BMD-51 | 0.011522 | 0.53470039 | 0.08547009 |
| VM37 | BMD-12 | 0.0141008 | 0.52392412 | 0.07092199 |
| VM37 | X21 | 0.01110787 | 0.55180765 | 0.06369427 |
| VM37 | X 34 | 0.00942473 | 0.53830086 | 0.38461538 |
| VM37 | X40 | 0.01311898 | 0.53535604 | 0.00599401 |
| VM37 | X49 | 0.00829638 | 0.45585455 | 0.76923077 |
| VM37 | X62 | 0.01019622 | 0.48213751 | 0.2 |
| VM37 | X65 | 0.00860919 | 0.45875141 | 0.58823529 |
| VM37 | X87 | 0.01355544 | 0.69511018 | 0.0129199 |
| VM37 | VR011 | 0.01251862 | 0.58545768 | 0.18867925 |
| VM37 | VR013 | 0.01078385 | 0.53185555 | 0.19230769 |
| VM37 | VR015 | 0.01562856 | 0.51778227 | 0.001998 |
| VM37 | VR016 | 0.01034964 | 0.56961789 | 0.41666667 |
| VM37 | VR018 | 0.01135844 | 0.6166435 | 0.16949153 |
| VM37 | VR022 | 0.01194944 | 0.53700628 | 0.16393443 |
| VM37 | VR022 | 0.01155412 | 0.64344471 | 0.10416667 |
| VM37 | VR024 | 0.01581697 | 0.51331626 | 0.16393443 |
| VM37 | VR025 | 0.00896964 | 0.43799055 | 0.37037037 |
| VM37 | VR032 | 0.01010148 | 0.45322068 | 0.33333333 |
| VM37 | VR033 | 0.00842333 | 0.46616422 | 0.83333333 |
| VM37 | VR035 | 0.01495881 | 0.56443743 | 0.000999 |
| VM37 | VR037 | 0.01046092 | 0.56368401 | 0.15151515 |
| VM37 | VR039 | 0.01352496 | 0.56386266 | 0.001998 |
| VM37 | VR048 | 0.01067352 | 0.48118631 | 0.08130081 |
| VM37 | DQ345305 | 0.01336201 | 0.60858072 | 0.06944444 |
| VM37 | CEDG036 | 0.01580864 | 0.51715259 | 0.01680672 |
| VM37 | CEDG291 | 0.01954083 | 0.6734614 | 0 |
| VM37 | CEDG220 | 0.01149005 | 0.53233869 | 0.23255814 |
| VM37 | CEDG136 | 0.01562225 | 0.57154634 | 0.002997 |
| VM37 | CEDG100 | 0.01455466 | 0.69886699 | 0.000999 |
| VM37 | CEDG096A | 0.01394056 | 0.70679741 | 0.00899101 |
| VM37 | CP00361 | 0.01884502 | 0.60065177 | 0 |
| VM37 | DMBSSR035 | 0.01326213 | 0.61099105 | 0.00899101 |
| VM37 | CEDG084 | 0.01305556 | 0.53012383 | 0.01129944 |
| VM37 | CEDG185 | 0.01324433 | 0.63340206 | 0.02906977 |
| VM37 | CEDC033 | 0.01209891 | 0.59995413 | 0.14285714 |
| VM37 | DMBSSR199 | 0.01172205 | 0.48829225 | 0.29411765 |
| VM37 | DMBSSR024 | 0.01297362 | 0.58693924 | 0.004995 |
| VM37 | GMES0337 | 0.01305896 | 0.53548673 | 0.07751938 |
| VM37 | DMBSSR001 | 0.01409243 | 0.55195215 | 0.00599401 |
| VM37 | CEDG118 | 0.01169061 | 0.55586528 | 0.06493506 |
| VM37 | CEDG146 | 0.01494833 | 0.54497308 | 0 |
| VM37 | CEDG225 | 0.01483799 | 0.62737616 | 0.003996 |
| VM37 | JMES1424 | 0.01188497 | 0.53462767 | 0.07633588 |
| VM37 | MBSSR008 | 0.01229385 | 0.58053572 | 0.00699301 |
| VM37 | CEDG271 | 0.01309919 | 0.62948789 | 0.01239157 |
| CEDG073 | J01263 | 0.02068684 | 0.70226242 | 0 |
| CEDG073 | PV-at001 | 0.01258444 | 0.53872942 | 0.0173913 |
| CEDG073 | PV-ag003 | 0.01236344 | 0.52945869 | 0.09009009 |
| CEDG073 | PV-ag005 | 0.01855756 | 0.62961681 | 0 |
| CEDG073 | Pv-ctt002 | 0.01339801 | 0.69389089 | 0.000999 |
| CEDG073 | SSR-IAC 127 | 0.01165652 | 0.55444778 | 0.3125 |
| CEDG073 | SSR-IAC 188 | 0.01407544 | 0.67496839 | 0.03125 |
| CEDG073 | SSR-IAC 195 | 0.01811995 | 0.55659113 | 0 |
| CEDG073 | DQ469392 | 0.01270213 | 0.64772748 | 0.04878049 |
| CEDG073 | DQ469393 | 0.0159161 | 0.60060947 | 0 |
| CEDG073 | PvM03 | 0.01109962 | 0.67570427 | 0.3125 |
| CEDG073 | PvM22 | 0.01248002 | 0.53044604 | 0.14285714 |
| CEDG073 | BMD-5 | 0.01509951 | 0.55654759 | 0.00799201 |
| CEDG073 | BMD-6 | 0.01542644 | 0.4361977 | 0.02915452 |
| CEDG073 | BMD-8 | 0.01355338 | 0.53568307 | 0.000999 |
| CEDG073 | BMD-13 | 0.01413512 | 0.71317216 | 0.000999 |
| CEDG073 | BMD-18 | 0.01967195 | 0.67794145 | 0 |
| CEDG073 | BMD-23 | 0.01220457 | 0.55949858 | 0.06329114 |
| CEDG073 | BMD-26 | 0.01223719 | 0.63550254 | 0.05847953 |
| CEDG073 | BMD-29 | 0.0182865 | 0.64449145 | 0.000999 |
| CEDG073 | BMD-31 | 0.01581692 | 0.59680617 | 0.001998 |
| CEDG073 | BMD-48 | 0.01482099 | 0.52378314 | 0.01239157 |
| CEDG073 | BMD-35 | 0.01408066 | 0.50414748 | 0.00799201 |
| CEDG073 | BMD-47 | 0.01305488 | 0.60174462 | 0.02932551 |
| CEDG073 | BMD-50 | 0.01875585 | 0.5473958 | 0 |
| CEDG073 | BMD-51 | 0.01225099 | 0.54202176 | 0.01703578 |
| CEDG073 | BMD-12 | 0.01548365 | 0.56557421 | 0.001998 |
| CEDG073 | X21 | 0.01267597 | 0.58260565 | 0.00799201 |
| CEDG073 | X 34 | 0.01473887 | 0.61702078 | 0.00899101 |
| CEDG073 | X40 | 0.01325112 | 0.49802759 | 0.4 |
| CEDG073 | X49 | 0.0121117 | 0.5323796 | 0.01322751 |
| CEDG073 | X62 | 0.00950954 | 0.49533949 | 0.28571429 |
| CEDG073 | X65 | 0.01194497 | 0.53188569 | 0.37037037 |
| CEDG073 | X87 | 0.01666505 | 0.74330717 | 0 |
| CEDG073 | VR011 | 0.01582974 | 0.64989405 | 0 |
| CEDG073 | VR013 | 0.0122158 | 0.56269691 | 0.14492754 |
| CEDG073 | VR015 | 0.01563013 | 0.52702984 | 0.004995 |
| CEDG073 | VR016 | 0.01418139 | 0.62709148 | 0.001998 |
| CEDG073 | VR018 | 0.0139552 | 0.67372405 | 0.004995 |
| CEDG073 | VR022 | 0.01432228 | 0.59982448 | 0.001998 |
| CEDG073 | VR022 | 0.01300059 | 0.67474606 | 0.01869159 |
| CEDG073 | VR024 | 0.01132022 | 0.45059419 | 0.28571429 |
| CEDG073 | VR025 | 0.01317602 | 0.54544649 | 0.03246753 |
| CEDG073 | VR032 | 0.01107583 | 0.50555884 | 0.38461538 |
| CEDG073 | VR033 | 0.01233704 | 0.49861588 | 0.05102041 |
| CEDG073 | VR035 | 0.01279691 | 0.52113867 | 0.05464481 |
| CEDG073 | VR037 | 0.01554612 | 0.67674166 | 0 |
| CEDG073 | VR039 | 0.01435927 | 0.60750824 | 0 |
| CEDG073 | VR048 | 0.01685193 | 0.58590738 | 0 |
| CEDG073 | DQ345305 | 0.0159333 | 0.64139368 | 0.002997 |
| CEDG073 | CEDG036 | 0.01417558 | 0.4965898 | 0.14492754 |
| CEDG073 | CEDG291 | 0.01303852 | 0.62690077 | 0.01165501 |
| CEDG073 | CEDG220 | 0.01828211 | 0.6282002 | 0 |
| CEDG073 | CEDG136 | 0.01364945 | 0.58499011 | 0.04926108 |
| CEDG073 | CEDG100 | 0.0146081 | 0.69645496 | 0.01447178 |
| CEDG073 | CEDG096A | 0.01455673 | 0.72102959 | 0.000999 |
| CEDG073 | CP00361 | 0.02069672 | 0.66546126 | 0 |
| CEDG073 | DMBSSR035 | 0.01511018 | 0.65167886 | 0.000999 |
| CEDG073 | CEDG084 | 0.01270521 | 0.57478227 | 0.27777778 |
| CEDG073 | CEDG185 | 0.01487172 | 0.66210256 | 0.002997 |
| CEDG073 | CEDC033 | 0.01399997 | 0.65004039 | 0.001998 |
| CEDG073 | DMBSSR199 | 0.01457998 | 0.55609201 | 0.001998 |
| CEDG073 | DMBSSR024 | 0.01484225 | 0.61247766 | 0.00799201 |
| CEDG073 | GMES0337 | 0.01178725 | 0.55122115 | 0.23255814 |
| CEDG073 | DMBSSR001 | 0.01435918 | 0.55247278 | 0.00699301 |
| CEDG073 | CEDG118 | 0.01318554 | 0.5592246 | 0.12987013 |
| CEDG073 | CEDG146 | 0.01751856 | 0.60022719 | 0 |
| CEDG073 | CEDG225 | 0.01697957 | 0.67277687 | 0 |
| CEDG073 | JMES1424 | 0.01357143 | 0.58979963 | 0.00599401 |
| CEDG073 | MBSSR008 | 0.02009348 | 0.67749536 | 0 |
| CEDG073 | CEDG271 | 0.02023329 | 0.71368638 | 0 |
| CEDG073 | VM37 | 0.01446547 | 0.61176329 | 0.003996 |
| CEDG071 | J01263 | 0.0117809 | 0.60733047 | 0.13157895 |
| CEDG071 | PV-at001 | 0.01187113 | 0.56330152 | 0.12345679 |
| CEDG071 | PV-ag003 | 0.01372453 | 0.53788005 | 0.03472222 |
| CEDG071 | PV-ag005 | 0.0133986 | 0.58347485 | 0.10752688 |
| CEDG071 | Pv-ctt002 | 0.01089319 | 0.63540668 | 0.33333333 |
| CEDG071 | SSR-IAC 127 | 0.01276159 | 0.5496242 | 0.10989011 |
| CEDG071 | SSR-IAC 188 | 0.01332669 | 0.67038055 | 0.01270648 |
| CEDG071 | SSR-IAC 195 | 0.01070332 | 0.46237127 | 0.29411765 |
| CEDG071 | DQ469392 | 0.01236241 | 0.63015189 | 0.10638298 |
| CEDG071 | DQ469393 | 0.01437292 | 0.55821463 | 0.00999001 |
| CEDG071 | PvM03 | 0.0140322 | 0.68279718 | 0.016 |
| CEDG071 | PvM22 | 0.0086107 | 0.44585655 | 0.52631579 |
| CEDG071 | BMD-5 | 0.01226406 | 0.52320979 | 0.03921569 |
| CEDG071 | BMD-6 | 0.01065489 | 0.38849173 | 0.45454545 |
| CEDG071 | BMD-8 | 0.01324048 | 0.53550218 | 0.07936508 |
| CEDG071 | BMD-13 | 0.01144173 | 0.67339911 | 0.03802281 |
| CEDG071 | BMD-18 | 0.01279642 | 0.59552444 | 0.00799201 |
| CEDG071 | BMD-23 | 0.01099698 | 0.52280459 | 0.5 |
| CEDG071 | BMD-26 | 0.01035247 | 0.59513719 | 0.08474576 |
| CEDG071 | BMD-29 | 0.01222332 | 0.57233514 | 0.05882353 |
| CEDG071 | BMD-31 | 0.01180229 | 0.51195786 | 0.02583979 |
| CEDG071 | BMD-48 | 0.01433759 | 0.52270424 | 0.001998 |
| CEDG071 | BMD-35 | 0.00661903 | 0.39143278 | 0.90909091 |
| CEDG071 | BMD-47 | 0.01174333 | 0.57592516 | 0.47619048 |
| CEDG071 | BMD-50 | 0.01145688 | 0.44326629 | 0.22727273 |
| CEDG071 | BMD-51 | 0.01362371 | 0.55702546 | 0.03623188 |
| CEDG071 | BMD-12 | 0.01756404 | 0.5380774 | 0.002997 |
| CEDG071 | X21 | 0.01352052 | 0.575501 | 0.02985075 |
| CEDG071 | X 34 | 0.01137861 | 0.57478948 | 0.47619048 |
| CEDG071 | X40 | 0.01367341 | 0.52772863 | 0.04784689 |
| CEDG071 | X49 | 0.01596927 | 0.53381862 | 0.01013171 |
| CEDG071 | X62 | 0.01383898 | 0.52906471 | 0.14084507 |
| CEDG071 | X65 | 0.01970649 | 0.56683032 | 0 |
| CEDG071 | X87 | 0.00994771 | 0.64750768 | 0.52631579 |
| CEDG071 | VR011 | 0.01283428 | 0.6489363 | 0.04608295 |
| CEDG071 | VR013 | 0.00898147 | 0.48776664 | 0.76923077 |
| CEDG071 | VR015 | 0.01569243 | 0.51400462 | 0.001998 |
| CEDG071 | VR016 | 0.01136539 | 0.58618522 | 0.16666667 |
| CEDG071 | VR018 | 0.01559467 | 0.67302623 | 0.01083424 |
| CEDG071 | VR022 | 0.01115816 | 0.51513808 | 0.09259259 |
| CEDG071 | VR022 | 0.01167632 | 0.65141609 | 0.04032258 |
| CEDG071 | VR024 | 0.01074834 | 0.42578402 | 0.66666667 |
| CEDG071 | VR025 | 0.01298736 | 0.502894 | 0.04784689 |
| CEDG071 | VR032 | 0.01200233 | 0.52476312 | 0.0330033 |
| CEDG071 | VR033 | 0.01401769 | 0.54756059 | 0.01422475 |
| CEDG071 | VR035 | 0.01309089 | 0.53663536 | 0.03690037 |
| CEDG071 | VR037 | 0.01219929 | 0.59637386 | 0.07936508 |
| CEDG071 | VR039 | 0.01157896 | 0.54444366 | 0.06329114 |
| CEDG071 | VR048 | 0.01086169 | 0.47709128 | 0.14084507 |
| CEDG071 | DQ345305 | 0.0129337 | 0.61909447 | 0.05154639 |
| CEDG071 | CEDG036 | 0.00865306 | 0.41359065 | 0.76923077 |
| CEDG071 | CEDG291 | 0.01567872 | 0.65695419 | 0.000999 |
| CEDG071 | CEDG220 | 0.01450922 | 0.56776326 | 0.03225806 |
| CEDG071 | CEDG136 | 0.01079597 | 0.56034238 | 0.23255814 |
| CEDG071 | CEDG100 | 0.01550488 | 0.65819709 | 0.000999 |
| CEDG071 | CEDG096A | 0.01179843 | 0.68036214 | 0.01293661 |
| CEDG071 | CP00361 | 0.01487293 | 0.61580532 | 0 |
| CEDG071 | DMBSSR035 | 0.01516875 | 0.6574775 | 0.001998 |
| CEDG071 | CEDG084 | 0.01175546 | 0.51486 | 0.26315789 |
| CEDG071 | CEDG185 | 0.01676109 | 0.65379752 | 0.000999 |
| CEDG071 | CEDC033 | 0.0138283 | 0.62867293 | 0.001998 |
| CEDG071 | DMBSSR199 | 0.01156515 | 0.49754388 | 0.35714286 |
| CEDG071 | DMBSSR024 | 0.01503473 | 0.62312625 | 0.001998 |
| CEDG071 | GMES0337 | 0.01328011 | 0.56399067 | 0.000999 |
| CEDG071 | DMBSSR001 | 0.01393008 | 0.53840381 | 0.000999 |
| CEDG071 | CEDG118 | 0.01695818 | 0.6192327 | 0 |
| CEDG071 | CEDG146 | 0.0099924 | 0.49332509 | 0.58823529 |
| CEDG071 | CEDG225 | 0.01445221 | 0.60757709 | 0.000999 |
| CEDG071 | JMES1424 | 0.0183962 | 0.60547284 | 0 |
| CEDG071 | MBSSR008 | 0.01054752 | 0.566548 | 0.10638298 |
| CEDG071 | CEDG271 | 0.01247101 | 0.59242928 | 0.2 |
| CEDG071 | VM37 | 0.01025815 | 0.53879481 | 0.55555556 |
| CEDG071 | CEDG073 | 0.0133505 | 0.60687683 | 0.01519757 |
| CP00226 | J01263 | 0.01319744 | 0.76808913 | 0.03745318 |
| CP00226 | PV-at001 | 0.01347223 | 0.70009451 | 0.02923977 |
| CP00226 | PV-ag003 | 0.01291888 | 0.67225463 | 0.001998 |
| CP00226 | PV-ag005 | 0.01282242 | 0.72081233 | 0.01148106 |
| CP00226 | Pv-ctt002 | 0.01101128 | 0.76041109 | 0.71428571 |
| CP00226 | SSR-IAC 127 | 0.01151619 | 0.6883984 | 0.16393443 |
| CP00226 | SSR-IAC 188 | 0.01098462 | 0.73863379 | 0.06134969 |
| CP00226 | SSR-IAC 195 | 0.01479705 | 0.65997469 | 0.00699301 |
| CP00226 | DQ469392 | 0.01444016 | 0.76211398 | 0 |
| CP00226 | DQ469393 | 0.01439287 | 0.7076924 | 0.004995 |
| CP00226 | PvM03 | 0.0110649 | 0.78278745 | 0.09708738 |
| CP00226 | PvM22 | 0.01102304 | 0.63558462 | 0.18181818 |
| CP00226 | BMD-5 | 0.018418 | 0.67824858 | 0 |
| CP00226 | BMD-6 | 0.01301926 | 0.53891731 | 0.14492754 |
| CP00226 | BMD-8 | 0.01317492 | 0.69111392 | 0.13157895 |
| CP00226 | BMD-13 | 0.01192476 | 0.77957366 | 0.04504505 |
| CP00226 | BMD-18 | 0.01628419 | 0.78595319 | 0 |
| CP00226 | BMD-23 | 0.01055489 | 0.65108765 | 0.20833333 |
| CP00226 | BMD-26 | 0.00972979 | 0.67004393 | 0.90909091 |
| CP00226 | BMD-29 | 0.01022217 | 0.66111574 | 0.35714286 |
| CP00226 | BMD-31 | 0.01503649 | 0.69356803 | 0.003996 |
| CP00226 | BMD-48 | 0.01229115 | 0.64067526 | 0.01779359 |
| CP00226 | BMD-35 | 0.01093196 | 0.62837031 | 0.3030303 |
| CP00226 | BMD-47 | 0.01477806 | 0.74950386 | 0.001998 |
| CP00226 | BMD-50 | 0.0138097 | 0.62358869 | 0.26315789 |
| CP00226 | BMD-51 | 0.01500546 | 0.735719 | 0.000999 |
| CP00226 | BMD-12 | 0.01202843 | 0.61856067 | 0.16129032 |
| CP00226 | X21 | 0.01272866 | 0.69796667 | 0.35714286 |
| CP00226 | X 34 | 0.01108569 | 0.68259831 | 0.3125 |
| CP00226 | X40 | 0.00983865 | 0.58534881 | 0.76923077 |
| CP00226 | X49 | 0.01101944 | 0.6231882 | 0.5 |
| CP00226 | X62 | 0.01242466 | 0.65818551 | 0.33333333 |
| CP00226 | X65 | 0.01276874 | 0.66848701 | 0.45454545 |
| CP00226 | X87 | 0.01249542 | 0.79742239 | 0.01228501 |
| CP00226 | VR011 | 0.01147883 | 0.7227474 | 0.27027027 |
| CP00226 | VR013 | 0.01124933 | 0.66297735 | 0.38461538 |
| CP00226 | VR015 | 0.01144988 | 0.63280273 | 0.06849315 |
| CP00226 | VR016 | 0.01344597 | 0.7430944 | 0.16129032 |
| CP00226 | VR018 | 0.01243825 | 0.76286337 | 0.01221001 |
| CP00226 | VR022 | 0.01066777 | 0.66301717 | 0.1754386 |
| CP00226 | VR022 | 0.0105819 | 0.74650087 | 0.37037037 |
| CP00226 | VR024 | 0.01207685 | 0.58293786 | 0.0210084 |
| CP00226 | VR025 | 0.01151342 | 0.62674691 | 0.76923077 |
| CP00226 | VR032 | 0.01104778 | 0.62128344 | 0.3030303 |
| CP00226 | VR033 | 0.01164035 | 0.64437988 | 0.13513514 |
| CP00226 | VR035 | 0.01310009 | 0.67656195 | 0.05208333 |
| CP00226 | VR037 | 0.01241598 | 0.74153756 | 0.45454545 |
| CP00226 | VR039 | 0.01344357 | 0.70897287 | 0.04329004 |
| CP00226 | VR048 | 0.01207875 | 0.64394229 | 0.15873016 |
| CP00226 | DQ345305 | 0.01039067 | 0.70452166 | 0.18181818 |
| CP00226 | CEDG036 | 0.01153062 | 0.61799764 | 0.19230769 |
| CP00226 | CEDG291 | 0.01355219 | 0.75540085 | 0.004995 |
| CP00226 | CEDG220 | 0.01762622 | 0.76241633 | 0 |
| CP00226 | CEDG136 | 0.01594516 | 0.72344242 | 0.000999 |
| CP00226 | CEDG100 | 0.0118796 | 0.747205 | 0.0390625 |
| CP00226 | CEDG096A | 0.01403677 | 0.8338684 | 0 |
| CP00226 | CP00361 | 0.0119798 | 0.68947983 | 0.04032258 |
| CP00226 | DMBSSR035 | 0.01946897 | 0.8040715 | 0 |
| CP00226 | CEDG084 | 0.01203102 | 0.6729258 | 0.05681818 |
| CP00226 | CEDG185 | 0.01330034 | 0.75500792 | 0.04587156 |
| CP00226 | CEDC033 | 0.01176542 | 0.74258964 | 0.26315789 |
| CP00226 | DMBSSR199 | 0.01011959 | 0.59869954 | 0.18867925 |
| CP00226 | DMBSSR024 | 0.01583091 | 0.76644653 | 0 |
| CP00226 | GMES0337 | 0.01345484 | 0.70176238 | 0.01557632 |
| CP00226 | DMBSSR001 | 0.01470556 | 0.70384701 | 0.002997 |
| CP00226 | CEDG118 | 0.01443017 | 0.7462634 | 0.01560062 |
| CP00226 | CEDG146 | 0.01712264 | 0.73256931 | 0.000999 |
| CP00226 | CEDG225 | 0.01607823 | 0.77765364 | 0.000999 |
| CP00226 | JMES1424 | 0.01570109 | 0.74043831 | 0 |
| CP00226 | MBSSR008 | 0.01122881 | 0.69162752 | 0.2173913 |
| CP00226 | CEDG271 | 0.01675374 | 0.77290737 | 0 |
| CP00226 | VM37 | 0.01442845 | 0.73302495 | 0.00699301 |
| CP00226 | CEDG073 | 0.01231672 | 0.71528652 | 0.16129032 |
| CP00226 | CEDG071 | 0.01510504 | 0.76758612 | 0.003996 |
| DMBSSR059 | J01263 | 0.01246127 | 0.61202595 | 0.00699301 |
| DMBSSR059 | PV-at001 | 0.01022676 | 0.51160666 | 0.1369863 |
| DMBSSR059 | PV-ag003 | 0.01057906 | 0.46694862 | 0.3125 |
| DMBSSR059 | PV-ag005 | 0.0119986 | 0.53994023 | 0.5 |
| DMBSSR059 | Pv-ctt002 | 0.01278964 | 0.66451705 | 0.12048193 |
| DMBSSR059 | SSR-IAC 127 | 0.0115117 | 0.52097423 | 0.41666667 |
| DMBSSR059 | SSR-IAC 188 | 0.01082199 | 0.62006806 | 0.07936508 |
| DMBSSR059 | SSR-IAC 195 | 0.0112834 | 0.47423827 | 0.09174312 |
| DMBSSR059 | DQ469392 | 0.0108541 | 0.56520904 | 0.45454545 |
| DMBSSR059 | DQ469393 | 0.01398991 | 0.52828858 | 0.02469136 |
| DMBSSR059 | PvM03 | 0.01358058 | 0.69788111 | 0 |
| DMBSSR059 | PvM22 | 0.01025046 | 0.45958054 | 0.15384615 |
| DMBSSR059 | BMD-5 | 0.0126183 | 0.50276798 | 0.02057613 |
| DMBSSR059 | BMD-6 | 0.01173916 | 0.38253372 | 0.3030303 |
| DMBSSR059 | BMD-8 | 0.01483495 | 0.51480931 | 0.01303781 |
| DMBSSR059 | BMD-13 | 0.01336991 | 0.66882696 | 0.01477105 |
| DMBSSR059 | BMD-18 | 0.01315833 | 0.59650021 | 0 |
| DMBSSR059 | BMD-23 | 0.01437069 | 0.5277078 | 0.25 |
| DMBSSR059 | BMD-26 | 0.00994801 | 0.58004605 | 0.12345679 |
| DMBSSR059 | BMD-29 | 0.01200413 | 0.54329208 | 0.07092199 |
| DMBSSR059 | BMD-31 | 0.0112952 | 0.4938737 | 0.10989011 |
| DMBSSR059 | BMD-48 | 0.01040943 | 0.45006955 | 0.25641026 |
| DMBSSR059 | BMD-35 | 0.01753323 | 0.5222033 | 0.000999 |
| DMBSSR059 | BMD-47 | 0.0141473 | 0.60457401 | 0.12195122 |
| DMBSSR059 | BMD-50 | 0.01138613 | 0.44891872 | 0.08403361 |
| DMBSSR059 | BMD-51 | 0.01505099 | 0.5572709 | 0.05649718 |
| DMBSSR059 | BMD-12 | 0.01095484 | 0.47483115 | 0.3125 |
| DMBSSR059 | X21 | 0.01339653 | 0.5699729 | 0.01070664 |
| DMBSSR059 | X 34 | 0.0126254 | 0.57290178 | 0.13157895 |
| DMBSSR059 | X40 | 0.01254542 | 0.50414805 | 0.18181818 |
| DMBSSR059 | X49 | 0.01234676 | 0.50363263 | 0.27027027 |
| DMBSSR059 | X62 | 0.0119614 | 0.50586124 | 0.07633588 |
| DMBSSR059 | X65 | 0.02539026 | 0.61619402 | 0 |
| DMBSSR059 | X87 | 0.01432118 | 0.71357261 | 0.002997 |
| DMBSSR059 | VR011 | 0.01406134 | 0.6188701 | 0.11235955 |
| DMBSSR059 | VR013 | 0.01029398 | 0.5194975 | 0.28571429 |
| DMBSSR059 | VR015 | 0.01002819 | 0.42966032 | 0.18867925 |
| DMBSSR059 | VR016 | 0.01419114 | 0.5884969 | 0.001998 |
| DMBSSR059 | VR018 | 0.01369608 | 0.63742005 | 0.00799201 |
| DMBSSR059 | VR022 | 0.01286382 | 0.52932341 | 0.17241379 |
| DMBSSR059 | VR022 | 0.0115839 | 0.6222321 | 0.02192982 |
| DMBSSR059 | VR024 | 0.0087812 | 0.41164253 | 0.06944444 |
| DMBSSR059 | VR025 | 0.02149504 | 0.5983417 | 0 |
| DMBSSR059 | VR032 | 0.01174492 | 0.48911232 | 0.02024291 |
| DMBSSR059 | VR033 | 0.01136121 | 0.48470931 | 0.10309278 |
| DMBSSR059 | VR035 | 0.0125653 | 0.47494246 | 0.09615385 |
| DMBSSR059 | VR037 | 0.01190533 | 0.57246699 | 0.20833333 |
| DMBSSR059 | VR039 | 0.01370636 | 0.53786878 | 0.08196721 |
| DMBSSR059 | VR048 | 0.0115457 | 0.48376172 | 0.01709402 |
| DMBSSR059 | DQ345305 | 0.01218343 | 0.56153964 | 0.66666667 |
| DMBSSR059 | CEDG036 | 0.01326158 | 0.47498517 | 0.03921569 |
| DMBSSR059 | CEDG291 | 0.01406729 | 0.62471813 | 0.00599401 |
| DMBSSR059 | CEDG220 | 0.01069657 | 0.51708686 | 0.2173913 |
| DMBSSR059 | CEDG136 | 0.01130056 | 0.54331684 | 0.14084507 |
| DMBSSR059 | CEDG100 | 0.01622055 | 0.65694124 | 0.000999 |
| DMBSSR059 | CEDG096A | 0.01171917 | 0.65911463 | 0.0280112 |
| DMBSSR059 | CP00361 | 0.01638211 | 0.61530111 | 0.003996 |
| DMBSSR059 | DMBSSR035 | 0.01613747 | 0.63167883 | 0 |
| DMBSSR059 | CEDG084 | 0.01599044 | 0.54899986 | 0.01457726 |
| DMBSSR059 | CEDG185 | 0.02829476 | 0.67653019 | 0 |
| DMBSSR059 | CEDC033 | 0.01685941 | 0.6574444 | 0 |
| DMBSSR059 | DMBSSR199 | 0.0138915 | 0.5055629 | 0.04424779 |
| DMBSSR059 | DMBSSR024 | 0.01194265 | 0.53932587 | 0.0130039 |
| DMBSSR059 | GMES0337 | 0.01479223 | 0.55377835 | 0.00599401 |
| DMBSSR059 | DMBSSR001 | 0.01476424 | 0.55566121 | 0.000999 |
| DMBSSR059 | CEDG118 | 0.01434937 | 0.57594704 | 0.003996 |
| DMBSSR059 | CEDG146 | 0.01577784 | 0.56731508 | 0.001998 |
| DMBSSR059 | CEDG225 | 0.01632776 | 0.63538195 | 0 |
| DMBSSR059 | JMES1424 | 0.01134426 | 0.50113587 | 0.078125 |
| DMBSSR059 | MBSSR008 | 0.0127293 | 0.56006677 | 0.0148368 |
| DMBSSR059 | CEDG271 | 0.01614358 | 0.61579691 | 0.001998 |
| DMBSSR059 | VM37 | 0.0139699 | 0.56122931 | 0.04219409 |
| DMBSSR059 | CEDG073 | 0.0136548 | 0.58472827 | 0.05181347 |
| DMBSSR059 | CEDG071 | 0.01422142 | 0.5757982 | 0.004995 |
| DMBSSR059 | CP00226 | 0.0135417 | 0.71245721 | 0.15873016 |
| VM27 | J01263 | 0.01475182 | 0.68295429 | 0 |
| VM27 | PV-at001 | 0.01429566 | 0.61946673 | 0.01872659 |
| VM27 | PV-ag003 | 0.01453686 | 0.61242094 | 0.08064516 |
| VM27 | PV-ag005 | 0.01476695 | 0.62211527 | 0.11235955 |
| VM27 | Pv-ctt002 | 0.01260669 | 0.69641985 | 0.04132231 |
| VM27 | SSR-IAC 127 | 0.01155197 | 0.58232019 | 0.35714286 |
| VM27 | SSR-IAC 188 | 0.01321926 | 0.6900964 | 0.07092199 |
| VM27 | SSR-IAC 195 | 0.01074715 | 0.51791163 | 0.15151515 |
| VM27 | DQ469392 | 0.01093149 | 0.62250984 | 0.76923077 |
| VM27 | DQ469393 | 0.01280323 | 0.58847749 | 0.02710027 |
| VM27 | PvM03 | 0.01151666 | 0.70296128 | 0.01226994 |
| VM27 | PvM22 | 0.01482085 | 0.61190148 | 0.002997 |
| VM27 | BMD-5 | 0.01704876 | 0.59607861 | 0.000999 |
| VM27 | BMD-6 | 0.01072711 | 0.43712079 | 0.71428571 |
| VM27 | BMD-8 | 0.01087567 | 0.5426541 | 0.07042254 |
| VM27 | BMD-13 | 0.01557625 | 0.74129161 | 0 |
| VM27 | BMD-18 | 0.01498113 | 0.65692594 | 0 |
| VM27 | BMD-23 | 0.01027476 | 0.5604421 | 0.43478261 |
| VM27 | BMD-26 | 0.01289025 | 0.65676011 | 0.03937008 |
| VM27 | BMD-29 | 0.01394305 | 0.62593295 | 0.03891051 |
| VM27 | BMD-31 | 0.01465767 | 0.60098992 | 0.003996 |
| VM27 | BMD-48 | 0.01249116 | 0.54096562 | 0.17857143 |
| VM27 | BMD-35 | 0.01671971 | 0.60729134 | 0.000999 |
| VM27 | BMD-47 | 0.01531693 | 0.6669805 | 0.03424658 |
| VM27 | BMD-50 | 0.0178649 | 0.60547867 | 0 |
| VM27 | BMD-51 | 0.01242254 | 0.60683244 | 0.17857143 |
| VM27 | BMD-12 | 0.01250978 | 0.56315622 | 0.28571429 |
| VM27 | X21 | 0.01149595 | 0.60848491 | 0.11764706 |
| VM27 | X 34 | 0.01055621 | 0.60590898 | 0.2173913 |
| VM27 | X40 | 0.0098709 | 0.52997557 | 0.16949153 |
| VM27 | X49 | 0.01084944 | 0.53720524 | 0.10204082 |
| VM27 | X62 | 0.01228081 | 0.59333921 | 0.03610108 |
| VM27 | X65 | 0.01070945 | 0.55352286 | 0.05050505 |
| VM27 | X87 | 0.0138184 | 0.75375312 | 0.00799201 |
| VM27 | VR011 | 0.01508756 | 0.69288893 | 0.01149425 |
| VM27 | VR013 | 0.01025637 | 0.56446771 | 0.52631579 |
| VM27 | VR015 | 0.01618461 | 0.57165223 | 0.001998 |
| VM27 | VR016 | 0.0123071 | 0.63744148 | 0.02617801 |
| VM27 | VR018 | 0.01194432 | 0.67155674 | 0.01636661 |
| VM27 | VR022 | 0.01114467 | 0.57747199 | 0.04807692 |
| VM27 | VR022 | 0.01325716 | 0.71865293 | 0.0286533 |
| VM27 | VR024 | 0.01023708 | 0.48583067 | 0.58823529 |
| VM27 | VR025 | 0.01086693 | 0.54023267 | 0.02150538 |
| VM27 | VR032 | 0.01170872 | 0.56051909 | 0.27777778 |
| VM27 | VR033 | 0.014519 | 0.606911 | 0.01828154 |
| VM27 | VR035 | 0.01366131 | 0.59724677 | 0.00799201 |
| VM27 | VR037 | 0.0111975 | 0.6394624 | 0.01153403 |
| VM27 | VR039 | 0.01405023 | 0.64015563 | 0 |
| VM27 | VR048 | 0.01592953 | 0.58013436 | 0 |
| VM27 | DQ345305 | 0.0114722 | 0.63917288 | 0.1369863 |
| VM27 | CEDG036 | 0.0111911 | 0.50641966 | 0.0729927 |
| VM27 | CEDG291 | 0.01143987 | 0.65175471 | 0.01154734 |
| VM27 | CEDG220 | 0.01446482 | 0.65021213 | 0 |
| VM27 | CEDG136 | 0.01067186 | 0.58837663 | 0.27027027 |
| VM27 | CEDG100 | 0.01114229 | 0.67139799 | 0.01703578 |
| VM27 | CEDG096A | 0.01407318 | 0.73533971 | 0 |
| VM27 | CP00361 | 0.02177449 | 0.70341504 | 0 |
| VM27 | DMBSSR035 | 0.01291422 | 0.65673657 | 0.01494768 |
| VM27 | CEDG084 | 0.01493862 | 0.6114589 | 0.00899101 |
| VM27 | CEDG185 | 0.01361236 | 0.70649894 | 0 |
| VM27 | CEDC033 | 0.01276468 | 0.65458795 | 0.01945525 |
| VM27 | DMBSSR199 | 0.01343465 | 0.58380487 | 0.01751313 |
| VM27 | DMBSSR024 | 0.01302812 | 0.62382773 | 0.0177305 |
| VM27 | GMES0337 | 0.01197083 | 0.57247574 | 0.08849558 |
| VM27 | DMBSSR001 | 0.01492654 | 0.60684229 | 0.000999 |
| VM27 | CEDG118 | 0.01914149 | 0.65051779 | 0 |
| VM27 | CEDG146 | 0.01643731 | 0.63652842 | 0 |
| VM27 | CEDG225 | 0.01652842 | 0.68027238 | 0 |
| VM27 | JMES1424 | 0.01561455 | 0.62777347 | 0 |
| VM27 | MBSSR008 | 0.01392446 | 0.66330654 | 0 |
| VM27 | CEDG271 | 0.01648203 | 0.67363811 | 0.0140056 |
| VM27 | VM37 | 0.01411874 | 0.64604206 | 0 |
| VM27 | CEDG073 | 0.01565406 | 0.66732711 | 0.002997 |
| VM27 | CEDG071 | 0.01547498 | 0.67979955 | 0 |
| VM27 | CP00226 | 0.01455723 | 0.76240864 | 0.03623188 |
| VM27 | DMBSSR059 | 0.01378625 | 0.64259726 | 0 |
| BM212 | J01263 | 0.02103409 | 0.77999071 | 0 |
| BM212 | PV-at001 | 0.01240535 | 0.62256655 | 0.05208333 |
| BM212 | PV-ag003 | 0.01580978 | 0.64534936 | 0.00899101 |
| BM212 | PV-ag005 | 0.0161636 | 0.69257542 | 0.001998 |
| BM212 | Pv-ctt002 | 0.01256517 | 0.72350438 | 0.00799201 |
| BM212 | SSR-IAC 127 | 0.01168338 | 0.62954177 | 0.1754386 |
| BM212 | SSR-IAC 188 | 0.01283964 | 0.72491157 | 0.04237288 |
| BM212 | SSR-IAC 195 | 0.01174933 | 0.55017727 | 0.28571429 |
| BM212 | DQ469392 | 0.01000934 | 0.66237553 | 0.35714286 |
| BM212 | DQ469393 | 0.01429283 | 0.66369217 | 0.002997 |
| BM212 | PvM03 | 0.01195511 | 0.77031321 | 0.03144654 |
| BM212 | PvM22 | 0.01021114 | 0.5523159 | 0.76923077 |
| BM212 | BMD-5 | 0.0161709 | 0.65241179 | 0.000999 |
| BM212 | BMD-6 | 0.01730764 | 0.5293334 | 0.0136612 |
| BM212 | BMD-8 | 0.01702648 | 0.66177418 | 0 |
| BM212 | BMD-13 | 0.01348421 | 0.76509882 | 0.002997 |
| BM212 | BMD-18 | 0.01822724 | 0.70903131 | 0 |
| BM212 | BMD-23 | 0.0108415 | 0.61251015 | 0.08130081 |
| BM212 | BMD-26 | 0.01472697 | 0.68153346 | 0.00699301 |
| BM212 | BMD-29 | 0.01416178 | 0.6312126 | 0.001998 |
| BM212 | BMD-31 | 0.01637418 | 0.64170363 | 0.000999 |
| BM212 | BMD-48 | 0.01160213 | 0.54130017 | 0.18867925 |
| BM212 | BMD-35 | 0.01729874 | 0.64431141 | 0 |
| BM212 | BMD-47 | 0.01027763 | 0.64088217 | 0.15873016 |
| BM212 | BMD-50 | 0.01756658 | 0.62540547 | 0 |
| BM212 | BMD-51 | 0.01514132 | 0.68113889 | 0 |
| BM212 | BMD-12 | 0.01286411 | 0.59993885 | 0.04484305 |
| BM212 | X21 | 0.01342514 | 0.67182788 | 0.00599401 |
| BM212 | X 34 | 0.00969691 | 0.61088917 | 0.71428571 |
| BM212 | X40 | 0.00923033 | 0.53944795 | 0.90909091 |
| BM212 | X49 | 0.00964098 | 0.56697893 | 0.3125 |
| BM212 | X62 | 0.01655225 | 0.66873211 | 0 |
| BM212 | X65 | 0.01602951 | 0.64395362 | 0.000999 |
| BM212 | X87 | 0.01410486 | 0.77208468 | 0.00599401 |
| BM212 | VR011 | 0.01225589 | 0.69054245 | 0.05555556 |
| BM212 | VR013 | 0.01236795 | 0.61299582 | 0.26315789 |
| BM212 | VR015 | 0.01254853 | 0.57495844 | 0.04310345 |
| BM212 | VR016 | 0.01616681 | 0.73624005 | 0 |
| BM212 | VR018 | 0.01378282 | 0.72937505 | 0.003996 |
| BM212 | VR022 | 0.01337362 | 0.64362681 | 0.02336449 |
| BM212 | VR022 | 0.01252124 | 0.72020637 | 0.1 |
| BM212 | VR024 | 0.01061321 | 0.50895776 | 0.37037037 |
| BM212 | VR025 | 0.01366225 | 0.6144039 | 0.01094092 |
| BM212 | VR032 | 0.01185132 | 0.58484523 | 0.27777778 |
| BM212 | VR033 | 0.01194637 | 0.59488341 | 0.0280112 |
| BM212 | VR035 | 0.01392349 | 0.6214217 | 0.01858736 |
| BM212 | VR037 | 0.01091024 | 0.66523992 | 0.18181818 |
| BM212 | VR039 | 0.01643753 | 0.68370853 | 0 |
| BM212 | VR048 | 0.01828041 | 0.66197992 | 0 |
| BM212 | DQ345305 | 0.0111742 | 0.64327471 | 0.06896552 |
| BM212 | CEDG036 | 0.01345481 | 0.58249103 | 0.00599401 |
| BM212 | CEDG291 | 0.01340414 | 0.69578193 | 0.01109878 |
| BM212 | CEDG220 | 0.01565065 | 0.68752324 | 0 |
| BM212 | CEDG136 | 0.01369393 | 0.64747732 | 0.002997 |
| BM212 | CEDG100 | 0.01334931 | 0.72676266 | 0.03968254 |
| BM212 | CEDG096A | 0.01675389 | 0.79686545 | 0 |
| BM212 | CP00361 | 0.01901369 | 0.70604914 | 0 |
| BM212 | DMBSSR035 | 0.0148467 | 0.71894005 | 0.000999 |
| BM212 | CEDG084 | 0.01285243 | 0.62400561 | 0.05714286 |
| BM212 | CEDG185 | 0.0193252 | 0.7669768 | 0 |
| BM212 | CEDC033 | 0.01719226 | 0.76696261 | 0 |
| BM212 | DMBSSR199 | 0.01710151 | 0.63525173 | 0 |
| BM212 | DMBSSR024 | 0.01629781 | 0.72826799 | 0 |
| BM212 | GMES0337 | 0.01299782 | 0.63894901 | 0.004995 |
| BM212 | DMBSSR001 | 0.01643896 | 0.68253442 | 0 |
| BM212 | CEDG118 | 0.01731177 | 0.68332232 | 0 |
| BM212 | CEDG146 | 0.01626086 | 0.65264324 | 0.000999 |
| BM212 | CEDG225 | 0.02409179 | 0.76546498 | 0 |
| BM212 | JMES1424 | 0.01558112 | 0.66728381 | 0 |
| BM212 | MBSSR008 | 0.01835049 | 0.71885475 | 0.000999 |
| BM212 | CEDG271 | 0.01746994 | 0.72680874 | 0.000999 |
| BM212 | VM37 | 0.01497554 | 0.6794564 | 0.003996 |
| BM212 | CEDG073 | 0.01918037 | 0.73642104 | 0 |
| BM212 | CEDG071 | 0.01501024 | 0.69881557 | 0.001998 |
| BM212 | CP00226 | 0.01556528 | 0.81790352 | 0 |
| BM212 | DMBSSR059 | 0.01717677 | 0.69342875 | 0 |
| BM212 | VM27 | 0.01613078 | 0.74615878 | 0 |
| CP1225 | J01263 | 0.01229135 | 0.69055667 | 0.01919386 |
| CP1225 | PV-at001 | 0.01024597 | 0.59095167 | 0.29411765 |
| CP1225 | PV-ag003 | 0.01230907 | 0.58414176 | 0.55555556 |
| CP1225 | PV-ag005 | 0.00852323 | 0.57543534 | 0.90909091 |
| CP1225 | Pv-ctt002 | 0.01066619 | 0.69341039 | 0.5 |
| CP1225 | SSR-IAC 127 | 0.00980034 | 0.58213975 | 0.27777778 |
| CP1225 | SSR-IAC 188 | 0.01202287 | 0.69761247 | 0.02840909 |
| CP1225 | SSR-IAC 195 | 0.01077798 | 0.51692271 | 0.66666667 |
| CP1225 | DQ469392 | 0.01150177 | 0.68277199 | 0.58823529 |
| CP1225 | DQ469393 | 0.01643319 | 0.66886496 | 0.002997 |
| CP1225 | PvM03 | 0.01110604 | 0.70669022 | 0.24390244 |
| CP1225 | PvM22 | 0.00918718 | 0.52444352 | 0.55555556 |
| CP1225 | BMD-5 | 0.01725719 | 0.65113163 | 0 |
| CP1225 | BMD-6 | 0.00948058 | 0.47591951 | 0.34482759 |
| CP1225 | BMD-8 | 0.01059905 | 0.55225561 | 0.66666667 |
| CP1225 | BMD-13 | 0.01173379 | 0.73932314 | 0.03484321 |
| CP1225 | BMD-18 | 0.0120219 | 0.64894842 | 0.02421308 |
| CP1225 | BMD-23 | 0.00843047 | 0.54620853 | 0.66666667 |
| CP1225 | BMD-26 | 0.01021255 | 0.65156672 | 0.55555556 |
| CP1225 | BMD-29 | 0.01082982 | 0.62172696 | 0.24390244 |
| CP1225 | BMD-31 | 0.01057512 | 0.57032752 | 0.28571429 |
| CP1225 | BMD-48 | 0.01114465 | 0.55608327 | 0.23809524 |
| CP1225 | BMD-35 | 0.01198486 | 0.57547613 | 0.04166667 |
| CP1225 | BMD-47 | 0.01018663 | 0.62154962 | 0.66666667 |
| CP1225 | BMD-50 | 0.01496744 | 0.57777176 | 0.000999 |
| CP1225 | BMD-51 | 0.01393549 | 0.63289209 | 0.00699301 |
| CP1225 | BMD-12 | 0.01058294 | 0.56283278 | 0.15625 |
| CP1225 | X21 | 0.01088167 | 0.61526946 | 0.47619048 |
| CP1225 | X 34 | 0.00998728 | 0.61667687 | 0.1 |
| CP1225 | X40 | 0.00782941 | 0.51773494 | 0.83333333 |
| CP1225 | X49 | 0.01076943 | 0.56609412 | 0.27777778 |
| CP1225 | X62 | 0.01026746 | 0.57629824 | 0.32258065 |
| CP1225 | X65 | 0.00956486 | 0.54631602 | 0.19607843 |
| CP1225 | X87 | 0.01090754 | 0.73154456 | 0.34482759 |
| CP1225 | VR011 | 0.00919913 | 0.63012205 | 0.55555556 |
| CP1225 | VR013 | 0.01161741 | 0.60311775 | 0.37037037 |
| CP1225 | VR015 | 0.00773475 | 0.46592863 | 0.76923077 |
| CP1225 | VR016 | 0.00841778 | 0.5935818 | 0.66666667 |
| CP1225 | VR018 | 0.01146545 | 0.70004399 | 0.04329004 |
| CP1225 | VR022 | 0.01107839 | 0.59815472 | 0.06622517 |
| CP1225 | VR022 | 0.01372026 | 0.72141449 | 0.004995 |
| CP1225 | VR024 | 0.0099493 | 0.50945877 | 0.5 |
| CP1225 | VR025 | 0.01658705 | 0.63916823 | 0.000999 |
| CP1225 | VR032 | 0.01116285 | 0.55428934 | 0.34482759 |
| CP1225 | VR033 | 0.01075701 | 0.57680509 | 0.25 |
| CP1225 | VR035 | 0.01040903 | 0.56847514 | 0.35714286 |
| CP1225 | VR037 | 0.01069143 | 0.62853732 | 0.09345794 |
| CP1225 | VR039 | 0.01163946 | 0.62099499 | 0.06578947 |
| CP1225 | VR048 | 0.01041296 | 0.56396955 | 0.02155172 |
| CP1225 | DQ345305 | 0.01127393 | 0.65245066 | 0.32258065 |
| CP1225 | CEDG036 | 0.01066227 | 0.55281834 | 0.12345679 |
| CP1225 | CEDG291 | 0.01338728 | 0.71826914 | 0.004995 |
| CP1225 | CEDG220 | 0.0117398 | 0.62981329 | 0.11764706 |
| CP1225 | CEDG136 | 0.01459646 | 0.67771085 | 0.01019368 |
| CP1225 | CEDG100 | 0.01431266 | 0.75185981 | 0 |
| CP1225 | CEDG096A | 0.01193266 | 0.72141669 | 0.001998 |
| CP1225 | CP00361 | 0.0131692 | 0.65228721 | 0.07194245 |
| CP1225 | DMBSSR035 | 0.0120336 | 0.66711847 | 0.00899101 |
| CP1225 | CEDG084 | 0.00986602 | 0.57754328 | 0.22222222 |
| CP1225 | CEDG185 | 0.01366152 | 0.69191811 | 0.000999 |
| CP1225 | CEDC033 | 0.01237697 | 0.67643642 | 0.12658228 |
| CP1225 | DMBSSR199 | 0.01092646 | 0.55646781 | 0.625 |
| CP1225 | DMBSSR024 | 0.01230091 | 0.64435797 | 0.04166667 |
| CP1225 | GMES0337 | 0.01197208 | 0.6041761 | 0.34482759 |
| CP1225 | DMBSSR001 | 0.0152801 | 0.61667406 | 0 |
| CP1225 | CEDG118 | 0.0146043 | 0.64929434 | 0.001998 |
| CP1225 | CEDG146 | 0.01153837 | 0.59966176 | 0.10204082 |
| CP1225 | CEDG225 | 0.01519091 | 0.6991814 | 0.000999 |
| CP1225 | JMES1424 | 0.01286303 | 0.63077939 | 0.06578947 |
| CP1225 | MBSSR008 | 0.01008082 | 0.59739271 | 0.27777778 |
| CP1225 | CEDG271 | 0.0154227 | 0.71280325 | 0.004995 |
| CP1225 | VM37 | 0.0137395 | 0.66339611 | 0.00799201 |
| CP1225 | CEDG073 | 0.01056339 | 0.61780689 | 0.3030303 |
| CP1225 | CEDG071 | 0.01170731 | 0.63392665 | 0.02040816 |
| CP1225 | CP00226 | 0.01233746 | 0.78019922 | 0.11363636 |
| CP1225 | DMBSSR059 | 0.01117692 | 0.61589267 | 0.27777778 |
| CP1225 | VM27 | 0.01045729 | 0.64563959 | 0.35714286 |
| CP1225 | BM212 | 0.01268287 | 0.72259534 | 0.01814882 |
| DMBSSR016 | J01263 | 0.01140799 | 0.62890512 | 0.2173913 |
| DMBSSR016 | PV-at001 | 0.00988286 | 0.52869778 | 0.38461538 |
| DMBSSR016 | PV-ag003 | 0.01452563 | 0.59163526 | 0.01119821 |
| DMBSSR016 | PV-ag005 | 0.01219228 | 0.62771124 | 0.02314815 |
| DMBSSR016 | Pv-ctt002 | 0.01089465 | 0.66768382 | 0.27777778 |
| DMBSSR016 | SSR-IAC 127 | 0.01064017 | 0.56678225 | 0.625 |
| DMBSSR016 | SSR-IAC 188 | 0.01085221 | 0.65465486 | 0.41666667 |
| DMBSSR016 | SSR-IAC 195 | 0.01263085 | 0.53332987 | 0.08 |
| DMBSSR016 | DQ469392 | 0.01054003 | 0.61641696 | 0.2 |
| DMBSSR016 | DQ469393 | 0.01060995 | 0.55005463 | 0.43478261 |
| DMBSSR016 | PvM03 | 0.012652 | 0.72068293 | 0.002997 |
| DMBSSR016 | PvM22 | 0.01118026 | 0.51660826 | 0.35714286 |
| DMBSSR016 | BMD-5 | 0.01519232 | 0.57943786 | 0.03322259 |
| DMBSSR016 | BMD-6 | 0.00735901 | 0.37856379 | 0.625 |
| DMBSSR016 | BMD-8 | 0.01519014 | 0.59415465 | 0.000999 |
| DMBSSR016 | BMD-13 | 0.01416521 | 0.75390958 | 0.004995 |
| DMBSSR016 | BMD-18 | 0.01423955 | 0.65111408 | 0.00899101 |
| DMBSSR016 | BMD-23 | 0.01451763 | 0.59122738 | 0.003996 |
| DMBSSR016 | BMD-26 | 0.01377271 | 0.67218708 | 0.0862069 |
| DMBSSR016 | BMD-29 | 0.01242747 | 0.59796622 | 0.2173913 |
| DMBSSR016 | BMD-31 | 0.01147614 | 0.57987698 | 0.2173913 |
| DMBSSR016 | BMD-48 | 0.01340169 | 0.54515613 | 0.06666667 |
| DMBSSR016 | BMD-35 | 0.01224665 | 0.52486679 | 0.3125 |
| DMBSSR016 | BMD-47 | 0.0112299 | 0.60602034 | 0.14084507 |
| DMBSSR016 | BMD-50 | 0.01474899 | 0.54246428 | 0.03472222 |
| DMBSSR016 | BMD-51 | 0.01746489 | 0.64794328 | 0 |
| DMBSSR016 | BMD-12 | 0.01724678 | 0.58946775 | 0.000999 |
| DMBSSR016 | X21 | 0.01504117 | 0.63468124 | 0.00799201 |
| DMBSSR016 | X 34 | 0.01411348 | 0.64303279 | 0.06493506 |
| DMBSSR016 | X40 | 0.00849575 | 0.47328803 | 0.71428571 |
| DMBSSR016 | X49 | 0.0130745 | 0.57514346 | 0.13157895 |
| DMBSSR016 | X62 | 0.01189577 | 0.55418964 | 0.4 |
| DMBSSR016 | X65 | 0.01073171 | 0.54756544 | 0.17241379 |
| DMBSSR016 | X87 | 0.01136537 | 0.69676269 | 0.13157895 |
| DMBSSR016 | VR011 | 0.01298939 | 0.65438307 | 0.002997 |
| DMBSSR016 | VR013 | 0.01214742 | 0.58998509 | 0.04854369 |
| DMBSSR016 | VR015 | 0.0110926 | 0.48917398 | 0.43478261 |
| DMBSSR016 | VR016 | 0.0111511 | 0.61586522 | 0.11764706 |
| DMBSSR016 | VR018 | 0.01034935 | 0.65507726 | 0.38461538 |
| DMBSSR016 | VR022 | 0.01060911 | 0.5605501 | 0.45454545 |
| DMBSSR016 | VR022 | 0.01206448 | 0.67859113 | 0.22727273 |
| DMBSSR016 | VR024 | 0.014097 | 0.51926404 | 0.02967359 |
| DMBSSR016 | VR025 | 0.01168212 | 0.54164404 | 0.2 |
| DMBSSR016 | VR032 | 0.01018405 | 0.48756484 | 0.66666667 |
| DMBSSR016 | VR033 | 0.01316947 | 0.56100587 | 0.04032258 |
| DMBSSR016 | VR035 | 0.01055314 | 0.50772748 | 0.3125 |
| DMBSSR016 | VR037 | 0.01309352 | 0.6599836 | 0.02666667 |
| DMBSSR016 | VR039 | 0.01539475 | 0.64768405 | 0.01398601 |
| DMBSSR016 | VR048 | 0.01576822 | 0.60026646 | 0.002997 |
| DMBSSR016 | DQ345305 | 0.01380779 | 0.65006975 | 0.01182033 |
| DMBSSR016 | CEDG036 | 0.01162672 | 0.52131335 | 0.10526316 |
| DMBSSR016 | CEDG291 | 0.0096908 | 0.61076325 | 0.625 |
| DMBSSR016 | CEDG220 | 0.01795096 | 0.66511805 | 0 |
| DMBSSR016 | CEDG136 | 0.00953906 | 0.55838395 | 0.3125 |
| DMBSSR016 | CEDG100 | 0.01280671 | 0.68787285 | 0.004995 |
| DMBSSR016 | CEDG096A | 0.01336645 | 0.70816828 | 0 |
| DMBSSR016 | CP00361 | 0.01422266 | 0.63723074 | 0.000999 |
| DMBSSR016 | DMBSSR035 | 0.01335846 | 0.6736922 | 0.01077586 |
| DMBSSR016 | CEDG084 | 0.013072 | 0.58932956 | 0.01221001 |
| DMBSSR016 | CEDG185 | 0.01328655 | 0.65725066 | 0.01996008 |
| DMBSSR016 | CEDC033 | 0.00953496 | 0.60397721 | 0.71428571 |
| DMBSSR016 | DMBSSR199 | 0.00997866 | 0.52315717 | 0.11764706 |
| DMBSSR016 | DMBSSR024 | 0.01166454 | 0.60586021 | 0.16129032 |
| DMBSSR016 | GMES0337 | 0.01032197 | 0.55389028 | 0.24390244 |
| DMBSSR016 | DMBSSR001 | 0.01266939 | 0.56552704 | 0.22727273 |
| DMBSSR016 | CEDG118 | 0.01601183 | 0.62481377 | 0.000999 |
| DMBSSR016 | CEDG146 | 0.01477832 | 0.59877718 | 0 |
| DMBSSR016 | CEDG225 | 0.01338489 | 0.66772364 | 0.09345794 |
| DMBSSR016 | JMES1424 | 0.01336373 | 0.5684817 | 0.18181818 |
| DMBSSR016 | MBSSR008 | 0.01311212 | 0.63281478 | 0.08474576 |
| DMBSSR016 | CEDG271 | 0.01398039 | 0.66467968 | 0.001998 |
| DMBSSR016 | VM37 | 0.01086063 | 0.59856203 | 0.18867925 |
| DMBSSR016 | CEDG073 | 0.0117701 | 0.60263069 | 0.0952381 |
| DMBSSR016 | CEDG071 | 0.01216012 | 0.61971864 | 0.03676471 |
| DMBSSR016 | CP00226 | 0.01404889 | 0.7664275 | 0.001998 |
| DMBSSR016 | DMBSSR059 | 0.01190619 | 0.58991997 | 0.15151515 |
| DMBSSR016 | VM27 | 0.01245695 | 0.66533418 | 0.0280112 |
| DMBSSR016 | BM212 | 0.01254535 | 0.68478606 | 0.06451613 |
| DMBSSR016 | CP1225 | 0.01339802 | 0.69431202 | 0.04830918 |
| CEDG075 | J01263 | 0.01089269 | 0.55629451 | 0.22222222 |
| CEDG075 | PV-at001 | 0.01240797 | 0.53065464 | 0.0625 |
| CEDG075 | PV-ag003 | 0.0111683 | 0.46247828 | 0.32258065 |
| CEDG075 | PV-ag005 | 0.01425218 | 0.55781481 | 0.03846154 |
| CEDG075 | Pv-ctt002 | 0.01062296 | 0.59917265 | 0.37037037 |
| CEDG075 | SSR-IAC 127 | 0.01087162 | 0.49910026 | 0.18867925 |
| CEDG075 | SSR-IAC 188 | 0.01305763 | 0.62924915 | 0.02604167 |
| CEDG075 | SSR-IAC 195 | 0.0069574 | 0.34969323 | 0.90909091 |
| CEDG075 | DQ469392 | 0.01061202 | 0.55966614 | 0.625 |
| CEDG075 | DQ469393 | 0.01504644 | 0.53633636 | 0.01811594 |
| CEDG075 | PvM03 | 0.01024421 | 0.5916905 | 0.52631579 |
| CEDG075 | PvM22 | 0.01421596 | 0.48933278 | 0.01776199 |
| CEDG075 | BMD-5 | 0.01937431 | 0.56299469 | 0 |
| CEDG075 | BMD-6 | 0.00902339 | 0.33612029 | 0.28571429 |
| CEDG075 | BMD-8 | 0.01119547 | 0.48695123 | 0.06134969 |
| CEDG075 | BMD-13 | 0.01368324 | 0.64949552 | 0.00799201 |
| CEDG075 | BMD-18 | 0.01561002 | 0.59360739 | 0 |
| CEDG075 | BMD-23 | 0.01180426 | 0.49952854 | 0.37037037 |
| CEDG075 | BMD-26 | 0.01382193 | 0.57945752 | 0.02227171 |
| CEDG075 | BMD-29 | 0.00994395 | 0.47868464 | 0.34482759 |
| CEDG075 | BMD-31 | 0.01650462 | 0.5054836 | 0.01141553 |
| CEDG075 | BMD-48 | 0.01687051 | 0.51267255 | 0.01855288 |
| CEDG075 | BMD-35 | 0.01373796 | 0.47099575 | 0.04201681 |
| CEDG075 | BMD-47 | 0.00950595 | 0.51466128 | 0.71428571 |
| CEDG075 | BMD-50 | 0.01583148 | 0.47808549 | 0.003996 |
| CEDG075 | BMD-51 | 0.01277987 | 0.526034 | 0.0137741 |
| CEDG075 | BMD-12 | 0.00901313 | 0.41369977 | 0.83333333 |
| CEDG075 | X21 | 0.01140261 | 0.52313501 | 0.01253133 |
| CEDG075 | X 34 | 0.01269458 | 0.56605872 | 0.15151515 |
| CEDG075 | X40 | 0.01473741 | 0.49450643 | 0.03448276 |
| CEDG075 | X49 | 0.01323983 | 0.50630248 | 0.05347594 |
| CEDG075 | X62 | 0.01241181 | 0.50697919 | 0.01988072 |
| CEDG075 | X65 | 0.01091658 | 0.47904601 | 0.43478261 |
| CEDG075 | X87 | 0.01106368 | 0.64503731 | 0.23809524 |
| CEDG075 | VR011 | 0.01215745 | 0.60482268 | 0.04672897 |
| CEDG075 | VR013 | 0.01018584 | 0.48348947 | 0.45454545 |
| CEDG075 | VR015 | 0.01256281 | 0.42452087 | 0.11627907 |
| CEDG075 | VR016 | 0.01281615 | 0.54915516 | 0.27027027 |
| CEDG075 | VR018 | 0.01353073 | 0.60334479 | 0.10309278 |
| CEDG075 | VR022 | 0.01550568 | 0.55969154 | 0 |
| CEDG075 | VR022 | 0.01486245 | 0.66029442 | 0.001998 |
| CEDG075 | VR024 | 0.01339415 | 0.44717753 | 0.4 |
| CEDG075 | VR025 | 0.01066772 | 0.45474473 | 0.4 |
| CEDG075 | VR032 | 0.01010904 | 0.46420924 | 0.17857143 |
| CEDG075 | VR033 | 0.01116978 | 0.46974862 | 0.15151515 |
| CEDG075 | VR035 | 0.01281738 | 0.47875506 | 0.09009009 |
| CEDG075 | VR037 | 0.01339718 | 0.61580829 | 0.00699301 |
| CEDG075 | VR039 | 0.01120667 | 0.54004082 | 0.03076923 |
| CEDG075 | VR048 | 0.01366062 | 0.49193289 | 0.07352941 |
| CEDG075 | DQ345305 | 0.01151636 | 0.55273178 | 0.14492754 |
| CEDG075 | CEDG036 | 0.01125173 | 0.44564801 | 0.14285714 |
| CEDG075 | CEDG291 | 0.01170964 | 0.58351853 | 0.05586592 |
| CEDG075 | CEDG220 | 0.01495957 | 0.55284259 | 0.000999 |
| CEDG075 | CEDG136 | 0.01178335 | 0.5486332 | 0.08333333 |
| CEDG075 | CEDG100 | 0.0442805 | 0.76552008 | 0 |
| CEDG075 | CEDG096A | 0.01387062 | 0.67764138 | 0 |
| CEDG075 | CP00361 | 0.0177153 | 0.59021062 | 0 |
| CEDG075 | DMBSSR035 | 0.01427914 | 0.60472617 | 0.002997 |
| CEDG075 | CEDG084 | 0.01659651 | 0.52674587 | 0.001998 |
| CEDG075 | CEDG185 | 0.02909468 | 0.68221299 | 0 |
| CEDG075 | CEDC033 | 0.01155372 | 0.56599823 | 0.07575758 |
| CEDG075 | DMBSSR199 | 0.0084679 | 0.41904037 | 0.83333333 |
| CEDG075 | DMBSSR024 | 0.01504313 | 0.58769889 | 0.002997 |
| CEDG075 | GMES0337 | 0.01301528 | 0.53477493 | 0.02932551 |
| CEDG075 | DMBSSR001 | 0.01629513 | 0.56087712 | 0 |
| CEDG075 | CEDG118 | 0.01755781 | 0.53447466 | 0 |
| CEDG075 | CEDG146 | 0.01661219 | 0.53767728 | 0.004995 |
| CEDG075 | CEDG225 | 0.01246427 | 0.578693 | 0.004995 |
| CEDG075 | JMES1424 | 0.02070249 | 0.60401868 | 0 |
| CEDG075 | MBSSR008 | 0.01504443 | 0.58405677 | 0 |
| CEDG075 | CEDG271 | 0.01076624 | 0.53668884 | 0.11235955 |
| CEDG075 | VM37 | 0.0125236 | 0.54485762 | 0.01455604 |
| CEDG075 | CEDG073 | 0.01418341 | 0.58447924 | 0.00899101 |
| CEDG075 | CEDG071 | 0.0186594 | 0.60222793 | 0 |
| CEDG075 | CP00226 | 0.0118018 | 0.68953905 | 0.15384615 |
| CEDG075 | DMBSSR059 | 0.01614128 | 0.5344767 | 0.05102041 |
| CEDG075 | VM27 | 0.01536422 | 0.63873165 | 0 |
| CEDG075 | BM212 | 0.01304615 | 0.65004545 | 0.01149425 |
| CEDG075 | CP1225 | 0.01335756 | 0.6144243 | 0.004995 |
| CEDG075 | DMBSSR016 | 0.01262777 | 0.60916498 | 0.05128205 |
| BMD2 | J01263 | 0.01440995 | 0.59078629 | 0.003996 |
| BMD2 | PV-at001 | 0.01412137 | 0.46780663 | 0.06451613 |
| BMD2 | PV-ag003 | 0.01139541 | 0.47392642 | 0.2173913 |
| BMD2 | PV-ag005 | 0.01676422 | 0.59086006 | 0.001998 |
| BMD2 | Pv-ctt002 | 0.01215546 | 0.62599234 | 0.00699301 |
| BMD2 | SSR-IAC 127 | 0.01109135 | 0.49545277 | 0.06493506 |
| BMD2 | SSR-IAC 188 | 0.0112377 | 0.59914718 | 0.34482759 |
| BMD2 | DQ469392 | 0.01162355 | 0.57425335 | 0.08333333 |
| BMD2 | DQ469393 | 0.01181326 | 0.50347203 | 0.16129032 |
| BMD2 | PvM03 | 0.01316669 | 0.66593543 | 0.04032258 |
| BMD2 | PvM22 | 0.0122982 | 0.49666879 | 0.23255814 |
| BMD2 | BMD-5 | 0.01142852 | 0.45652733 | 0.22222222 |
| BMD2 | BMD-6 | 0.01123477 | 0.38427878 | 0.11235955 |
| BMD2 | BMD-8 | 0.01237803 | 0.48930547 | 0.01788909 |
| BMD2 | BMD-13 | 0.01502112 | 0.65284751 | 0.01984127 |
| BMD2 | BMD-18 | 0.01432914 | 0.55975195 | 0.03623188 |
| BMD2 | BMD-23 | 0.01210083 | 0.49062233 | 0.1754386 |
| BMD2 | BMD-26 | 0.0098102 | 0.54264147 | 0.58823529 |
| BMD2 | BMD-29 | 0.01267616 | 0.50820019 | 0.09009009 |
| BMD2 | BMD-31 | 0.01895204 | 0.56232854 | 0 |
| BMD2 | BMD-48 | 0.00984118 | 0.41096419 | 0.52631579 |
| BMD2 | BMD-35 | 0.01438843 | 0.42080729 | 0.04098361 |
| BMD2 | BMD-47 | 0.00940589 | 0.52013728 | 0.41666667 |
| BMD2 | BMD-50 | 0.01236194 | 0.4371589 | 0.06451613 |
| BMD2 | BMD-51 | 0.01120429 | 0.48709439 | 0.04739336 |
| BMD2 | BMD-12 | 0.01148727 | 0.47454735 | 0.07462687 |
| BMD2 | X21 | 0.01213611 | 0.54263157 | 0.02237136 |
| BMD2 | X 34 | 0.00835239 | 0.49339175 | 0.76923077 |
| BMD2 | X40 | 0.00863667 | 0.42293844 | 0.47619048 |
| BMD2 | X49 | 0.01020624 | 0.43097587 | 0.5 |
| BMD2 | X62 | 0.01496181 | 0.49176232 | 0.13513514 |
| BMD2 | X65 | 0.00893566 | 0.44762952 | 0.76923077 |
| BMD2 | X87 | 0.01016371 | 0.60090558 | 0.58823529 |
| BMD2 | VR011 | 0.01118221 | 0.57140178 | 0.27777778 |
| BMD2 | VR013 | 0.01039273 | 0.49591896 | 0.14285714 |
| BMD2 | VR015 | 0.01147481 | 0.42304683 | 0.25 |
| BMD2 | VR016 | 0.01213256 | 0.52768069 | 0.3125 |
| BMD2 | VR018 | 0.01509156 | 0.64651279 | 0.01146789 |
| BMD2 | VR022 | 0.0116342 | 0.51691056 | 0.03508772 |
| BMD2 | VR022 | 0.01713851 | 0.68000066 | 0 |
| BMD2 | VR024 | 0.01452818 | 0.4558528 | 0.02202643 |
| BMD2 | VR025 | 0.0105964 | 0.43821075 | 0.45454545 |
| BMD2 | VR032 | 0.01004035 | 0.45170518 | 0.71428571 |
| BMD2 | VR033 | 0.0121155 | 0.45823129 | 0.28571429 |
| BMD2 | VR035 | 0.01450825 | 0.49176493 | 0.01088139 |
| BMD2 | VR037 | 0.00951643 | 0.49780677 | 0.52631579 |
| BMD2 | VR039 | 0.01460014 | 0.56539603 | 0.001998 |
| BMD2 | VR048 | 0.01315056 | 0.46273285 | 0.03802281 |
| BMD2 | DQ345305 | 0.01714091 | 0.59333601 | 0.00699301 |
| BMD2 | CEDG036 | 0.01071931 | 0.40797184 | 0.47619048 |
| BMD2 | CEDG291 | 0.0146411 | 0.61294813 | 0.02178649 |
| BMD2 | CEDG220 | 0.01241568 | 0.49065993 | 0.12987013 |
| BMD2 | CEDG136 | 0.013422 | 0.5174503 | 0.34482759 |
| BMD2 | CEDG100 | 0.01670745 | 0.65648271 | 0.003996 |
| BMD2 | CEDG096A | 0.01478151 | 0.68626752 | 0.001998 |
| BMD2 | CP00361 | 0.01177854 | 0.51664602 | 0.03184713 |
| BMD2 | DMBSSR035 | 0.01590787 | 0.57461807 | 0.04761905 |
| BMD2 | CEDG084 | 0.01489876 | 0.53026813 | 0.02680965 |
| BMD2 | CEDG185 | 0.02011255 | 0.64592371 | 0.002997 |
| BMD2 | CEDC033 | 0.01466493 | 0.62061808 | 0.004995 |
| BMD2 | DMBSSR199 | 0.0135133 | 0.45433318 | 0.03030303 |
| BMD2 | DMBSSR024 | 0.01434976 | 0.55466998 | 0.01470588 |
| BMD2 | GMES0337 | 0.01185066 | 0.47428718 | 0.34482759 |
| BMD2 | DMBSSR001 | 0.01095067 | 0.48224028 | 0.43478261 |
| BMD2 | CEDG118 | 0.01390511 | 0.53486916 | 0.01331558 |
| BMD2 | CEDG146 | 0.0136448 | 0.49302162 | 0.07874016 |
| BMD2 | CEDG225 | 0.01523585 | 0.60104713 | 0.01996008 |
| BMD2 | JMES1424 | 0.01420934 | 0.51938802 | 0.05524862 |
| BMD2 | MBSSR008 | 0.0105315 | 0.51659604 | 0.76923077 |
| BMD2 | CEDG271 | 0.01236523 | 0.54741498 | 0.66666667 |
| BMD2 | VM37 | 0.0096153 | 0.50114538 | 0.1754386 |
| BMD2 | CEDG073 | 0.01214589 | 0.53896427 | 0 |
| BMD2 | CEDG071 | 0.01407809 | 0.55568906 | 0.02398082 |
| BMD2 | CP00226 | 0.01633229 | 0.71533921 | 0.000999 |
| BMD2 | DMBSSR059 | 0.01429075 | 0.56029083 | 0.04784689 |
| BMD2 | VM27 | 0.01292557 | 0.59357399 | 0.03246753 |
| BMD2 | BM212 | 0.01420919 | 0.63954822 | 0.000999 |
| BMD2 | CP1225 | 0.0095185 | 0.57468764 | 0.32258065 |
| BMD2 | DMBSSR016 | 0.0097005 | 0.53653283 | 0.4 |
| BMD2 | CEDG075 | 0.01293226 | 0.52069488 | 0.1369863 |
| BM149 | J01263 | 0.01645541 | 0.6666145 | 0 |
| BM149 | PV-at001 | 0.01010679 | 0.51344932 | 0.5 |
| BM149 | PV-ag003 | 0.01354947 | 0.54126079 | 0.25 |
| BM149 | PV-ag005 | 0.01356315 | 0.60423999 | 0.02114165 |
| BM149 | Pv-ctt002 | 0.01570001 | 0.68868747 | 0.01088139 |
| BM149 | SSR-IAC 127 | 0.00881097 | 0.51234197 | 0.90909091 |
| BM149 | SSR-IAC 188 | 0.009666 | 0.60811606 | 0.83333333 |
| BM149 | SSR-IAC 195 | 0.01591541 | 0.53474565 | 0.02222222 |
| BM149 | DQ469392 | 0.01157479 | 0.61268192 | 0.5 |
| BM149 | DQ469393 | 0.01285321 | 0.559456 | 0.02375297 |
| BM149 | PvM03 | 0.01230329 | 0.6748409 | 0.00799201 |
| BM149 | PvM22 | 0.01205338 | 0.50343867 | 0.55555556 |
| BM149 | BMD-5 | 0.01501772 | 0.55514552 | 0.02638522 |
| BM149 | BMD-6 | 0.01410433 | 0.47686852 | 0.2 |
| BM149 | BMD-8 | 0.01120757 | 0.50208854 | 0.03703704 |
| BM149 | BMD-13 | 0.01547707 | 0.72339693 | 0.00899101 |
| BM149 | BMD-18 | 0.01732242 | 0.65260235 | 0 |
| BM149 | BMD-23 | 0.01955256 | 0.61158029 | 0.02932551 |
| BM149 | BMD-26 | 0.01021279 | 0.59738676 | 0.06097561 |
| BM149 | BMD-29 | 0.01467517 | 0.60263154 | 0.03278689 |
| BM149 | BMD-31 | 0.0130121 | 0.56876499 | 0.05154639 |
| BM149 | BMD-48 | 0.01352014 | 0.52877579 | 0.02020202 |
| BM149 | BMD-35 | 0.01512415 | 0.52916371 | 0.03676471 |
| BM149 | BMD-47 | 0.01926253 | 0.6288319 | 0.15625 |
| BM149 | BMD-50 | 0.01448421 | 0.51464701 | 0.000999 |
| BM149 | BMD-51 | 0.01704086 | 0.62288558 | 0.00699301 |
| BM149 | BMD-12 | 0.01547048 | 0.54480311 | 0.52631579 |
| BM149 | X21 | 0.01263288 | 0.57985597 | 0.01733102 |
| BM149 | X 34 | 0.01144881 | 0.57241866 | 0.38461538 |
| BM149 | X40 | 0.01082681 | 0.5141143 | 0.3030303 |
| BM149 | X49 | 0.01361216 | 0.56563247 | 0.00699301 |
| BM149 | X62 | 0.0169649 | 0.57219871 | 0.00799201 |
| BM149 | X65 | 0.01215454 | 0.5393028 | 0.05952381 |
| BM149 | X87 | 0.01245325 | 0.71006511 | 0.06756757 |
| BM149 | VR011 | 0.01598203 | 0.66476805 | 0.000999 |
| BM149 | VR013 | 0.0088987 | 0.50470218 | 0.90909091 |
| BM149 | VR015 | 0.0179169 | 0.57437423 | 0.002997 |
| BM149 | VR016 | 0.01041698 | 0.57493143 | 0.06944444 |
| BM149 | VR018 | 0.01439727 | 0.66821886 | 0.00599401 |
| BM149 | VR022 | 0.01398179 | 0.5875008 | 0.06622517 |
| BM149 | VR022 | 0.01580055 | 0.6875777 | 0.004995 |
| BM149 | VR024 | 0.01285469 | 0.48493469 | 0.71428571 |
| BM149 | VR025 | 0.01298166 | 0.53958855 | 0 |
| BM149 | VR032 | 0.01212667 | 0.54296688 | 0.11235955 |
| BM149 | VR033 | 0.0143519 | 0.55453689 | 0.02469136 |
| BM149 | VR035 | 0.0111522 | 0.50414271 | 0.23809524 |
| BM149 | VR037 | 0.01255062 | 0.60837269 | 0.0304878 |
| BM149 | VR039 | 0.01412762 | 0.59629657 | 0 |
| BM149 | VR048 | 0.01314612 | 0.53173765 | 0.01060445 |
| BM149 | DQ345305 | 0.01711487 | 0.63531336 | 0.02079002 |
| BM149 | CEDG036 | 0.01169354 | 0.51253415 | 0.28571429 |
| BM149 | CEDG291 | 0.01409847 | 0.61234528 | 0.01081081 |
| BM149 | CEDG220 | 0.02222876 | 0.64531309 | 0 |
| BM149 | CEDG136 | 0.01595203 | 0.6348876 | 0.000999 |
| BM149 | CEDG100 | 0.01770753 | 0.74136897 | 0 |
| BM149 | CEDG096A | 0.01278689 | 0.67398358 | 0.02457002 |
| BM149 | CP00361 | 0.01475444 | 0.62219155 | 0.02145923 |
| BM149 | DMBSSR035 | 0.01603873 | 0.65206748 | 0.000999 |
| BM149 | CEDG084 | 0.00937182 | 0.50950971 | 0.20833333 |
| BM149 | CEDG185 | 0.01659706 | 0.67586296 | 0.000999 |
| BM149 | CEDC033 | 0.01346868 | 0.6464995 | 0.004995 |
| BM149 | DMBSSR199 | 0.01595827 | 0.58419791 | 0 |
| BM149 | DMBSSR024 | 0.01577514 | 0.60957287 | 0.000999 |
| BM149 | GMES0337 | 0.01335913 | 0.57097253 | 0.01243781 |
| BM149 | DMBSSR001 | 0.01735234 | 0.60053819 | 0.004995 |
| BM149 | CEDG118 | 0.01448553 | 0.60339122 | 0.000999 |
| BM149 | CEDG146 | 0.0172158 | 0.59603278 | 0.004995 |
| BM149 | CEDG225 | 0.01537142 | 0.65281379 | 0 |
| BM149 | JMES1424 | 0.0198334 | 0.61014542 | 0.000999 |
| BM149 | MBSSR008 | 0.01299377 | 0.59662264 | 0.05128205 |
| BM149 | CEDG271 | 0.02154144 | 0.71335756 | 0.002997 |
| BM149 | VM37 | 0.020534 | 0.67384825 | 0 |
| BM149 | CEDG073 | 0.0154942 | 0.62987229 | 0 |
| BM149 | CEDG071 | 0.01822926 | 0.65645348 | 0 |
| BM149 | CP00226 | 0.01493078 | 0.74654623 | 0.003996 |
| BM149 | DMBSSR059 | 0.01023871 | 0.52985153 | 0.04524887 |
| BM149 | VM27 | 0.01858022 | 0.66910133 | 0 |
| BM149 | BM212 | 0.01425709 | 0.68865236 | 0 |
| BM149 | CP1225 | 0.01474777 | 0.66052472 | 0.02717391 |
| BM149 | DMBSSR016 | 0.01271193 | 0.63552157 | 0.08403361 |
| BM149 | CEDG075 | 0.01609028 | 0.64260593 | 0 |
| BM149 | BMD2 | 0.01401044 | 0.55832828 | 0.16393443 |
| CP08695 | J01263 | 0.01292243 | 0.64502496 | 0.003996 |
| CP08695 | PV-at001 | 0.01295201 | 0.54518174 | 0.04291845 |
| CP08695 | PV-ag003 | 0.01184441 | 0.51329658 | 0.38461538 |
| CP08695 | PV-ag005 | 0.01684595 | 0.61762157 | 0 |
| CP08695 | Pv-ctt002 | 0.01033128 | 0.63265004 | 0.28571429 |
| CP08695 | SSR-IAC 127 | 0.01006453 | 0.52570396 | 0.58823529 |
| CP08695 | SSR-IAC 188 | 0.01446999 | 0.68978937 | 0.000999 |
| CP08695 | SSR-IAC 195 | 0.01697569 | 0.53767994 | 0.00899101 |
| CP08695 | DQ469392 | 0.00915635 | 0.56518012 | 0.58823529 |
| CP08695 | DQ469393 | 0.01124466 | 0.51925146 | 0.32258065 |
| CP08695 | PvM03 | 0.01180423 | 0.69946614 | 0.02415459 |
| CP08695 | PvM22 | 0.01472049 | 0.52135701 | 0.05494505 |
| CP08695 | BMD-5 | 0.01340217 | 0.5427494 | 0.03546099 |
| CP08695 | BMD-6 | 0.00941086 | 0.37377918 | 0.47619048 |
| CP08695 | BMD-8 | 0.01610852 | 0.55440629 | 0 |
| CP08695 | BMD-13 | 0.01219008 | 0.67093858 | 0.03095975 |
| CP08695 | BMD-18 | 0.01567807 | 0.61723217 | 0 |
| CP08695 | BMD-23 | 0.01186403 | 0.52391926 | 0.15625 |
| CP08695 | BMD-26 | 0.01070385 | 0.59011995 | 0.15384615 |
| CP08695 | BMD-29 | 0.01266937 | 0.59804791 | 0.00799201 |
| CP08695 | BMD-31 | 0.01778085 | 0.5893345 | 0 |
| CP08695 | BMD-48 | 0.01106102 | 0.46293117 | 0.12048193 |
| CP08695 | BMD-35 | 0.0155746 | 0.51898194 | 0.003996 |
| CP08695 | BMD-47 | 0.01152634 | 0.60314009 | 0.07092199 |
| CP08695 | BMD-50 | 0.01804113 | 0.53844049 | 0 |
| CP08695 | BMD-51 | 0.01724723 | 0.61972438 | 0 |
| CP08695 | BMD-12 | 0.01496782 | 0.53805914 | 0.01062699 |
| CP08695 | X21 | 0.01352883 | 0.58112882 | 0.002997 |
| CP08695 | X 34 | 0.01294411 | 0.60894451 | 0.03412969 |
| CP08695 | X40 | 0.01497586 | 0.56133749 | 0.003996 |
| CP08695 | X49 | 0.01176052 | 0.51281981 | 0.11764706 |
| CP08695 | X62 | 0.012127 | 0.53113615 | 0.1369863 |
| CP08695 | X65 | 0.01022415 | 0.48955573 | 0.3125 |
| CP08695 | X87 | 0.01043991 | 0.67071678 | 0.19607843 |
| CP08695 | VR011 | 0.01249749 | 0.61913859 | 0.04484305 |
| CP08695 | VR013 | 0.01196787 | 0.54127548 | 0.08928571 |
| CP08695 | VR015 | 0.01775483 | 0.55102405 | 0 |
| CP08695 | VR016 | 0.01269888 | 0.59697976 | 0.05524862 |
| CP08695 | VR018 | 0.01089484 | 0.64365384 | 0.11235955 |
| CP08695 | VR022 | 0.01037187 | 0.50818127 | 0.1010101 |
| CP08695 | VR022 | 0.01103888 | 0.63524038 | 0.25 |
| CP08695 | VR024 | 0.01074285 | 0.45547056 | 0.09174312 |
| CP08695 | VR025 | 0.01757128 | 0.55721923 | 0.001998 |
| CP08695 | VR032 | 0.01126394 | 0.47669989 | 0.20408163 |
| CP08695 | VR033 | 0.01690747 | 0.55842855 | 0 |
| CP08695 | VR035 | 0.01163386 | 0.48472465 | 0.06329114 |
| CP08695 | VR037 | 0.01091656 | 0.57321613 | 0.21276596 |
| CP08695 | VR039 | 0.01618758 | 0.61824939 | 0 |
| CP08695 | VR048 | 0.01401958 | 0.53192958 | 0.01901141 |
| CP08695 | DQ345305 | 0.012509 | 0.58879087 | 0.06711409 |
| CP08695 | CEDG036 | 0.01556002 | 0.54024029 | 0 |
| CP08695 | CEDG291 | 0.01555019 | 0.64264723 | 0.002997 |
| CP08695 | CEDG220 | 0.01396763 | 0.57713119 | 0 |
| CP08695 | CEDG136 | 0.01170679 | 0.56731929 | 0.21276596 |
| CP08695 | CEDG100 | 0.01777928 | 0.74312734 | 0 |
| CP08695 | CEDG096A | 0.01163465 | 0.6876304 | 0.01466276 |
| CP08695 | CP00361 | 0.01676062 | 0.65677201 | 0 |
| CP08695 | DMBSSR035 | 0.01790984 | 0.70765708 | 0 |
| CP08695 | CEDG084 | 0.01394187 | 0.56755958 | 0.002997 |
| CP08695 | CEDG185 | 0.01899884 | 0.69938151 | 0 |
| CP08695 | CEDC033 | 0.01224226 | 0.63331222 | 0.0141844 |
| CP08695 | DMBSSR199 | 0.01319648 | 0.52448173 | 0.0591716 |
| CP08695 | DMBSSR024 | 0.01165991 | 0.57070413 | 0.04255319 |
| CP08695 | GMES0337 | 0.01473325 | 0.54423253 | 0.01618123 |
| CP08695 | DMBSSR001 | 0.01591163 | 0.59797806 | 0 |
| CP08695 | CEDG118 | 0.0183623 | 0.63885917 | 0 |
| CP08695 | CEDG146 | 0.02010778 | 0.62801084 | 0 |
| CP08695 | CEDG225 | 0.01859683 | 0.65781116 | 0 |
| CP08695 | JMES1424 | 0.01384282 | 0.57169482 | 0.01026694 |
| CP08695 | MBSSR008 | 0.01713317 | 0.64435593 | 0 |
| CP08695 | CEDG271 | 0.01892965 | 0.68807469 | 0 |
| CP08695 | VM37 | 0.0127496 | 0.58963796 | 0.01579779 |
| CP08695 | CEDG073 | 0.01523765 | 0.6397517 | 0.001998 |
| CP08695 | CEDG071 | 0.01225648 | 0.58306803 | 0.02538071 |
| CP08695 | CP00226 | 0.013393 | 0.74816159 | 0.003996 |
| CP08695 | DMBSSR059 | 0.01360089 | 0.57559176 | 0.01824818 |
| CP08695 | VM27 | 0.01365467 | 0.63561895 | 0 |
| CP08695 | BM212 | 0.01639932 | 0.7218273 | 0 |
| CP08695 | CP1225 | 0.01275219 | 0.64108451 | 0.02717391 |
| CP08695 | DMBSSR016 | 0.01741207 | 0.6935403 | 0 |
| CP08695 | CEDG075 | 0.01768867 | 0.60945064 | 0 |
| CP08695 | BMD2 | 0.01922033 | 0.60546805 | 0 |
| CP08695 | BM149 | 0.01353529 | 0.62703351 | 0.00899101 |
| CEDG008 | J01263 | 0.0144721 | 0.69182315 | 0.000999 |
| CEDG008 | PV-at001 | 0.01626094 | 0.608327 | 0.003996 |
| CEDG008 | PV-ag003 | 0.01161204 | 0.5679709 | 0.33333333 |
| CEDG008 | PV-ag005 | 0.01385894 | 0.6260129 | 0.05434783 |
| CEDG008 | Pv-ctt002 | 0.01089399 | 0.65993804 | 0.3125 |
| CEDG008 | SSR-IAC 127 | 0.00956332 | 0.55115502 | 0.20408163 |
| CEDG008 | SSR-IAC 188 | 0.01192498 | 0.68906163 | 0.55555556 |
| CEDG008 | SSR-IAC 195 | 0.01084269 | 0.50461745 | 0.33333333 |
| CEDG008 | DQ469392 | 0.01027193 | 0.62169119 | 0.12048193 |
| CEDG008 | DQ469393 | 0.01451086 | 0.59827509 | 0.35714286 |
| CEDG008 | PvM03 | 0.01246744 | 0.71707993 | 0.0729927 |
| CEDG008 | PvM22 | 0.01218723 | 0.54905702 | 0.21276596 |
| CEDG008 | BMD-5 | 0.01553287 | 0.59590557 | 0.01748252 |
| CEDG008 | BMD-6 | 0.01455755 | 0.4758008 | 0.001998 |
| CEDG008 | BMD-8 | 0.01138006 | 0.56676222 | 0.13513514 |
| CEDG008 | BMD-13 | 0.01807696 | 0.76811996 | 0 |
| CEDG008 | BMD-18 | 0.01374227 | 0.63122375 | 0.05434783 |
| CEDG008 | BMD-23 | 0.01165117 | 0.58079958 | 0.35714286 |
| CEDG008 | BMD-26 | 0.01140706 | 0.64088409 | 0.21276596 |
| CEDG008 | BMD-29 | 0.01299668 | 0.58848128 | 0.0286533 |
| CEDG008 | BMD-31 | 0.01364839 | 0.59467473 | 0.002997 |
| CEDG008 | BMD-48 | 0.01161739 | 0.53912461 | 0.15151515 |
| CEDG008 | BMD-35 | 0.01356507 | 0.55495796 | 0.11764706 |
| CEDG008 | BMD-47 | 0.01092131 | 0.62275629 | 0.41666667 |
| CEDG008 | BMD-50 | 0.01276753 | 0.55179932 | 0.0286533 |
| CEDG008 | BMD-51 | 0.013638 | 0.60771233 | 0.15151515 |
| CEDG008 | BMD-12 | 0.00882902 | 0.51747311 | 0.66666667 |
| CEDG008 | X21 | 0.01403691 | 0.62257083 | 0.02590674 |
| CEDG008 | X 34 | 0.01093743 | 0.61235678 | 0.45454545 |
| CEDG008 | X40 | 0.00973951 | 0.52779218 | 0.76923077 |
| CEDG008 | X49 | 0.00970354 | 0.53935088 | 0.90909091 |
| CEDG008 | X62 | 0.01070694 | 0.54074286 | 0.4 |
| CEDG008 | X65 | 0.01282923 | 0.55674449 | 0.11627907 |
| CEDG008 | X87 | 0.01125571 | 0.70419031 | 0.58823529 |
| CEDG008 | VR011 | 0.01612023 | 0.66902874 | 0.001998 |
| CEDG008 | VR013 | 0.00981593 | 0.55577286 | 0.35714286 |
| CEDG008 | VR015 | 0.0078799 | 0.45517883 | 0.83333333 |
| CEDG008 | VR016 | 0.01222579 | 0.63930968 | 0.625 |
| CEDG008 | VR018 | 0.01978747 | 0.74676014 | 0 |
| CEDG008 | VR022 | 0.01203884 | 0.59348714 | 0.00599401 |
| CEDG008 | VR022 | 0.01610754 | 0.74749938 | 0.001998 |
| CEDG008 | VR024 | 0.0071297 | 0.41876781 | 0.71428571 |
| CEDG008 | VR025 | 0.01648013 | 0.63229203 | 0.00599401 |
| CEDG008 | VR032 | 0.01287733 | 0.56442578 | 0.03246753 |
| CEDG008 | VR033 | 0.01066723 | 0.55465035 | 0.55555556 |
| CEDG008 | VR035 | 0.01232547 | 0.54633863 | 0.1369863 |
| CEDG008 | VR037 | 0.01370491 | 0.6662357 | 0.01531394 |
| CEDG008 | VR039 | 0.01460524 | 0.61106414 | 0.14492754 |
| CEDG008 | VR048 | 0.01156788 | 0.54303603 | 0.15873016 |
| CEDG008 | DQ345305 | 0.01122271 | 0.6289655 | 0.12345679 |
| CEDG008 | CEDG036 | 0.01197335 | 0.54267583 | 0.18518519 |
| CEDG008 | CEDG291 | 0.01224834 | 0.67092966 | 0.11904762 |
| CEDG008 | CEDG220 | 0.0119718 | 0.60561073 | 0.07751938 |
| CEDG008 | CEDG136 | 0.01646982 | 0.65818377 | 0 |
| CEDG008 | CEDG100 | 0.02329115 | 0.72273179 | 0 |
| CEDG008 | CEDG096A | 0.01336693 | 0.73169205 | 0.000999 |
| CEDG008 | CP00361 | 0.01407454 | 0.62481071 | 0.001998 |
| CEDG008 | DMBSSR035 | 0.01499339 | 0.67508106 | 0.01908397 |
| CEDG008 | CEDG084 | 0.01083252 | 0.55074279 | 0.12987013 |
| CEDG008 | CEDG185 | 0.02209737 | 0.71151159 | 0 |
| CEDG008 | CEDC033 | 0.01161327 | 0.64722686 | 0.08695652 |
| CEDG008 | DMBSSR199 | 0.0125791 | 0.52797316 | 0.25 |
| CEDG008 | DMBSSR024 | 0.01296867 | 0.62186367 | 0.01672241 |
| CEDG008 | GMES0337 | 0.01496894 | 0.61769784 | 0.05154639 |
| CEDG008 | DMBSSR001 | 0.01266663 | 0.58341169 | 0.05291005 |
| CEDG008 | CEDG118 | 0.01124399 | 0.58145581 | 0.18867925 |
| CEDG008 | CEDG146 | 0.01215875 | 0.57050669 | 0.11363636 |
| CEDG008 | CEDG225 | 0.01504562 | 0.68186243 | 0.00699301 |
| CEDG008 | JMES1424 | 0.01596062 | 0.59385194 | 0.04291845 |
| CEDG008 | MBSSR008 | 0.01588383 | 0.66163533 | 0.00699301 |
| CEDG008 | CEDG271 | 0.01580329 | 0.64391879 | 0.001998 |
| CEDG008 | VM37 | 0.01138004 | 0.60392533 | 0.0625 |
| CEDG008 | CEDG073 | 0.01181558 | 0.60559625 | 0.23255814 |
| CEDG008 | CEDG071 | 0.01873712 | 0.67951341 | 0.000999 |
| CEDG008 | CP00226 | 0.01146837 | 0.71920246 | 0.2 |
| CEDG008 | DMBSSR059 | 0.02017 | 0.69014739 | 0 |
| CEDG008 | VM27 | 0.01755356 | 0.73122411 | 0 |
| CEDG008 | BM212 | 0.01224236 | 0.68654549 | 0.09009009 |
| CEDG008 | CP1225 | 0.01364072 | 0.69063784 | 0.004995 |
| CEDG008 | DMBSSR016 | 0.01125824 | 0.64166044 | 0.02724796 |
| CEDG008 | CEDG075 | 0.02838579 | 0.67866519 | 0 |
| CEDG008 | BMD2 | 0.01368151 | 0.59618576 | 0.07518797 |
| CEDG008 | BM149 | 0.01436989 | 0.66989042 | 0.00699301 |
| CEDG008 | CP08695 | 0.01252849 | 0.63250219 | 0.02096436 |
| CEDG256 | J01263 | 0.01705555 | 0.68838132 | 0 |
| CEDG256 | PV-at001 | 0.01092059 | 0.50927225 | 0.55555556 |
| CEDG256 | PV-ag003 | 0.01617398 | 0.53941457 | 0.14285714 |
| CEDG256 | PV-ag005 | 0.01691491 | 0.63234257 | 0.00899101 |
| CEDG256 | Pv-ctt002 | 0.01444601 | 0.67877463 | 0.06578947 |
| CEDG256 | SSR-IAC 127 | 0.01324778 | 0.58042732 | 0.14492754 |
| CEDG256 | SSR-IAC 188 | 0.01307383 | 0.671207 | 0.06849315 |
| CEDG256 | SSR-IAC 195 | 0.01017799 | 0.46387036 | 0.27027027 |
| CEDG256 | DQ469392 | 0.01231857 | 0.6116508 | 0.19230769 |
| CEDG256 | DQ469393 | 0.01722254 | 0.60353043 | 0.0117096 |
| CEDG256 | PvM03 | 0.01369642 | 0.71080789 | 0.000999 |
| CEDG256 | PvM22 | 0.01332504 | 0.52391604 | 0.01760563 |
| CEDG256 | BMD-5 | 0.01861367 | 0.58533826 | 0 |
| CEDG256 | BMD-6 | 0.01607636 | 0.42669453 | 0.15384615 |
| CEDG256 | BMD-8 | 0.01689208 | 0.6035759 | 0 |
| CEDG256 | BMD-13 | 0.01490003 | 0.70814791 | 0.000999 |
| CEDG256 | BMD-18 | 0.01639638 | 0.65306279 | 0 |
| CEDG256 | BMD-23 | 0.01643071 | 0.58447666 | 0.18181818 |
| CEDG256 | BMD-26 | 0.01312612 | 0.59689127 | 0.23255814 |
| CEDG256 | BMD-29 | 0.01380123 | 0.57421112 | 0.003996 |
| CEDG256 | BMD-31 | 0.01261934 | 0.54755069 | 0.06802721 |
| CEDG256 | BMD-48 | 0.01612384 | 0.53244037 | 0.00599401 |
| CEDG256 | BMD-35 | 0.01119092 | 0.46506709 | 0.47619048 |
| CEDG256 | BMD-47 | 0.01648157 | 0.63365549 | 0.02754821 |
| CEDG256 | BMD-50 | 0.01718628 | 0.54101735 | 0 |
| CEDG256 | BMD-51 | 0.01384961 | 0.57371951 | 0.01179245 |
| CEDG256 | BMD-12 | 0.01163505 | 0.5017661 | 0.05434783 |
| CEDG256 | X21 | 0.01299239 | 0.58824624 | 0.01497006 |
| CEDG256 | X 34 | 0.01689963 | 0.64958452 | 0 |
| CEDG256 | X40 | 0.01306068 | 0.51185938 | 0.05102041 |
| CEDG256 | X49 | 0.01162579 | 0.50923544 | 0.08849558 |
| CEDG256 | X62 | 0.01315742 | 0.55438954 | 0.06756757 |
| CEDG256 | X65 | 0.01055548 | 0.49391926 | 0.21276596 |
| CEDG256 | X87 | 0.01461833 | 0.70224688 | 0.02293578 |
| CEDG256 | VR011 | 0.01232373 | 0.61845943 | 0.07462687 |
| CEDG256 | VR013 | 0.00899964 | 0.51571783 | 0.71428571 |
| CEDG256 | VR015 | 0.0118336 | 0.45353978 | 0.5 |
| CEDG256 | VR016 | 0.01336197 | 0.60326058 | 0.03636364 |
| CEDG256 | VR018 | 0.01647609 | 0.70194864 | 0 |
| CEDG256 | VR022 | 0.01738248 | 0.59311946 | 0.004995 |
| CEDG256 | VR022 | 0.02012542 | 0.7412846 | 0 |
| CEDG256 | VR024 | 0.00969097 | 0.4552675 | 0.11627907 |
| CEDG256 | VR025 | 0.01532701 | 0.54614245 | 0.01980198 |
| CEDG256 | VR032 | 0.01605439 | 0.5801254 | 0.02309469 |
| CEDG256 | VR033 | 0.01562178 | 0.55724423 | 0.002997 |
| CEDG256 | VR035 | 0.02078644 | 0.57344367 | 0.000999 |
| CEDG256 | VR037 | 0.014411 | 0.62101544 | 0.13888889 |
| CEDG256 | VR039 | 0.01417324 | 0.59965591 | 0.03378378 |
| CEDG256 | VR048 | 0.01503458 | 0.53043905 | 0.08928571 |
| CEDG256 | DQ345305 | 0.01387817 | 0.6181011 | 0.01968504 |
| CEDG256 | CEDG036 | 0.00830409 | 0.44480101 | 0.71428571 |
| CEDG256 | CEDG291 | 0.01282622 | 0.61141245 | 0.002997 |
| CEDG256 | CEDG220 | 0.0155037 | 0.60780443 | 0 |
| CEDG256 | CEDG136 | 0.01122656 | 0.57545493 | 0.19230769 |
| CEDG256 | CEDG100 | 0.03373016 | 0.74954248 | 0 |
| CEDG256 | CEDG096A | 0.01369491 | 0.69446681 | 0.001998 |
| CEDG256 | CP00361 | 0.01862033 | 0.64554604 | 0 |
| CEDG256 | DMBSSR035 | 0.01427395 | 0.61902187 | 0.01623377 |
| CEDG256 | CEDG084 | 0.01352144 | 0.56375792 | 0.02717391 |
| CEDG256 | CEDG185 | 0.04808023 | 0.77559797 | 0 |
| CEDG256 | CEDC033 | 0.01348431 | 0.63678625 | 0.00899101 |
| CEDG256 | DMBSSR199 | 0.010969 | 0.5078856 | 0.55555556 |
| CEDG256 | DMBSSR024 | 0.01108347 | 0.5642498 | 0.0209205 |
| CEDG256 | GMES0337 | 0.01086242 | 0.53707163 | 0.19230769 |
| CEDG256 | DMBSSR001 | 0.01760487 | 0.61365447 | 0 |
| CEDG256 | CEDG118 | 0.02418518 | 0.64907519 | 0 |
| CEDG256 | CEDG146 | 0.0147511 | 0.56683569 | 0.00699301 |
| CEDG256 | CEDG225 | 0.01686072 | 0.68005279 | 0 |
| CEDG256 | JMES1424 | 0.01916285 | 0.59449289 | 0 |
| CEDG256 | MBSSR008 | 0.01421185 | 0.61775909 | 0 |
| CEDG256 | CEDG271 | 0.01988612 | 0.68443479 | 0 |
| CEDG256 | VM37 | 0.01218843 | 0.57058135 | 0.18867925 |
| CEDG256 | CEDG073 | 0.01578811 | 0.61429361 | 0.0120919 |
| CEDG256 | CEDG071 | 0.01502475 | 0.6048859 | 0.00799201 |
| CEDG256 | CP00226 | 0.01179098 | 0.69297665 | 0.32258065 |
| CEDG256 | DMBSSR059 | 0.02957716 | 0.64853922 | 0 |
| CEDG256 | VM27 | 0.0122735 | 0.64619005 | 0.04081633 |
| CEDG256 | BM212 | 0.01608469 | 0.71130407 | 0 |
| CEDG256 | CP1225 | 0.01471972 | 0.68811191 | 0.002997 |
| CEDG256 | DMBSSR016 | 0.01595963 | 0.67320216 | 0 |
| CEDG256 | CEDG075 | 0.03381882 | 0.67104402 | 0 |
| CEDG256 | BMD2 | 0.0176877 | 0.58206869 | 0.004995 |
| CEDG256 | BM149 | 0.01274697 | 0.59884349 | 0.04081633 |
| CEDG256 | CP08695 | 0.01752245 | 0.63865513 | 0 |
| CEDG256 | CEDG008 | 0.02652903 | 0.70891975 | 0 |
| BM146 | J01263 | 0.01385578 | 0.63823478 | 0.00599401 |
| BM146 | PV-at001 | 0.01369291 | 0.55849893 | 0.14285714 |
| BM146 | PV-ag003 | 0.01412613 | 0.54927501 | 0.04065041 |
| BM146 | PV-ag005 | 0.01744122 | 0.63236154 | 0.000999 |
| BM146 | Pv-ctt002 | 0.01276752 | 0.66071907 | 0.06578947 |
| BM146 | SSR-IAC 127 | 0.01326793 | 0.58346705 | 0.01602564 |
| BM146 | SSR-IAC 188 | 0.01345665 | 0.67435578 | 0.01838235 |
| BM146 | SSR-IAC 195 | 0.0130022 | 0.49612579 | 0.02188184 |
| BM146 | DQ469392 | 0.01309707 | 0.64309599 | 0.12345679 |
| BM146 | DQ469393 | 0.01505643 | 0.58915375 | 0.01792115 |
| BM146 | PvM03 | 0.01385228 | 0.66624296 | 0.004995 |
| BM146 | PvM22 | 0.01255894 | 0.52838864 | 0.07575758 |
| BM146 | BMD-5 | 0.0210397 | 0.65275786 | 0 |
| BM146 | BMD-6 | 0.01170263 | 0.43559174 | 0.10204082 |
| BM146 | BMD-8 | 0.0106618 | 0.50762858 | 0.14084507 |
| BM146 | BMD-13 | 0.01575865 | 0.71967684 | 0 |
| BM146 | BMD-18 | 0.01653061 | 0.62992149 | 0 |
| BM146 | BMD-23 | 0.01178789 | 0.53327994 | 0.24390244 |
| BM146 | BMD-26 | 0.01174716 | 0.57839776 | 0.33333333 |
| BM146 | BMD-29 | 0.01846305 | 0.63288478 | 0 |
| BM146 | BMD-31 | 0.01407239 | 0.55227955 | 0.08547009 |
| BM146 | BMD-48 | 0.01856555 | 0.5753801 | 0.002997 |
| BM146 | BMD-35 | 0.01008533 | 0.48474045 | 0.35714286 |
| BM146 | BMD-47 | 0.01644787 | 0.63348359 | 0.003996 |
| BM146 | BMD-50 | 0.01527972 | 0.54798475 | 0.00699301 |
| BM146 | BMD-51 | 0.01456832 | 0.58177988 | 0.02066116 |
| BM146 | BMD-12 | 0.01314776 | 0.54172102 | 0.00799201 |
| BM146 | X21 | 0.01730467 | 0.63420754 | 0 |
| BM146 | X 34 | 0.01201878 | 0.60649659 | 0.04366812 |
| BM146 | X40 | 0.01540364 | 0.55676075 | 0.01466276 |
| BM146 | X49 | 0.01181902 | 0.52024541 | 0.00899101 |
| BM146 | X62 | 0.01043484 | 0.51591888 | 0.29411765 |
| BM146 | X65 | 0.01187268 | 0.54492682 | 0.01213592 |
| BM146 | X87 | 0.01306566 | 0.70793644 | 0.02597403 |
| BM146 | VR011 | 0.0155451 | 0.66980223 | 0.003996 |
| BM146 | VR013 | 0.01098668 | 0.54096115 | 0.43478261 |
| BM146 | VR015 | 0.0118813 | 0.47655351 | 0.18181818 |
| BM146 | VR016 | 0.01524148 | 0.61799916 | 0.02688172 |
| BM146 | VR018 | 0.01811725 | 0.67795273 | 0 |
| BM146 | VR022 | 0.01526696 | 0.60411033 | 0 |
| BM146 | VR022 | 0.01669279 | 0.73129641 | 0 |
| BM146 | VR024 | 0.0120284 | 0.47981223 | 0.16949153 |
| BM146 | VR025 | 0.01346308 | 0.55161422 | 0.02717391 |
| BM146 | VR032 | 0.0110636 | 0.53076949 | 0.33333333 |
| BM146 | VR033 | 0.01396725 | 0.53744639 | 0.02212389 |
| BM146 | VR035 | 0.01814089 | 0.57662814 | 0.000999 |
| BM146 | VR037 | 0.01402849 | 0.64115527 | 0.01154734 |
| BM146 | VR039 | 0.01431797 | 0.57567397 | 0.00899101 |
| BM146 | VR048 | 0.01406822 | 0.51243884 | 0.0173913 |
| BM146 | DQ345305 | 0.01234883 | 0.58359378 | 0.55555556 |
| BM146 | CEDG036 | 0.0101458 | 0.47432906 | 0.3030303 |
| BM146 | CEDG291 | 0.01274367 | 0.63954645 | 0.00699301 |
| BM146 | CEDG220 | 0.01291345 | 0.57673969 | 0.02941176 |
| BM146 | CEDG136 | 0.01516688 | 0.61633327 | 0 |
| BM146 | CEDG100 | 0.03113608 | 0.73474373 | 0 |
| BM146 | CEDG096A | 0.0139278 | 0.7235184 | 0 |
| BM146 | CP00361 | 0.01622185 | 0.61734546 | 0 |
| BM146 | DMBSSR035 | 0.01667014 | 0.66604714 | 0 |
| BM146 | CEDG084 | 0.0154289 | 0.59025976 | 0 |
| BM146 | CEDG185 | 0.02638502 | 0.69824475 | 0 |
| BM146 | CEDC033 | 0.01325795 | 0.63315007 | 0.002997 |
| BM146 | DMBSSR199 | 0.01168922 | 0.51319247 | 0.35714286 |
| BM146 | DMBSSR024 | 0.01705586 | 0.63550309 | 0 |
| BM146 | GMES0337 | 0.01000907 | 0.50367693 | 0.5 |
| BM146 | DMBSSR001 | 0.01733936 | 0.6093415 | 0 |
| BM146 | CEDG118 | 0.01990315 | 0.6144577 | 0 |
| BM146 | CEDG146 | 0.01771947 | 0.58420722 | 0.01293661 |
| BM146 | CEDG225 | 0.01566669 | 0.63669902 | 0 |
| BM146 | JMES1424 | 0.01656034 | 0.60520932 | 0 |
| BM146 | MBSSR008 | 0.01472561 | 0.63169737 | 0 |
| BM146 | CEDG271 | 0.016078 | 0.63436137 | 0.00699301 |
| BM146 | VM37 | 0.01424237 | 0.62230569 | 0.002997 |
| BM146 | CEDG073 | 0.01639423 | 0.61718319 | 0.001998 |
| BM146 | CEDG071 | 0.01583504 | 0.58701078 | 0 |
| BM146 | CP00226 | 0.01327104 | 0.71083636 | 0.01508296 |
| BM146 | DMBSSR059 | 0.02020175 | 0.61659141 | 0 |
| BM146 | VM27 | 0.01855547 | 0.70720345 | 0 |
| BM146 | BM212 | 0.01408429 | 0.68803029 | 0.003996 |
| BM146 | CP1225 | 0.01359835 | 0.65528278 | 0.004995 |
| BM146 | DMBSSR016 | 0.01105787 | 0.59405659 | 0.07575758 |
| BM146 | CEDG075 | 0.04687731 | 0.74600346 | 0 |
| BM146 | BMD2 | 0.01281449 | 0.56238242 | 0.04504505 |
| BM146 | BM149 | 0.01293966 | 0.59781131 | 0.01336898 |
| BM146 | CP08695 | 0.01562705 | 0.63947067 | 0.002997 |
| BM146 | CEDG008 | 0.03390495 | 0.73165297 | 0 |
| BM146 | CEDG256 | 0.03611948 | 0.71160344 | 0 |
| CEDG050 | J01263 | 0.01784424 | 0.71729923 | 0 |
| CEDG050 | PV-at001 | 0.01508053 | 0.59158946 | 0.01470588 |
| CEDG050 | PV-ag003 | 0.01601229 | 0.59736834 | 0.003996 |
| CEDG050 | PV-ag005 | 0.01415156 | 0.63080138 | 0.04329004 |
| CEDG050 | Pv-ctt002 | 0.01509267 | 0.71073714 | 0 |
| CEDG050 | SSR-IAC 127 | 0.0129414 | 0.59785818 | 0.22727273 |
| CEDG050 | SSR-IAC 188 | 0.01276878 | 0.67666293 | 0.23255814 |
| CEDG050 | SSR-IAC 195 | 0.00976495 | 0.4818381 | 0.02808989 |
| CEDG050 | DQ469392 | 0.01587764 | 0.6687351 | 0.04672897 |
| CEDG050 | DQ469393 | 0.01400557 | 0.59061944 | 0.05181347 |
| CEDG050 | PvM03 | 0.01333963 | 0.71032116 | 0.02688172 |
| CEDG050 | PvM22 | 0.01648803 | 0.54695955 | 0.05714286 |
| CEDG050 | BMD-5 | 0.01965629 | 0.61155814 | 0 |
| CEDG050 | BMD-6 | 0.01263615 | 0.44071144 | 0.2173913 |
| CEDG050 | BMD-8 | 0.01486352 | 0.57980366 | 0 |
| CEDG050 | BMD-13 | 0.01570158 | 0.73859538 | 0 |
| CEDG050 | BMD-18 | 0.01930199 | 0.68201228 | 0 |
| CEDG050 | BMD-23 | 0.01813509 | 0.59054983 | 0.3125 |
| CEDG050 | BMD-26 | 0.02108348 | 0.67401348 | 0 |
| CEDG050 | BMD-29 | 0.02190624 | 0.66005002 | 0 |
| CEDG050 | BMD-31 | 0.01602691 | 0.58690891 | 0.01715266 |
| CEDG050 | BMD-48 | 0.01845117 | 0.58588139 | 0 |
| CEDG050 | BMD-35 | 0.01275028 | 0.51799069 | 0.09345794 |
| CEDG050 | BMD-47 | 0.01506699 | 0.63475856 | 0.001998 |
| CEDG050 | BMD-50 | 0.01965831 | 0.59497927 | 0 |
| CEDG050 | BMD-51 | 0.01617582 | 0.64181998 | 0 |
| CEDG050 | BMD-12 | 0.0116481 | 0.53028808 | 0.18181818 |
| CEDG050 | X21 | 0.01324921 | 0.59188088 | 0.004995 |
| CEDG050 | X 34 | 0.01726534 | 0.67665477 | 0 |
| CEDG050 | X40 | 0.01617875 | 0.59153782 | 0.001998 |
| CEDG050 | X49 | 0.01568918 | 0.57118846 | 0 |
| CEDG050 | X62 | 0.01331753 | 0.58535634 | 0.003996 |
| CEDG050 | X65 | 0.01227759 | 0.5588722 | 0.10638298 |
| CEDG050 | X87 | 0.01185501 | 0.69314652 | 0.01584786 |
| CEDG050 | VR011 | 0.01383121 | 0.66075866 | 0.00899101 |
| CEDG050 | VR013 | 0.01140008 | 0.56956015 | 0.29411765 |
| CEDG050 | VR015 | 0.00947045 | 0.4767921 | 0.66666667 |
| CEDG050 | VR016 | 0.01698307 | 0.6799016 | 0 |
| CEDG050 | VR018 | 0.01728363 | 0.68543909 | 0 |
| CEDG050 | VR022 | 0.01356213 | 0.5845209 | 0.002997 |
| CEDG050 | VR022 | 0.0146381 | 0.69200676 | 0 |
| CEDG050 | VR024 | 0.01395272 | 0.52706871 | 0.03703704 |
| CEDG050 | VR025 | 0.01642789 | 0.59855918 | 0 |
| CEDG050 | VR032 | 0.01222359 | 0.55565558 | 0.23255814 |
| CEDG050 | VR033 | 0.0161395 | 0.60775697 | 0 |
| CEDG050 | VR035 | 0.01382322 | 0.57113341 | 0.02739726 |
| CEDG050 | VR037 | 0.01523689 | 0.66256006 | 0 |
| CEDG050 | VR039 | 0.01680916 | 0.64153162 | 0 |
| CEDG050 | VR048 | 0.01692935 | 0.58934548 | 0 |
| CEDG050 | DQ345305 | 0.01106687 | 0.58904993 | 0.4 |
| CEDG050 | CEDG036 | 0.01377463 | 0.52117118 | 0.00899101 |
| CEDG050 | CEDG291 | 0.01133826 | 0.59935191 | 0.01187648 |
| CEDG050 | CEDG220 | 0.01642522 | 0.63398498 | 0.000999 |
| CEDG050 | CEDG136 | 0.01376831 | 0.63387474 | 0.02898551 |
| CEDG050 | CEDG100 | 0.02514755 | 0.75574731 | 0 |
| CEDG050 | CEDG096A | 0.01487042 | 0.73806282 | 0 |
| CEDG050 | CP00361 | 0.01932235 | 0.68490757 | 0 |
| CEDG050 | DMBSSR035 | 0.01637605 | 0.68649291 | 0 |
| CEDG050 | CEDG084 | 0.01444699 | 0.58860172 | 0.02967359 |
| CEDG050 | CEDG185 | 0.02762046 | 0.7514218 | 0 |
| CEDG050 | CEDC033 | 0.01657018 | 0.69892965 | 0 |
| CEDG050 | DMBSSR199 | 0.01144217 | 0.53153182 | 0.01085776 |
| CEDG050 | DMBSSR024 | 0.01530629 | 0.64662451 | 0.000999 |
| CEDG050 | GMES0337 | 0.01620708 | 0.59346814 | 0.01703578 |
| CEDG050 | DMBSSR001 | 0.01939036 | 0.60786381 | 0 |
| CEDG050 | CEDG118 | 0.02380342 | 0.64240979 | 0 |
| CEDG050 | CEDG146 | 0.02159973 | 0.64031309 | 0 |
| CEDG050 | CEDG225 | 0.01967656 | 0.70115414 | 0 |
| CEDG050 | JMES1424 | 0.02336582 | 0.69227664 | 0 |
| CEDG050 | MBSSR008 | 0.01273573 | 0.6022432 | 0.01068376 |
| CEDG050 | CEDG271 | 0.021759 | 0.70307736 | 0 |
| CEDG050 | VM37 | 0.01567267 | 0.66346918 | 0 |
| CEDG050 | CEDG073 | 0.01771498 | 0.6747199 | 0 |
| CEDG050 | CEDG071 | 0.01594307 | 0.63753398 | 0 |
| CEDG050 | CP00226 | 0.01393907 | 0.73343222 | 0.10638298 |
| CEDG050 | DMBSSR059 | 0.01326681 | 0.59487673 | 0.000999 |
| CEDG050 | VM27 | 0.01920482 | 0.71505966 | 0 |
| CEDG050 | BM212 | 0.01424444 | 0.70057954 | 0.003996 |
| CEDG050 | CP1225 | 0.01487496 | 0.68541048 | 0 |
| CEDG050 | DMBSSR016 | 0.01304091 | 0.64757904 | 0.01005025 |
| CEDG050 | CEDG075 | 0.02523699 | 0.61099801 | 0 |
| CEDG050 | BMD2 | 0.01156768 | 0.54141527 | 0.04608295 |
| CEDG050 | BM149 | 0.01976471 | 0.6867241 | 0 |
| CEDG050 | CP08695 | 0.01746662 | 0.68181171 | 0 |
| CEDG050 | CEDG008 | 0.0203163 | 0.6802939 | 0.002997 |
| CEDG050 | CEDG256 | 0.03048818 | 0.72417273 | 0 |
| CEDG050 | BM146 | 0.03716346 | 0.74377298 | 0 |
| CEDG150 | J01263 | 0.01257507 | 0.6697552 | 0.001998 |
| CEDG150 | PV-at001 | 0.01203782 | 0.60100411 | 0.06410256 |
| CEDG150 | PV-ag003 | 0.01271481 | 0.59054426 | 0.21276596 |
| CEDG150 | PV-ag005 | 0.01679172 | 0.69550316 | 0 |
| CEDG150 | Pv-ctt002 | 0.0131247 | 0.71684328 | 0.001998 |
| CEDG150 | SSR-IAC 127 | 0.01152257 | 0.61018208 | 0.06802721 |
| CEDG150 | SSR-IAC 188 | 0.00982485 | 0.65058078 | 0.43478261 |
| CEDG150 | SSR-IAC 195 | 0.01213013 | 0.53877055 | 0.16129032 |
| CEDG150 | DQ469392 | 0.01153845 | 0.66320394 | 0.07692308 |
| CEDG150 | DQ469393 | 0.01453112 | 0.62577264 | 0.00799201 |
| CEDG150 | PvM03 | 0.01125445 | 0.7010106 | 0.09803922 |
| CEDG150 | PvM22 | 0.01217229 | 0.57108254 | 0.05649718 |
| CEDG150 | BMD-5 | 0.01769387 | 0.64253555 | 0 |
| CEDG150 | BMD-6 | 0.01291436 | 0.49706532 | 0.05050505 |
| CEDG150 | BMD-8 | 0.00969826 | 0.52975866 | 0.66666667 |
| CEDG150 | BMD-13 | 0.01279396 | 0.72628775 | 0.00899101 |
| CEDG150 | BMD-18 | 0.01352464 | 0.66165822 | 0 |
| CEDG150 | BMD-23 | 0.01154233 | 0.59500568 | 0.04830918 |
| CEDG150 | BMD-26 | 0.01144833 | 0.65095975 | 0.15625 |
| CEDG150 | BMD-29 | 0.01367767 | 0.63989455 | 0 |
| CEDG150 | BMD-31 | 0.01287362 | 0.61016704 | 0.06802721 |
| CEDG150 | BMD-48 | 0.0232929 | 0.66132782 | 0 |
| CEDG150 | BMD-35 | 0.0116682 | 0.55171668 | 0.76923077 |
| CEDG150 | BMD-47 | 0.01249172 | 0.64149448 | 0.16666667 |
| CEDG150 | BMD-50 | 0.01825514 | 0.58785693 | 0.000999 |
| CEDG150 | BMD-51 | 0.01014986 | 0.56404382 | 0.27027027 |
| CEDG150 | BMD-12 | 0.01093138 | 0.54173705 | 0.18867925 |
| CEDG150 | X21 | 0.01423599 | 0.645866 | 0.000999 |
| CEDG150 | X 34 | 0.01321995 | 0.65151764 | 0.08333333 |
| CEDG150 | X40 | 0.01105105 | 0.54158518 | 0.11111111 |
| CEDG150 | X49 | 0.01157797 | 0.55373483 | 0.09433962 |
| CEDG150 | X62 | 0.01333348 | 0.60773748 | 0.02132196 |
| CEDG150 | X65 | 0.01196394 | 0.56950181 | 0.09259259 |
| CEDG150 | X87 | 0.011456 | 0.72700299 | 0.03690037 |
| CEDG150 | VR011 | 0.01478774 | 0.71406171 | 0 |
| CEDG150 | VR013 | 0.00967573 | 0.5557708 | 0.58823529 |
| CEDG150 | VR015 | 0.01415229 | 0.55538202 | 0.0310559 |
| CEDG150 | VR016 | 0.0137416 | 0.65282946 | 0.02840909 |
| CEDG150 | VR018 | 0.01655197 | 0.72161381 | 0 |
| CEDG150 | VR022 | 0.01399569 | 0.63671862 | 0.01964637 |
| CEDG150 | VR022 | 0.01565713 | 0.74787858 | 0.002997 |
| CEDG150 | VR024 | 0.01340089 | 0.53342596 | 0.05 |
| CEDG150 | VR025 | 0.0144514 | 0.60824735 | 0.03289474 |
| CEDG150 | VR032 | 0.0099783 | 0.54421474 | 0.3125 |
| CEDG150 | VR033 | 0.01744098 | 0.63259909 | 0 |
| CEDG150 | VR035 | 0.01394557 | 0.59898289 | 0.0265252 |
| CEDG150 | VR037 | 0.01346247 | 0.68266506 | 0.01845018 |
| CEDG150 | VR039 | 0.0114688 | 0.6052527 | 0.03205128 |
| CEDG150 | VR048 | 0.01811978 | 0.62005537 | 0.001998 |
| CEDG150 | DQ345305 | 0.01105424 | 0.63954243 | 0.14285714 |
| CEDG150 | CEDG036 | 0.01553427 | 0.58712959 | 0 |
| CEDG150 | CEDG291 | 0.01276402 | 0.66408 | 0.001998 |
| CEDG150 | CEDG220 | 0.01492589 | 0.65236463 | 0 |
| CEDG150 | CEDG136 | 0.01286466 | 0.6461841 | 0.00799201 |
| CEDG150 | CEDG100 | 0.04324541 | 0.83452574 | 0 |
| CEDG150 | CEDG096A | 0.01198045 | 0.72485598 | 0.00599401 |
| CEDG150 | CP00361 | 0.01663157 | 0.68472197 | 0 |
| CEDG150 | DMBSSR035 | 0.01500687 | 0.68374647 | 0 |
| CEDG150 | CEDG084 | 0.01575349 | 0.63058639 | 0.02262443 |
| CEDG150 | CEDG185 | 0.02424125 | 0.75310228 | 0 |
| CEDG150 | CEDC033 | 0.01188117 | 0.6522565 | 0.07518797 |
| CEDG150 | DMBSSR199 | 0.0144342 | 0.58178822 | 0.12195122 |
| CEDG150 | DMBSSR024 | 0.01459165 | 0.67111135 | 0 |
| CEDG150 | GMES0337 | 0.01504301 | 0.63895225 | 0.00599401 |
| CEDG150 | DMBSSR001 | 0.01495408 | 0.61156563 | 0.01497006 |
| CEDG150 | CEDG118 | 0.01468158 | 0.6226528 | 0.003996 |
| CEDG150 | CEDG146 | 0.01336274 | 0.59769313 | 0.10752688 |
| CEDG150 | CEDG225 | 0.01540853 | 0.69069585 | 0 |
| CEDG150 | JMES1424 | 0.01807559 | 0.67220008 | 0 |
| CEDG150 | MBSSR008 | 0.0136294 | 0.65981 | 0.001998 |
| CEDG150 | CEDG271 | 0.02109011 | 0.73799021 | 0 |
| CEDG150 | VM37 | 0.01235427 | 0.63170635 | 0.03344482 |
| CEDG150 | CEDG073 | 0.01513199 | 0.65852236 | 0 |
| CEDG150 | CEDG071 | 0.01300739 | 0.60570405 | 0.05988024 |
| CEDG150 | CP00226 | 0.01220392 | 0.74218793 | 0.03496503 |
| CEDG150 | DMBSSR059 | 0.01661195 | 0.63703474 | 0.000999 |
| CEDG150 | VM27 | 0.01351351 | 0.67144145 | 0.000999 |
| CEDG150 | BM212 | 0.01454292 | 0.72720604 | 0.000999 |
| CEDG150 | CP1225 | 0.0137886 | 0.71537553 | 0.01529052 |
| CEDG150 | DMBSSR016 | 0.01402441 | 0.67687803 | 0.002997 |
| CEDG150 | CEDG075 | 0.03961502 | 0.746017 | 0 |
| CEDG150 | BMD2 | 0.01090646 | 0.55434446 | 0.5 |
| CEDG150 | BM149 | 0.01619769 | 0.71083645 | 0 |
| CEDG150 | CP08695 | 0.01426742 | 0.68486692 | 0 |
| CEDG150 | CEDG008 | 0.0264415 | 0.73057144 | 0 |
| CEDG150 | CEDG256 | 0.03106279 | 0.73423806 | 0 |
| CEDG150 | BM146 | 0.04408876 | 0.78925551 | 0 |
| CEDG150 | CEDG050 | 0.02189966 | 0.72344054 | 0 |
| CEDG267 | J01263 | 0.01797935 | 0.67079135 | 0 |
| CEDG267 | PV-at001 | 0.0123675 | 0.55569773 | 0.04310345 |
| CEDG267 | PV-ag003 | 0.01141565 | 0.49772775 | 0.625 |
| CEDG267 | PV-ag005 | 0.01648946 | 0.62148953 | 0.002997 |
| CEDG267 | Pv-ctt002 | 0.01321567 | 0.65928536 | 0 |
| CEDG267 | SSR-IAC 127 | 0.01203606 | 0.5538036 | 0.19230769 |
| CEDG267 | SSR-IAC 188 | 0.01153814 | 0.63400022 | 0.4 |
| CEDG267 | SSR-IAC 195 | 0.01159647 | 0.46570353 | 0.17241379 |
| CEDG267 | DQ469392 | 0.01293426 | 0.61851485 | 0.01329787 |
| CEDG267 | DQ469393 | 0.01257731 | 0.54488977 | 0.02141328 |
| CEDG267 | PvM03 | 0.01104104 | 0.63845476 | 0.25641026 |
| CEDG267 | PvM22 | 0.01037268 | 0.48069898 | 0.43478261 |
| CEDG267 | BMD-5 | 0.02152532 | 0.61877179 | 0 |
| CEDG267 | BMD-6 | 0.01445364 | 0.42997288 | 0.04424779 |
| CEDG267 | BMD-8 | 0.00997467 | 0.49292629 | 0.13333333 |
| CEDG267 | BMD-13 | 0.01328518 | 0.69137282 | 0.03355705 |
| CEDG267 | BMD-18 | 0.01768988 | 0.63904987 | 0 |
| CEDG267 | BMD-23 | 0.01093469 | 0.51469684 | 0.55555556 |
| CEDG267 | BMD-26 | 0.01291277 | 0.61253147 | 0.10752688 |
| CEDG267 | BMD-29 | 0.01829288 | 0.6080546 | 0 |
| CEDG267 | BMD-31 | 0.0130396 | 0.52731176 | 0.2 |
| CEDG267 | BMD-48 | 0.01173076 | 0.47749497 | 0.43478261 |
| CEDG267 | BMD-35 | 0.01419049 | 0.50671839 | 0.03076923 |
| CEDG267 | BMD-47 | 0.01044393 | 0.54682432 | 0.35714286 |
| CEDG267 | BMD-50 | 0.01502534 | 0.5247364 | 0.01023541 |
| CEDG267 | BMD-51 | 0.01685118 | 0.61333078 | 0 |
| CEDG267 | BMD-12 | 0.0133773 | 0.51770947 | 0.04761905 |
| CEDG267 | X21 | 0.01283841 | 0.57289177 | 0.00899101 |
| CEDG267 | X 34 | 0.01034559 | 0.5593111 | 0.45454545 |
| CEDG267 | X40 | 0.01310925 | 0.49815786 | 0.02079002 |
| CEDG267 | X49 | 0.0113603 | 0.49477787 | 0.34482759 |
| CEDG267 | X62 | 0.01099096 | 0.51309021 | 0.4 |
| CEDG267 | X65 | 0.01256831 | 0.53297891 | 0.0952381 |
| CEDG267 | X87 | 0.01311912 | 0.68366739 | 0.02906977 |
| CEDG267 | VR011 | 0.01318367 | 0.62136214 | 0.02192982 |
| CEDG267 | VR013 | 0.01339585 | 0.54871049 | 0.28571429 |
| CEDG267 | VR015 | 0.01397688 | 0.48618535 | 0.03355705 |
| CEDG267 | VR016 | 0.01191957 | 0.58157294 | 0.23809524 |
| CEDG267 | VR018 | 0.01241216 | 0.65521562 | 0.00599401 |
| CEDG267 | VR022 | 0.01352073 | 0.54581639 | 0.00899101 |
| CEDG267 | VR022 | 0.01232609 | 0.66095546 | 0.06060606 |
| CEDG267 | VR024 | 0.01768922 | 0.53137193 | 0.002997 |
| CEDG267 | VR025 | 0.01648126 | 0.55646375 | 0.003996 |
| CEDG267 | VR032 | 0.00921774 | 0.4851191 | 0.45454545 |
| CEDG267 | VR033 | 0.01420854 | 0.5715608 | 0.02105263 |
| CEDG267 | VR035 | 0.01361298 | 0.52803501 | 0.03333333 |
| CEDG267 | VR037 | 0.01163547 | 0.59700552 | 0.35714286 |
| CEDG267 | VR039 | 0.01410368 | 0.58698653 | 0.06493506 |
| CEDG267 | VR048 | 0.01318616 | 0.51273795 | 0.02857143 |
| CEDG267 | DQ345305 | 0.01414255 | 0.62213041 | 0.00599401 |
| CEDG267 | CEDG036 | 0.01245095 | 0.51256572 | 0.06944444 |
| CEDG267 | CEDG291 | 0.01290655 | 0.64755399 | 0 |
| CEDG267 | CEDG220 | 0.01452964 | 0.58285339 | 0.002997 |
| CEDG267 | CEDG136 | 0.01393779 | 0.59276975 | 0.01492537 |
| CEDG267 | CEDG100 | 0.03478243 | 0.76438937 | 0 |
| CEDG267 | CEDG096A | 0.01601476 | 0.7335531 | 0 |
| CEDG267 | CP00361 | 0.02008004 | 0.61434063 | 0 |
| CEDG267 | DMBSSR035 | 0.01328488 | 0.61603262 | 0.01828154 |
| CEDG267 | CEDG084 | 0.01435092 | 0.53128817 | 0.01785714 |
| CEDG267 | CEDG185 | 0.02801724 | 0.75318297 | 0 |
| CEDG267 | CEDC033 | 0.01249069 | 0.58214864 | 0.02386635 |
| CEDG267 | DMBSSR199 | 0.01521522 | 0.53720577 | 0.02320186 |
| CEDG267 | DMBSSR024 | 0.01425059 | 0.58498481 | 0.002997 |
| CEDG267 | GMES0337 | 0.01265232 | 0.54347086 | 0.12820513 |
| CEDG267 | DMBSSR001 | 0.013871 | 0.54921067 | 0.05681818 |
| CEDG267 | CEDG118 | 0.01440352 | 0.56510754 | 0.000999 |
| CEDG267 | CEDG146 | 0.01606023 | 0.57432733 | 0 |
| CEDG267 | CEDG225 | 0.01410364 | 0.62836162 | 0.01663894 |
| CEDG267 | JMES1424 | 0.01733095 | 0.6020258 | 0.002997 |
| CEDG267 | MBSSR008 | 0.0154311 | 0.61092601 | 0 |
| CEDG267 | CEDG271 | 0.01980088 | 0.67295982 | 0 |
| CEDG267 | VM37 | 0.01543267 | 0.59721632 | 0 |
| CEDG267 | CEDG073 | 0.01565726 | 0.61495216 | 0 |
| CEDG267 | CEDG071 | 0.01593141 | 0.58339311 | 0.02380952 |
| CEDG267 | CP00226 | 0.01250058 | 0.71567267 | 0.04273504 |
| CEDG267 | DMBSSR059 | 0.01703627 | 0.62674345 | 0.000999 |
| CEDG267 | VM27 | 0.01530482 | 0.68709536 | 0 |
| CEDG267 | BM212 | 0.01804008 | 0.69027152 | 0 |
| CEDG267 | CP1225 | 0.01423187 | 0.67049902 | 0 |
| CEDG267 | DMBSSR016 | 0.01548604 | 0.6369114 | 0.000999 |
| CEDG267 | CEDG075 | 0.04046222 | 0.71593444 | 0 |
| CEDG267 | BMD2 | 0.0109169 | 0.52946937 | 0.04651163 |
| CEDG267 | BM149 | 0.01356405 | 0.60578605 | 0.13888889 |
| CEDG267 | CP08695 | 0.01482571 | 0.62991606 | 0 |
| CEDG267 | CEDG008 | 0.02919694 | 0.72738306 | 0 |
| CEDG267 | CEDG256 | 0.02859268 | 0.667865 | 0 |
| CEDG267 | BM146 | 0.0304598 | 0.67931251 | 0 |
| CEDG267 | CEDG050 | 0.02152894 | 0.66606794 | 0 |
| CEDG267 | CEDG150 | 0.03313546 | 0.76169968 | 0 |
| CEDG115 | J01263 | 0.01310443 | 0.73446792 | 0.000999 |
| CEDG115 | PV-at001 | 0.01423402 | 0.67748121 | 0.10526316 |
| CEDG115 | PV-ag003 | 0.0148398 | 0.67029874 | 0.11235955 |
| CEDG115 | Pv-ctt002 | 0.01257922 | 0.73209434 | 0.14285714 |
| CEDG115 | SSR-IAC 127 | 0.01237293 | 0.69305387 | 0.17857143 |
| CEDG115 | SSR-IAC 188 | 0.01223795 | 0.75784326 | 0.08547009 |
| CEDG115 | SSR-IAC 195 | 0.011223 | 0.60387191 | 0.43478261 |
| CEDG115 | DQ469392 | 0.01202699 | 0.71577453 | 0.41666667 |
| CEDG115 | DQ469393 | 0.01449451 | 0.70726235 | 0.05780347 |
| CEDG115 | PvM03 | 0.01611351 | 0.77554269 | 0 |
| CEDG115 | PvM22 | 0.01479578 | 0.62566976 | 0.03184713 |
| CEDG115 | BMD-5 | 0.01322373 | 0.63562149 | 0.000999 |
| CEDG115 | BMD-6 | 0.01250919 | 0.56502183 | 0.06711409 |
| CEDG115 | BMD-8 | 0.00897422 | 0.58972382 | 0.66666667 |
| CEDG115 | BMD-13 | 0.01195984 | 0.75546867 | 0.07194245 |
| CEDG115 | BMD-18 | 0.01207129 | 0.71104735 | 0.02096436 |
| CEDG115 | BMD-23 | 0.01402024 | 0.62842767 | 0.71428571 |
| CEDG115 | BMD-26 | 0.01220088 | 0.72553887 | 0.04464286 |
| CEDG115 | BMD-29 | 0.01330435 | 0.67773368 | 0.05208333 |
| CEDG115 | BMD-31 | 0.01345265 | 0.67138068 | 0.03378378 |
| CEDG115 | BMD-48 | 0.01870039 | 0.70008301 | 0 |
| CEDG115 | BMD-35 | 0.01212595 | 0.61661576 | 0.18181818 |
| CEDG115 | BMD-50 | 0.01391011 | 0.62337741 | 0.04830918 |
| CEDG115 | BMD-51 | 0.0132406 | 0.67943269 | 0.041841 |
| CEDG115 | BMD-12 | 0.01078844 | 0.60705075 | 0.45454545 |
| CEDG115 | X21 | 0.01354112 | 0.68352592 | 0.004995 |
| CEDG115 | X40 | 0.0148756 | 0.65918032 | 0.003996 |
| CEDG115 | X49 | 0.01124973 | 0.62907552 | 0.16949153 |
| CEDG115 | X62 | 0.01292907 | 0.6654873 | 0.01298701 |
| CEDG115 | X65 | 0.01234364 | 0.64834049 | 0.05882353 |
| CEDG115 | VR011 | 0.01335524 | 0.74644089 | 0.03389831 |
| CEDG115 | VR013 | 0.01225213 | 0.67242014 | 0.29411765 |
| CEDG115 | VR015 | 0.01311405 | 0.62839549 | 0.03030303 |
| CEDG115 | VR016 | 0.01386063 | 0.71771089 | 0.02518892 |
| CEDG115 | VR018 | 0.01299929 | 0.7272384 | 0.01567398 |
| CEDG115 | VR022 | 0.01749828 | 0.71403533 | 0 |
| CEDG115 | VR022 | 0.01332565 | 0.76138061 | 0.02695418 |
| CEDG115 | VR024 | 0.01023261 | 0.5542651 | 0.625 |
| CEDG115 | VR025 | 0.01347886 | 0.64938028 | 0.02824859 |
| CEDG115 | VR032 | 0.01248906 | 0.64676562 | 0.18518519 |
| CEDG115 | VR033 | 0.01379508 | 0.66743303 | 0.00599401 |
| CEDG115 | VR035 | 0.01430979 | 0.6544203 | 0.01428571 |
| CEDG115 | VR037 | 0.01160129 | 0.7027065 | 0.07633588 |
| CEDG115 | VR039 | 0.01349432 | 0.69500512 | 0.01094092 |
| CEDG115 | VR048 | 0.01129804 | 0.61589275 | 0.14492754 |
| CEDG115 | DQ345305 | 0.01226622 | 0.690068 | 0.1010101 |
| CEDG115 | CEDG036 | 0.01253563 | 0.63450407 | 0.00599401 |
| CEDG115 | CEDG291 | 0.01009753 | 0.6875266 | 0.23809524 |
| CEDG115 | CEDG220 | 0.01438861 | 0.69205035 | 0.00599401 |
| CEDG115 | CEDG136 | 0.01053942 | 0.66370222 | 0.34482759 |
| CEDG115 | CEDG100 | 0.01909428 | 0.76340173 | 0 |
| CEDG115 | CEDG096A | 0.01278807 | 0.7833854 | 0.001998 |
| CEDG115 | CP00361 | 0.0144935 | 0.70590899 | 0.000999 |
| CEDG115 | DMBSSR035 | 0.012208 | 0.70167341 | 0.01602564 |
| CEDG115 | CEDG084 | 0.01669123 | 0.67493089 | 0.000999 |
| CEDG115 | CEDC033 | 0.01434642 | 0.74024366 | 0 |
| CEDG115 | DMBSSR199 | 0.01291459 | 0.65287062 | 0.0952381 |
| CEDG115 | DMBSSR024 | 0.0143993 | 0.70684252 | 0.001998 |
| CEDG115 | GMES0337 | 0.01237971 | 0.65738371 | 0.11235955 |
| CEDG115 | DMBSSR001 | 0.01569785 | 0.70650816 | 0 |
| CEDG115 | CEDG118 | 0.01674119 | 0.70476475 | 0 |
| CEDG115 | CEDG146 | 0.01394965 | 0.69523762 | 0.02325581 |
| CEDG115 | CEDG225 | 0.01379718 | 0.70223166 | 0.000999 |
| CEDG115 | JMES1424 | 0.01629121 | 0.69061397 | 0.000999 |
| CEDG115 | MBSSR008 | 0.01245303 | 0.69914276 | 0.00799201 |
| CEDG115 | CEDG271 | 0.01502192 | 0.71880597 | 0.02314815 |
| CEDG115 | VM37 | 0.01196488 | 0.71124637 | 0.09615385 |
| CEDG115 | CEDG073 | 0.01488173 | 0.74976963 | 0.01438849 |
| CEDG115 | CEDG071 | 0.01655506 | 0.69811171 | 0.001998 |
| CEDG115 | CP00226 | 0.01061533 | 0.73664673 | 0.76923077 |
| CEDG115 | DMBSSR059 | 0.01427821 | 0.68822949 | 0.004995 |
| CEDG115 | VM27 | 0.01598912 | 0.76567968 | 0 |
| CEDG115 | BM212 | 0.01243725 | 0.75559791 | 0.01408451 |
| CEDG115 | CP1225 | 0.01162981 | 0.73212861 | 0.04016064 |
| CEDG115 | DMBSSR016 | 0.00987067 | 0.69880222 | 0.55555556 |
| CEDG115 | CEDG075 | 0.02548023 | 0.72004631 | 0 |
| CEDG115 | BMD2 | 0.01196278 | 0.64342902 | 0.27027027 |
| CEDG115 | BM149 | 0.01372244 | 0.69022748 | 0.00699301 |
| CEDG115 | CP08695 | 0.01272606 | 0.71269244 | 0.003996 |
| CEDG115 | CEDG008 | 0.02401169 | 0.77554168 | 0 |
| CEDG115 | CEDG256 | 0.02343578 | 0.74215912 | 0.000999 |
| CEDG115 | BM146 | 0.02761025 | 0.75538437 | 0 |
| CEDG115 | CEDG150 | 0.02270492 | 0.77746495 | 0 |
| CEDG115 | CEDG267 | 0.02472128 | 0.76415989 | 0 |
| CEDG060 | J01263 | 0.01305666 | 0.52835458 | 0.04405286 |
| CEDG060 | PV-at001 | 0.01795938 | 0.54107737 | 0 |
| CEDG060 | PV-ag003 | 0.01532789 | 0.46081258 | 0.00899101 |
| CEDG060 | PV-ag005 | 0.01345739 | 0.50804028 | 0.03164557 |
| CEDG060 | Pv-ctt002 | 0.010084 | 0.53873432 | 0.58823529 |
| CEDG060 | SSR-IAC 127 | 0.00905649 | 0.42641606 | 0.625 |
| CEDG060 | SSR-IAC 188 | 0.00945819 | 0.52013438 | 0.66666667 |
| CEDG060 | SSR-IAC 195 | 0.01841742 | 0.42871087 | 0.00599401 |
| CEDG060 | DQ469392 | 0.0131223 | 0.52463912 | 0.07352941 |
| CEDG060 | DQ469393 | 0.01357522 | 0.48970258 | 0.04587156 |
| CEDG060 | PvM03 | 0.01256177 | 0.59758277 | 0.04926108 |
| CEDG060 | PvM22 | 0.01166195 | 0.3907042 | 0.33333333 |
| CEDG060 | BMD-5 | 0.01503316 | 0.46114565 | 0.004995 |
| CEDG060 | BMD-6 | 0.0089346 | 0.28375061 | 0.52631579 |
| CEDG060 | BMD-8 | 0.01368712 | 0.41192994 | 0.03125 |
| CEDG060 | BMD-13 | 0.01227683 | 0.61053102 | 0.03067485 |
| CEDG060 | BMD-18 | 0.01542838 | 0.53876467 | 0.000999 |
| CEDG060 | BMD-23 | 0.01726117 | 0.50394707 | 0.01062699 |
| CEDG060 | BMD-26 | 0.0087863 | 0.47450936 | 0.76923077 |
| CEDG060 | BMD-29 | 0.00997785 | 0.44470341 | 0.34482759 |
| CEDG060 | BMD-31 | 0.01818813 | 0.49467004 | 0.000999 |
| CEDG060 | BMD-48 | 0.01134597 | 0.37857513 | 0.20833333 |
| CEDG060 | BMD-35 | 0.01486065 | 0.43927187 | 0.01228501 |
| CEDG060 | BMD-47 | 0.01130957 | 0.5106936 | 0.3030303 |
| CEDG060 | BMD-50 | 0.02193531 | 0.47461731 | 0 |
| CEDG060 | BMD-51 | 0.01264093 | 0.47486596 | 0.07142857 |
| CEDG060 | BMD-12 | 0.01175013 | 0.41875257 | 0.04464286 |
| CEDG060 | X21 | 0.01364593 | 0.49980599 | 0.0257732 |
| CEDG060 | X 34 | 0.00953603 | 0.46859947 | 0.71428571 |
| CEDG060 | X40 | 0.00868734 | 0.38574716 | 0.47619048 |
| CEDG060 | X49 | 0.01057912 | 0.39325142 | 0.55555556 |
| CEDG060 | X62 | 0.00939134 | 0.38408527 | 0.29411765 |
| CEDG060 | X65 | 0.01437969 | 0.46298547 | 0.03448276 |
| CEDG060 | X87 | 0.00947569 | 0.54928211 | 0.66666667 |
| CEDG060 | VR011 | 0.01121423 | 0.49233393 | 0.52631579 |
| CEDG060 | VR013 | 0.00836946 | 0.41982331 | 0.47619048 |
| CEDG060 | VR015 | 0.01399314 | 0.40673335 | 0.05494505 |
| CEDG060 | VR016 | 0.00835019 | 0.43995799 | 0.90909091 |
| CEDG060 | VR018 | 0.01870937 | 0.63890097 | 0 |
| CEDG060 | VR022 | 0.00938624 | 0.43930459 | 0.34482759 |
| CEDG060 | VR022 | 0.01355886 | 0.60116669 | 0.01166861 |
| CEDG060 | VR024 | 0.01004906 | 0.36332213 | 0.27027027 |
| CEDG060 | VR025 | 0.0091073 | 0.37600122 | 0.83333333 |
| CEDG060 | VR032 | 0.01496971 | 0.42781972 | 0.00599401 |
| CEDG060 | VR033 | 0.01280798 | 0.4327303 | 0.32258065 |
| CEDG060 | VR035 | 0.01348815 | 0.40598201 | 0.28571429 |
| CEDG060 | VR037 | 0.01188583 | 0.51609698 | 0.07751938 |
| CEDG060 | VR039 | 0.0134698 | 0.49477016 | 0.00699301 |
| CEDG060 | VR048 | 0.01417357 | 0.43438819 | 0.02762431 |
| CEDG060 | DQ345305 | 0.01114874 | 0.48031635 | 0.41666667 |
| CEDG060 | CEDG036 | 0.01611216 | 0.44002119 | 0.003996 |
| CEDG060 | CEDG291 | 0.01446093 | 0.57559398 | 0.003996 |
| CEDG060 | CEDG220 | 0.01290861 | 0.46795771 | 0.04065041 |
| CEDG060 | CEDG136 | 0.02327782 | 0.59415601 | 0 |
| CEDG060 | CEDG100 | 0.01898745 | 0.62710435 | 0 |
| CEDG060 | CEDG096A | 0.01417595 | 0.63515342 | 0 |
| CEDG060 | CP00361 | 0.01242704 | 0.44901545 | 0.16129032 |
| CEDG060 | DMBSSR035 | 0.01358122 | 0.52171128 | 0.004995 |
| CEDG060 | CEDG084 | 0.01352643 | 0.44381595 | 0.07142857 |
| CEDG060 | CEDG185 | 0.020084 | 0.62211649 | 0 |
| CEDG060 | CEDC033 | 0.01258048 | 0.53308133 | 0.32258065 |
| CEDG060 | DMBSSR199 | 0.01129549 | 0.39390239 | 0.625 |
| CEDG060 | DMBSSR024 | 0.01356532 | 0.46276732 | 0.1369863 |
| CEDG060 | GMES0337 | 0.01273589 | 0.45103665 | 0.04081633 |
| CEDG060 | DMBSSR001 | 0.014846 | 0.46577465 | 0.02392344 |
| CEDG060 | CEDG118 | 0.01137818 | 0.45291434 | 0.15384615 |
| CEDG060 | CEDG146 | 0.01454276 | 0.48687385 | 0.02617801 |
| CEDG060 | CEDG225 | 0.01482686 | 0.5455594 | 0.02688172 |
| CEDG060 | JMES1424 | 0.01897414 | 0.51974553 | 0 |
| CEDG060 | MBSSR008 | 0.01473196 | 0.54605127 | 0 |
| CEDG060 | CEDG271 | 0.01462278 | 0.55060626 | 0.0203666 |
| CEDG060 | VM37 | 0.01703648 | 0.53158441 | 0.001998 |
| CEDG060 | CEDG073 | 0.01112654 | 0.46718997 | 0.12987013 |
| CEDG060 | CEDG071 | 0.01287199 | 0.51763691 | 0.0265252 |
| CEDG060 | CP00226 | 0.01314694 | 0.65626849 | 0.01464129 |
| CEDG060 | DMBSSR059 | 0.01936195 | 0.49376072 | 0.002997 |
| CEDG060 | VM27 | 0.01290754 | 0.56484693 | 0.01422475 |
| CEDG060 | BM212 | 0.01202749 | 0.55764495 | 0.32258065 |
| CEDG060 | CP1225 | 0.01279895 | 0.57194986 | 0.04651163 |
| CEDG060 | DMBSSR016 | 0.01053244 | 0.50680191 | 0.27777778 |
| CEDG060 | CEDG075 | 0.01643089 | 0.5102017 | 0.00599401 |
| CEDG060 | BMD2 | 0.01593165 | 0.47208234 | 0.10752688 |
| CEDG060 | BM149 | 0.01647222 | 0.53818654 | 0.00699301 |
| CEDG060 | CP08695 | 0.01106009 | 0.448901 | 0.37037037 |
| CEDG060 | CEDG008 | 0.0204847 | 0.60683316 | 0 |
| CEDG060 | CEDG256 | 0.02289483 | 0.58870804 | 0 |
| CEDG060 | BM146 | 0.01282891 | 0.50706949 | 0.11494253 |
| CEDG060 | CEDG050 | 0.01373364 | 0.54058975 | 0.06535948 |
| CEDG060 | CEDG150 | 0.01521334 | 0.58998295 | 0.000999 |
| CEDG060 | CEDG267 | 0.01515073 | 0.5208675 | 0.02985075 |
| CEDG060 | CEDG115 | 0.01277346 | 0.63810326 | 0.00899101 |
